# A Reverse Engineering Approach to the Suppression of Citation Biases Reveals Universal Properties of Citation Distributions

**DOI:** 10.1371/journal.pone.0033833

**Published:** 2012-03-29

**Authors:** Filippo Radicchi, Claudio Castellano

**Affiliations:** 1 Departament d'Enginyeria Quimica, Universitat Rovira i Virgili, Catalunya, Spain; 2 Howard Hughes Medical Institute (HHMI), Northwestern University, Evanston, Illinois, United States of America; 3 Department of Chemical and Biological Engineering, Northwestern University, Evanston, Illinois, United States of America; 4 Istituto dei Sistemi Complessi (ISC-CNR), Italy; 5 Dipartimento di Fisica, “Sapienza” Università di Roma, Roma, Italy; Rensselaer Polytechnic Institute, United States of America

## Abstract

The large amount of information contained in bibliographic databases has recently boosted the use of citations, and other indicators based on citation numbers, as tools for the quantitative assessment of scientific research. Citations counts are often interpreted as proxies for the scientific influence of papers, journals, scholars, and institutions. However, a rigorous and scientifically grounded methodology for a correct use of citation counts is still missing. In particular, cross-disciplinary comparisons in terms of raw citation counts systematically favors scientific disciplines with higher citation and publication rates. Here we perform an exhaustive study of the citation patterns of millions of papers, and derive a simple transformation of citation counts able to suppress the disproportionate citation counts among scientific domains. We find that the transformation is well described by a power-law function, and that the parameter values of the transformation are typical features of each scientific discipline. Universal properties of citation patterns descend therefore from the fact that citation distributions for papers in a specific field are all part of the same family of univariate distributions.

## Introduction

The use of bibliographic databases plays a practical, and crucial, role in modern science. Citations between scientific publications are in fact commonly used as quantitative indicators for the importance of scientific papers, as proxies for the influence of publications in the scientific community. General criticisms to the use of citation counts have been made [1]–[3], and the real meaning of a citation between papers can be very different and context dependent [4]. Nevertheless, a citation can be viewed as a tangible acknowledgment of the citing paper to the cited one. Thus, the more citations a paper has accumulated, the more influential the paper can be considered for its own scientific community of reference. The same unit of measure (i.e., a citation) is commonly used as the basis for the quantitative evaluation of individual scholars [5], [6], journals [7], departments [8], universities and institutions [9], and even entire countries [10]. Especially at the level of individual scientists, numerical indicators based on citation counts are evaluation tools of fundamental importance for decisions about hiring [11] and/or grant awards [12].

As a matter of fact, citation practice is widespread, still basic properties of citation patterns are not completely clear. For example, we know that citations are broadly distributed, but, we do not know the exact functional form of citation distributions. In his seminal paper, de Solla Price proposed a power-law model for explaining how papers accumulate citations [13]. However, more recent studies indicate several, sometimes very different, possibilities: power-laws [14], [15], stretched exponentials [16], [17], log-normals [18]–[20], and modified Bessel functions [21].

At the same time, it is common practice to attribute the same value to each citation, in spite of the fact that citation counts strongly depend on the field [22]. For example, a paper in mathematics typically gets less citations than a paper in molecular biology. There are in fact large variations among scientific communities, mostly related to the different citation habits of each community. Such disproportions show up in the typical values of the most common bibliometric indicators based on raw citation counts. The most influential journal in mathematics, *Annals of Mathematics*, has impact factor [7] roughly equal to 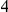 according to the 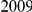 edition of the Journal Citation Reports (JCR) database [23], while its counterpart in molecular biology, *Cell*, has impact factor 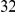, eight times larger. Similarly, there are several chemists with 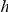-index [5] larger than 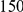 [24], while for a computer scientist it is very hard to have an 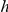-index larger than 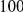 [25]. Notice that the values of the 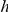-index for chemists have been calculated in 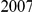, while those for computer scientists in 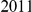. For the same year of reference, we should expect that the difference is even larger than what reported here. Such disproportions in citation counts make the use of raw citation numbers very precarious in many cases and call for alternative, more fair, measures. It is important to stress that in this paper we denote as “bias” the the systematic error that is introduced when using raw citation numbers to compare papers belonging to different fields. With this term we do not indicate any prejudice, nor we make any claim about the causes of the field dependence empirically observed.

Although methods based on percentile ranks have been recently considered [26], [27], the traditional approach to the suppression of field-dependence in citation counts is based on normalized indicators. The raw number of citations is divided by a discipline dependent factor, and the aim of this linear transformation is to suppress eventual disproportions among the citation patterns of different research fields. Various methods have been proposed using this kind of approach [28]–[32]. In this context, particularly relevant is the study performed in [19] (based on the the relative indicator originally developed in [33]), where citation distributions of different scientific disciplines are shown to have the same functional form, differing only for a single scaling factor (the average number of citations received by papers within each scientific discipline). The study is, however, limited to a small number of papers and scientific disciplines, and therefore not conclusive. The same approach of [19] has also been used for more refined classification of publications in physics [34] and chemistry [35], showing in general a good agreement with the previous claim of [19]. More recently, Albarrán *et al.* [36] and Waltman *et al.* [37] have analyzed much larger datasets of scientific publications, and showed that the result of [19] holds for many but not for all scientific disciplines. These studies cast some doubts on the validity of the results in [19], but, on the other hand, do not propose any alternative method for bias suppression.

Here, we perform an exhaustive analysis of about 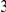 millions of papers published in six different years (spanning almost 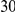 years of scientific production) and in more than 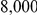 journals listed in the Web Of Science (WOS) [38] database. We use the classification of journals in subject-categories (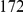 in total) as defined in the 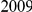 edition of the Journal Citation Reports (JCR) database [23], and systematically study the patterns of citations received by papers within single subject-categories. Despite some journals cover a rather broad range of topics, a subject-category is a relatively accurate classification of the general content of a journal. Examples of JCR subject-categories are “Mathematics”, “Reproductive Biology” and “Physics, Condensed Matter”. Subject-categories can be considered as good approximations for scientific disciplines.

We propose a transformation of raw citations numbers such that the distributions of transformed citation counts are the same for all subject-categories. We study the properties of this transformation and find strong regularities among scientific disciplines. The transformation is almost linear for the majority of the subject-categories. Exceptions to this rule are present, but, in general, we find that all citation distributions are part the same family of univariate distributions. In particular, the rescaling considered in [19], despite not strictly correct, is a very good approximation of the transformation able to make citation counts not depending on the scientific domain.

## Results

### Modeling citation distributions

**Figure 1:**
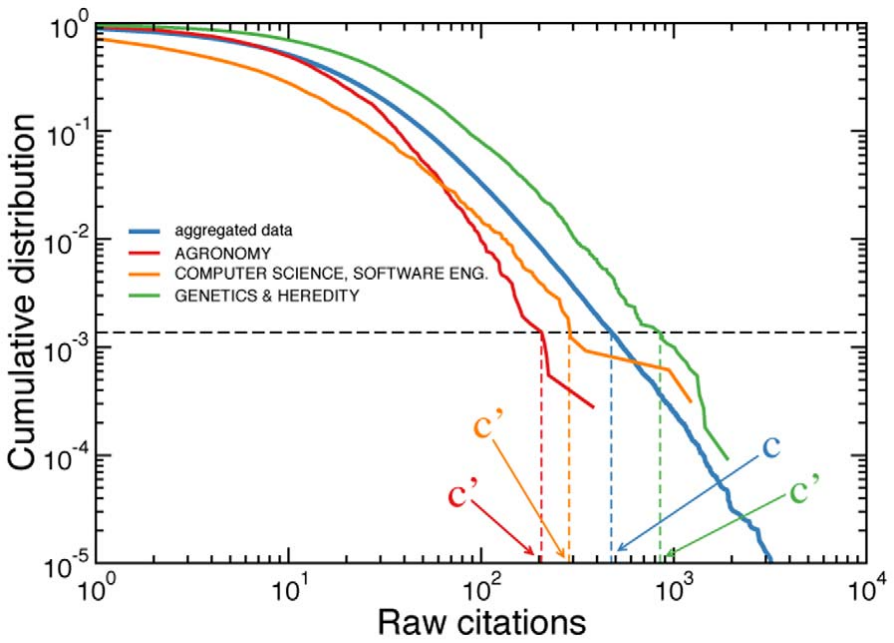
Cumulative distribution of raw citation counts for papers published in 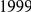
 **.** The blue curve is calculated by aggregating all papers of all subject-categories (average number of citations 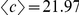). The red curve, the orange curve and the green curve are calculated by considering only papers within the subject-categories “Agronomy” (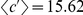), “Computer science, software engineering” (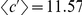) and “Genetics & heredity” (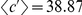), respectively. The figure illustrates the mapping of 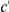 into 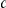. Citation counts 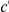 of single subject-categories are matched with the value of 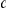 which corresponds to same value of the cumulative distributions.

For the same year of publication, the raw citation patterns of single subject-categories may be very different. Variations are a consequence of different publication and citation habits among scientific disciplines. In Fig. 1 for example, we plot the cumulative distributions of citations received by papers published in journals belonging to three different subject-categories. The shape of the three cumulative distributions is not exactly the same, and the difference is not accounted for by a single scaling factor [19]. Dividing raw citation counts by a scaling factor (e.g., the average number of citations of the subject-category) would in fact correspond, in the logarithmic scale, to a horizontal rigid translation of the cumulative distribution. However, as Fig. 1 shows, this linear transformation is not sufficient to make all cumulative distributions coincide. By looking at the figure, the cumulative distributions of the raw citation counts for papers published in journals within the subject-categories “Computer science, software engineering” and “Genetics & heredity” have a pretty similar shape, and thus the possibility to obtain a good collapse of the curves by simply rescaling citation counts seems reasonable. Conversely, the cumulative distribution of the citations received by papers published in journals of the subject-category “Agronomy” has a different shape. The curve bends down faster than the curves corresponding to the other two subject-categories. In this case, a linear transformation of citation counts would hardly help to make this curve coincide with the others. Making citation counts independent of the subject-categories seems therefore not possible with the use of linear transformations, because the difference between citation distributions of different subject-categories is not only due to a single scaling factor.

In order to make further progress, here we invert the approach to the problem. We know that citation patterns of single subject-categories may be different, but we do not know how to transform citation counts in order to make them similar. We implement therefore a mapping able to make all cumulative distributions coincide, and study the properties of this transformation. We use a sort of “reverse engineering” approach: instead of introducing a transformation and checking whether it works, here we impose that the transformation must work and from this assumption we derive its precise form.

**Figure 2:**
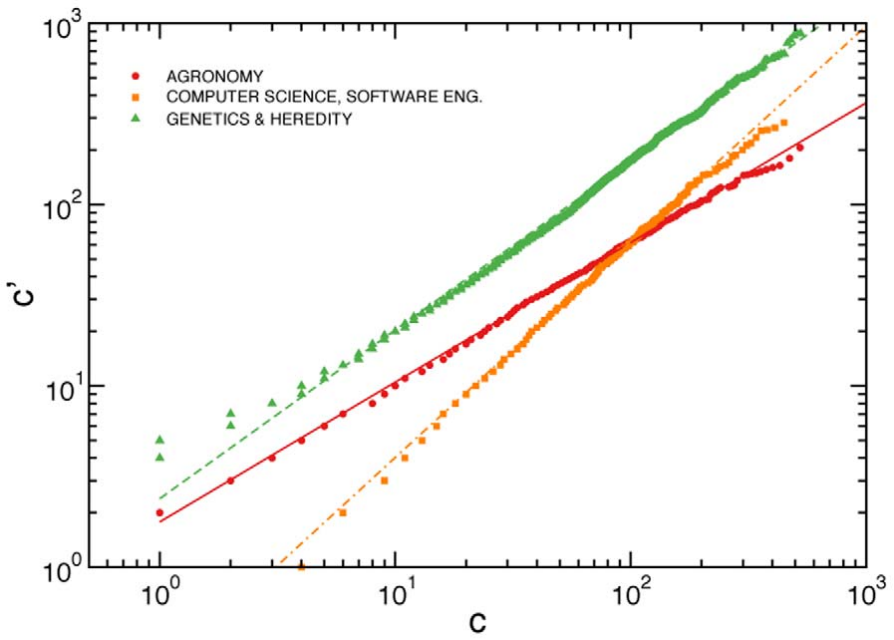
Transformation of citation counts. Citations within single subject-categories. 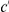 are plotted against citation counts of the aggregated data 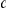. The quantities 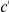 and 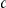 are related by a power-law relation (Eq. 1). Different subject-categories have different values of the transformation factor 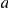 and the transformation exponent 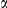. The best estimates of 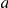 and 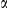 for the subject-categories considered in this figure (the same subject-categories as those appearing in Fig. 1) are: 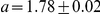 and 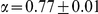 for “Agronomy”, 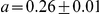 and 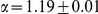 for “Computer science, software engineering”, 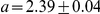 and 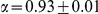 for “Genetics & heredity”. The results of the complete analysis for all subject-categories and years of publication are reported in the Supporting Information S2, S3, S4, S5, S6, and S7.

The idea is pretty simple and straightforward. We use as curve of reference the cumulative distribution 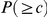 of raw citation counts 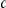 obtained by aggregating together all subject-categories (see Fig. 1). The choice of the curve of reference is in principle arbitrary, and affects the explicit form of the transformation. The use of the aggregated dataset as reference seems, however, a very reasonable choice because it does not require the introduction of any parameter. In general, other choices for the reference curve are possible, but the only important constraints are (i) using the same system of reference for all subject-categories and (ii) producing a mapping that preserves the natural order of citation counts within the same subject-category. We then focus on a specific subject-category 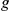, and consider the cumulative distribution 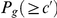 of the raw citations 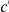 received by papers published in journals within subject-category 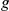. To each value of 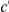, we associate a single value of 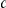 in the system of reference, where 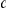 is determined as the value for which 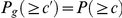. In practice, we implement the mapping by sorting in ascending order all citation counts of the 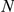 papers present in the aggregated dataset, and then by associating to each different value of 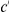, in the dataset of subject-category 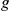, the value of 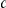 that appears in the 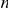-th position of the sorted list, with 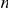 equal to the integer value closest to 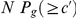. In this procedure, different values of 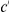 may correspond to the same value of 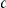. Such event is more likely to happen for low values of 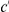, while, for large values of 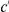, the mapping is always unique (see Fig. 2).

The plot 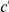 *vs.* 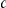 is equivalent to a quantile-quantile (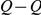) plot, a graphical non-parametric method generally used for comparing two probability distributions [39]. If the comparison is made between two samples of randomly and identically distributed variates, all points in the corresponding 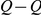 plot, should approximately lay on the line 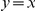. If the difference between the two samples is just a scaling factor 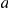, then all points in the 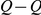 plot should instead lay on the line 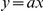. Very interestingly in the case of citation distributions, we empirically find that the relation between 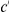 and 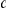 can be described by a power-law function
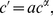
(1)where 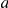 and 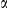 are respectively the pre-factor and the exponent of the mapping (see Fig. 2). The functional form of Eq. 1 holds for virtually all subject-categories and all publication years considered in this study (see Supporting Information S2, S3, S4, S5, S6, and S7). Few exceptions are present, the most noticeable represented by the hybrid subject-category “Multidisciplinary sciences”.

The citation distributions of the subject-categories for which Eq. 1 holds are univariate distributions belonging to the same log-location-scale family [40]. A log-location-scale family of distributions is a class of distributions 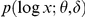 of continuous variables 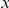 that can be rewritten in terms of the same reference distribution 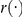 as 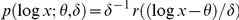, for any choice of the location parameter 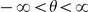 and the scale parameter 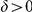 [41]. Citation distributions are defined for discrete variables, but still according to Eq. 1 we can write 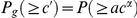, where 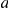 and 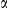 respectively represent the log-location and the log-scale parameters. In few words, our empirical finding tells us that citation distributions are part of the same log-location-scale family of discrete distributions. Weibull and log-normal distributions are well known log-location-scale families.

### Cumulative distribution of transformed citations

**Figure 3:**
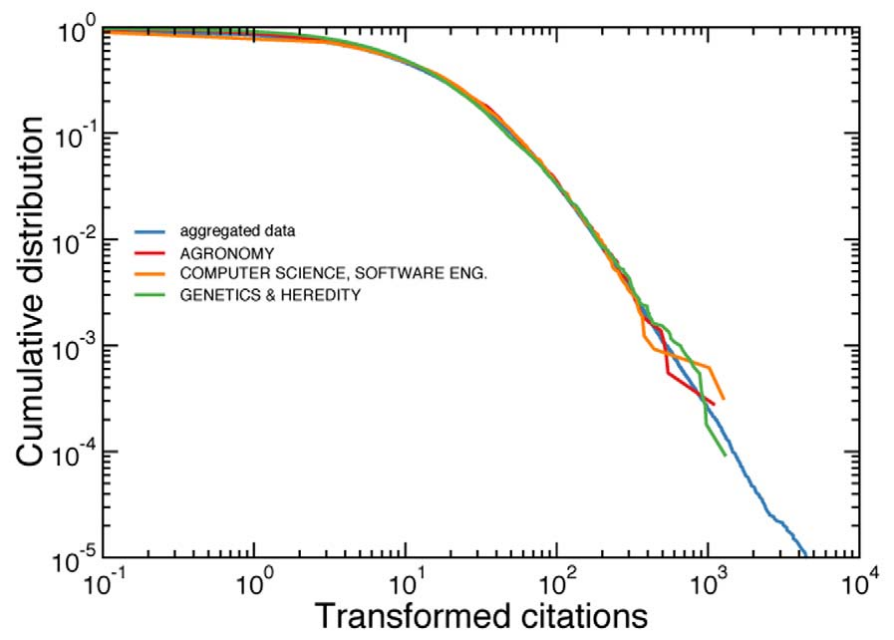
Cumulative distribution of the transformed citation counts. When raw citation numbers are transformed according to Eq. 2, the cumulative distributions of different subject-categories become very similar. All citation distributions are mapped on top of the cumulative distribution obtained by aggregating all subject-categories together (the common reference curve in the transformation). We consider here the same subject-categories as those considered in Figs. 1 and 2. The complete analysis of all subject-categories and years of publication is reported in the Supporting Information S2, S3, S4, S5, S6, and S7.

By definition, the transformation 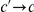 maps the cumulative distribution on top of the cumulative distribution of reference (i.e., the one calculated for the aggregated data). Therefore, if the same transformation is applied to the citation numbers of all subject-categories, all cumulative distributions concide, providing a systematic deletion of differences present in the citation patterns. Eq. 1 tells us that the mapping 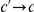 is simple. The citations 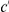 received by papers published in journals within a specific subject-category can be simply transformed as
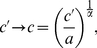
(2)if we want to make all citation distributions of single subject-categories coincide with the cumulative distribution of reference. Fig. 3 shows the cumulative distributions resulting after the application of Eq. 2. The cumulative distributions of the transformed citation counts are very similar. Small deviations are still visible at low values of the transformed citation counts, when the discreteness of citation numbers become more important.

### Quantitative test of bias suppression

The fact that all cumulative distributions of transformed citation counts coincide seems able to place all subject-categories on the same footing: when raw citations are transformed according to Eq. 2, the fraction of papers with a given value of the transformed citation counts is almost the same for all subject-categories. To quantitatively assess such a qualitative result, we perform an additional test. The aim of the transformation of Eq. 2 is to suppress inevitable biases in raw citation counts among subject-categories, and thus we compare our results with the outcome expected in the absence of biases.

The situation can be modeled in the following terms. We aggregate all papers of all subject-categories together, and extract the top 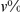 of publications according to the value of their transformed citations. We then compute the proportion of papers in each subject-category that are part of the top 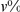 Assuming all cumulative distributions to be the same, we expect these proportions to have values close to 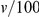. However, since the number of papers in each subject-category is finite, the proportions of papers belonging to the top 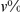 are affected by fluctuations, which can be precisely computed (see the Methods section for details). By checking if the outcome of our selection process is compatible with the results expected assuming a random and unbiased selection process, we test whether we have effectively removed citation biases.

**Figure 4:**
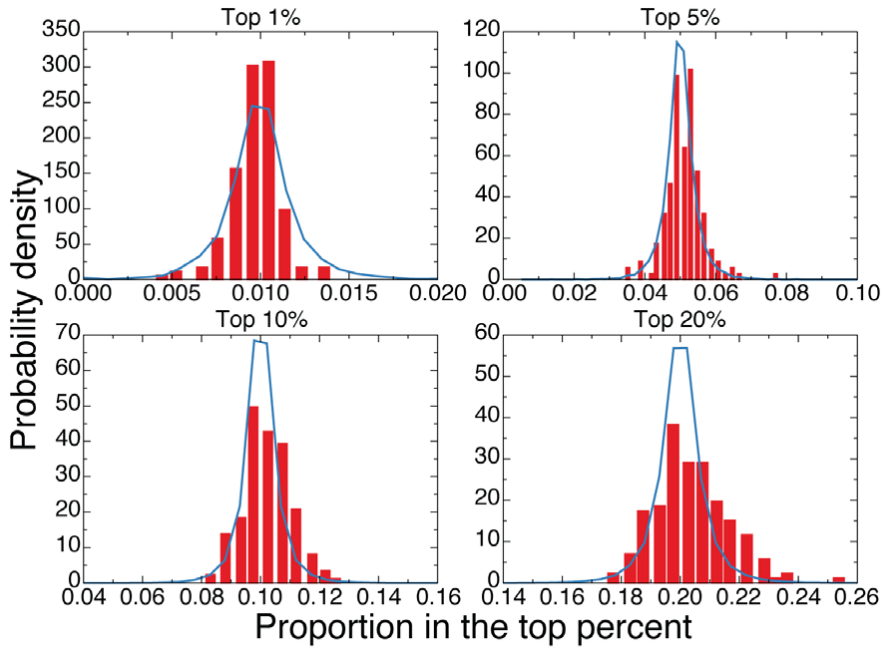
Comparison between expected and observed proportions of top cited papers. Probability density function of the proportion of papers belonging to a particular subject-category and that are part of the top 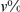 of papers in the aggregated dataset. Red boxes are computed on real data, while blue curves represent the density distributions valid for unbiased selection processes. We consider different values of 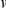: 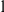, 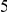, 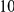 and 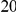. These results refer to papers published in 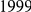.

**Figure 5:**
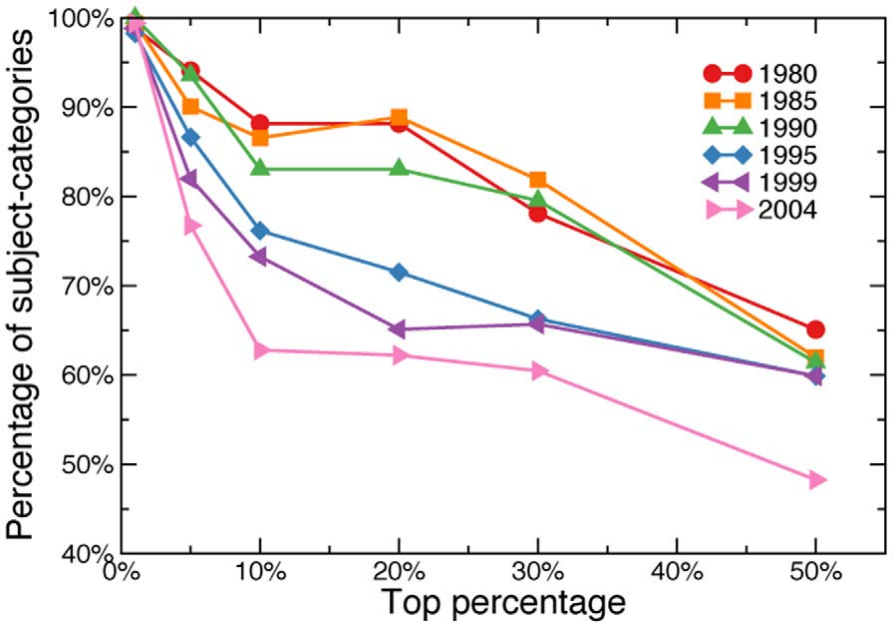
Effectiveness of the proposed normalization technique. Percentage of subject-categories whose proportion values, after normalization, fall into the 95% confidence interval of values predicted in our null model. Percentage values are plotted as functions of the percentage 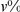 of top papers considered in the analysis. We plot separate curves for different publication years.

The results of this analysis are reported in Fig. 4 for papers published in 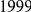, and in the Supporting Information S1, S2, S3, S4, S5, S6, and S7 for other publication years. In general, the transformation of Eq. 2 produces, for all years of publication, results that are consistent with an unbiased selection process, if 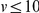 (see Fig. 5). For the most relevant part of the curve (i.e., highly cited papers), the simple transformation of Eq. 2 effectively removes systematic differences in citation patterns among subject-categories. Conversely, for higher values of 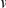, the discreteness of citation numbers becomes more relevant, the power-law mapping of Eq. 1 becomes less descriptive, and the distribution of the proportion of top 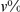 papers measured for real data, despite still centered around the expected value, is wider than expected. The results are even better for papers published before year 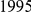 because the comparison between observed and expected proportions of papers in the top 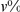 is very good up to 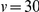. The reason could be due to a higher stability of citation patterns for all subject-categories, since all papers have had more than 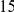 years to accumulate citations [18].

### Values of the transformation parameters

**Figure 6:**
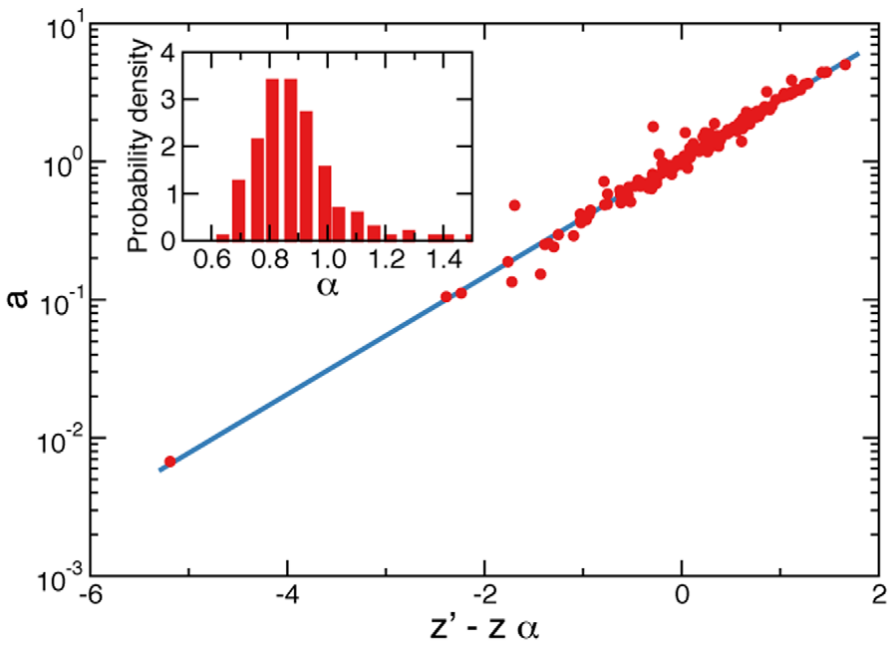
Properties of the transformation parameters. In the inset, we report the density distribution of the transformation exponents 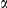 calculated for all subject-categories. In the main plot, we show the relation between the transformation exponent 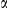, the transformation factor 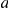, and the parameters 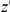 and 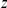 for the same data points as those appearing in the inset. The relation between the various quantities is fitted by the function 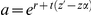, with 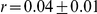 and 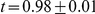 (blue line). Both plots have been obtained by analyzing papers published in 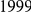, but the same results are valid also for different years of publications as shown in Figs. S115 and S116.

The values of the transformation factor 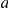 and the transformation exponent 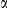 for the same subject-category are pretty stable when measured over different years of publication. In particular, the value of 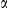 is very robust, suggesting that the shape of the cumulative distribution of single subject-categories does not vary with time. For example, over a span of almost 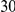 years, the values 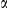 for the subject-category “Agronomy” range in the interval 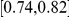, for “Computer science, software engineering” range in the interval 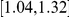, and for “Genetics & heredity” range in the interval 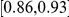. Tables reporting the complete results for all subject-categories and publication years can be found in the Supporting Information S2, S3, S4, S5, S6, and S7. The density distribution of the transformation exponents is peaked around 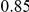 which means that the shape of the distributions is in the majority of the cases the same and the only difference is a scaling factor (see inset of Fig. 6 and Supporting Information S8).

Moreover, the transformation factor 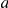 and the transformation exponent 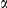 are related. Let us consider what happens for log-normal distributions. A log-normal distribution is given by 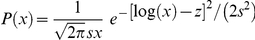, where 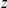 and 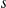 are the parameters of the distribution. The parameters 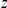 and 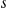 are related to the mean 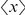 and variance 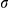 of the distribution: 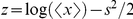 and 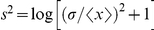. A 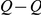 plot between two log-normal distributions with parameters 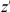 and 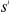, and 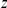 and 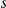, respectively, shows a perfect power-law scaling as the one given by Eq. 1. In this case, 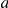 and 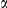 are related to the parameters of the distributions by
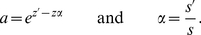
(3)We checked whether Eq. 3 is valid also in the case of the citation distributions considered here. In Fig. 6, we show the results obtained for publication year 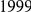, while the plots for the other publication years are reported in Supporting Information S8. In general for citation distributions, Eq. 3 should be generalized to 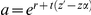, with small but non vanishing values of 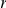 and values of 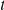 slightly different from one. We conclude that the citations for single subject-categories are distributed almost log-normally and this reflects in the values of transformation parameters.

The 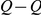 plot between two log-normal distributions helps also understanding why the typical values of 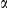 are generally smaller than one (inset of Fig. 6 and Supporting Information S8). According to our choice, the reference distribution is given by the aggregation of all subject-categories, and this means that the variance of the resulting distribution is mainly determined by those of the subject-categories with higher variances. For the majority of the subject-categories we have 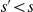, that is 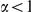.

## Discussion

The practical importance of citation counts in modern science is substantial, and growing. Citation numbers (or numerical indicators derived from them) are commonly used as basic units of measure for the scientific relevance not only of papers, but also of scientists [5], [6], journals [7], departments [8], universities and institutions [9], and even entire countries [10]. Citations are direct measures of popularity and influence, and the use of citation numbers is a common evaluation tool for awarding institutional positions [11] and grants [12]. Unfortunately, the direct use of raw citations is in most of the cases misleading, especially when applied to cross-disciplinary comparisons [22]. Citations have different weights depending on the context where they are used, and proper scales of measurements are required for the formulation of objective quantitative criteria of assessment. Saying that a paper in biology is more influential than a paper in mathematics, only because the former has received a number of citations three times larger than the latter, is incorrect. Differences in publication and citation habits among scientific disciplines are reflected in citation and publication counts, and generally cause disproportions that favor disciplines with higher publication and citation rates with respect to those disciplines where publications and citations are created at slower rates. In a certain sense, the situation is similar to the comparison of the length of two streets, one long three and the other two, but without knowing that the length of the first is measured in kilometers while the other in miles.

Differences in citation patterns among scientific domains have been known for a long time [22] and several attempts to the suppression of discipline dependent factors in raw citation counts have been already proposed in the past [19], [28]–[32]. The most common methodology consists in dividing citation counts by a constant factor, and thus replacing raw with normalized citation numbers. Each normalization procedure is, however, based on some assumption. Scientific disciplines differ not only in citation numbers, but also in publication numbers, length of references and author lists, etc. A universal criterion for the complete suppression of differences among scientific domains probably does not exist. There are too many factors to account for, and consequently the “philosophy” at the basis of a “fair” normalization procedure is subjective. The formulation of the so-called fractional citation count is, for example, based on a particular idea of fairness [32]. Citations are normalized by assigning to each citation originated by a paper a weight equal to the inverse of the total number of cited references in that paper. According to this procedure, the weight of each published paper equals one, but disciplines with higher publication rates are still favored when compared with disciplines with lower publication rates.

In this paper, we consider a different notion of fairness, based on the reasonable but strong assumption that each discipline or field of research has the same importance for the development of scientific knowledge. A fair numerical indicator, based on citation numbers, must then assume values that do not depend on the particular scientific domain under consideration. Under this assumption, the probability to find a paper with a given value of the fair indicator must not depend on the discipline of the paper, or equivalently, the distribution of normalized indicators must be the same for all disciplines. It is clear that our notion of fairness strongly depends on the classification of papers into categories (disciplines, fields, topics). Also, it is important to remark that other possible definitions of fairness could be stated, without relying on the assumption that each discipline or research field has the same importance for scientific development.

We have then proposed a simple but rigorous method for the implementation of our notion of fairness. We have studied the citation patterns of papers published in more than 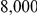 scientific journals. Our analysis covers six different years of publication, spanning over almost 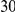 years of scientific production, and includes three millions of papers. We have found strong regularities in how citations are attributed to papers dealing with similar scientific topics of research (i.e., subject-categories). In particular, we have introduced a simple mapping able to transform the citation distribution of papers published within specific subject-categories into the same distribution. Very interestingly, the transformation turns out to be described by a power-law function, which depends on two parameters (pre-factor and exponent). Each specific subject-category is characterized by its parameters, which are stable over different publication years. For the vast majority of the subject-categories, the power-law exponent assumes approximately the same value suggesting that the main difference between the citation distribution of different subject-categories is given only by a scaling factor. There are, however, subject-categories for which the transformation is not a power-law function. In general, these are hybrid subject-categories, as for example “Multidisciplinary sciences”, or not so well defined subject-categories, as for example “Engineering, petroleum” or “Biodiversity conservation”. In the latter cases, the subject-categories are not well defined because papers within these subject-categories are also part of other broader subject-categories. Since the classification of JCR is made at journal level, papers published in multi-category journals are automatically attributed to more subject-categories. In this way for example, 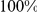 of papers published in 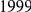 in journals within the subject-category “Biodiversity conservation” are also part of “Ecology”, and 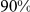 of papers published in 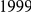 within “Engineering, petroleum” are also part of “Energy & fuels”. These observations cast some doubts regarding the classification of JCR, which probably requires serious revisions, especially because it seems that the classification places on the same footing very broad subject-categories and more specific ones. Despite that, the results reported in this paper support the claim that citation distributions are universal, in the sense that they are all part of the same family of univariate distributions (i.e., a log-location-scale family [40], [41]). Each citation distribution can be obtained from the same reference distribution with the only prescription of transforming the logarithm of its argument with suitably chosen location and scale parameters. The transformation generalizes therefore the rescaling of [19], that can be considered a good approximation of the full transformation able to suppress field-dependent differences in citation patterns.

In general, all results obtained in this paper could seem to be explained by assuming that the citations received by papers in each subject-category are continuous variables obeying log-normal distributions. However, this is only approximately true. First, citations are, by definition, non negative discrete numbers. Secondly, even assuming their discreteness, the distribution of citations received by papers within the same subject-category is not statistically consistent with a discrete log-normal distribution. We systematically tested this hypothesis for all subject-categories and publication years, and found that the log-normality of citation distributions cannot be rejected only for a very limited number of subject-categories (see Tables in Supporting Information S9). For papers published in 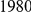, 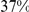 of the subject-categories have distributions consistent with log-normals (at 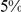 significance level). This proportion, however, decreases for more recent years of publication: 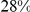 in 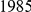, 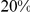 in 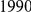, 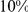 in 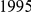, 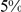 in 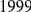 and 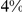 in 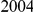. While the number of citations received by papers published in the same year and journal are log-normally distributed [18], [20], we should not expect the same for subject-categories. Subject-categories are given by the aggregation of more journals, and the convolution of many log-normals with different averages and variances is not necessarily a log-normal distribution.

We believe that the methods and results reported in this paper can be of great relevance for the entire scientific community. Citation counts and measures based on citations are powerful tools for the quantitative assessment of science, especially in our modern era in which millions of individuals are involved in research but decisions (i.e., allocation of funds) need to be quickly taken. The use of citations is already a common practice, and in the near future will become a necessity. As individuals directly involved in this business, we should therefore develop the best methodologies able to avoid the misuse of citation numbers.

## Materials and Methods

### Datasets

We considered papers published in six distinct years: 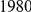, 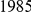, 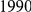, 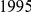, 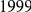 and 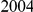. We downloaded from the WOS database [38] a total of 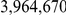 documents published in 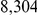 scientific journals. Journal titles have been obtained from [23], and correspond to all journals classified in at least one subject-category by the 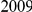 edition of JCR. According to the JCR classification, a journal may be classified in more than one subject-category. For example, the journal Physical Review D is classified in the subject-categories “Astronomy & astrophysics” and “Physics, particles & fields”. It is also important to stress that JCR classification is made at journal level, and thus does not allow a proper distinction of papers in research topics, whenever papers are published in multi-category journals. In this respect, we adopted, for simplicity, a multiplicative strategy, in which papers published in multi-category journals are simultaneously associated with all corresponding subject-categories. We considered only documents written in “English”, and classified as “Article”, “Letter”, “Note” or “Proceedings Paper”. We obtained a total of 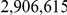 publications on which we based our study. More in detail, we considered in our study 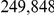 documents published in 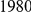, 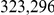 in 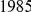, 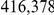 in 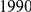, 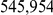 in 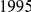, 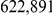 in 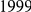 and 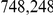 in 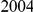. Summary tables regarding the proportion of documents written in different languages and about the types of published material can be found in the Supporting Information S1. We included in our analysis both cited and uncited publications. The information about the number of cites received by each publication was obtained from the WOS database (field “time cited”) between May 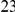 and May 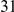, 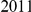.

### Test of bias suppression

The statistical test proposed here is very similar to the one introduced in [42]. The unbiased selection of papers is equivalent to a simple urn model [43], where papers (marbles) of different subject-categories (colors) are randomly extracted, one by one, without replacement. The total number of papers in the urn is 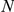, each subject-category 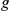 is represented by 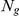 papers, and the total number of extracted papers is 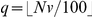. The number 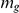 of papers of subject-category 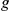, extracted in the unbiased selection process, is a random variate obeying a univariate hypergeometric distribution. The proportion of papers of subject-category 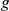 is still distributed in the same way, with the only difference of the change of variable 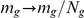 [if 
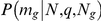
 indicates the hypergeometric distribution, the fraction 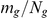 obeys the distribution 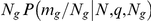]. Similarly, the joint distribution of the number of papers 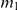, 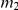, …, 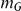, belonging respectively to subject-categories 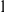, 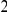, …, 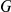 and that have been extracted in the unbiased selection, obey a multivariate hypergeometric distribution. In principle, one could calculate the expected distribution for the proportions of papers belonging to each subject-category and that are part of the top 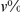, namely 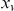 by considering all possible extractions 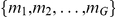, weighting each extraction with the multivariate hypergeometric distribution, and counting how many times in each extraction the quantity 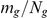 (for all subject-categories 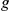) equals 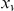. In practice, it is much simpler to simulate many times (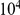 times in our analysis) the process of unbiased selection, and obtain a good approximation of the probability density of the proportions of papers present in the top 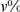. This probability density represents the correct term of comparison for what observed in real data, and furnishes a quantitative criterion for the assessment of whether the transformation of Eq. 2 is able to suppress subject-category biases in citation counts or not.

## Supporting Information

Supporting Information S1 **Publication types and language of publications.**
(PDF)

Supporting Information S2
**Complete analysis for publication year** 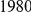 **.**
(PDF)

Supporting Information S3
**Complete analysis for publication year** 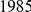 **.**
(PDF)

Supporting Information S4
**Complete analysis for publication year** 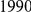 **.**
(PDF)

Supporting Information S5
**Complete analysis for publication year** 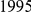 **.**
(PDF)

Supporting Information S6
**Complete analysis for publication year** 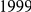 **.**
(PDF)

Supporting Information S7
**Complete analysis for publication year** 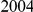 **.**
(PDF)

Supporting Information S8 **Summary figures for all years of publication.**
(PDF)

Supporting Information S9 **Log-normal fit of the citation distributions.**
(PDF)
