# Supplementary material for: A Reverse Engineering Approach to the Suppression of Citation Biases Reveals Universal Properties of Citation Distributions
Source: PLoS One. 2012 Mar 29;7(3):e33833. doi: 10.1371/journal.pone.0033833 (PMC3315498; doi:10.1371/journal.pone.0033833)
Supplement: Supporting Information S1 — Publication types and language of publications. (PDF) [file pone.0033833.s001.pdf]

Pub. type	Number of pub.	Percentage of pub.
Art Exhibit Review	1	0.00%
Article	222, 223	66.25%
Biographical-Item	3	0.00%
Book Review	3, 206	0.96%
Chronology	1	0.00%
Correction	10	0.00%
Correction, Addition	2, 744	0.82%
Discussion	687	0.20%
Editorial Material	8, 720	2.60%
Item About an Individual	117	0.03%
Letter	19, 491	5.81%
Meeting Abstract	48, 489	14.46%
Note	25, 596	7.63%
Review	4, 133	1.23%
**Total**	**335, 421**	**100.00%**

Table S1: Publication year 1980.

Language	Number of pub.	Percentage of pub.
Afrikaans	3	0.00%
Czech	382	0.11%
Danish	1	0.00%
Dutch	153	0.05%
English	314,826	93.86%
Finnish	41	0.01%
French	4,872	1.45%
German	9,520	2.84%
Greek	1	0.00%
Hungarian	215	0.06%
Italian	462	0.14%
Japanese	1,112	0.33%
Multi-Language	1	0.00%
Norwegian	11	0.00%
Polish	378	0.11%
Portuguese	102	0.03%
Rumanian	231	0.07%
Russian	1,467	0.44%
Serbo-Croatian	35	0.01%
Slovak	7	0.00%
Spanish	1,601	0.48%
**Total**	**335,421**	**100.00%**

Table S2: Publication year 1980.

Pub. type	Number of pub.	Percentage of pub.
Art Exhibit Review	1	0.00%
Article	288,673	65.27%
Biographical-Item	10	0.00%
Book Review	3,773	0.85%
Chronology	7	0.00%
Correction	21	0.00%
Correction, Addition	3,690	0.83%
Database Review	2	0.00%
Discussion	1,159	0.26%
Editorial Material	14,156	3.20%
Hardware Review	6	0.00%
Item About an Individual	1,090	0.25%
Letter	25,335	5.73%
Meeting Abstract	68,447	15.48%
Not Specified	1	0.00%
Note	27,883	6.30%
Review	7,980	1.80%
Software Review	68	0.02%
**Total**	**442,302**	**100.00%**

Table S3: Publication year 1985.

Language	Number of pub.	Percentage of pub.
Afrikaans	18	0.00%
Chinese	400	0.09%
Czech	394	0.09%
Danish	1	0.00%
Dutch	216	0.05%
English	416,332	94.13%
Finnish	55	0.01%
French	6,733	1.52%
German	9,832	2.22%
Hungarian	175	0.04%
Italian	55	0.01%
Japanese	3,054	0.69%
Polish	248	0.06%
Portuguese	393	0.09%
Rumanian	193	0.04%
Russian	1,810	0.41%
Serbo-Croatian	53	0.01%
Slovene	11	0.00%
Spanish	2,327	0.53%
Swedish	1	0.00%
**Total**	**442,302**	**100.00%**

Table S4: Publication year 1985.

Pub. type	Number of pub.	Percentage of pub.
Article	338, 398	65.44%
Bibliography	156	0.03%
Biographical-Item	18	0.00%
Book Review	3, 741	0.72%
Chronology	2	0.00%
Correction	12	0.00%
Correction, Addition	4, 088	0.79%
Database Review	3	0.00%
Discussion	1, 266	0.24%
Editorial Material	20, 605	3.98%
Hardware Review	21	0.00%
Item About an Individual	1, 222	0.24%
Letter	29, 722	5.75%
Meeting Abstract	40, 848	7.90%
Note	32, 688	6.32%
Proceedings Paper	33, 523	6.48%
Reprint	2	0.00%
Review	10, 500	2.03%
Software Review	291	0.06%
**Total**	**517, 106**	**100.00%**

Table S5: Publication year 1990.

Language	Number of pub.	Percentage of pub.
Afrikaans	9	0.00%
Arabic	2	0.00%
Chinese	394	0.08%
Czech	421	0.08%
Dutch	166	0.03%
English	496,887	96.09%
Finnish	92	0.02%
French	5,136	0.99%
German	7,348	1.42%
Hungarian	214	0.04%
Italian	18	0.00%
Japanese	1,804	0.35%
Multi-Language	2	0.00%
Polish	210	0.04%
Portuguese	288	0.06%
Rumanian	154	0.03%
Russian	1,801	0.35%
Serbo-Croatian	52	0.01%
Slovak	20	0.00%
Slovene	18	0.00%
Spanish	2,070	0.40%
**Total**	**517,106**	**100.00%**

Table S6: Publication year 1990.

Pub. type	Number of pub.	Percentage of pub.
Article	423, 885	58.43%
Bibliography	188	0.03%
Biographical-Item	31	0.00%
Book Review	4, 410	0.61%
Chronology	2	0.00%
Correction	83	0.01%
Correction, Addition	5, 388	0.74%
Database Review	12	0.00%
Discussion	1, 484	0.20%
Editorial Material	31, 190	4.30%
Hardware Review	54	0.01%
Item About an Individual	2, 891	0.40%
Letter	35, 967	4.96%
Meeting Abstract	97, 133	13.39%
News Item	513	0.07%
Note	50, 081	6.90%
Proceedings Paper	54, 690	7.54%
Reprint	525	0.07%
Review	16, 514	2.28%
Software Review	394	0.05%
**Total**	**725, 435**	**100.00%**

Table S7: Publication year 1995.

Language	Number of pub.	Percentage of pub.
Afrikaans	7	0.00%
Chinese	1,140	0.16%
Croatian	163	0.02%
Czech	313	0.04%
Danish	19	0.00%
Dutch	268	0.04%
English	702,690	96.86%
Estonian	8	0.00%
Finnish	79	0.01%
French	6,007	0.83%
German	8,401	1.16%
Hungarian	191	0.03%
Italian	18	0.00%
Japanese	1,886	0.26%
Korean	45	0.01%
Latin	1	0.00%
Norwegian	1	0.00%
Polish	238	0.03%
Portuguese	351	0.05%
Rumanian	233	0.03%
Russian	1,185	0.16%
Slovak	27	0.00%
Slovene	51	0.01%
Spanish	2,109	0.29%
Swedish	2	0.00%
Ukrainian	1	0.00%
Welsh	1	0.00%
**Total**	**725,435**	**100.00%**

Table S8: Publication year 1995.

Pub. type	Number of pub.	Percentage of pub.
Article	545,308	63.28%
Bibliography	205	0.02%
Biographical-Item	3,460	0.40%
Book Review	3,986	0.46%
Correction	5,809	0.67%
Database Review	8	0.00%
Editorial Material	37,503	4.35%
Fiction, Creative Prose	2	0.00%
Hardware Review	24	0.00%
Letter	34,756	4.03%
Meeting Abstract	122,339	14.20%
News Item	19,111	2.22%
Note	1	0.00%
Poetry	2	0.00%
Proceedings Paper	65,322	7.58%
Reprint	573	0.07%
Review	22,946	2.66%
Software Review	340	0.04%
**Total**	**861,695**	**100.00%**

Table S9: Publication year 1999.

Language	Number of pub.	Percentage of pub.
Arabic	4	0.00%
Chinese	3,155	0.37%
Croatian	164	0.02%
Czech	398	0.05%
Dutch	195	0.02%
English	834,760	96.87%
Estonian	2	0.00%
Finnish	61	0.01%
French	6,443	0.75%
Georgian	1	0.00%
German	8,285	0.96%
Hungarian	153	0.02%
Italian	67	0.01%
Japanese	2,041	0.24%
Korean	109	0.01%
Polish	419	0.05%
Portuguese	901	0.10%
Rumanian	183	0.02%
Russian	932	0.11%
Slovak	22	0.00%
Slovene	47	0.01%
Spanish	3,176	0.37%
Swedish	1	0.00%
Turkish	169	0.02%
Welsh	7	0.00%
**Total**	**861,695**	**100.00%**

Table S10: Publication year 1999.

Pub. type	Number of pub.	Percentage of pub.
Abstract of Published Item	1	0.00%
Article	655,980	60.59%
Bibliography	102	0.01%
Biographical-Item	3,781	0.35%
Book Review	3,560	0.33%
Correction	7,764	0.72%
Database Review	5	0.00%
Editorial Material	48,964	4.52%
Hardware Review	17	0.00%
Letter	33,591	3.10%
Meeting Abstract	190,524	17.60%
News Item	18,994	1.75%
Not Specified	1	0.00%
Proceedings Paper	81,656	7.54%
Reprint	526	0.05%
Review	37,092	3.43%
Software Review	153	0.01%
**Total**	**1,082,711**	**100.00%**

Table S11: Publication year 2004.

Language	Number of pub.	Percentage of pub.
Afrikaans	1	0.00%
Chinese	5,875	0.54%
Croatian	156	0.01%
Czech	285	0.03%
Danish	13	0.00%
Dutch	172	0.02%
English	1,054,224	97.37%
Finnish	76	0.01%
French	5,769	0.53%
Georgian	2	0.00%
German	7,761	0.72%
Greek	1	0.00%
Hungarian	134	0.01%
Italian	70	0.01%
Japanese	1,603	0.15%
Korean	71	0.01%
Persian	1	0.00%
Polish	493	0.05%
Portuguese	1,220	0.11%
Rumanian	236	0.02%
Russian	1,036	0.10%
Slovak	12	0.00%
Slovene	56	0.01%
Spanish	3,363	0.31%
Turkish	57	0.01%
Ukrainian	8	0.00%
Welsh	15	0.00%
**Total**	**1,082,711**	**100.00%**

Table S12: Publication year 2004.
