# Supplementary material for: A Reverse Engineering Approach to the Suppression of Citation Biases Reveals Universal Properties of Citation Distributions
Source: PLoS One. 2012 Mar 29;7(3):e33833. doi: 10.1371/journal.pone.0033833 (PMC3315498; doi:10.1371/journal.pone.0033833)
Supplement: Supporting Information S2 — Complete analysis for publication year . (PDF) [file pone.0033833.s002.pdf]

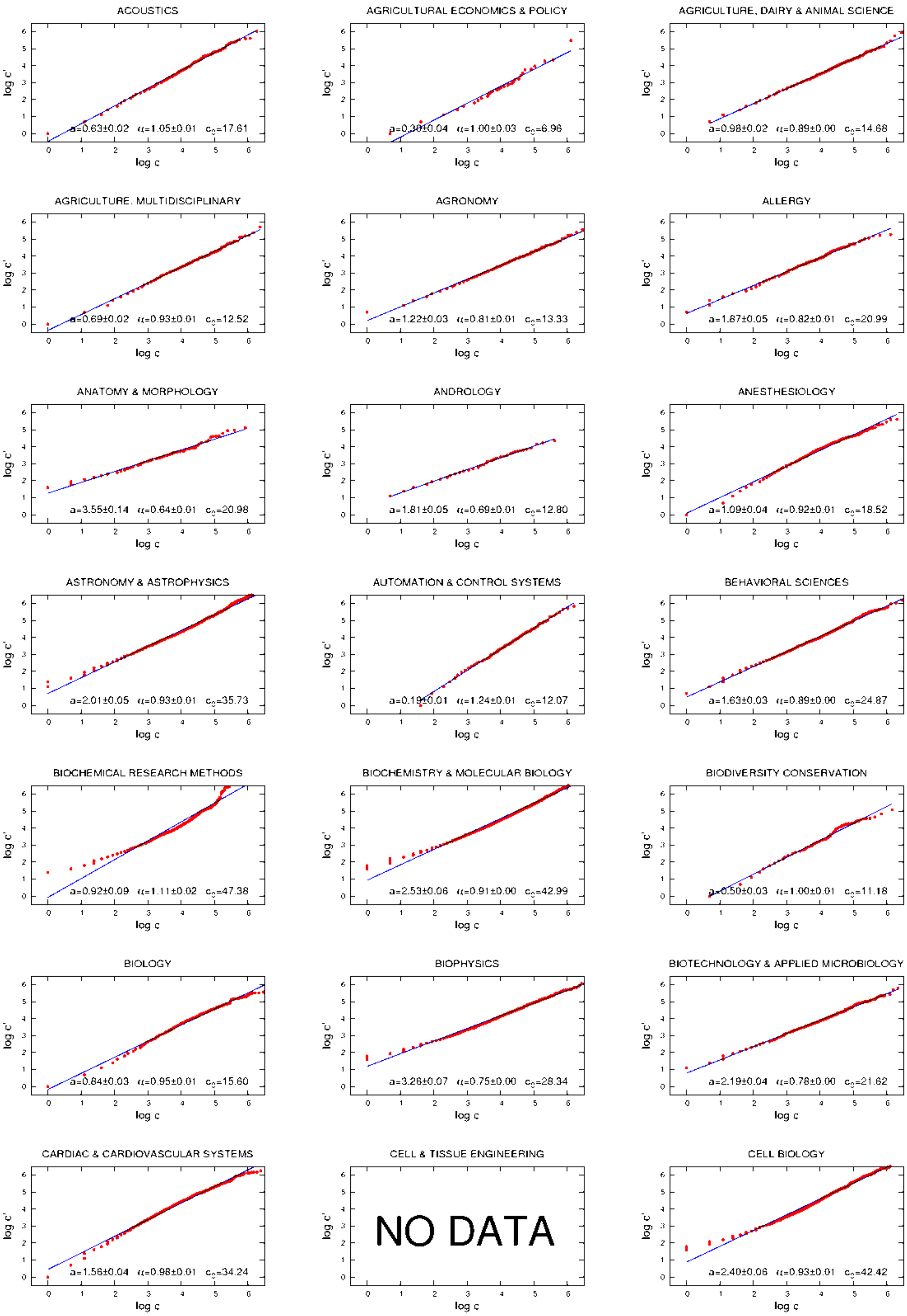

Figure S1: Publication year 1980.

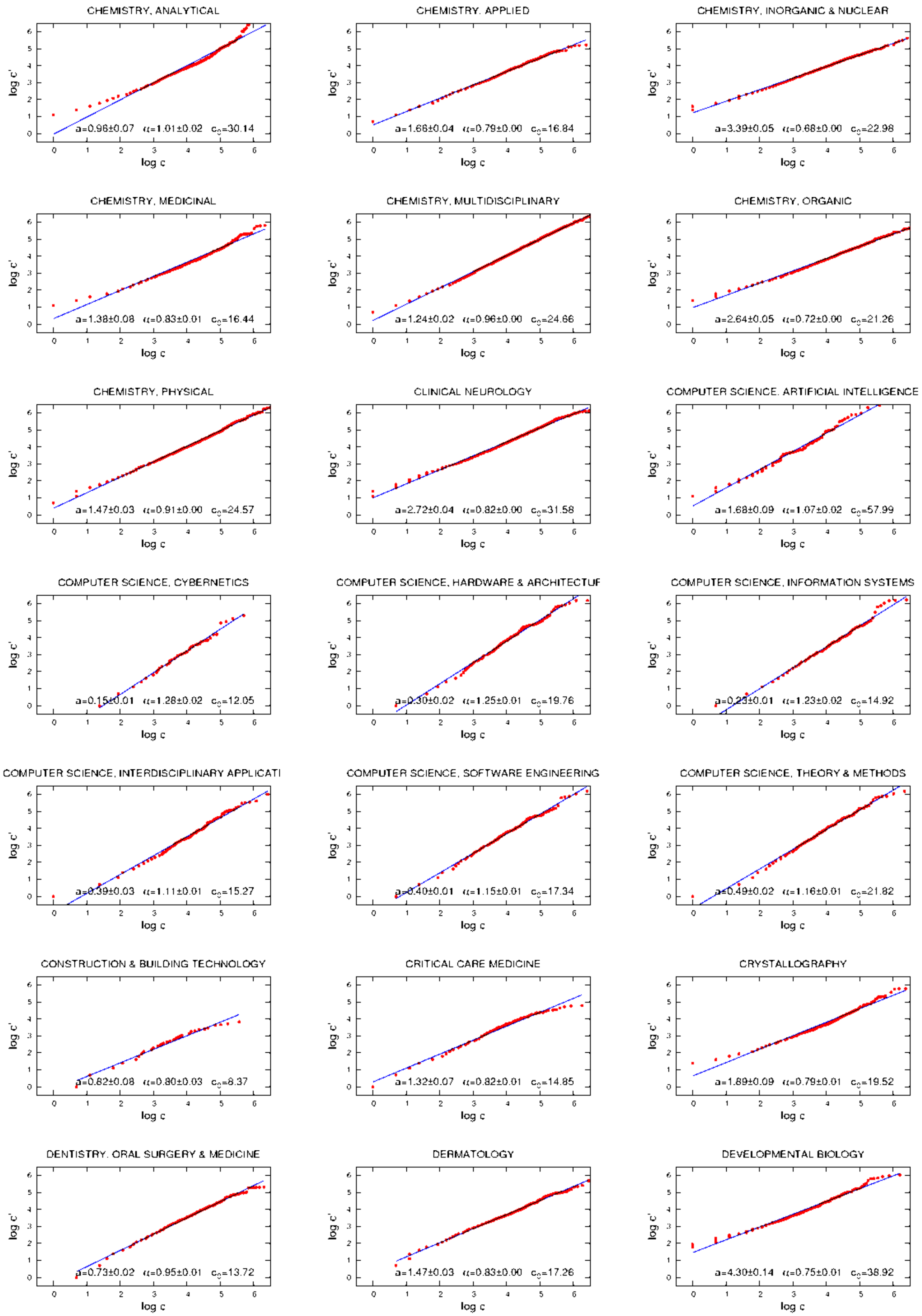

Figure S2: Publication year 1980.

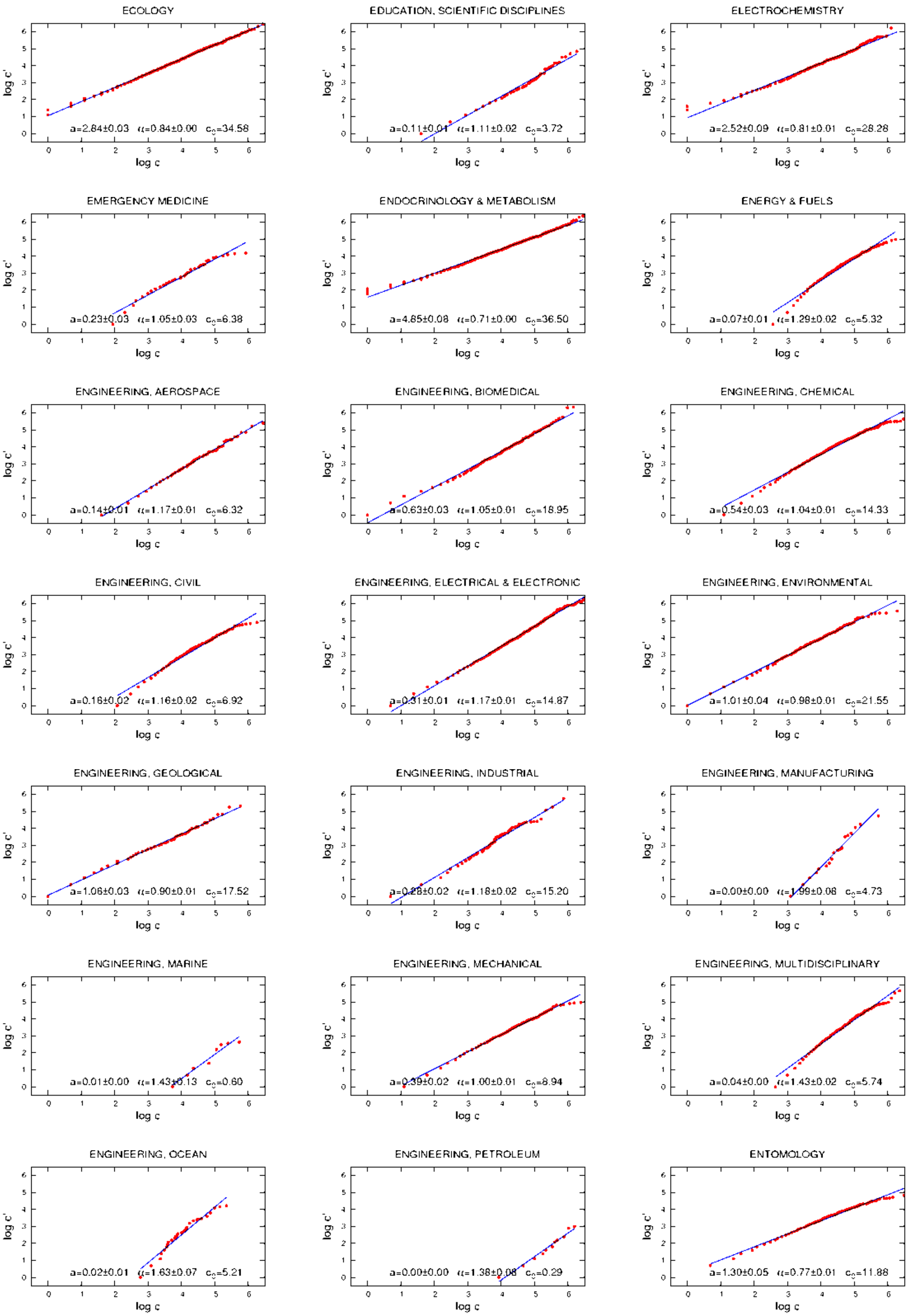

Figure S3: Publication year 1980.

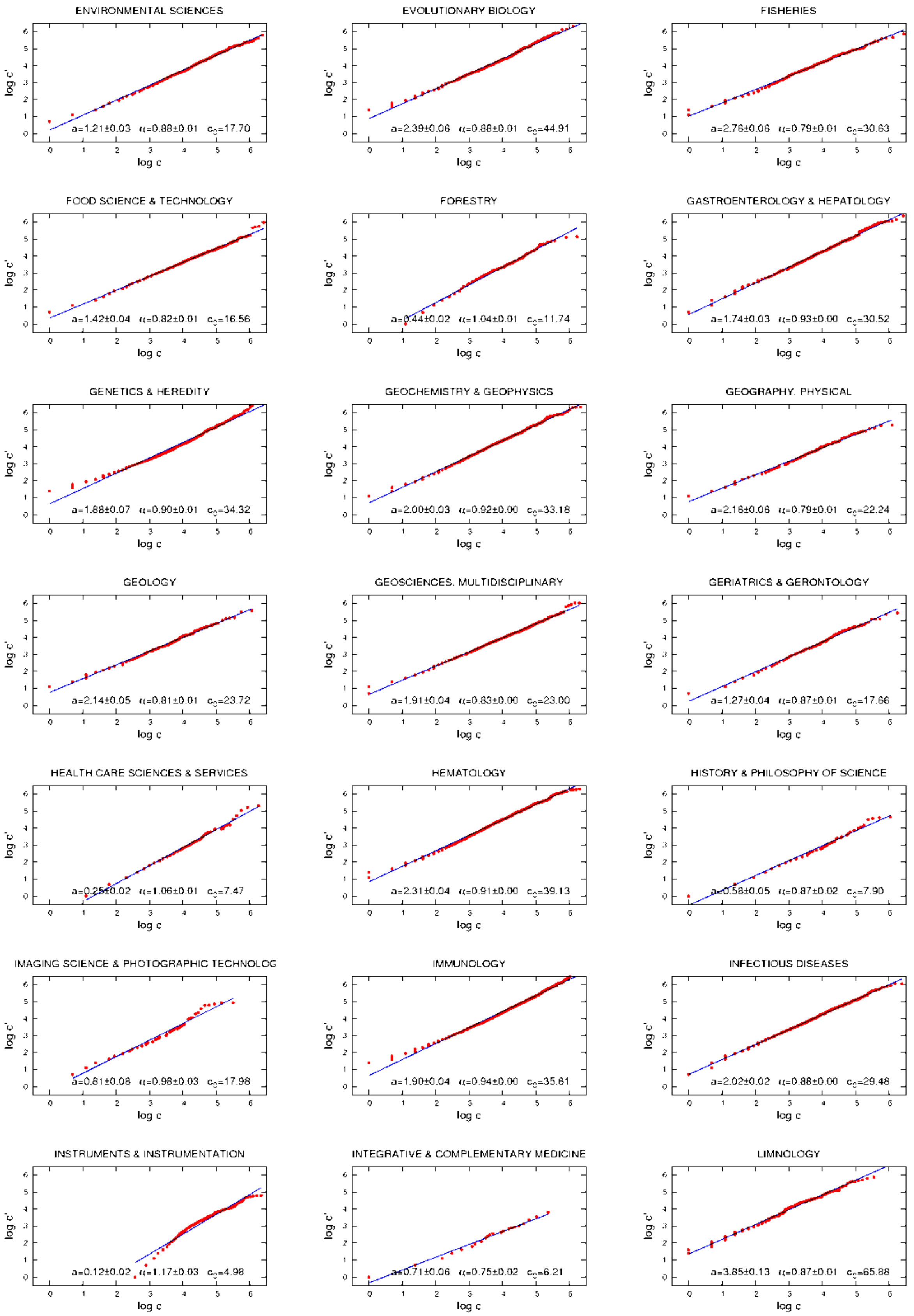

Figure S4: Publication year 1980.

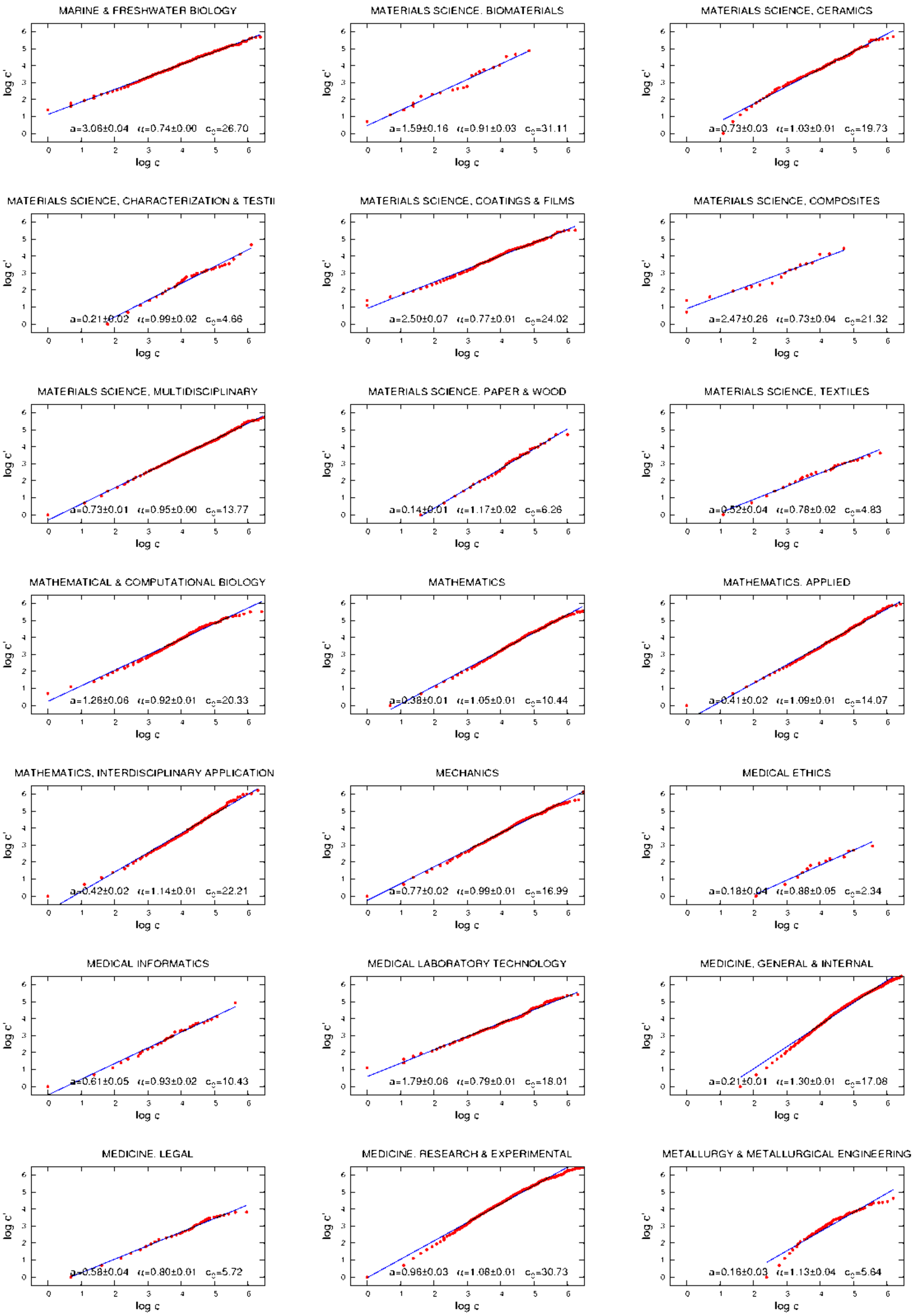

Figure S5: Publication year 1980.

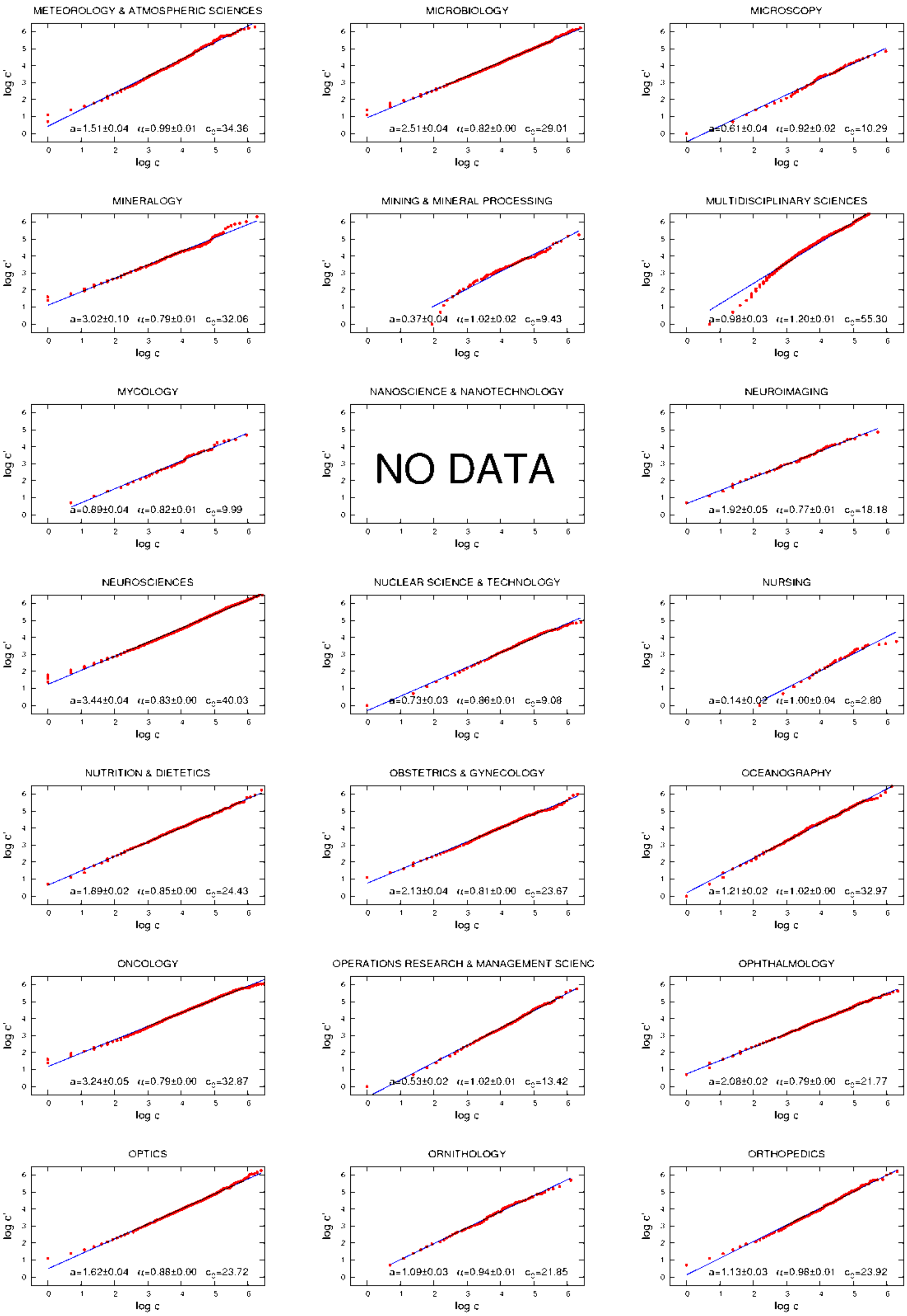

Figure S6: Publication year 1980.

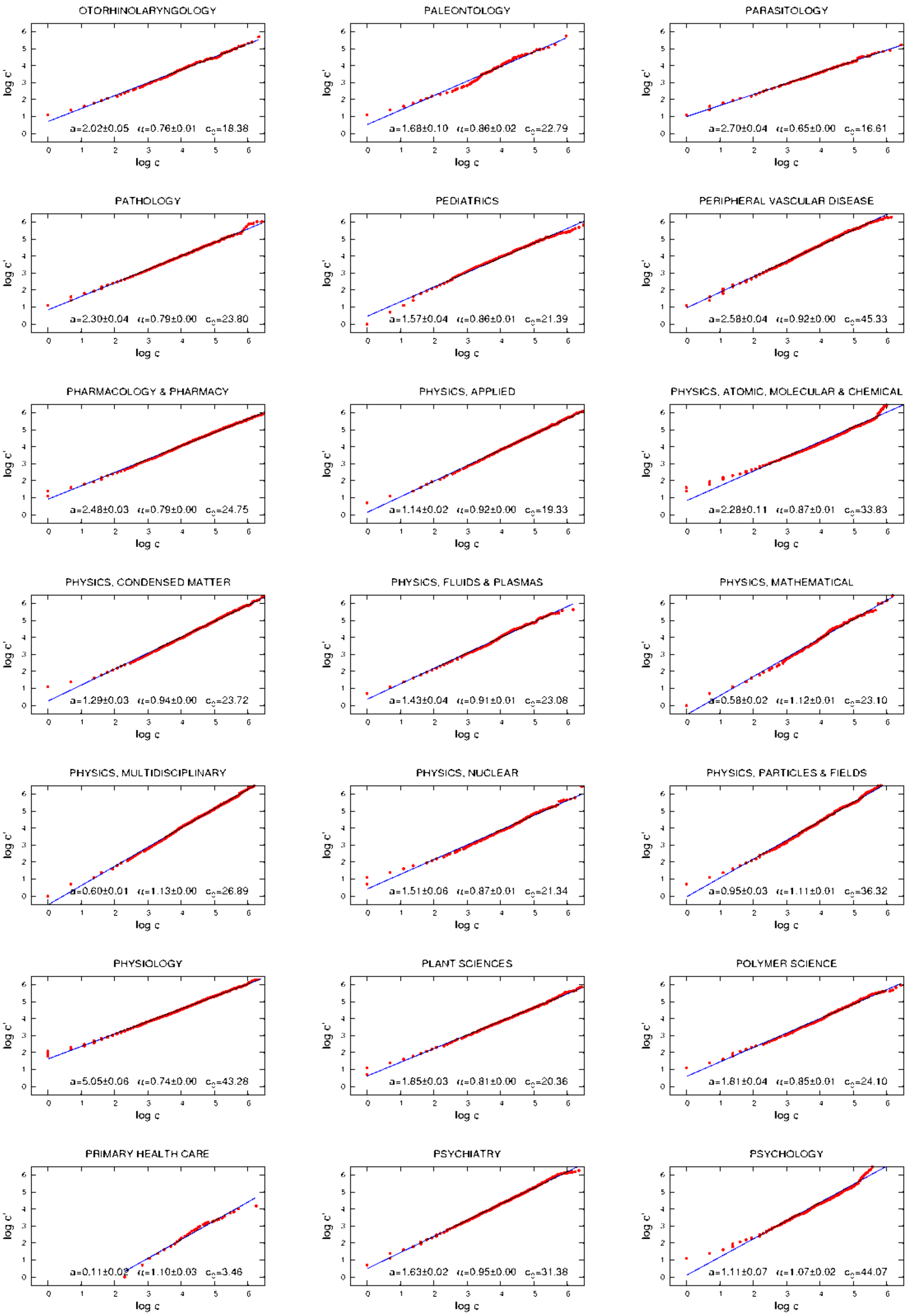

Figure S7: Publication year 1980.

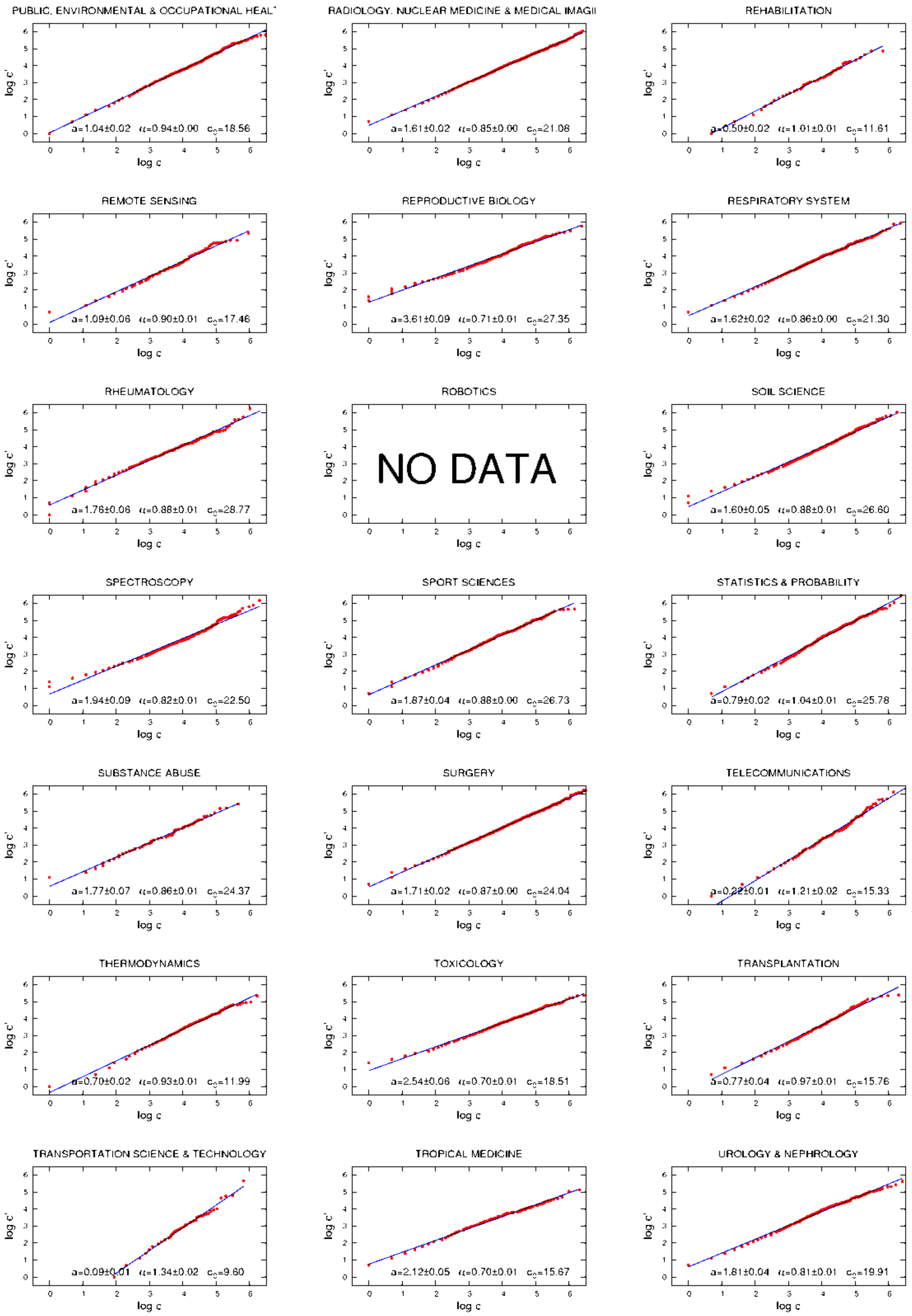

Figure S8: Publication year 1980.

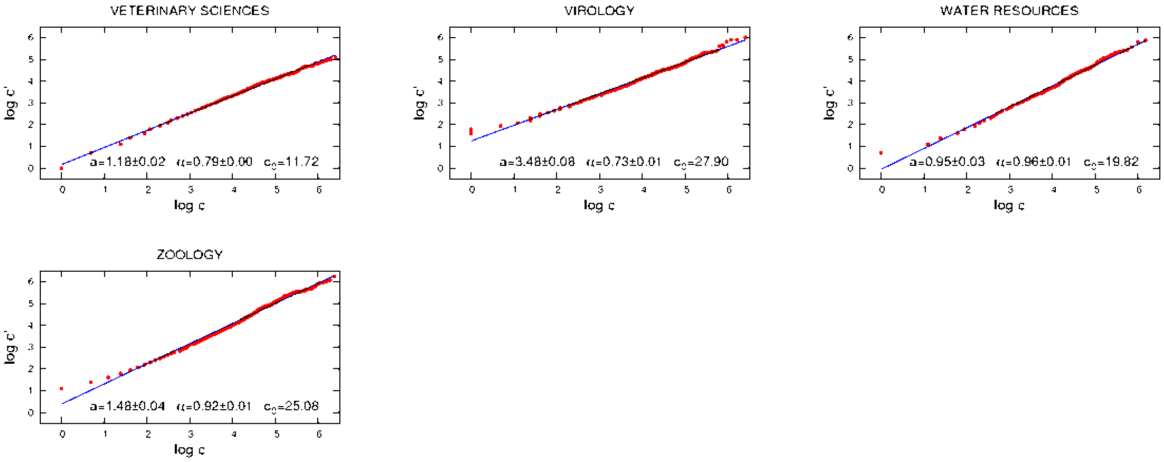

Figure S9: Publication year 1980.

Subject-category	a	α	$\langle c \rangle$	N
ACOUSTICS	0.63 ± 0.02	1.05 ± 0.01	17.61	1,232
AGRICULTURAL ECONOMICS & POLICY	0.30 ± 0.04	1.00 ± 0.03	6.96	444
AGRICULTURE, DAIRY & ANIMAL SCIENCE	0.98 ± 0.02	0.89 ± 0.00	14.68	1,763
AGRICULTURE, MULTIDISCIPLINARY	0.69 ± 0.02	0.93 ± 0.01	12.52	1,518
AGRONOMY	1.22 ± 0.03	0.81 ± 0.01	13.33	1,858
ALLERGY	1.87 ± 0.05	0.82 ± 0.01	20.99	461
ANATOMY & MORPHOLOGY	3.55 ± 0.14	0.64 ± 0.01	20.98	305
ANDROLOGY	1.81 ± 0.05	0.69 ± 0.01	12.79	161
ANESTHESIOLOGY	1.09 ± 0.04	0.92 ± 0.01	18.52	1,375
ASTRONOMY & ASTROPHYSICS	2.01 ± 0.05	0.93 ± 0.01	35.73	4,146
AUTOMATION & CONTROL SYSTEMS	0.19 ± 0.01	1.24 ± 0.01	12.07	1,082
BEHAVIORAL SCIENCES	1.63 ± 0.03	0.89 ± 0.00	24.87	1,908
BIOCHEMICAL RESEARCH METHODS	0.92 ± 0.09	1.11 ± 0.02	47.38	1,589
BIOCHEMISTRY & MOLECULAR BIOLOGY	2.53 ± 0.06	0.91 ± 0.00	42.99	14,673
BIODIVERSITY CONSERVATION	0.50 ± 0.03	1.00 ± 0.01	11.18	504
BIOLOGY	0.84 ± 0.03	0.95 ± 0.01	15.60	2,834
BIOPHYSICS	3.26 ± 0.07	0.75 ± 0.00	28.34	4,364
BIOTECHNOLOGY & APPLIED MICROBIOLOGY	2.19 ± 0.04	0.78 ± 0.00	21.62	2,159
CARDIAC & CARDIOVASCULAR SYSTEMS	1.56 ± 0.04	0.98 ± 0.01	34.24	3,144
CELL & TISSUE ENGINEERING	–	–	–	0
CELL BIOLOGY	2.40 ± 0.06	0.93 ± 0.01	42.42	5,255
CHEMISTRY, ANALYTICAL	0.96 ± 0.07	1.01 ± 0.02	30.14	3,174
CHEMISTRY, APPLIED	1.66 ± 0.04	0.79 ± 0.00	16.84	1,569
CHEMISTRY, INORGANIC & NUCLEAR	3.39 ± 0.05	0.68 ± 0.00	22.98	2,552
CHEMISTRY, MEDICINAL	1.38 ± 0.08	0.83 ± 0.01	16.44	2,185
CHEMISTRY, MULTIDISCIPLINARY	1.24 ± 0.02	0.96 ± 0.00	24.66	8,653
CHEMISTRY, ORGANIC	2.64 ± 0.05	0.72 ± 0.00	21.26	5,772
CHEMISTRY, PHYSICAL	1.47 ± 0.03	0.91 ± 0.00	24.57	6,711
CLINICAL NEUROLOGY	2.72 ± 0.04	0.82 ± 0.00	31.58	3,736
COMPUTER SCIENCE, ARTIFICIAL INTELLIGENCE	1.68 ± 0.09	1.07 ± 0.02	57.99	152
COMPUTER SCIENCE, CYBERNETICS	0.15 ± 0.01	1.28 ± 0.02	12.05	197
COMPUTER SCIENCE, HARDWARE & ARCHITECTURE	0.30 ± 0.02	1.25 ± 0.01	19.76	848
COMPUTER SCIENCE, INFORMATION SYSTEMS	0.23 ± 0.01	1.23 ± 0.02	14.92	816
COMPUTER SCIENCE, INTERDISCIPLINARY APPLICATIONS	0.39 ± 0.03	1.11 ± 0.01	15.27	845
COMPUTER SCIENCE, SOFTWARE ENGINEERING	0.40 ± 0.01	1.15 ± 0.01	17.34	831
COMPUTER SCIENCE, THEORY & METHODS	0.49 ± 0.02	1.16 ± 0.01	21.82	722
CONSTRUCTION & BUILDING TECHNOLOGY	0.82 ± 0.08	0.80 ± 0.03	8.37	146
CRITICAL CARE MEDICINE	1.32 ± 0.07	0.82 ± 0.01	14.85	609
CRYSTALLOGRAPHY	1.89 ± 0.09	0.79 ± 0.01	19.52	1,551
DENTISTRY, ORAL SURGERY & MEDICINE	0.73 ± 0.02	0.95 ± 0.01	13.72	2,077
DERMATOLOGY	1.47 ± 0.03	0.83 ± 0.00	17.26	1,866
DEVELOPMENTAL BIOLOGY	4.30 ± 0.14	0.75 ± 0.01	38.92	546
ECOLOGY	2.84 ± 0.03	0.84 ± 0.00	34.58	2,667
EDUCATION, SCIENTIFIC DISCIPLINES	0.11 ± 0.01	1.11 ± 0.02	3.72	1,234
ELECTROCHEMISTRY	2.52 ± 0.09	0.81 ± 0.01	28.28	1,302
EMERGENCY MEDICINE	0.23 ± 0.03	1.05 ± 0.03	6.38	306
ENDOCRINOLOGY & METABOLISM	4.85 ± 0.08	0.71 ± 0.00	36.50	3,617
ENERGY & FUELS	0.07 ± 0.01	1.29 ± 0.02	5.32	2,294
ENGINEERING, AEROSPACE	0.14 ± 0.01	1.17 ± 0.01	6.32	918
ENGINEERING, BIOMEDICAL	0.63 ± 0.03	1.05 ± 0.01	18.95	1,018

Table S13: Publication year 1980.

Subject-category	a	α	$\langle c \rangle$	N
ENGINEERING, CHEMICAL	0.54 ± 0.03	1.04 ± 0.01	14.33	3,802
ENGINEERING, CIVIL	0.16 ± 0.02	1.16 ± 0.02	6.92	1,232
ENGINEERING, ELECTRICAL & ELECTRONIC	0.31 ± 0.01	1.17 ± 0.01	14.87	5,755
ENGINEERING, ENVIRONMENTAL	1.01 ± 0.04	0.98 ± 0.01	21.55	647
ENGINEERING, GEOLOGICAL	1.06 ± 0.03	0.90 ± 0.01	17.52	220
ENGINEERING, INDUSTRIAL	0.28 ± 0.02	1.18 ± 0.02	15.20	274
ENGINEERING, MANUFACTURING	0.00 ± 0.00	1.99 ± 0.08	4.73	198
ENGINEERING, MARINE	0.01 ± 0.00	1.43 ± 0.13	0.60	205
ENGINEERING, MECHANICAL	0.39 ± 0.02	1.00 ± 0.01	8.94	1,583
ENGINEERING, MULTIDISCIPLINARY	0.04 ± 0.00	1.43 ± 0.02	5.74	2,211
ENGINEERING, OCEAN	0.02 ± 0.01	1.63 ± 0.07	5.21	188
ENGINEERING, PETROLEUM	0.00 ± 0.00	1.38 ± 0.08	0.29	1,092
ENTOMOLOGY	1.30 ± 0.05	0.77 ± 0.01	11.88	1,985
ENVIRONMENTAL SCIENCES	1.21 ± 0.03	0.88 ± 0.01	17.70	3,048
EVOLUTIONARY BIOLOGY	2.39 ± 0.06	0.88 ± 0.01	44.91	898
FISHERIES	2.76 ± 0.06	0.79 ± 0.01	30.63	893
FOOD SCIENCE & TECHNOLOGY	1.42 ± 0.04	0.82 ± 0.01	16.56	2,548
FORESTRY	0.44 ± 0.02	1.04 ± 0.01	11.74	576
GASTROENTEROLOGY & HEPATOLOGY	1.74 ± 0.03	0.93 ± 0.00	30.52	1,708
GENETICS & HEREDITY	1.88 ± 0.07	0.90 ± 0.01	34.32	2,515
GEOCHEMISTRY & GEOPHYSICS	2.00 ± 0.03	0.92 ± 0.00	33.18	2,177
GEOGRAPHY, PHYSICAL	2.16 ± 0.06	0.79 ± 0.01	22.24	428
GEOLOGY	2.14 ± 0.05	0.81 ± 0.01	23.72	413
GEOSCIENCES, MULTIDISCIPLINARY	1.91 ± 0.04	0.83 ± 0.00	23.00	2,026
GERIATRICS & GERONTOLOGY	1.27 ± 0.04	0.87 ± 0.01	17.66	599
HEALTH CARE SCIENCES & SERVICES	0.25 ± 0.02	1.06 ± 0.01	7.47	629
HEMATOLOGY	2.31 ± 0.04	0.91 ± 0.00	39.13	2,725
HISTORY & PHILOSOPHY OF SCIENCE	0.58 ± 0.05	0.87 ± 0.02	7.90	386
IMAGING SCIENCE & PHOTOGRAPHIC TECHNOLOGY	0.81 ± 0.08	0.98 ± 0.03	17.98	128
IMMUNOLOGY	1.90 ± 0.04	0.94 ± 0.00	35.61	6,467
INFECTIOUS DISEASES	2.02 ± 0.02	0.88 ± 0.00	29.48	1,561
INSTRUMENTS & INSTRUMENTATION	0.12 ± 0.02	1.17 ± 0.03	4.98	2,197
INTEGRATIVE & COMPLEMENTARY MEDICINE	0.71 ± 0.06	0.75 ± 0.02	6.21	97
LIMNOLOGY	3.85 ± 0.13	0.87 ± 0.01	65.88	277
MARINE & FRESHWATER BIOLOGY	3.06 ± 0.04	0.74 ± 0.00	26.70	2,297
MATERIALS SCIENCE, BIOMATERIALS	1.59 ± 0.16	0.91 ± 0.03	31.11	36
MATERIALS SCIENCE, CERAMICS	0.73 ± 0.03	1.03 ± 0.01	19.73	1,076
MATERIALS SCIENCE, CHARACTERIZATION & TESTING	0.21 ± 0.02	0.99 ± 0.02	4.66	442
MATERIALS SCIENCE, COATINGS & FILMS	2.50 ± 0.07	0.77 ± 0.01	24.02	1,152
MATERIALS SCIENCE, COMPOSITES	2.47 ± 0.26	0.73 ± 0.04	21.32	28
MATERIALS SCIENCE, MULTIDISCIPLINARY	0.73 ± 0.01	0.95 ± 0.00	13.77	5,489
MATERIALS SCIENCE, PAPER & WOOD	0.14 ± 0.01	1.17 ± 0.02	6.26	349
MATERIALS SCIENCE, TEXTILES	0.52 ± 0.04	0.78 ± 0.02	4.83	235
MATHEMATICAL & COMPUTATIONAL BIOLOGY	1.26 ± 0.06	0.92 ± 0.01	20.33	834
MATHEMATICS	0.38 ± 0.01	1.05 ± 0.01	10.44	5,421
MATHEMATICS, APPLIED	0.41 ± 0.02	1.09 ± 0.01	14.07	2,480
MATHEMATICS, INTERDISCIPLINARY APPLICATIONS	0.42 ± 0.02	1.14 ± 0.01	22.21	1,323
MECHANICS	0.77 ± 0.02	0.99 ± 0.01	16.99	2,893
MEDICAL ETHICS	0.18 ± 0.04	0.88 ± 0.05	2.34	143
MEDICAL INFORMATICS	0.61 ± 0.05	0.93 ± 0.02	10.43	162

Table S14: Publication year 1980.

Subject-category	a	α	$\langle c \rangle$	N
MEDICAL LABORATORY TECHNOLOGY	1.79 ± 0.06	0.79 ± 0.01	18.01	1,379
MEDICINE, GENERAL & INTERNAL	0.21 ± 0.01	1.30 ± 0.01	17.08	14,744
MEDICINE, LEGAL	0.58 ± 0.04	0.80 ± 0.01	5.72	326
MEDICINE, RESEARCH & EXPERIMENTAL	0.96 ± 0.03	1.08 ± 0.01	30.73	4,480
METALLURGY & METALLURGICAL ENGINEERING	0.16 ± 0.03	1.13 ± 0.04	5.64	1,076
METEOROLOGY & ATMOSPHERIC SCIENCES	1.51 ± 0.04	0.99 ± 0.01	34.36	1,123
MICROBIOLOGY	2.51 ± 0.04	0.82 ± 0.00	29.01	4,039
MICROSCOPY	0.61 ± 0.04	0.92 ± 0.02	10.29	334
MINERALOGY	3.02 ± 0.10	0.79 ± 0.01	32.06	634
MINING & MINERAL PROCESSING	0.37 ± 0.04	1.02 ± 0.02	9.43	736
MULTIDISCIPLINARY SCIENCES	0.98 ± 0.03	1.20 ± 0.01	55.30	4,879
MYCOLOGY	0.89 ± 0.04	0.82 ± 0.01	9.99	329
NANOSCIENCE & NANOTECHNOLOGY	–	–	–	0
NEUROIMAGING	1.92 ± 0.05	0.77 ± 0.01	18.18	203
NEUROSCIENCES	3.44 ± 0.04	0.83 ± 0.00	40.03	7,751
NUCLEAR SCIENCE & TECHNOLOGY	0.73 ± 0.03	0.86 ± 0.01	9.08	2,477
NURSING	0.14 ± 0.02	1.00 ± 0.04	2.80	660
NUTRITION & DIETETICS	1.89 ± 0.02	0.85 ± 0.00	24.43	1,677
OBSTETRICS & GYNECOLOGY	2.13 ± 0.04	0.81 ± 0.00	23.67	2,060
OCEANOGRAPHY	1.21 ± 0.02	1.02 ± 0.00	32.97	979
ONCOLOGY	3.24 ± 0.05	0.79 ± 0.00	32.87	4,938
OPERATIONS RESEARCH & MANAGEMENT SCIENCE	0.53 ± 0.02	1.02 ± 0.01	13.42	1,297
OPHTHALMOLOGY	2.08 ± 0.02	0.79 ± 0.00	21.77	2,123
OPTICS	1.62 ± 0.04	0.88 ± 0.00	23.72	3,226
ORNITHOLOGY	1.09 ± 0.03	0.94 ± 0.01	21.85	443
ORTHOPEDICS	1.13 ± 0.03	0.98 ± 0.01	23.92	1,383
OTORHINOLARYNGOLOGY	2.02 ± 0.05	0.76 ± 0.01	18.38	1,413
PALEONTOLOGY	1.68 ± 0.10	0.86 ± 0.02	22.79	330
PARASITOLOGY	2.70 ± 0.04	0.65 ± 0.00	16.61	858
PATHOLOGY	2.30 ± 0.04	0.79 ± 0.00	23.80	2,557
PEDIATRICS	1.57 ± 0.04	0.86 ± 0.01	21.39	2,936
PERIPHERAL VASCULAR DISEASE	2.58 ± 0.04	0.92 ± 0.00	45.33	1,929
PHARMACOLOGY & PHARMACY	2.48 ± 0.03	0.79 ± 0.00	24.75	10,063
PHYSICS, APPLIED	1.14 ± 0.02	0.92 ± 0.00	19.33	6,240
PHYSICS, ATOMIC, MOLECULAR & CHEMICAL	2.28 ± 0.11	0.87 ± 0.01	33.83	5,959
PHYSICS, CONDENSED MATTER	1.29 ± 0.03	0.94 ± 0.00	23.72	5,820
PHYSICS, FLUIDS & PLASMAS	1.43 ± 0.04	0.91 ± 0.01	23.08	1,042
PHYSICS, MATHEMATICAL	0.58 ± 0.02	1.12 ± 0.01	23.10	1,048
PHYSICS, MULTIDISCIPLINARY	0.60 ± 0.01	1.13 ± 0.00	26.89	6,870
PHYSICS, NUCLEAR	1.51 ± 0.06	0.87 ± 0.01	21.34	1,719
PHYSICS, PARTICLES & FIELDS	0.95 ± 0.03	1.11 ± 0.01	36.32	1,480
PHYSIOLOGY	5.05 ± 0.06	0.74 ± 0.00	43.28	3,330
PLANT SCIENCES	1.85 ± 0.03	0.81 ± 0.00	20.36	6,050
POLYMER SCIENCE	1.81 ± 0.04	0.85 ± 0.01	24.10	2,573
PRIMARY HEALTH CARE	0.11 ± 0.02	1.10 ± 0.03	3.46	595
PSYCHIATRY	1.63 ± 0.02	0.95 ± 0.00	31.38	2,919
PSYCHOLOGY	1.11 ± 0.07	1.07 ± 0.02	44.07	1,186
PUBLIC, ENVIRONMENTAL & OCCUPATIONAL HEALTH	1.04 ± 0.02	0.94 ± 0.00	18.56	2,876
RADIOLOGY, NUCLEAR MEDICINE & MEDICAL IMAGING	1.61 ± 0.02	0.85 ± 0.00	21.08	4,132
REHABILITATION	0.50 ± 0.02	1.01 ± 0.01	11.61	247

Table S15: Publication year 1980.

Subject-category	a	α	$\langle c \rangle$	N
REMOTE SENSING	1.09 ± 0.06	0.90 ± 0.01	17.46	329
REPRODUCTIVE BIOLOGY	3.61 ± 0.09	0.71 ± 0.01	27.35	780
RESPIRATORY SYSTEM	1.62 ± 0.02	0.86 ± 0.00	21.30	1,497
RHEUMATOLOGY	1.76 ± 0.06	0.88 ± 0.01	28.77	720
ROBOTICS	–	–	–	0
SOIL SCIENCE	1.60 ± 0.05	0.88 ± 0.01	26.60	1,207
SPECTROSCOPY	1.94 ± 0.09	0.82 ± 0.01	22.50	1,367
SPORT SCIENCES	1.87 ± 0.04	0.88 ± 0.00	26.73	1,008
STATISTICS & PROBABILITY	0.79 ± 0.02	1.04 ± 0.01	25.78	1,524
SUBSTANCE ABUSE	1.77 ± 0.07	0.86 ± 0.01	24.37	175
SURGERY	1.71 ± 0.02	0.87 ± 0.00	24.04	6,954
TELECOMMUNICATIONS	0.22 ± 0.01	1.21 ± 0.02	15.33	967
THERMODYNAMICS	0.70 ± 0.02	0.93 ± 0.01	11.99	1,160
TOXICOLOGY	2.54 ± 0.06	0.70 ± 0.01	18.51	1,804
TRANSPLANTATION	0.77 ± 0.04	0.97 ± 0.01	15.76	680
TRANSPORTATION SCIENCE & TECHNOLOGY	0.09 ± 0.01	1.34 ± 0.02	9.60	250
TROPICAL MEDICINE	2.12 ± 0.05	0.70 ± 0.01	15.67	698
UROLOGY & NEPHROLOGY	1.81 ± 0.04	0.81 ± 0.01	19.91	1,656
VETERINARY SCIENCES	1.18 ± 0.02	0.79 ± 0.00	11.72	3,318
VIROLOGY	3.48 ± 0.08	0.73 ± 0.01	27.90	1,648
WATER RESOURCES	0.95 ± 0.03	0.96 ± 0.01	19.82	1,046
ZOOLOGY	1.48 ± 0.04	0.92 ± 0.01	25.08	3,147
**TOTAL**	1.00 ± 0.00	1.00 ± 0.00	24.55	355,140

Table S16: Publication year 1980.

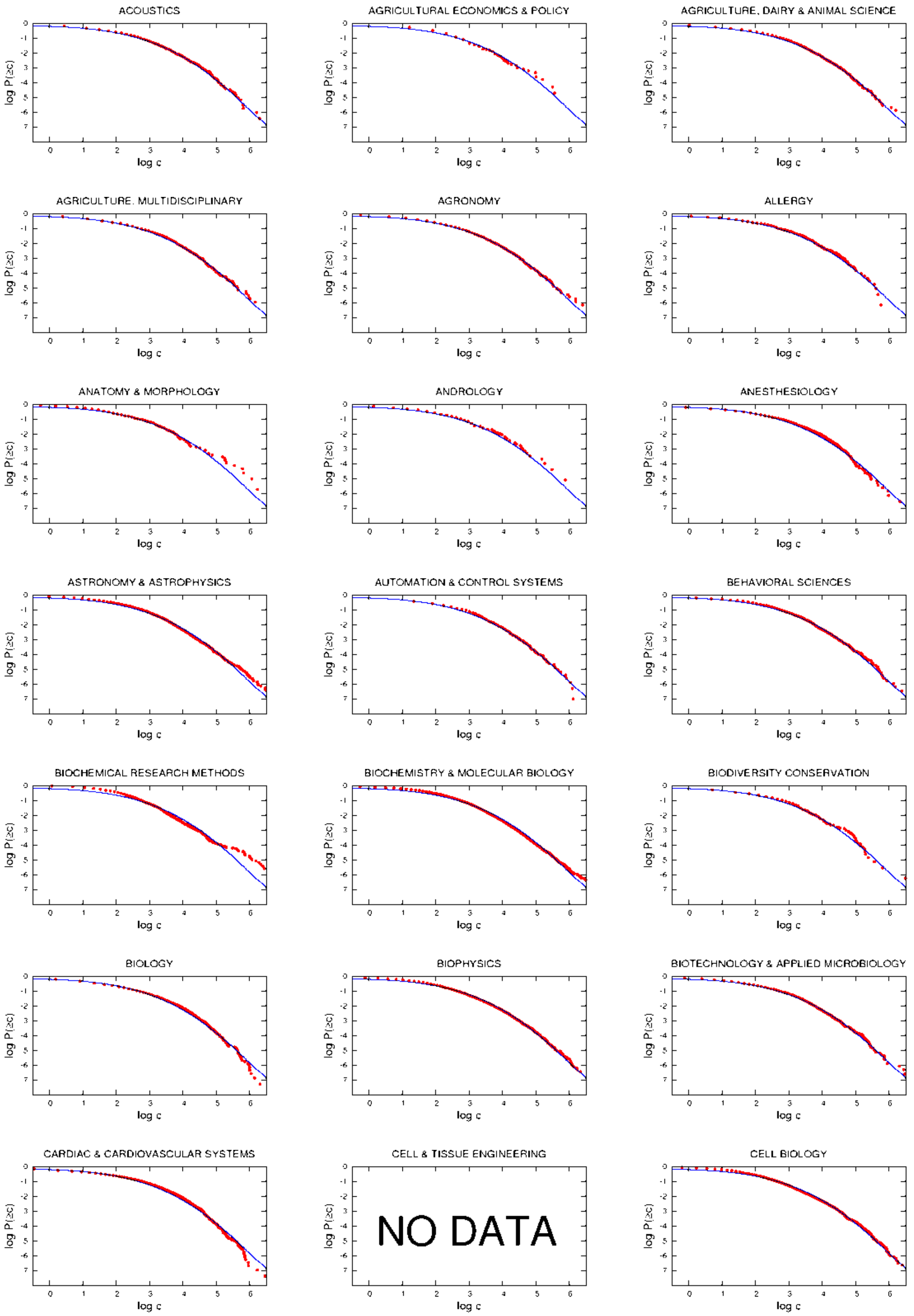

Figure S10: Publication year 1980.

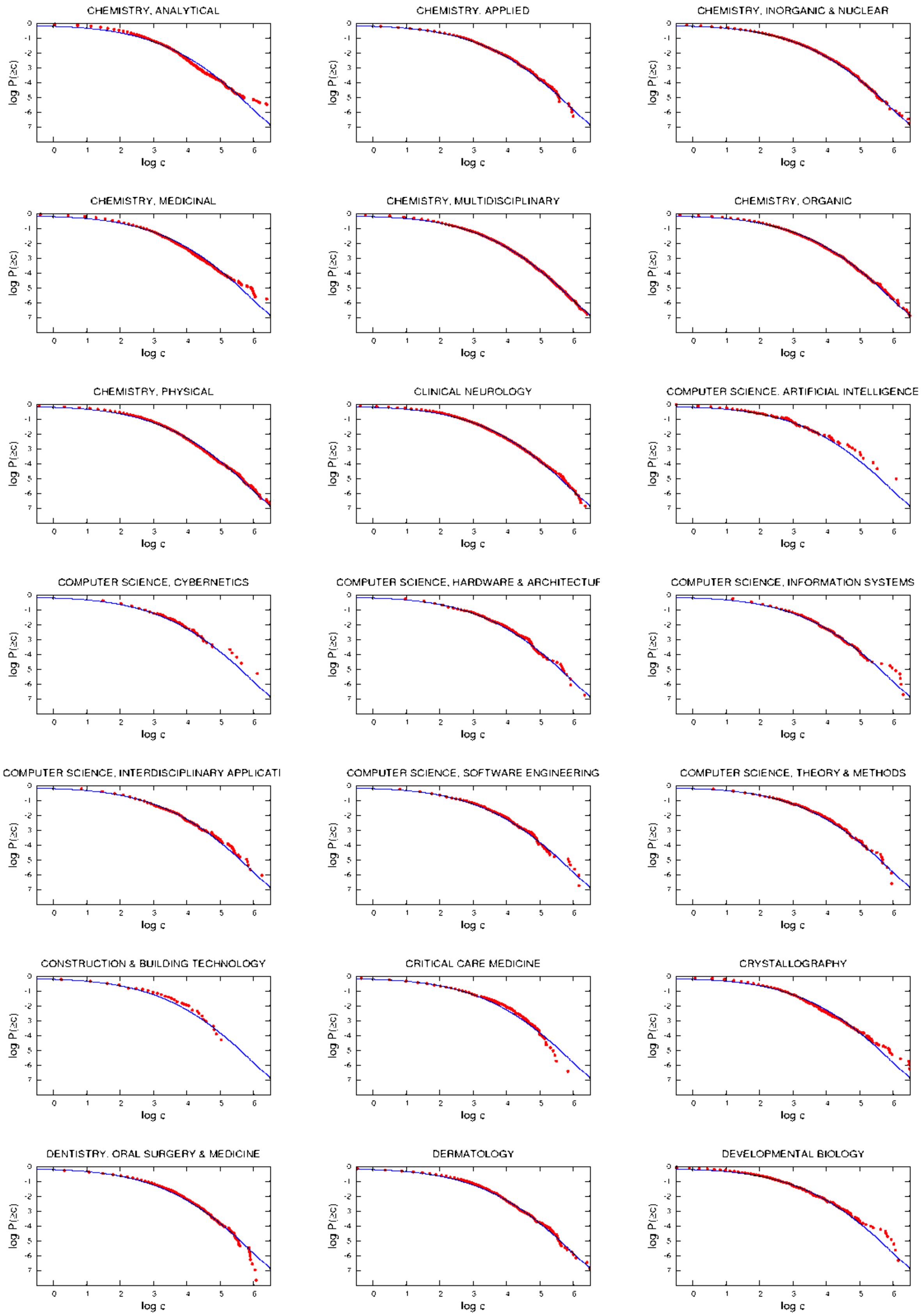

Figure S11: Publication year 1980.

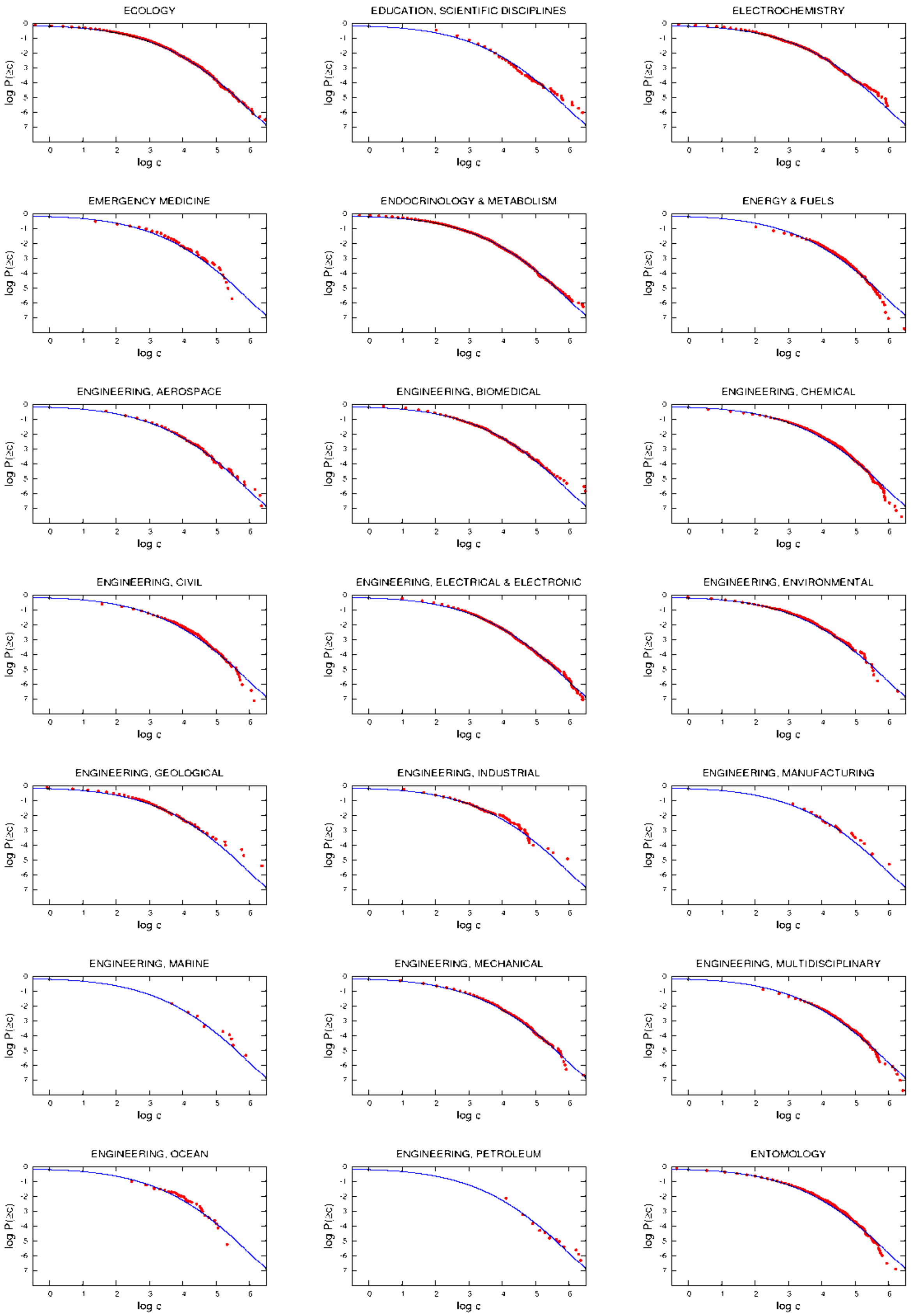

Figure S12: Publication year 1980.

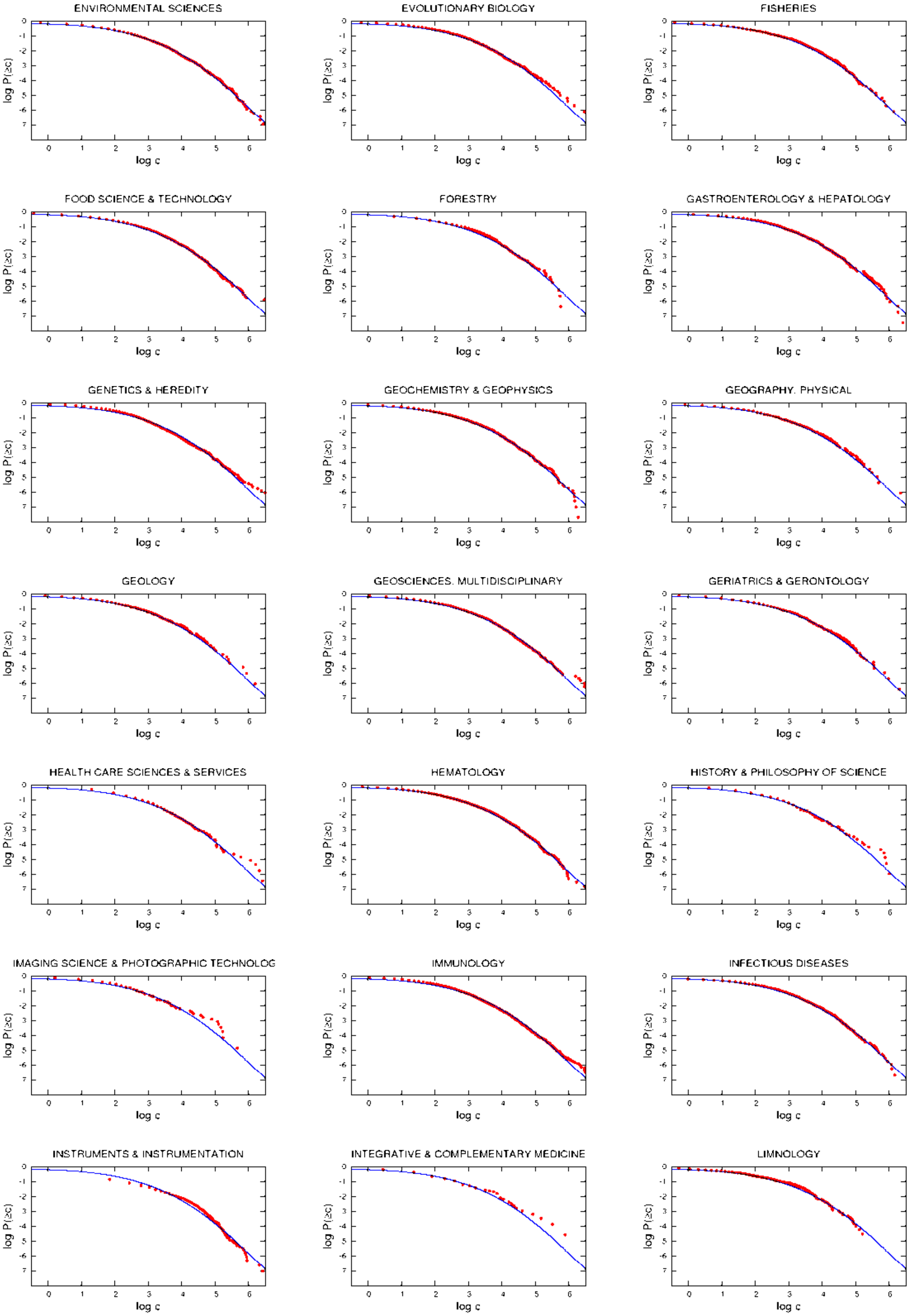

Figure S13: Publication year 1980.

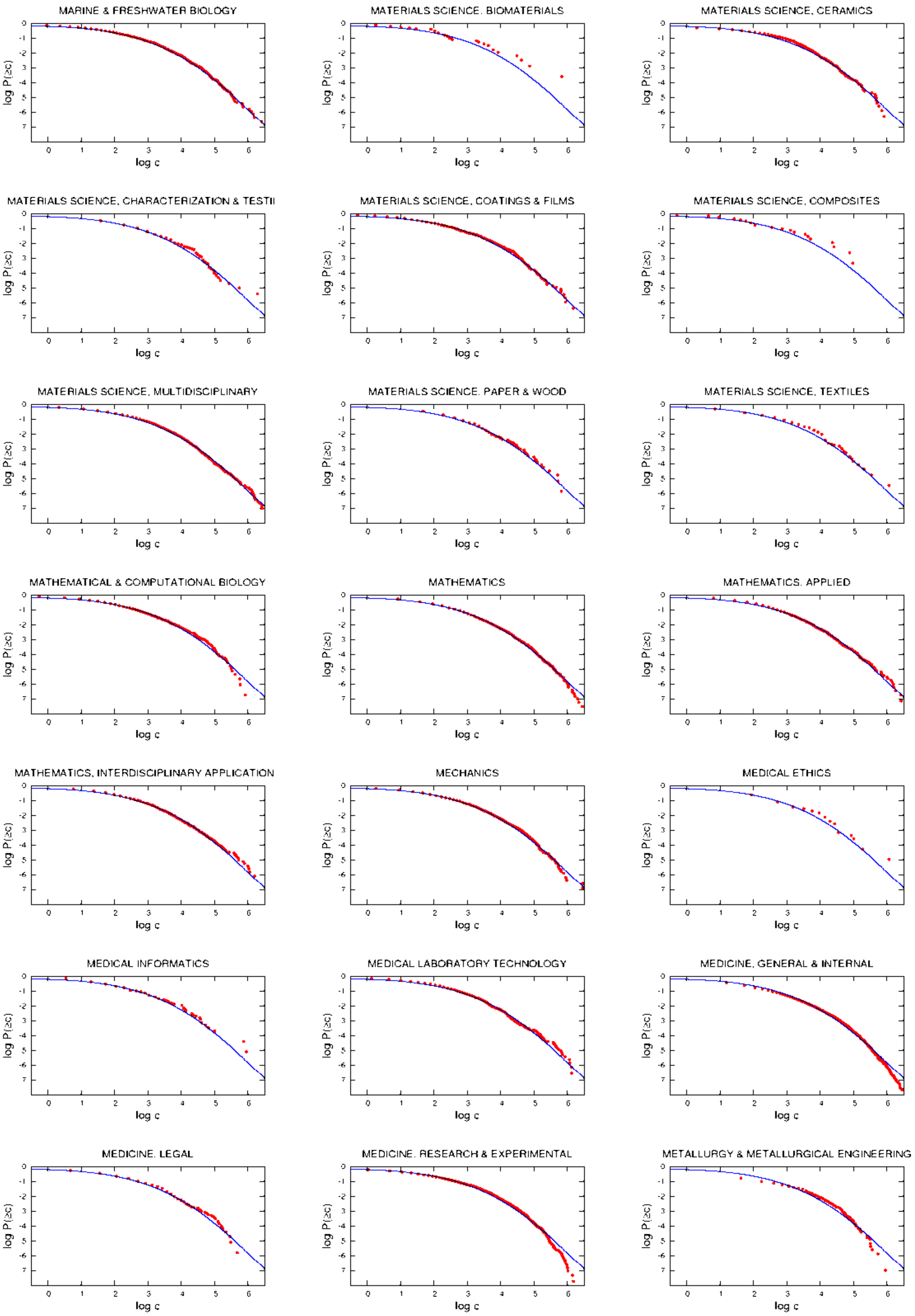

Figure S14: Publication year 1980.

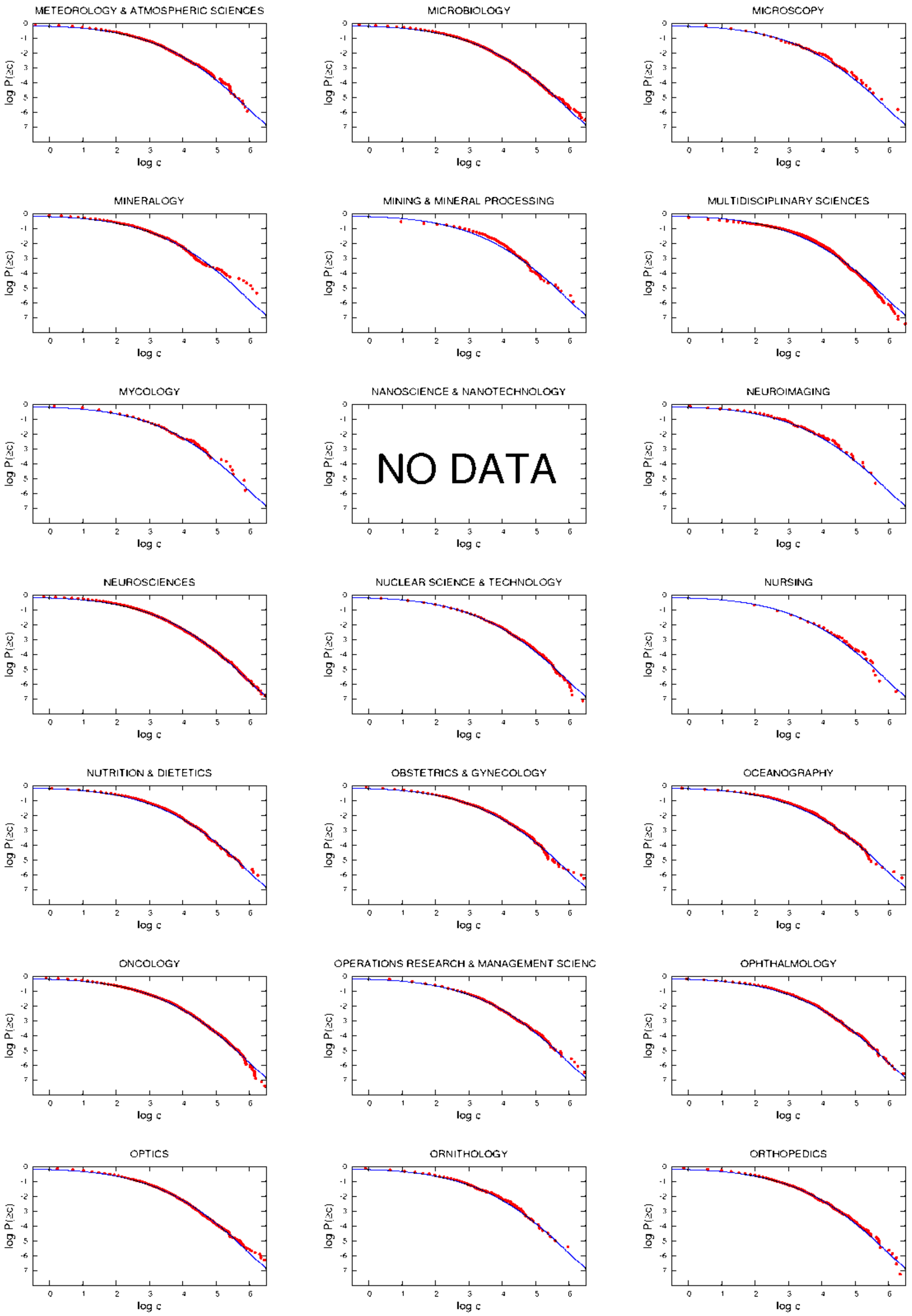

Figure S15: Publication year 1980.

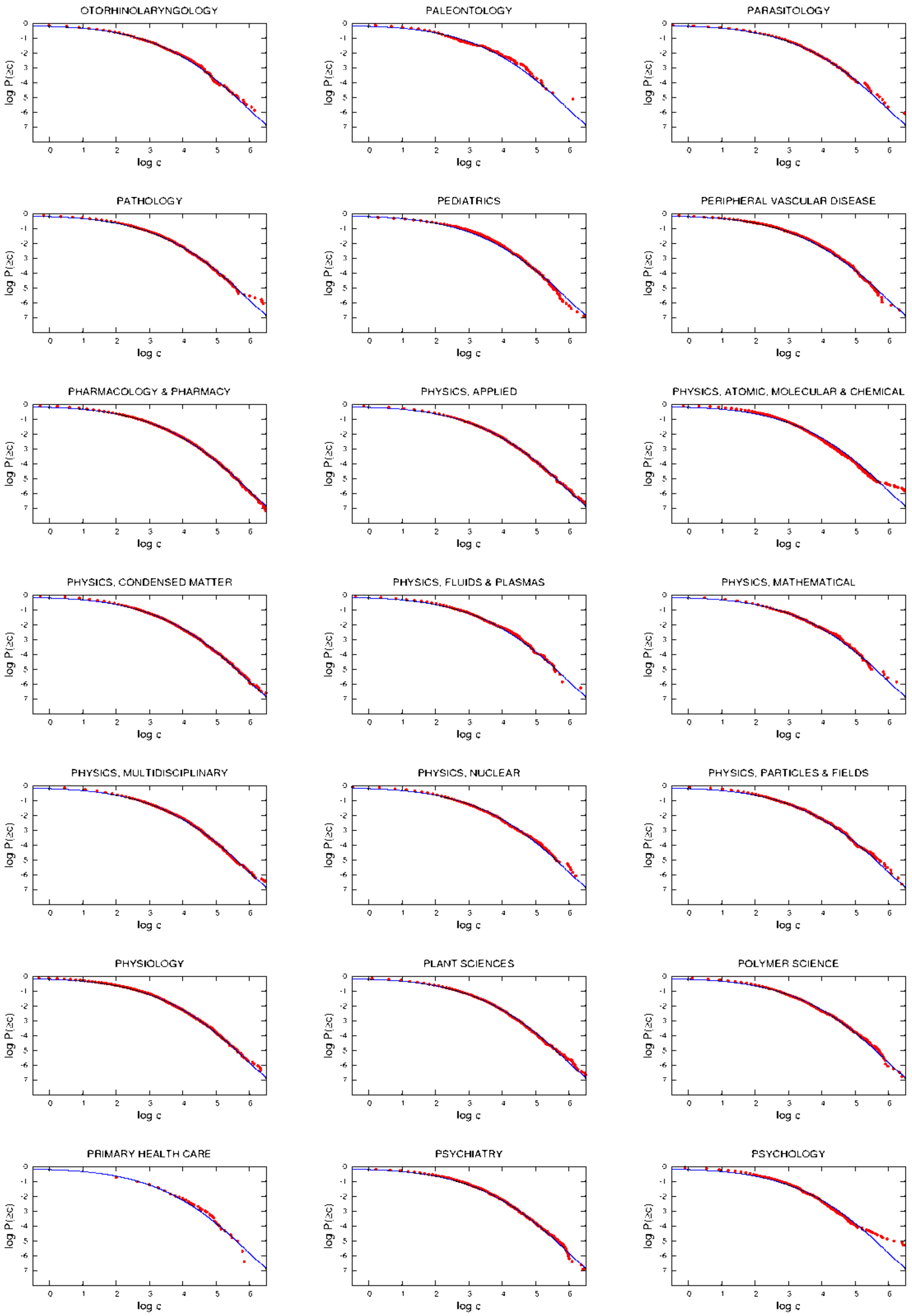

Figure S16: Publication year 1980.

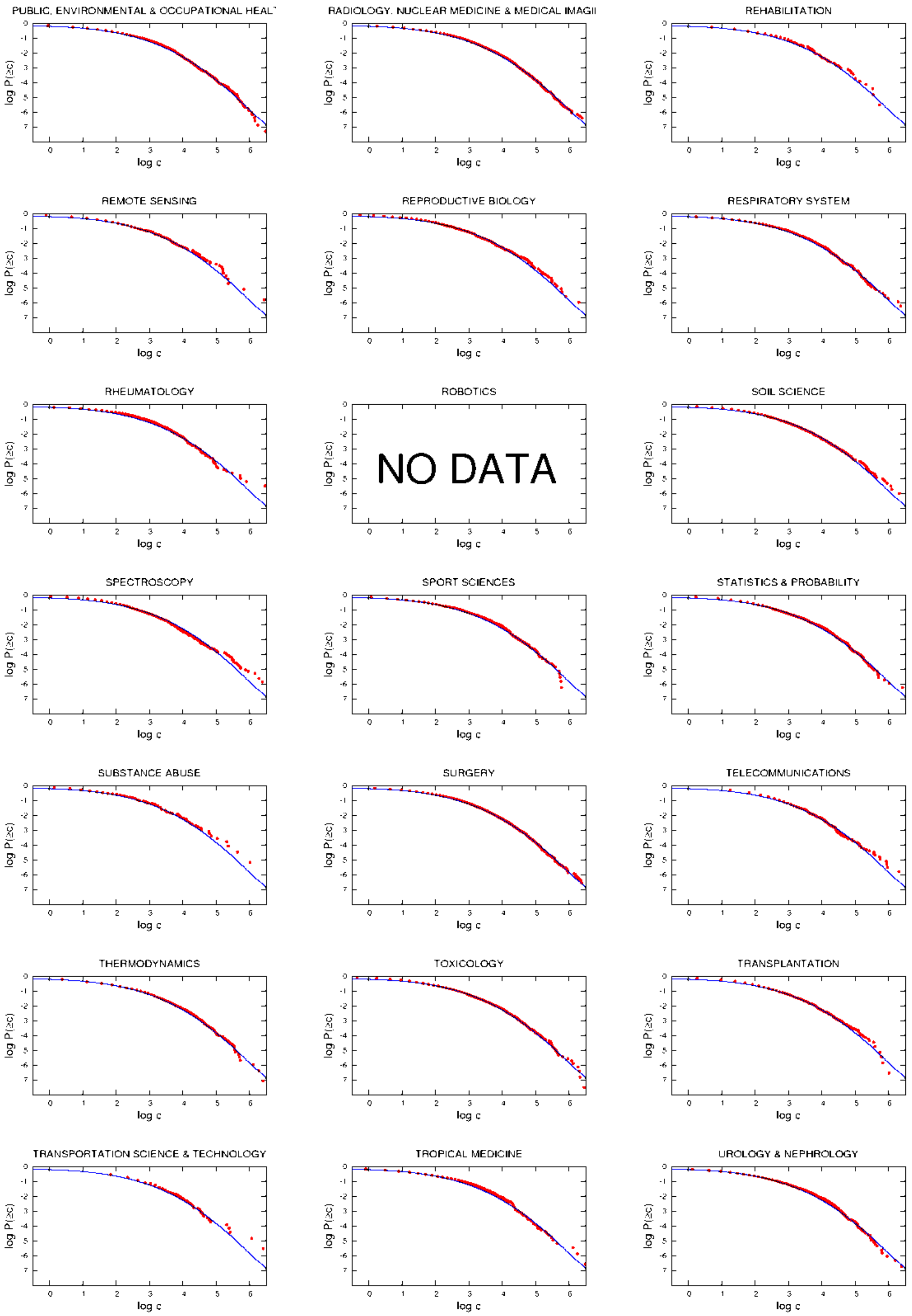

Figure S17: Publication year 1980.

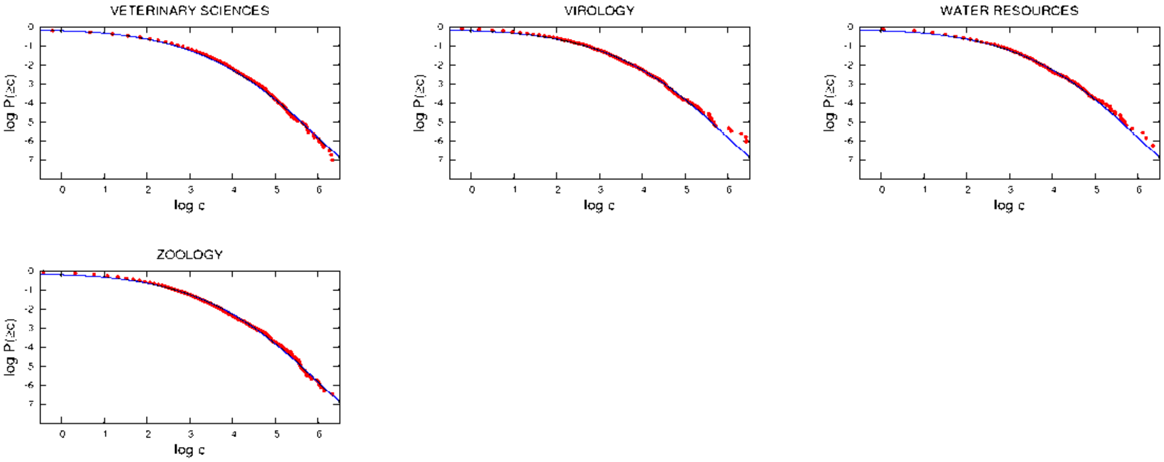

Figure S18: Publication year 1980.

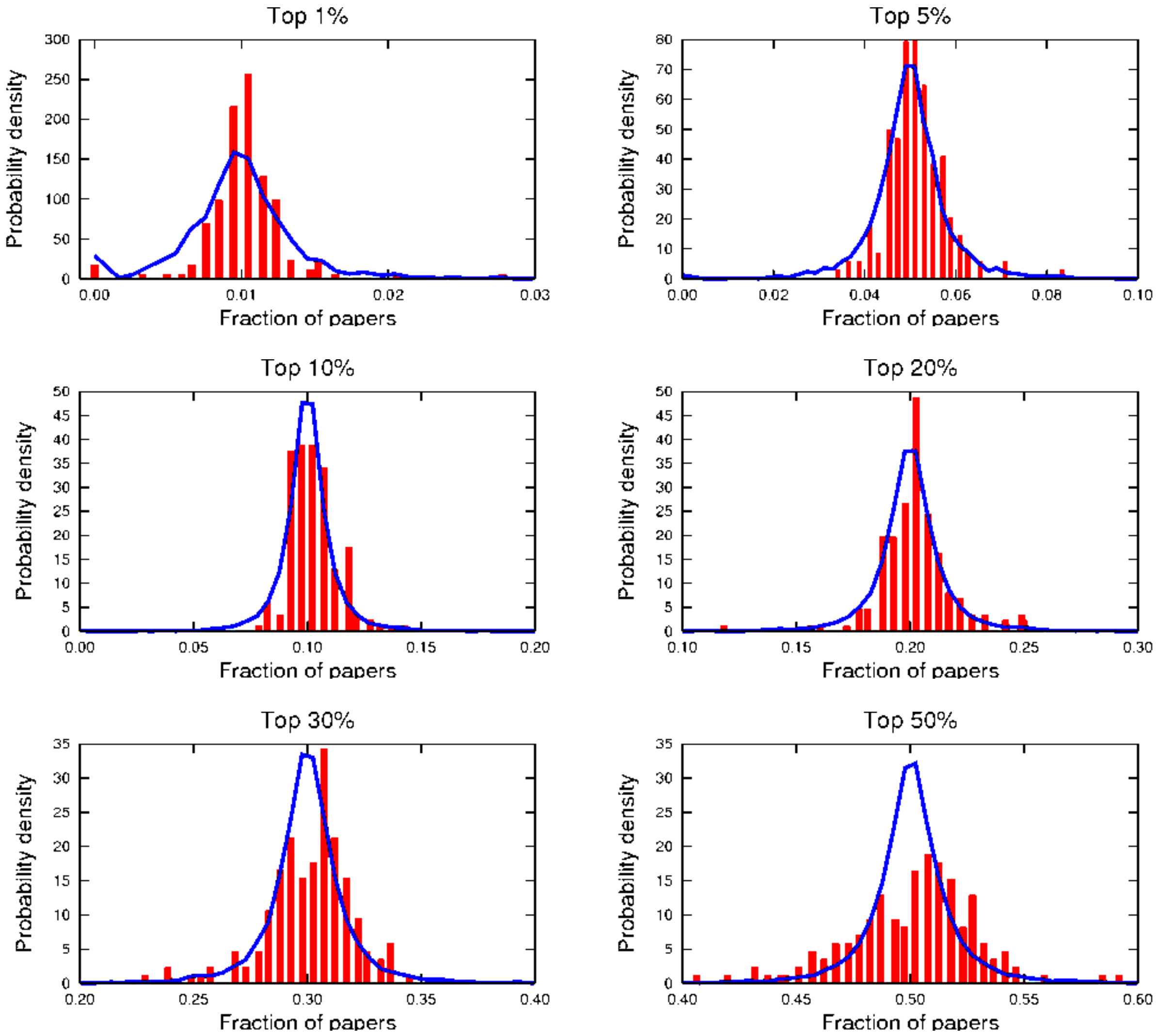

Figure S19: Publication year 1980.
