# Supplementary material for: A Reverse Engineering Approach to the Suppression of Citation Biases Reveals Universal Properties of Citation Distributions
Source: PLoS One. 2012 Mar 29;7(3):e33833. doi: 10.1371/journal.pone.0033833 (PMC3315498; doi:10.1371/journal.pone.0033833)
Supplement: Supporting Information S3 — Complete analysis for publication year . (PDF) [file pone.0033833.s003.pdf]

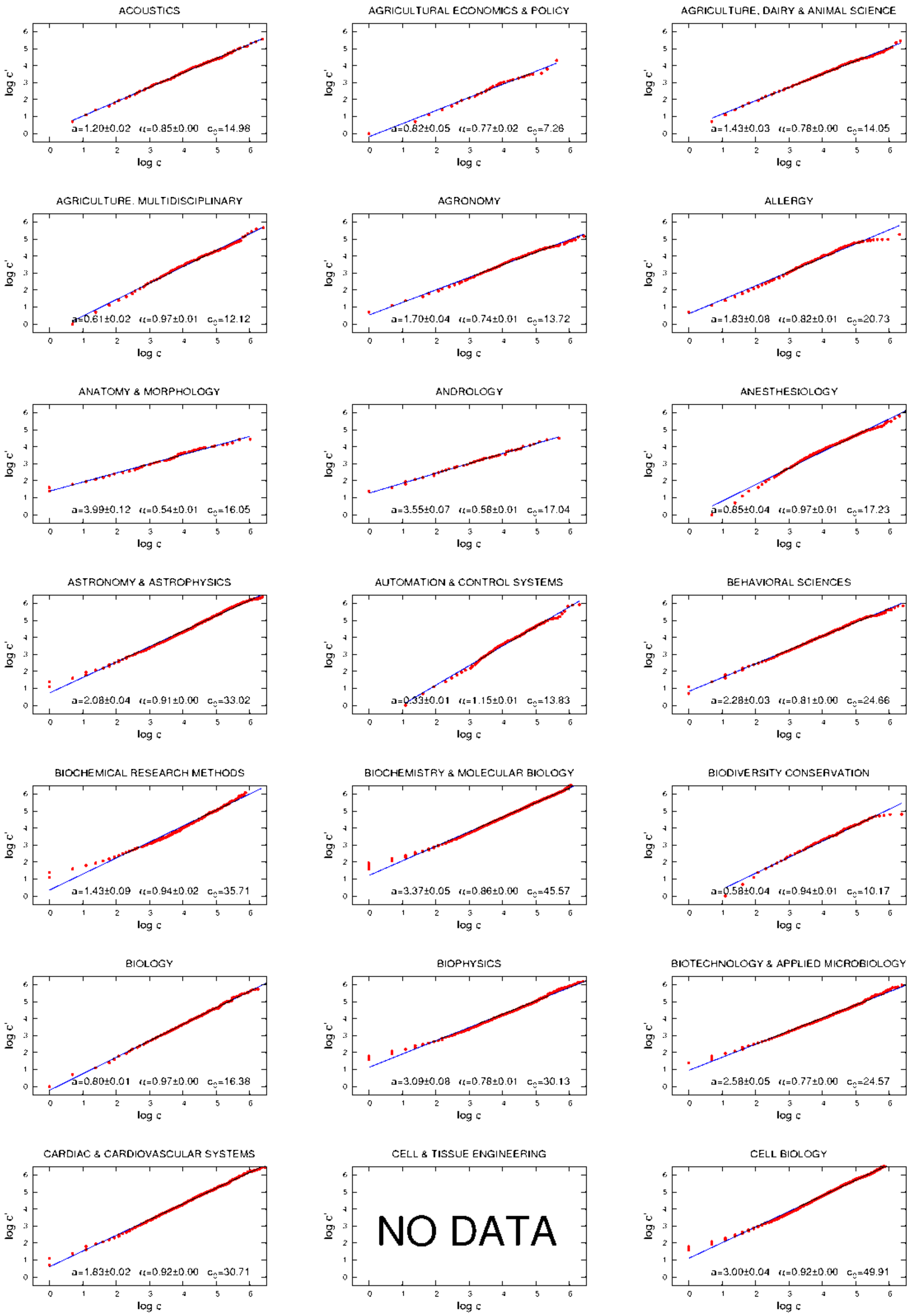

Figure S20: Publication year 1985.

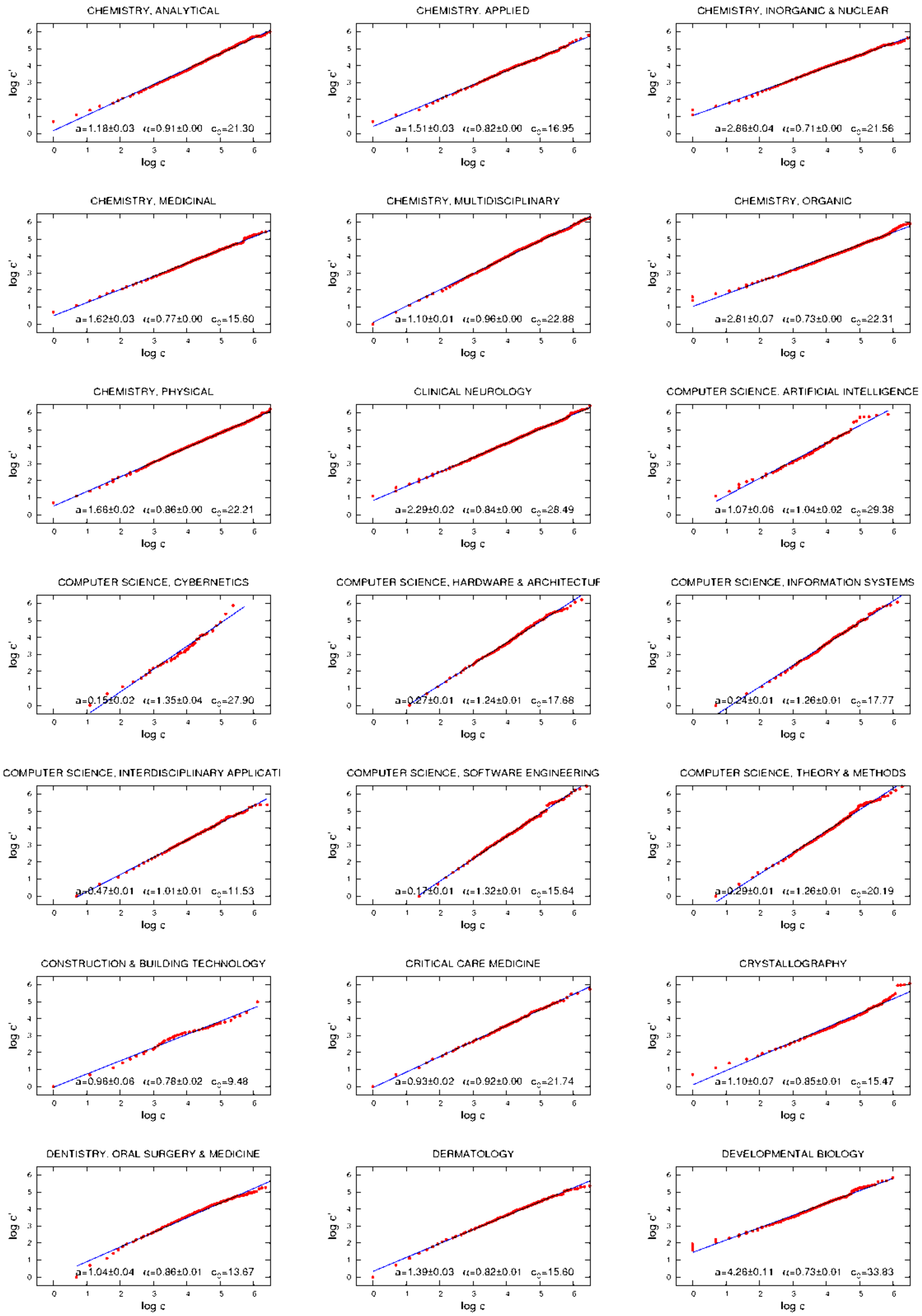

Figure S21: Publication year 1985.

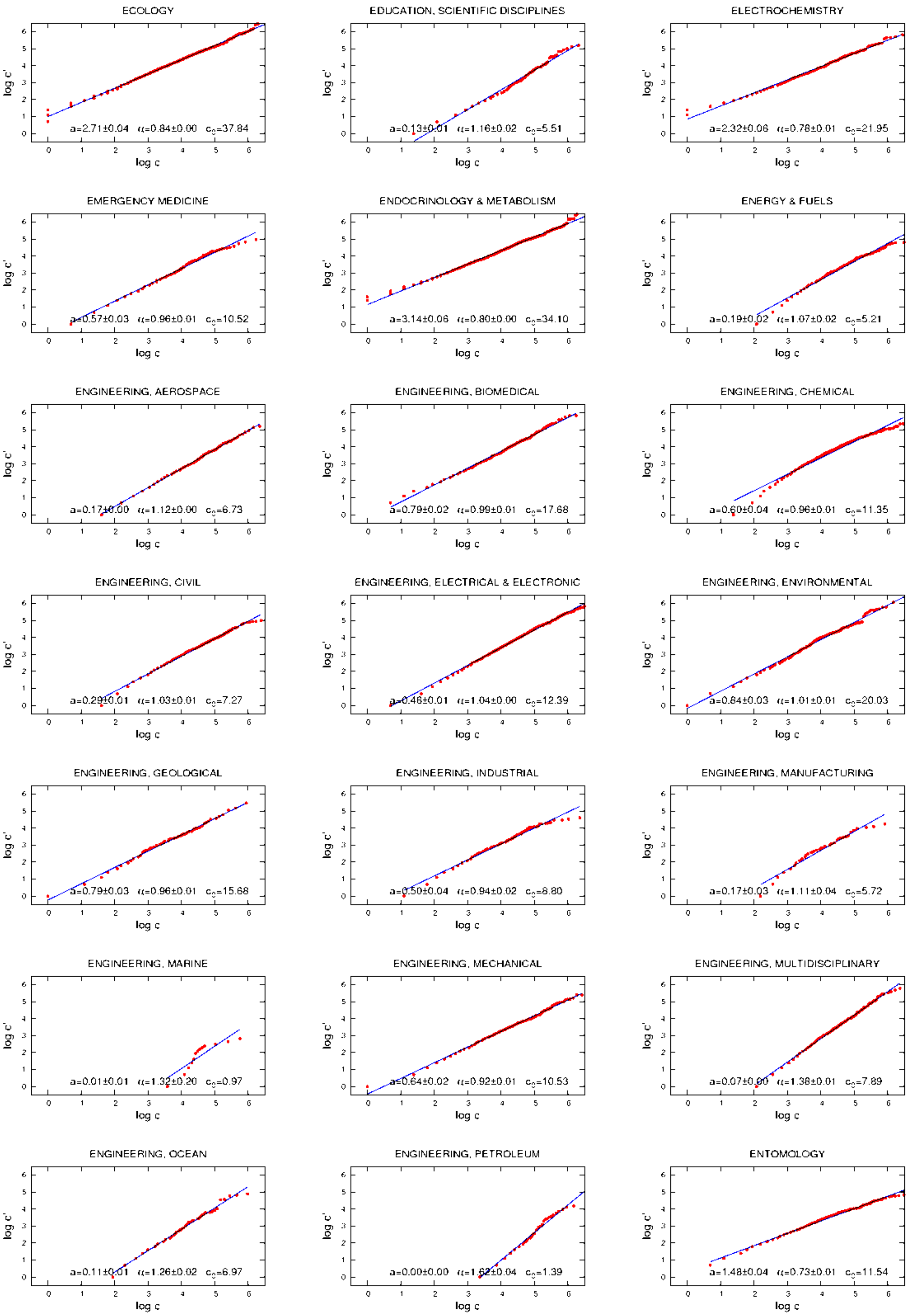

Figure S22: Publication year 1985.

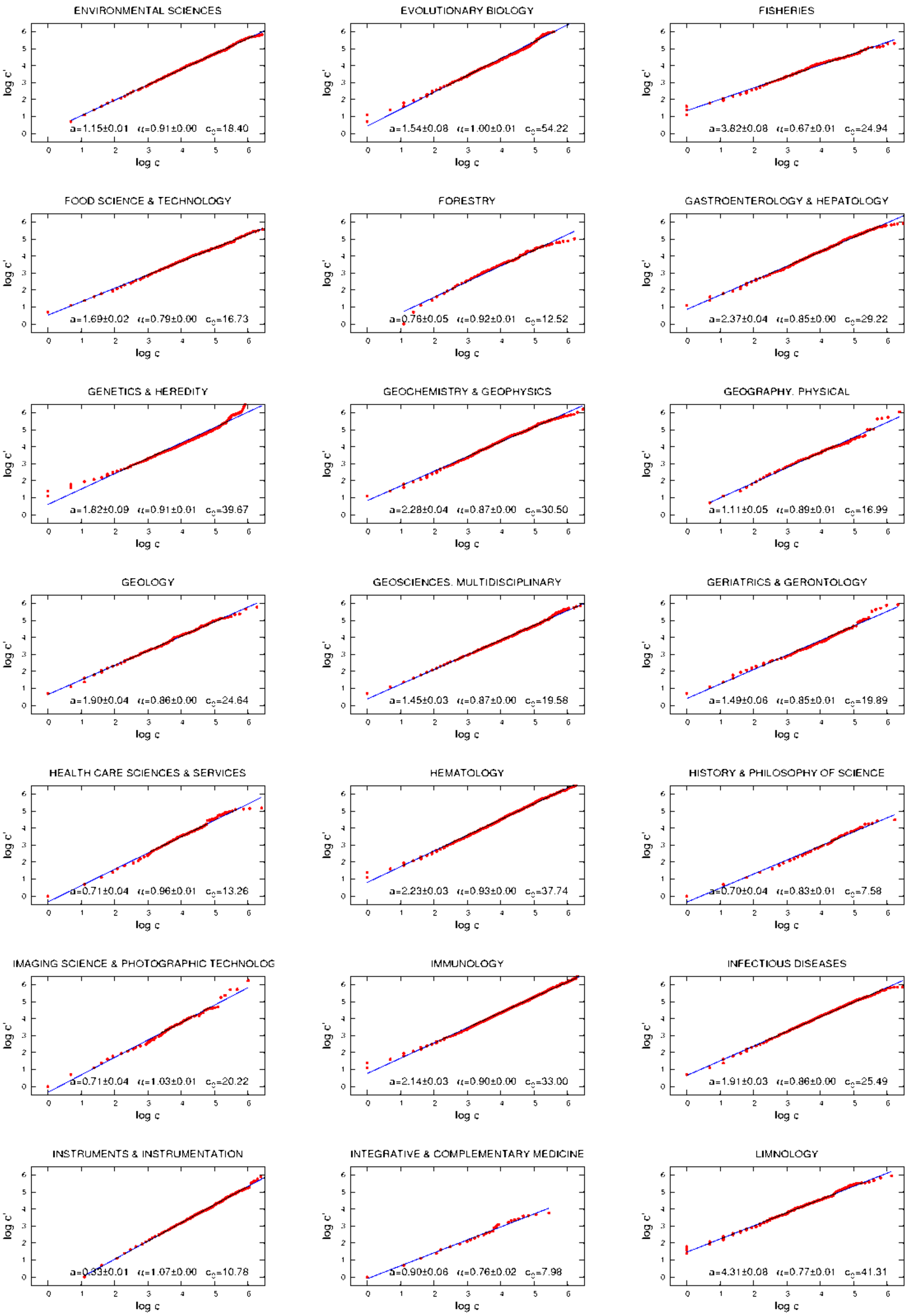

Figure S23: Publication year 1985.

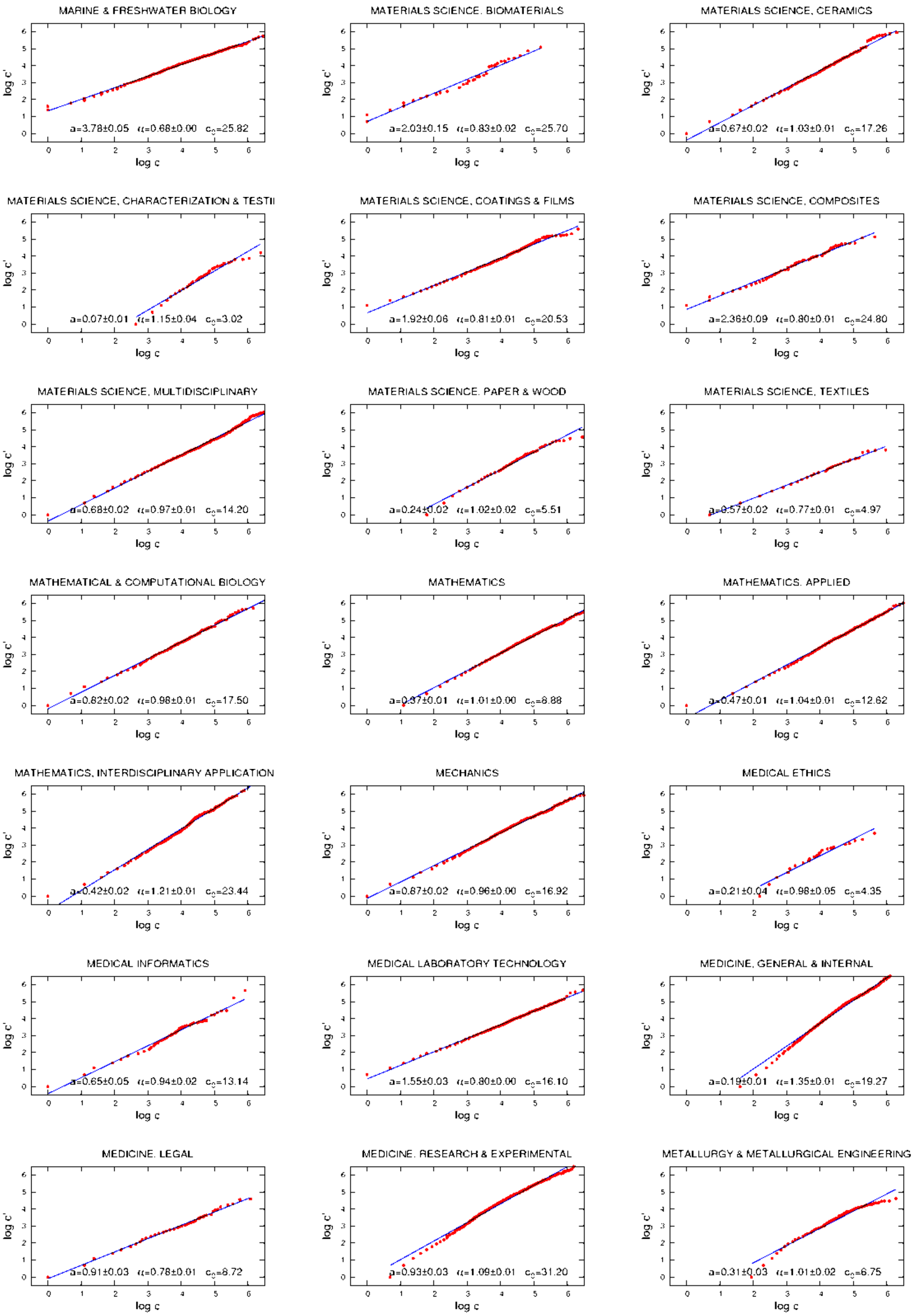

Figure S24: Publication year 1985.

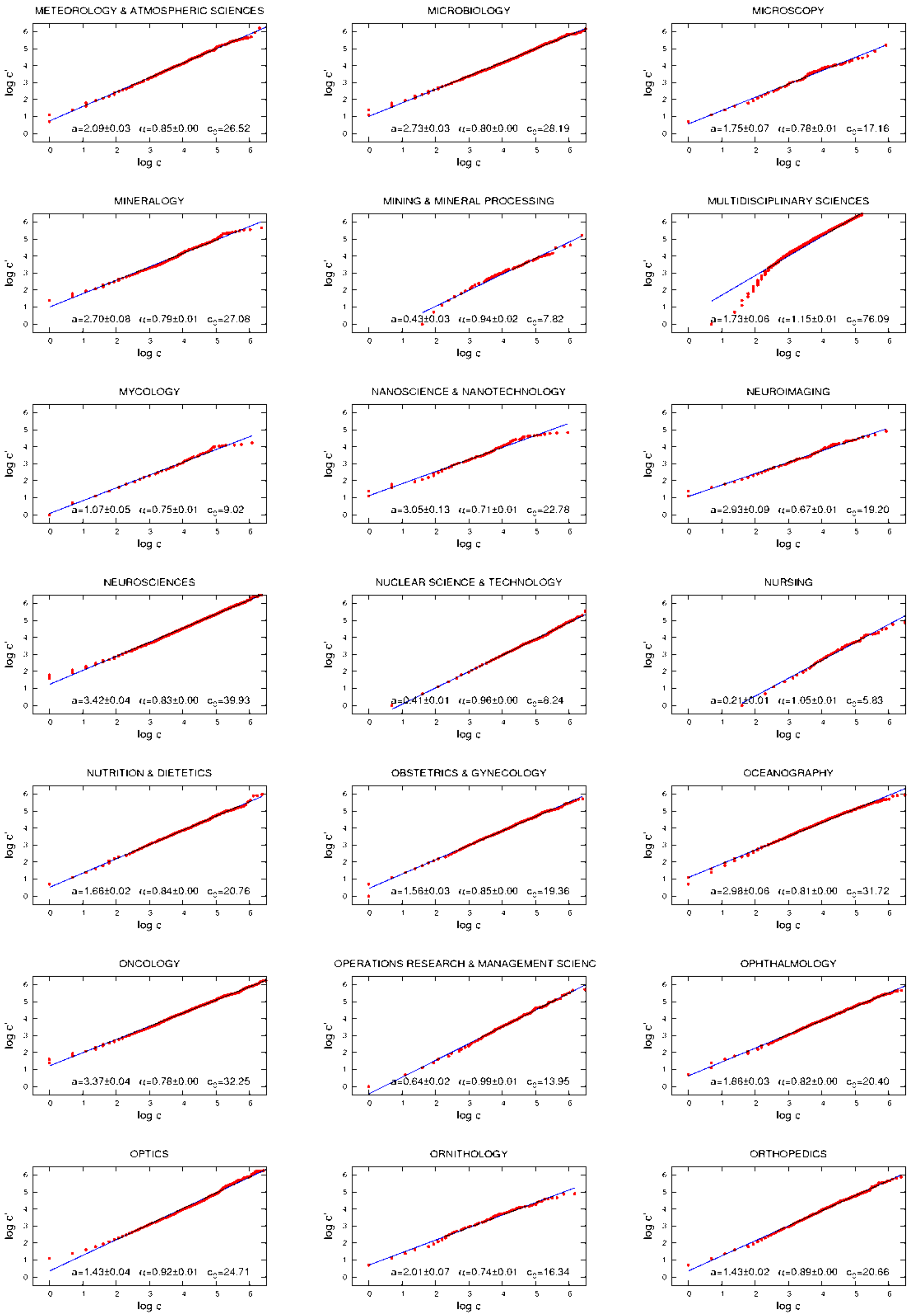

Figure S25: Publication year 1985.

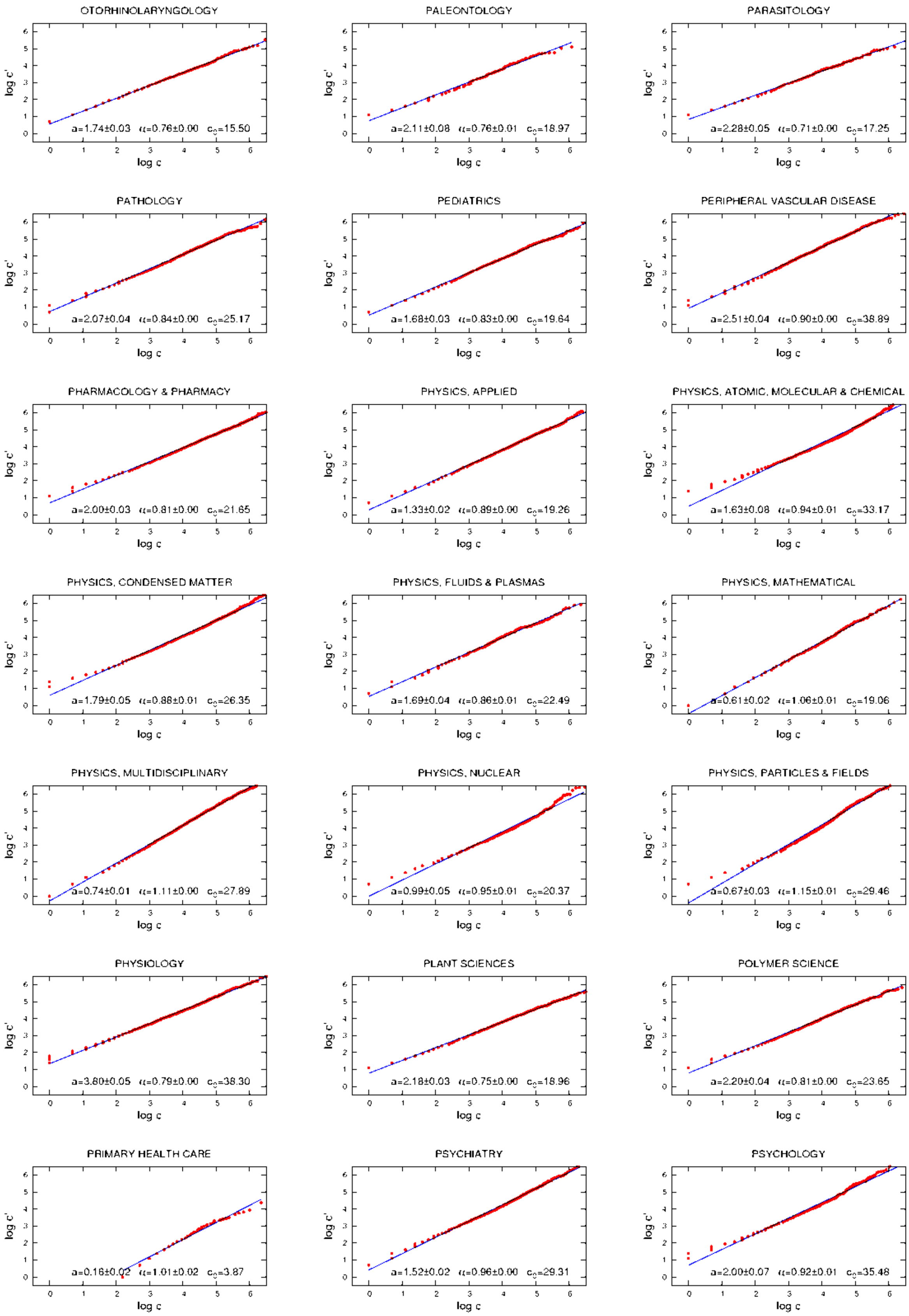

Figure S26: Publication year 1985.

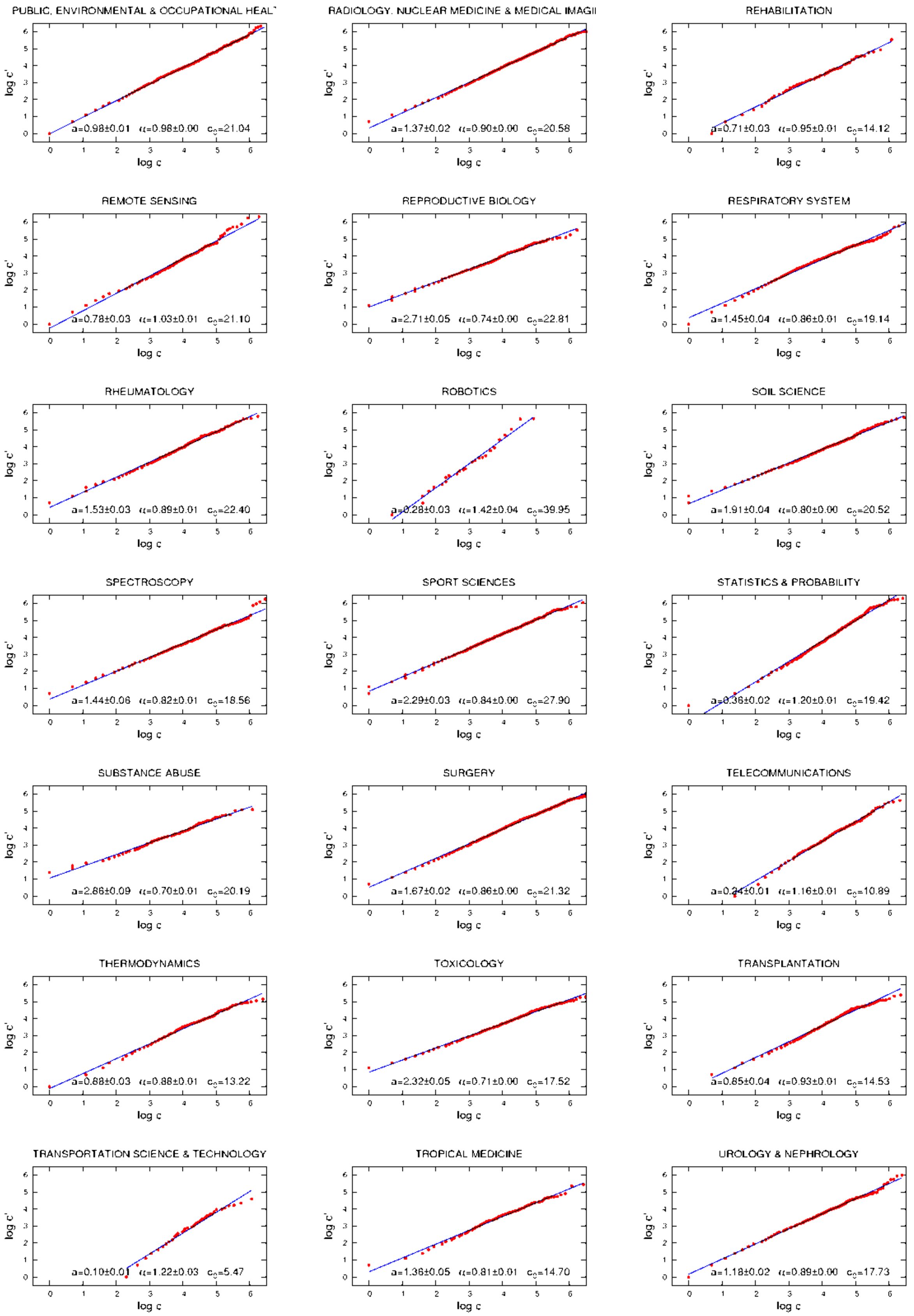

Figure S27: Publication year 1985.

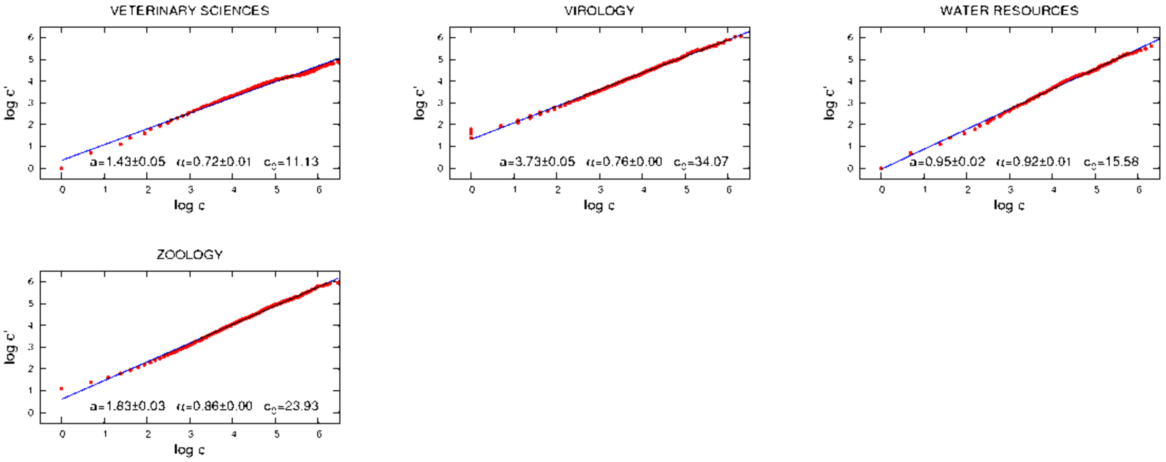

Figure S28: Publication year 1985.

Subject-category	a	α	$\langle c \rangle$	N
ACOUSTICS	1.20 ± 0.02	0.85 ± 0.00	14.98	1,468
AGRICULTURAL ECONOMICS & POLICY	0.82 ± 0.05	0.77 ± 0.02	7.26	450
AGRICULTURE, DAIRY & ANIMAL SCIENCE	1.43 ± 0.03	0.78 ± 0.00	14.05	2,034
AGRICULTURE, MULTIDISCIPLINARY	0.61 ± 0.02	0.97 ± 0.01	12.12	1,529
AGRONOMY	1.70 ± 0.04	0.74 ± 0.01	13.72	2,384
ALLERGY	1.83 ± 0.08	0.82 ± 0.01	20.73	636
ANATOMY & MORPHOLOGY	3.99 ± 0.12	0.54 ± 0.01	16.05	351
ANDROLOGY	3.55 ± 0.07	0.58 ± 0.01	17.04	174
ANESTHESIOLOGY	0.85 ± 0.04	0.97 ± 0.01	17.23	1,911
ASTRONOMY & ASTROPHYSICS	2.08 ± 0.04	0.91 ± 0.00	33.02	5,153
AUTOMATION & CONTROL SYSTEMS	0.33 ± 0.01	1.15 ± 0.01	13.83	1,257
BEHAVIORAL SCIENCES	2.28 ± 0.03	0.81 ± 0.00	24.66	2,331
BIOCHEMICAL RESEARCH METHODS	1.43 ± 0.09	0.94 ± 0.02	35.71	2,078
BIOCHEMISTRY & MOLECULAR BIOLOGY	3.37 ± 0.05	0.86 ± 0.00	45.57	18,570
BIODIVERSITY CONSERVATION	0.58 ± 0.04	0.94 ± 0.01	10.17	716
BIOLOGY	0.80 ± 0.01	0.97 ± 0.00	16.38	2,868
BIOPHYSICS	3.09 ± 0.08	0.78 ± 0.01	30.13	4,662
BIOTECHNOLOGY & APPLIED MICROBIOLOGY	2.58 ± 0.05	0.77 ± 0.00	24.57	2,917
CARDIAC & CARDIOVASCULAR SYSTEMS	1.83 ± 0.02	0.92 ± 0.00	30.71	4,995
CELL & TISSUE ENGINEERING	–	–	–	0
CELL BIOLOGY	3.00 ± 0.04	0.92 ± 0.00	49.91	7,420
CHEMISTRY, ANALYTICAL	1.18 ± 0.03	0.91 ± 0.00	21.30	5,321
CHEMISTRY, APPLIED	1.51 ± 0.03	0.82 ± 0.00	16.95	1,628
CHEMISTRY, INORGANIC & NUCLEAR	2.86 ± 0.04	0.71 ± 0.00	21.56	3,977
CHEMISTRY, MEDICINAL	1.62 ± 0.03	0.77 ± 0.00	15.60	2,840
CHEMISTRY, MULTIDISCIPLINARY	1.10 ± 0.01	0.96 ± 0.00	22.88	9,962
CHEMISTRY, ORGANIC	2.81 ± 0.07	0.73 ± 0.00	22.31	7,231
CHEMISTRY, PHYSICAL	1.66 ± 0.02	0.86 ± 0.00	22.21	8,988
CLINICAL NEUROLOGY	2.29 ± 0.02	0.84 ± 0.00	28.49	5,375
COMPUTER SCIENCE, ARTIFICIAL INTELLIGENCE	1.07 ± 0.06	1.04 ± 0.02	29.38	243
COMPUTER SCIENCE, CYBERNETICS	0.15 ± 0.02	1.35 ± 0.04	27.90	195
COMPUTER SCIENCE, HARDWARE & ARCHITECTURE	0.27 ± 0.01	1.24 ± 0.01	17.68	1,134
COMPUTER SCIENCE, INFORMATION SYSTEMS	0.24 ± 0.01	1.26 ± 0.01	17.77	876
COMPUTER SCIENCE, INTERDISCIPLINARY APPLICATIONS	0.47 ± 0.01	1.01 ± 0.01	11.53	1,529
COMPUTER SCIENCE, SOFTWARE ENGINEERING	0.17 ± 0.01	1.32 ± 0.01	15.64	1,502
COMPUTER SCIENCE, THEORY & METHODS	0.29 ± 0.01	1.26 ± 0.01	20.19	1,180
CONSTRUCTION & BUILDING TECHNOLOGY	0.96 ± 0.06	0.78 ± 0.02	9.48	433
CRITICAL CARE MEDICINE	0.93 ± 0.02	0.92 ± 0.00	21.74	891
CRYSTALLOGRAPHY	1.10 ± 0.07	0.85 ± 0.01	15.47	2,672
DENTISTRY, ORAL SURGERY & MEDICINE	1.04 ± 0.04	0.86 ± 0.01	13.67	2,833
DERMATOLOGY	1.39 ± 0.03	0.82 ± 0.01	15.60	2,651
DEVELOPMENTAL BIOLOGY	4.25 ± 0.11	0.73 ± 0.01	33.83	665
ECOLOGY	2.71 ± 0.04	0.84 ± 0.00	37.84	3,726
EDUCATION, SCIENTIFIC DISCIPLINES	0.13 ± 0.01	1.16 ± 0.02	5.51	1,310
ELECTROCHEMISTRY	2.32 ± 0.06	0.78 ± 0.01	21.95	1,616
EMERGENCY MEDICINE	0.57 ± 0.03	0.96 ± 0.01	10.52	560
ENDOCRINOLOGY & METABOLISM	3.14 ± 0.06	0.80 ± 0.00	34.10	4,871
ENERGY & FUELS	0.19 ± 0.02	1.07 ± 0.02	5.21	2,698
ENGINEERING, AEROSPACE	0.17 ± 0.00	1.12 ± 0.00	6.73	1,395
ENGINEERING, BIOMEDICAL	0.79 ± 0.02	0.99 ± 0.01	17.68	1,128

Table S17: Publication year 1985.

Subject-category	a	α	$\langle c \rangle$	N
ENGINEERING, CHEMICAL	0.60 ± 0.04	0.96 ± 0.01	11.35	5,088
ENGINEERING, CIVIL	0.29 ± 0.01	1.03 ± 0.01	7.27	2,239
ENGINEERING, ELECTRICAL & ELECTRONIC	0.46 ± 0.01	1.04 ± 0.00	12.39	9,114
ENGINEERING, ENVIRONMENTAL	0.84 ± 0.03	1.01 ± 0.01	20.03	964
ENGINEERING, GEOLOGICAL	0.79 ± 0.03	0.96 ± 0.01	15.68	307
ENGINEERING, INDUSTRIAL	0.50 ± 0.04	0.94 ± 0.02	8.80	705
ENGINEERING, MANUFACTURING	0.17 ± 0.03	1.11 ± 0.04	5.72	282
ENGINEERING, MARINE	0.01 ± 0.01	1.32 ± 0.20	0.97	200
ENGINEERING, MECHANICAL	0.64 ± 0.02	0.92 ± 0.01	10.53	2,334
ENGINEERING, MULTIDISCIPLINARY	0.07 ± 0.00	1.38 ± 0.01	7.89	2,129
ENGINEERING, OCEAN	0.11 ± 0.01	1.26 ± 0.02	6.97	329
ENGINEERING, PETROLEUM	0.00 ± 0.00	1.62 ± 0.04	1.39	961
ENTOMOLOGY	1.48 ± 0.04	0.73 ± 0.01	11.54	2,664
ENVIRONMENTAL SCIENCES	1.15 ± 0.01	0.91 ± 0.00	18.40	4,750
EVOLUTIONARY BIOLOGY	1.54 ± 0.08	1.00 ± 0.01	54.22	1,138
FISHERIES	3.82 ± 0.08	0.67 ± 0.01	24.94	1,058
FOOD SCIENCE & TECHNOLOGY	1.69 ± 0.02	0.79 ± 0.00	16.73	3,196
FORESTRY	0.76 ± 0.05	0.92 ± 0.01	12.52	1,030
GASTROENTEROLOGY & HEPATOLOGY	2.37 ± 0.04	0.85 ± 0.00	29.22	2,602
GENETICS & HEREDITY	1.82 ± 0.09	0.91 ± 0.01	39.67	3,136
GEOCHEMISTRY & GEOPHYSICS	2.28 ± 0.04	0.87 ± 0.00	30.50	2,507
GEOGRAPHY, PHYSICAL	1.11 ± 0.05	0.89 ± 0.01	16.99	718
GEOLOGY	1.90 ± 0.04	0.86 ± 0.00	24.64	595
GEOSCIENCES, MULTIDISCIPLINARY	1.45 ± 0.03	0.87 ± 0.00	19.58	2,968
GERIATRICS & GERONTOLOGY	1.49 ± 0.06	0.85 ± 0.01	19.89	663
HEALTH CARE SCIENCES & SERVICES	0.71 ± 0.04	0.96 ± 0.01	13.26	762
HEMATOLOGY	2.23 ± 0.03	0.93 ± 0.00	37.74	3,406
HISTORY & PHILOSOPHY OF SCIENCE	0.70 ± 0.04	0.83 ± 0.01	7.58	540
IMAGING SCIENCE & PHOTOGRAPHIC TECHNOLOGY	0.71 ± 0.04	1.03 ± 0.01	20.22	342
IMMUNOLOGY	2.14 ± 0.03	0.90 ± 0.00	33.00	8,667
INFECTIOUS DISEASES	1.91 ± 0.03	0.86 ± 0.00	25.49	2,547
INSTRUMENTS & INSTRUMENTATION	0.33 ± 0.01	1.07 ± 0.00	10.78	3,692
INTEGRATIVE & COMPLEMENTARY MEDICINE	0.90 ± 0.06	0.76 ± 0.02	7.98	108
LIMNOLOGY	4.31 ± 0.08	0.77 ± 0.01	41.31	444
MARINE & FRESHWATER BIOLOGY	3.78 ± 0.05	0.68 ± 0.00	25.82	3,267
MATERIALS SCIENCE, BIOMATERIALS	2.03 ± 0.15	0.83 ± 0.02	25.70	70
MATERIALS SCIENCE, CERAMICS	0.67 ± 0.02	1.03 ± 0.01	17.26	1,250
MATERIALS SCIENCE, CHARACTERIZATION & TESTING	0.07 ± 0.01	1.15 ± 0.04	3.02	737
MATERIALS SCIENCE, COATINGS & FILMS	1.92 ± 0.06	0.81 ± 0.01	20.53	1,315
MATERIALS SCIENCE, COMPOSITES	2.36 ± 0.09	0.80 ± 0.01	24.80	158
MATERIALS SCIENCE, MULTIDISCIPLINARY	0.68 ± 0.02	0.97 ± 0.01	14.20	5,919
MATERIALS SCIENCE, PAPER & WOOD	0.24 ± 0.02	1.02 ± 0.02	5.51	817
MATERIALS SCIENCE, TEXTILES	0.57 ± 0.02	0.77 ± 0.01	4.97	322
MATHEMATICAL & COMPUTATIONAL BIOLOGY	0.82 ± 0.02	0.98 ± 0.01	17.50	931
MATHEMATICS	0.37 ± 0.01	1.01 ± 0.00	8.88	7,895
MATHEMATICS, APPLIED	0.47 ± 0.01	1.04 ± 0.01	12.62	3,619
MATHEMATICS, INTERDISCIPLINARY APPLICATIONS	0.42 ± 0.02	1.21 ± 0.01	23.44	1,332
MECHANICS	0.87 ± 0.02	0.96 ± 0.00	16.92	3,640
MEDICAL ETHICS	0.21 ± 0.04	0.98 ± 0.05	4.35	158
MEDICAL INFORMATICS	0.65 ± 0.05	0.94 ± 0.02	13.14	283

Table S18: Publication year 1985.

Subject-category	a	α	$\langle c \rangle$	N
MEDICAL LABORATORY TECHNOLOGY	1.55 ± 0.03	0.80 ± 0.00	16.10	1,657
MEDICINE, GENERAL & INTERNAL	0.19 ± 0.01	1.35 ± 0.01	19.27	15,623
MEDICINE, LEGAL	0.91 ± 0.03	0.78 ± 0.01	8.72	403
MEDICINE, RESEARCH & EXPERIMENTAL	0.93 ± 0.03	1.09 ± 0.01	31.20	4,958
METALLURGY & METALLURGICAL ENGINEERING	0.31 ± 0.03	1.01 ± 0.02	6.75	1,198
METEOROLOGY & ATMOSPHERIC SCIENCES	2.09 ± 0.03	0.85 ± 0.00	26.52	1,880
MICROBIOLOGY	2.73 ± 0.03	0.80 ± 0.00	28.19	5,452
MICROSCOPY	1.75 ± 0.07	0.78 ± 0.01	17.16	286
MINERALOGY	2.70 ± 0.08	0.79 ± 0.01	27.08	708
MINING & MINERAL PROCESSING	0.43 ± 0.03	0.94 ± 0.02	7.82	740
MULTIDISCIPLINARY SCIENCES	1.73 ± 0.06	1.15 ± 0.01	76.09	7,785
MYCOLOGY	1.07 ± 0.05	0.75 ± 0.01	9.02	401
NANOSCIENCE & NANOTECHNOLOGY	3.05 ± 0.13	0.71 ± 0.01	22.78	316
NEUROIMAGING	2.93 ± 0.09	0.67 ± 0.01	19.20	292
NEUROSCIENCES	3.42 ± 0.04	0.83 ± 0.00	39.93	10,403
NUCLEAR SCIENCE & TECHNOLOGY	0.41 ± 0.01	0.96 ± 0.00	8.24	5,203
NURSING	0.21 ± 0.01	1.05 ± 0.01	5.83	877
NUTRITION & DIETETICS	1.66 ± 0.02	0.84 ± 0.00	20.76	2,159
OBSTETRICS & GYNECOLOGY	1.56 ± 0.03	0.85 ± 0.00	19.36	3,074
OCEANOGRAPHY	2.98 ± 0.06	0.81 ± 0.00	31.72	1,746
ONCOLOGY	3.37 ± 0.04	0.78 ± 0.00	32.25	6,156
OPERATIONS RESEARCH & MANAGEMENT SCIENCE	0.64 ± 0.02	0.99 ± 0.01	13.95	1,712
OPHTHALMOLOGY	1.86 ± 0.03	0.82 ± 0.00	20.40	2,914
OPTICS	1.43 ± 0.04	0.92 ± 0.01	24.71	4,696
ORNITHOLOGY	2.01 ± 0.07	0.74 ± 0.01	16.34	475
ORTHOPEDICS	1.43 ± 0.02	0.89 ± 0.00	20.66	2,167
OTORHINOLARYNGOLOGY	1.74 ± 0.03	0.76 ± 0.00	15.50	1,689
PALEONTOLOGY	2.11 ± 0.08	0.76 ± 0.01	18.97	406
PARASITOLOGY	2.28 ± 0.05	0.71 ± 0.00	17.25	958
PATHOLOGY	2.07 ± 0.04	0.84 ± 0.00	25.17	3,345
PEDIATRICS	1.68 ± 0.03	0.83 ± 0.00	19.64	3,886
PERIPHERAL VASCULAR DISEASE	2.51 ± 0.04	0.90 ± 0.00	38.89	2,446
PHARMACOLOGY & PHARMACY	2.00 ± 0.03	0.81 ± 0.00	21.65	13,427
PHYSICS, APPLIED	1.33 ± 0.02	0.89 ± 0.00	19.26	7,536
PHYSICS, ATOMIC, MOLECULAR & CHEMICAL	1.63 ± 0.08	0.94 ± 0.01	33.17	6,966
PHYSICS, CONDENSED MATTER	1.79 ± 0.05	0.88 ± 0.01	26.35	6,787
PHYSICS, FLUIDS & PLASMAS	1.69 ± 0.04	0.86 ± 0.01	22.49	1,383
PHYSICS, MATHEMATICAL	0.61 ± 0.02	1.06 ± 0.01	19.06	1,394
PHYSICS, MULTIDISCIPLINARY	0.74 ± 0.01	1.11 ± 0.00	27.89	7,853
PHYSICS, NUCLEAR	0.99 ± 0.05	0.95 ± 0.01	20.37	2,516
PHYSICS, PARTICLES & FIELDS	0.67 ± 0.03	1.15 ± 0.01	29.46	2,596
PHYSIOLOGY	3.80 ± 0.05	0.79 ± 0.00	38.30	4,505
PLANT SCIENCES	2.18 ± 0.03	0.75 ± 0.00	18.96	7,989
POLYMER SCIENCE	2.20 ± 0.04	0.81 ± 0.00	23.65	3,010
PRIMARY HEALTH CARE	0.16 ± 0.02	1.01 ± 0.02	3.87	690
PSYCHIATRY	1.52 ± 0.02	0.96 ± 0.00	29.31	4,330
PSYCHOLOGY	2.00 ± 0.07	0.92 ± 0.01	35.48	1,442
PUBLIC, ENVIRONMENTAL & OCCUPATIONAL HEALTH	0.98 ± 0.01	0.98 ± 0.00	21.04	4,024
RADIOLOGY, NUCLEAR MEDICINE & MEDICAL IMAGING	1.37 ± 0.02	0.90 ± 0.00	20.58	5,504
REHABILITATION	0.71 ± 0.03	0.95 ± 0.01	14.12	392

Table S19: Publication year 1985.

Subject-category	a	α	$\langle c \rangle$	N
REMOTE SENSING	0.78 ± 0.03	1.03 ± 0.01	21.10	611
REPRODUCTIVE BIOLOGY	2.71 ± 0.05	0.74 ± 0.00	22.81	1,085
RESPIRATORY SYSTEM	1.45 ± 0.04	0.86 ± 0.01	19.14	1,844
RHEUMATOLOGY	1.53 ± 0.03	0.89 ± 0.01	22.40	1,126
ROBOTICS	0.28 ± 0.03	1.42 ± 0.04	39.95	43
SOIL SCIENCE	1.91 ± 0.04	0.80 ± 0.00	20.52	1,603
SPECTROSCOPY	1.44 ± 0.06	0.82 ± 0.01	18.56	2,549
SPORT SCIENCES	2.29 ± 0.03	0.84 ± 0.00	27.90	1,517
STATISTICS & PROBABILITY	0.36 ± 0.02	1.20 ± 0.01	19.42	2,295
SUBSTANCE ABUSE	2.86 ± 0.09	0.70 ± 0.01	20.19	410
SURGERY	1.67 ± 0.02	0.86 ± 0.00	21.32	9,570
TELECOMMUNICATIONS	0.24 ± 0.01	1.16 ± 0.01	10.89	1,319
THERMODYNAMICS	0.88 ± 0.03	0.88 ± 0.01	13.22	1,500
TOXICOLOGY	2.32 ± 0.05	0.71 ± 0.00	17.52	2,618
TRANSPLANTATION	0.85 ± 0.04	0.93 ± 0.01	14.53	1,375
TRANSPORTATION SCIENCE & TECHNOLOGY	0.10 ± 0.01	1.22 ± 0.03	5.47	386
TROPICAL MEDICINE	1.36 ± 0.05	0.81 ± 0.01	14.70	790
UROLOGY & NEPHROLOGY	1.18 ± 0.02	0.89 ± 0.00	17.73	2,238
VETERINARY SCIENCES	1.43 ± 0.05	0.72 ± 0.01	11.13	4,175
VIROLOGY	3.73 ± 0.05	0.76 ± 0.00	34.07	1,862
WATER RESOURCES	0.95 ± 0.02	0.92 ± 0.01	15.58	1,944
ZOOLOGY	1.83 ± 0.03	0.86 ± 0.00	23.93	4,233
**TOTAL**	$\mathbf{1}.00 \pm 0.00$	$\mathbf{1}.00 \pm 0.00$	**2**4.13	**4**70,680

Table S20: Publication year 1985.

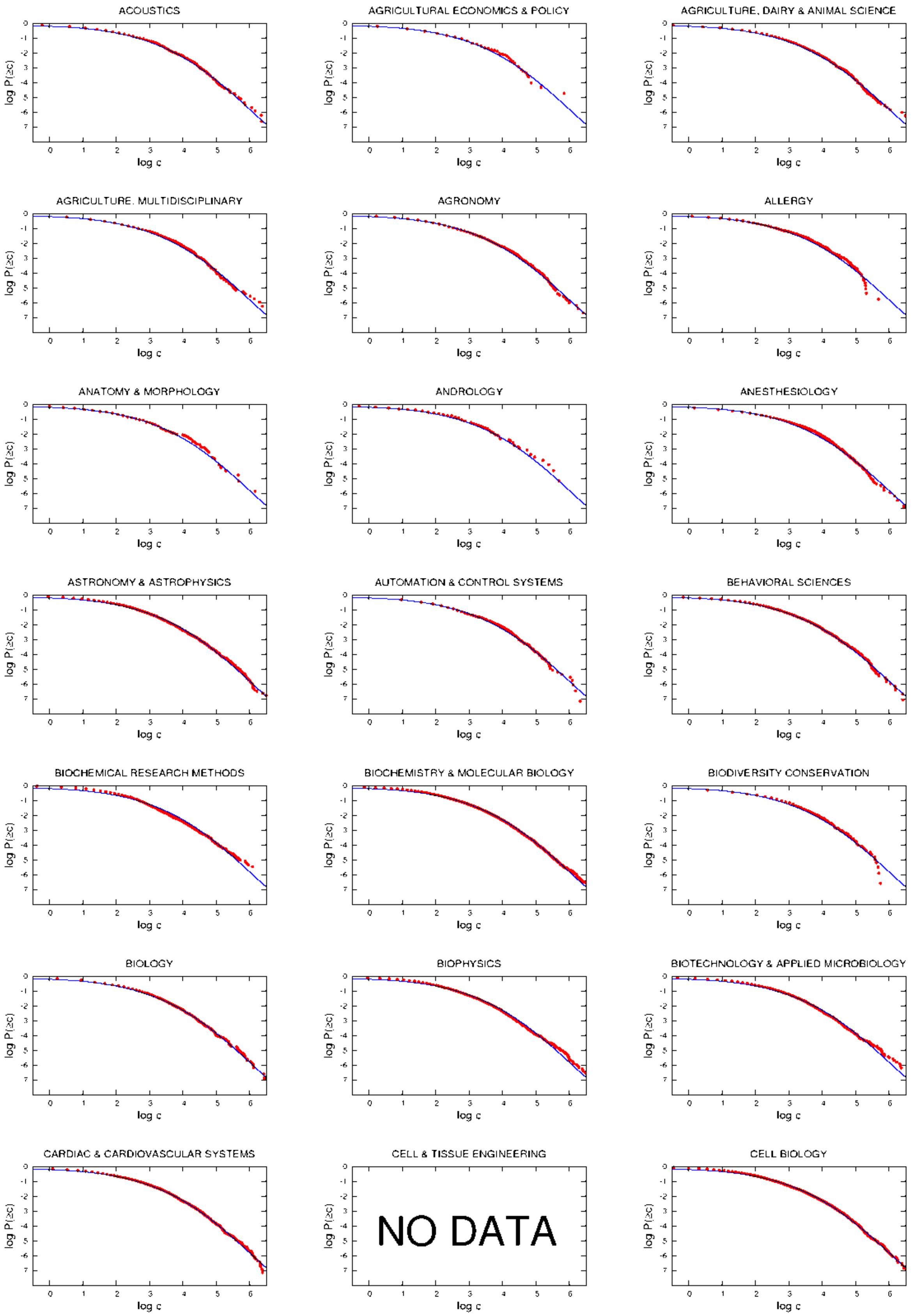

Figure S29: Publication year 1985.

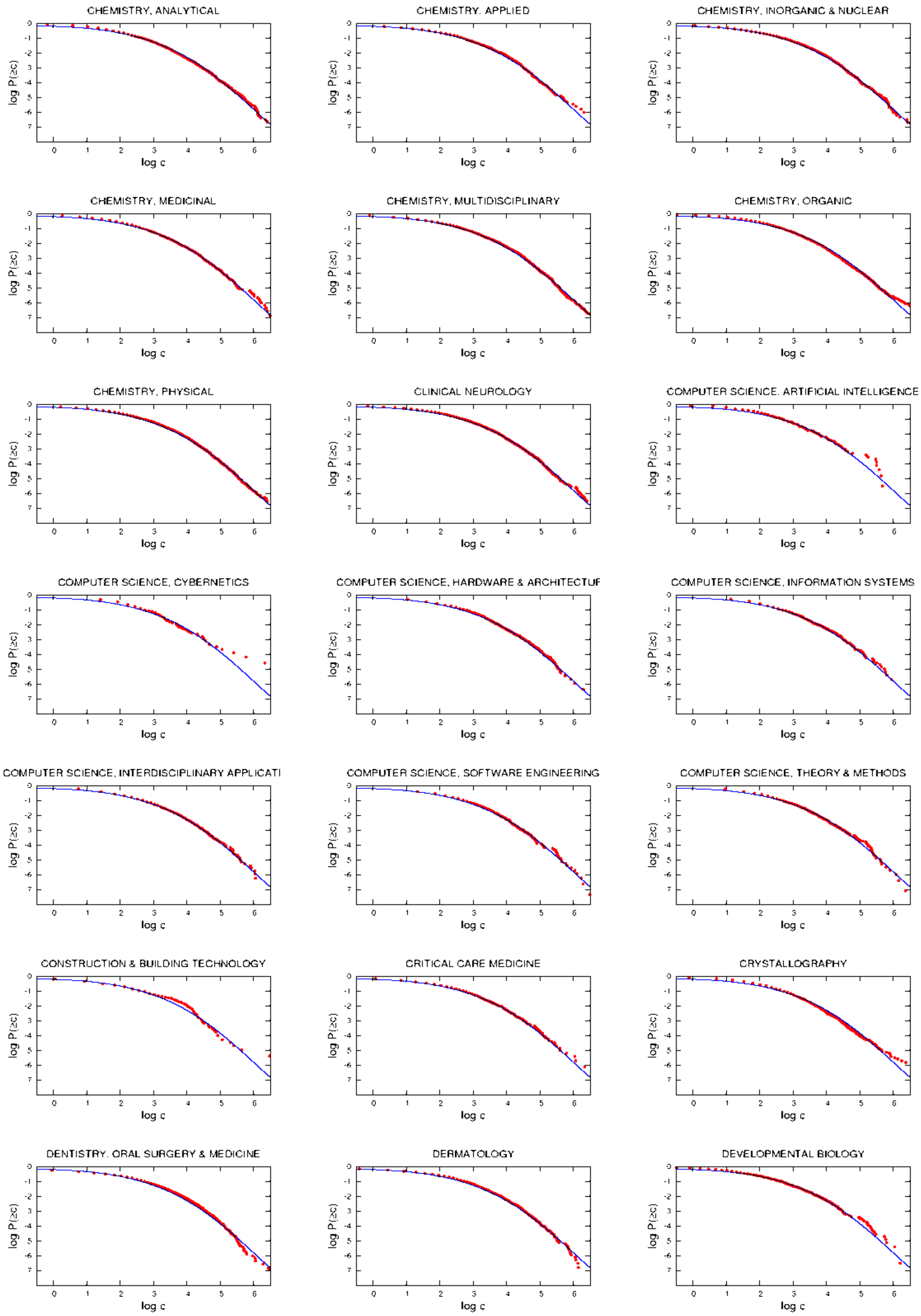

Figure S30: Publication year 1985.

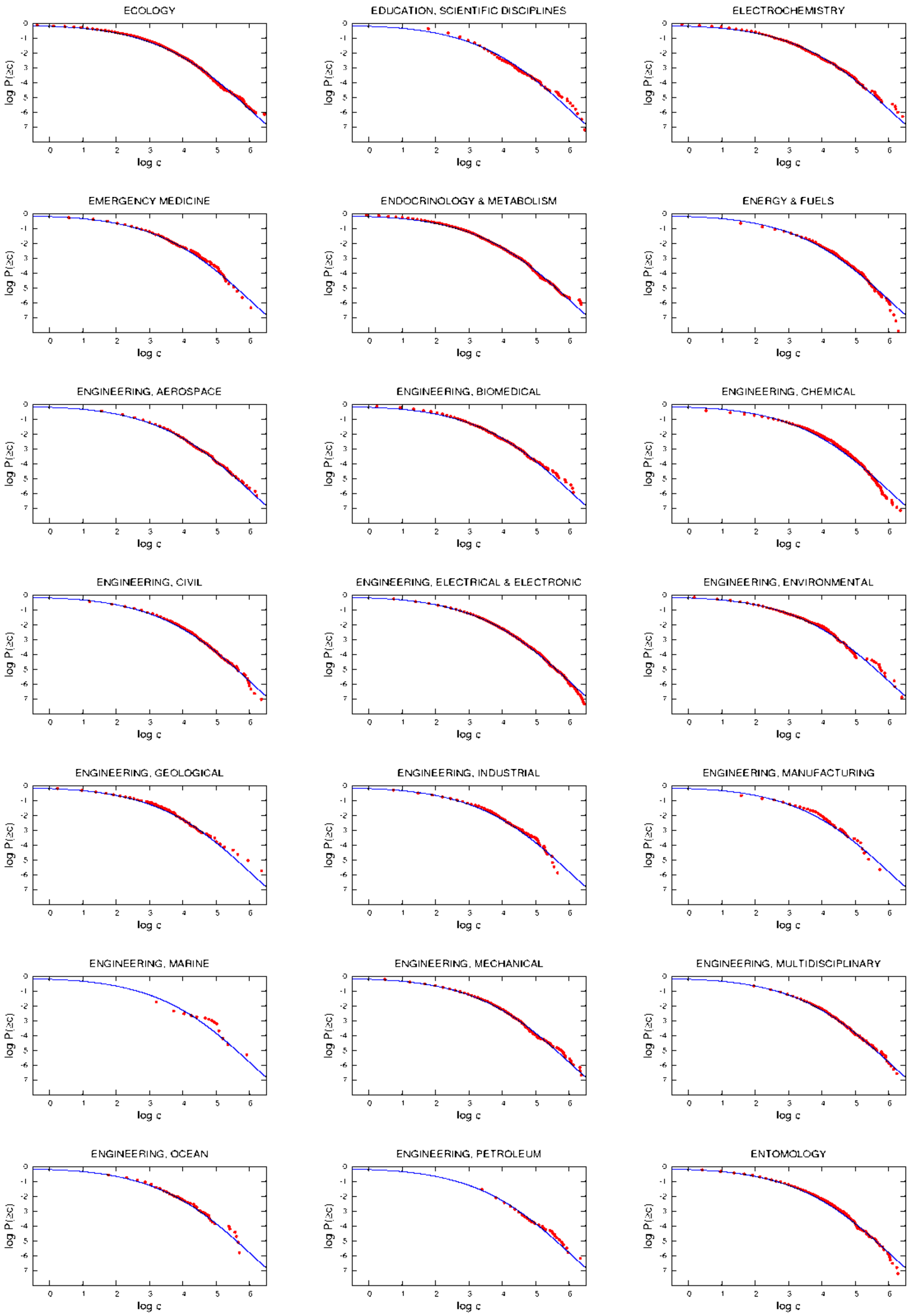

Figure S31: Publication year 1985.

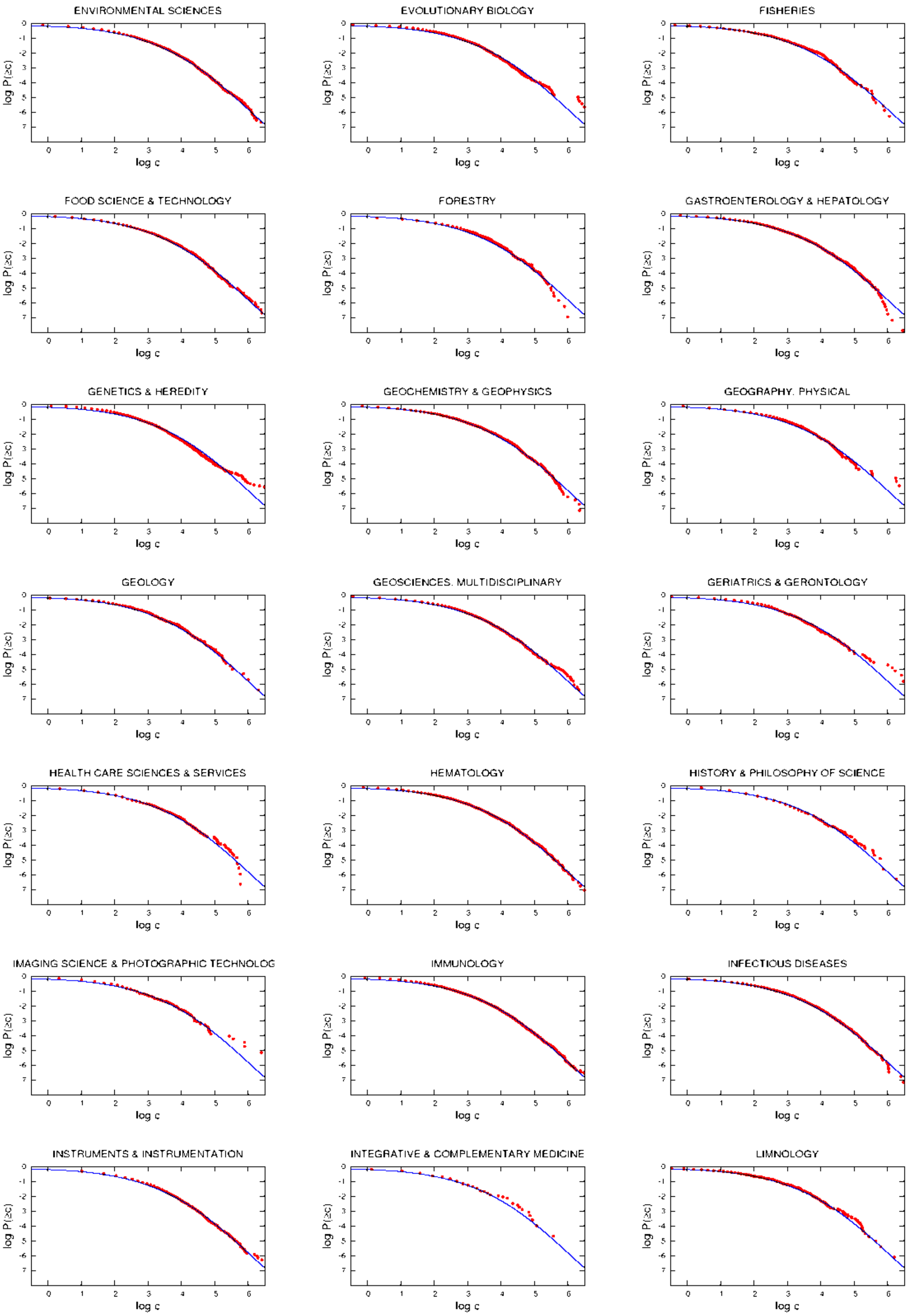

Figure S32: Publication year 1985.

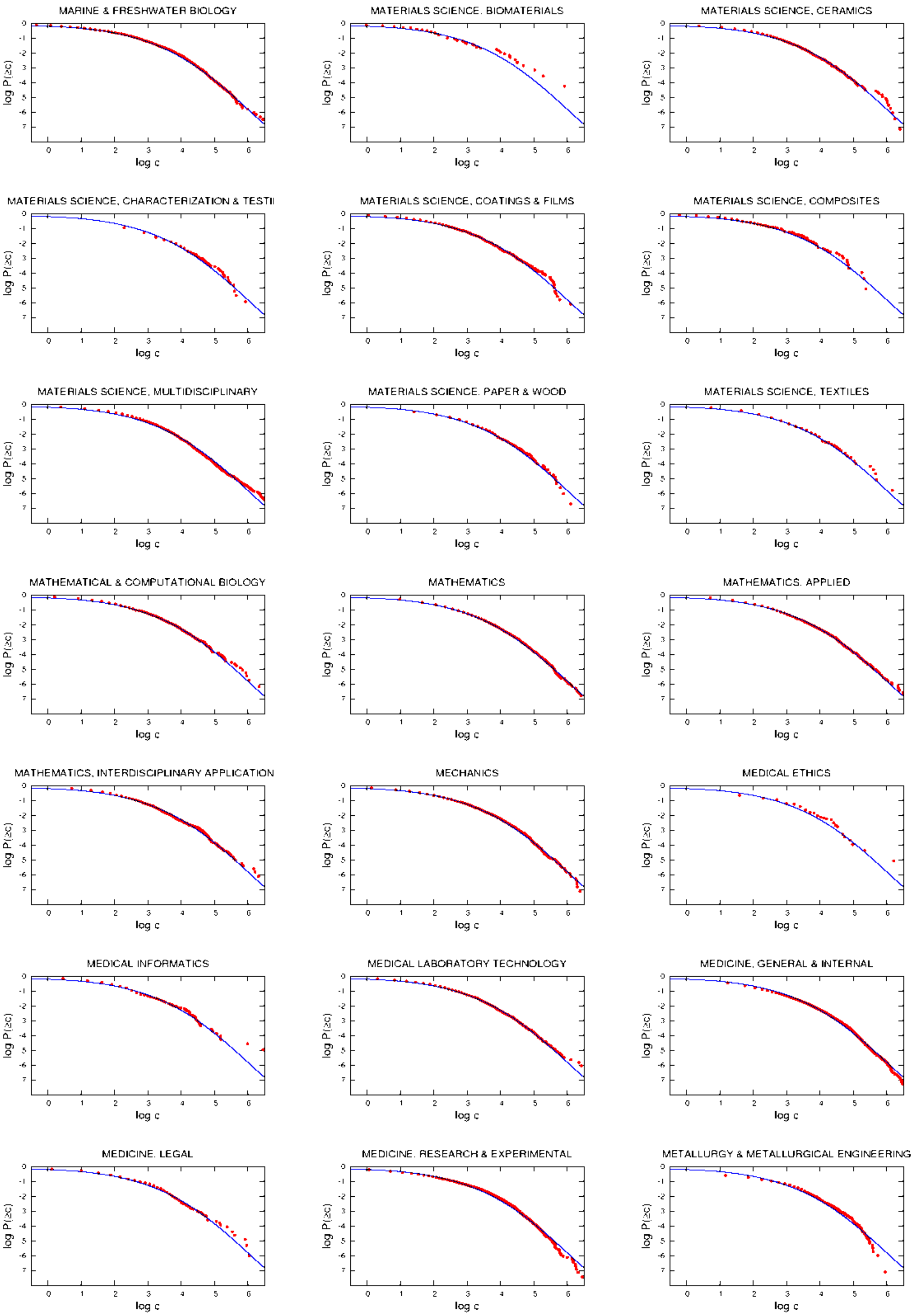

Figure S33: Publication year 1985.

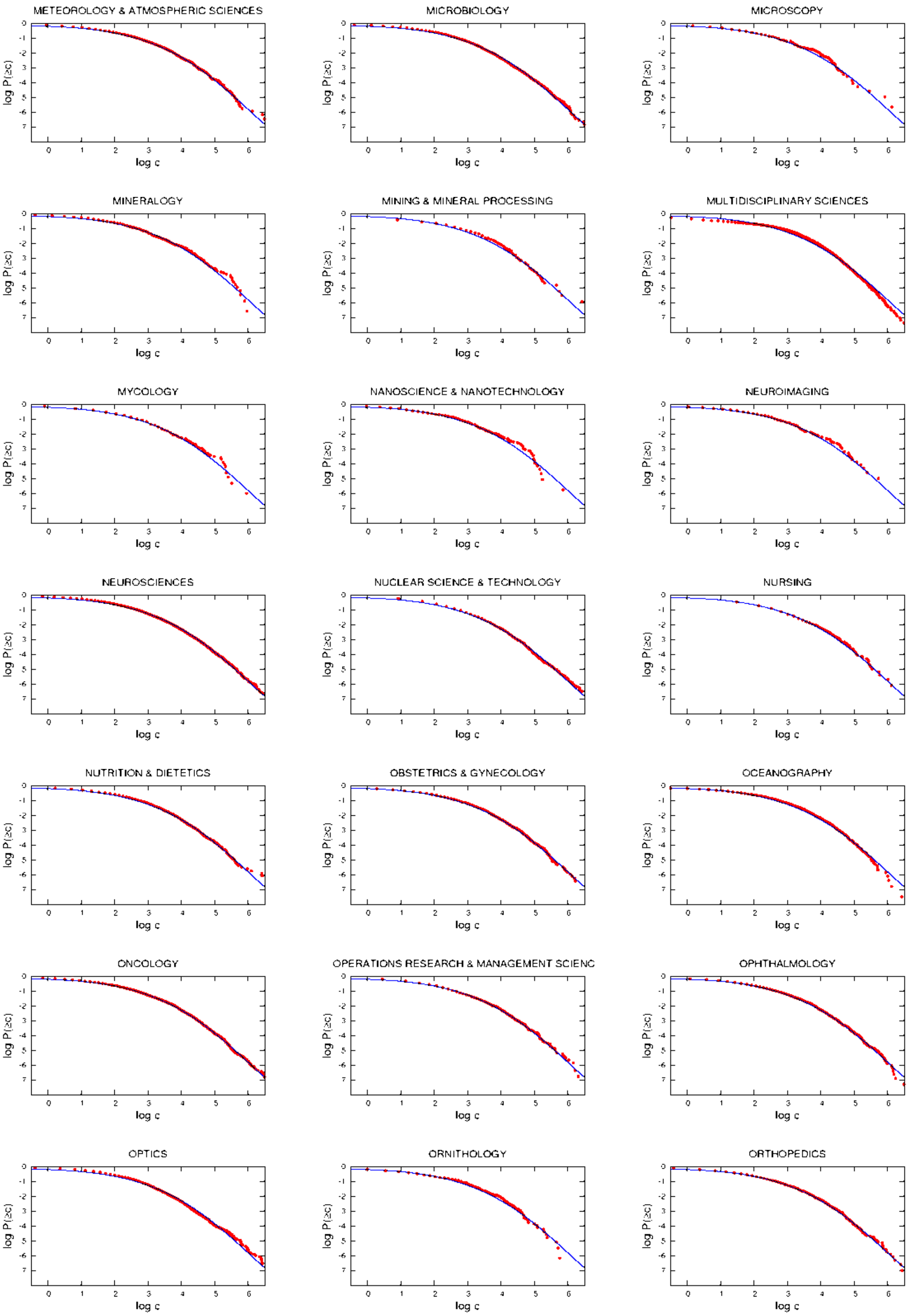

Figure S34: Publication year 1985.

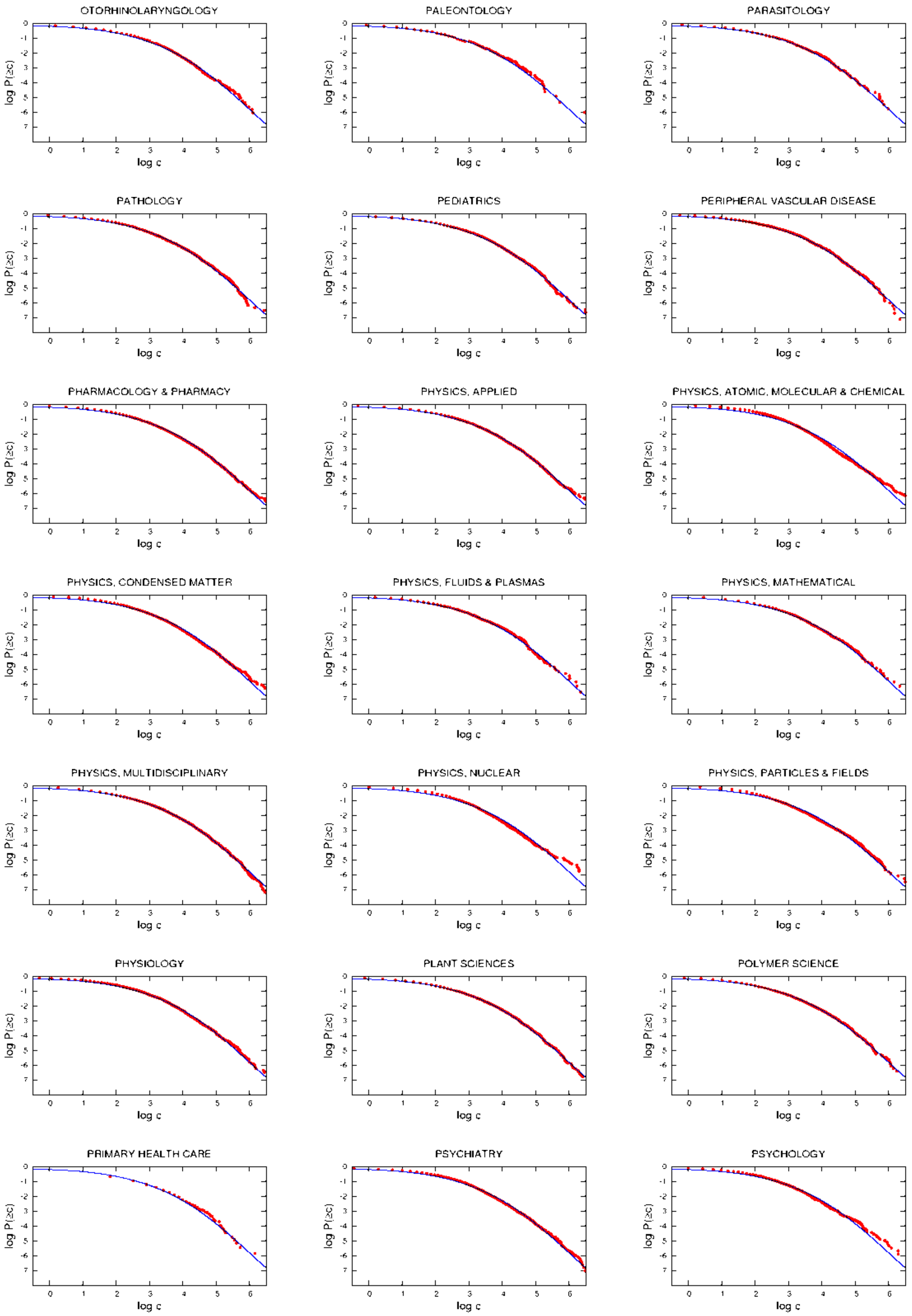

Figure S35: Publication year 1985.

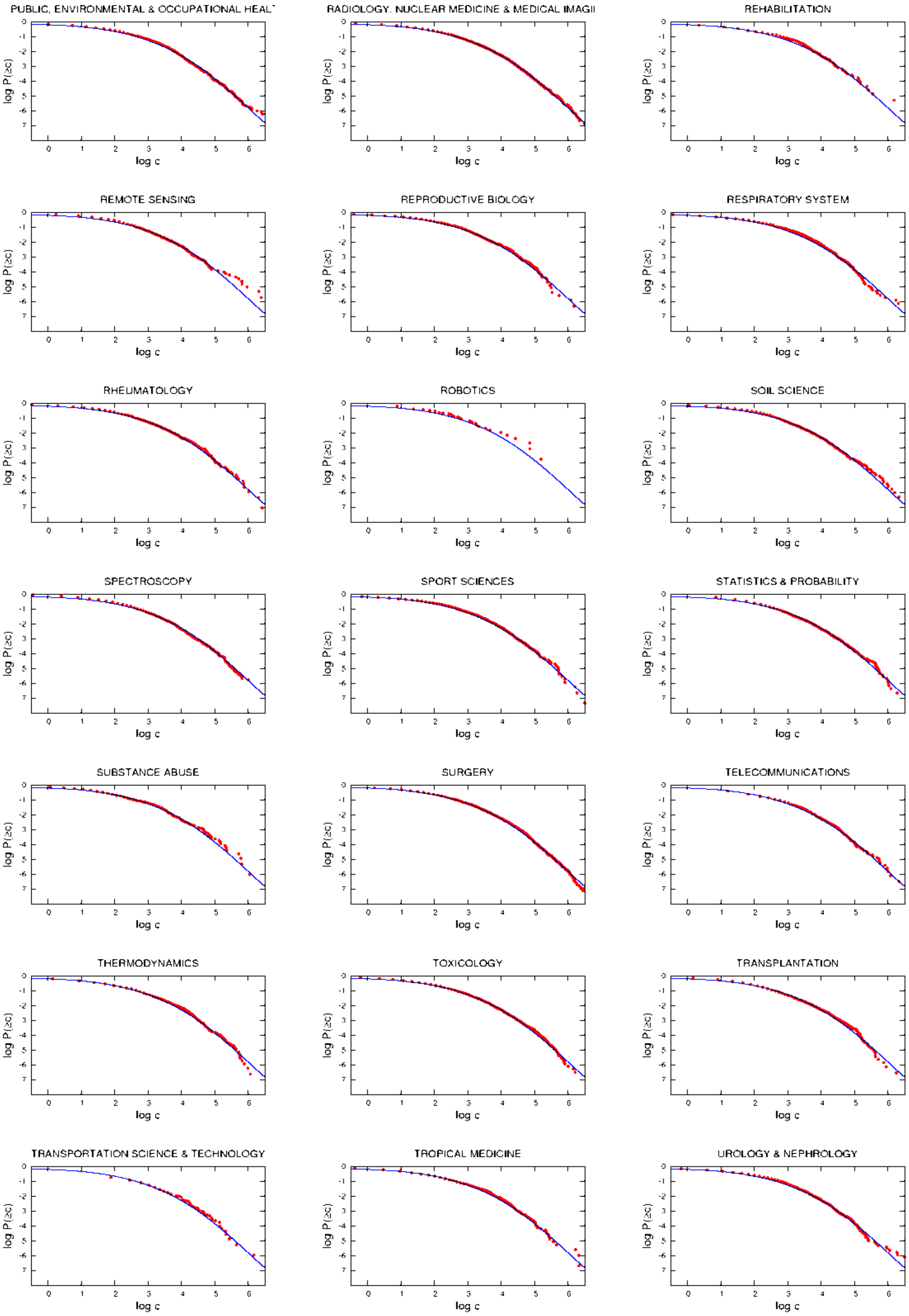

Figure S36: Publication year 1985.

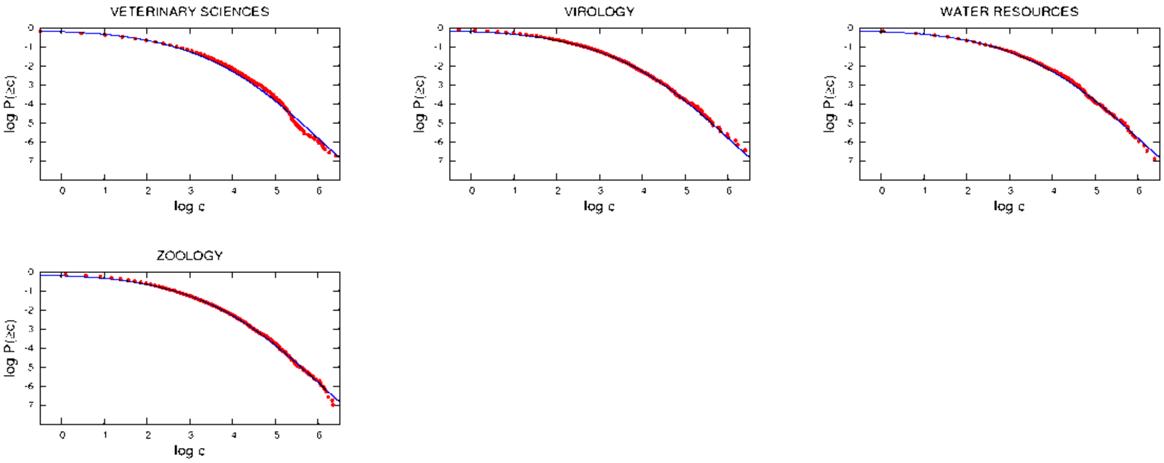

Figure S37: Publication year 1985.

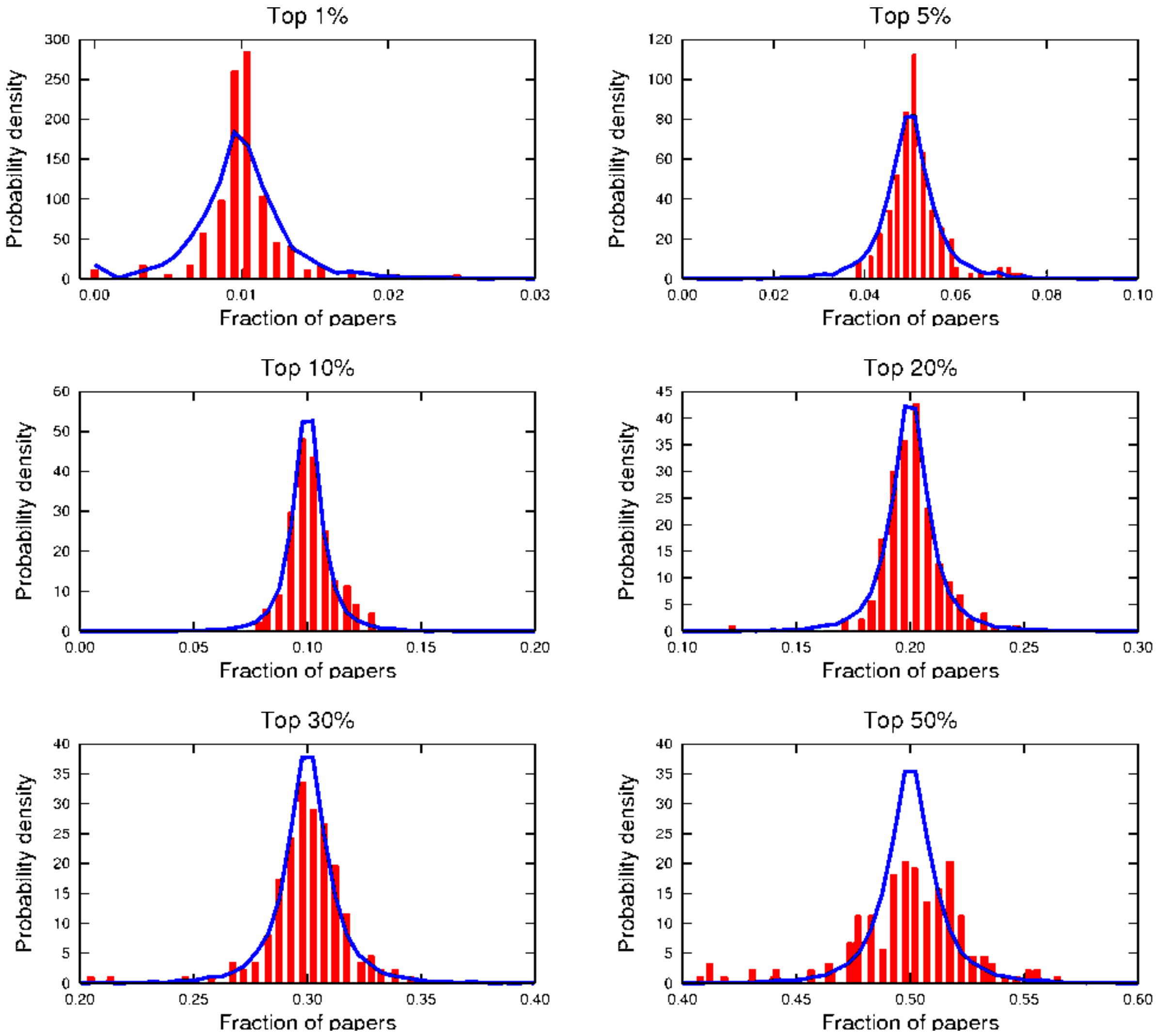

Figure S38: Publication year 1985.
