# Supplementary material for: A Reverse Engineering Approach to the Suppression of Citation Biases Reveals Universal Properties of Citation Distributions
Source: PLoS One. 2012 Mar 29;7(3):e33833. doi: 10.1371/journal.pone.0033833 (PMC3315498; doi:10.1371/journal.pone.0033833)
Supplement: Supporting Information S4 — Complete analysis for publication year . (PDF) [file pone.0033833.s004.pdf]

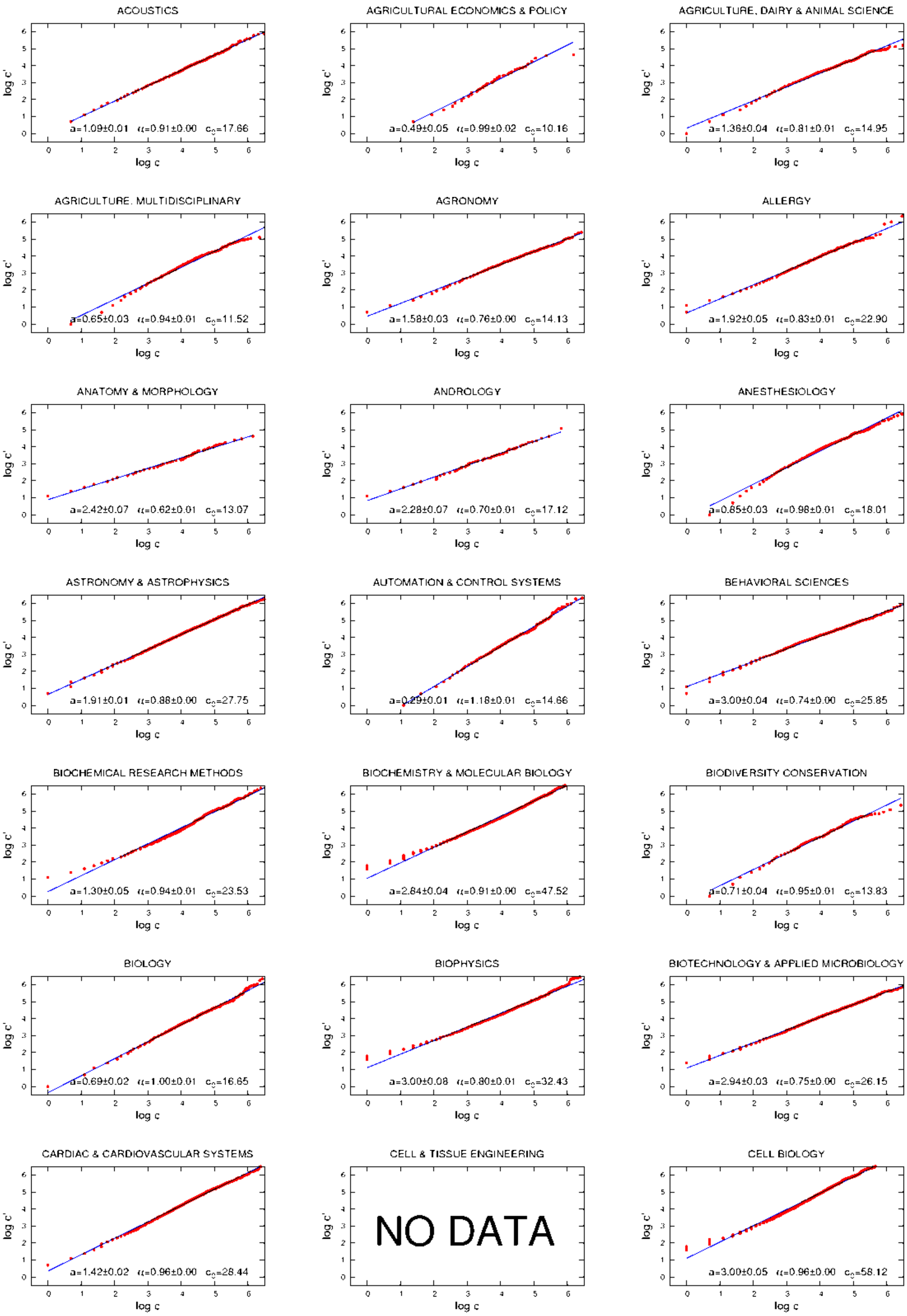

Figure S39: Publication year 1990.

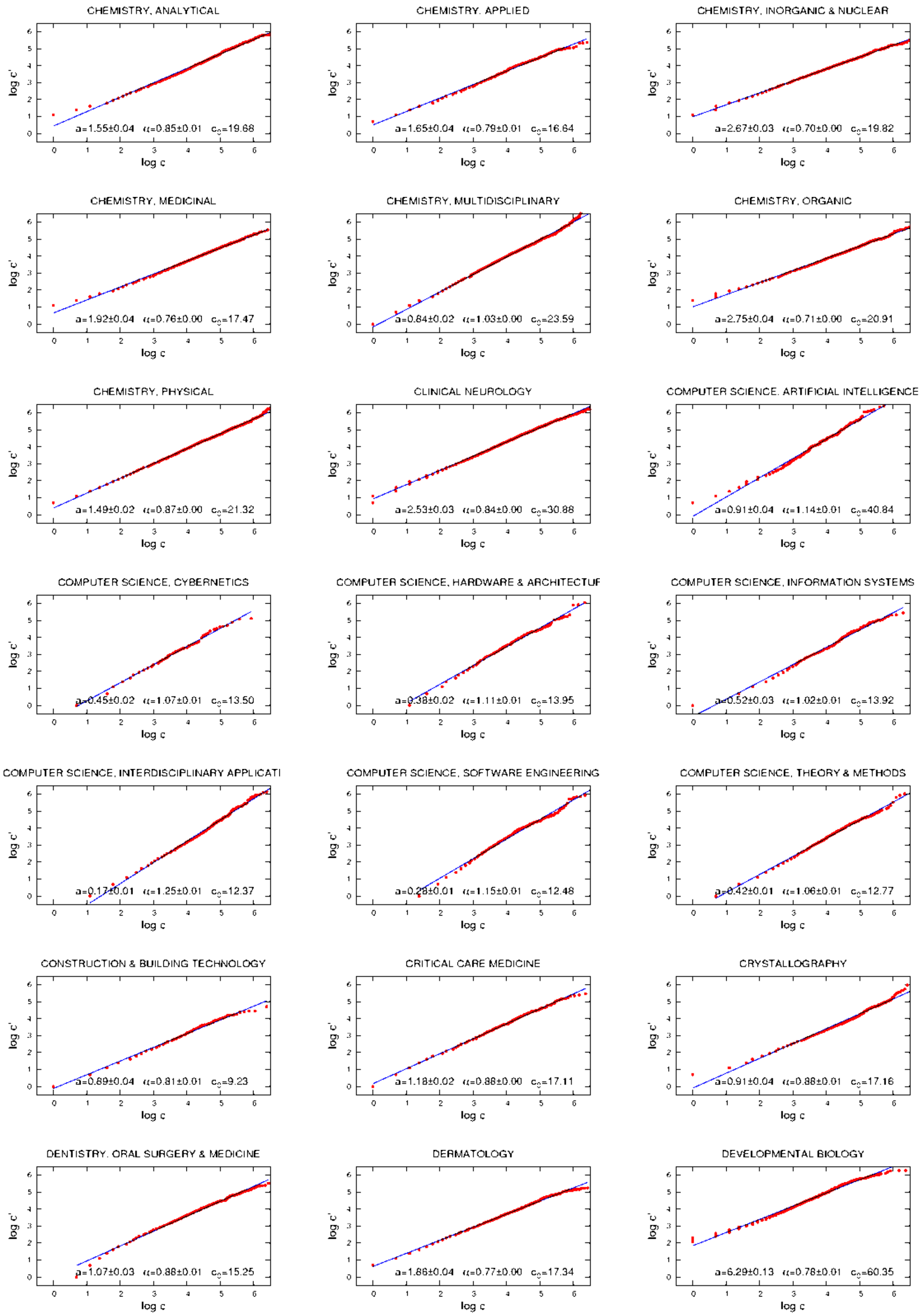

Figure S40: Publication year 1990.

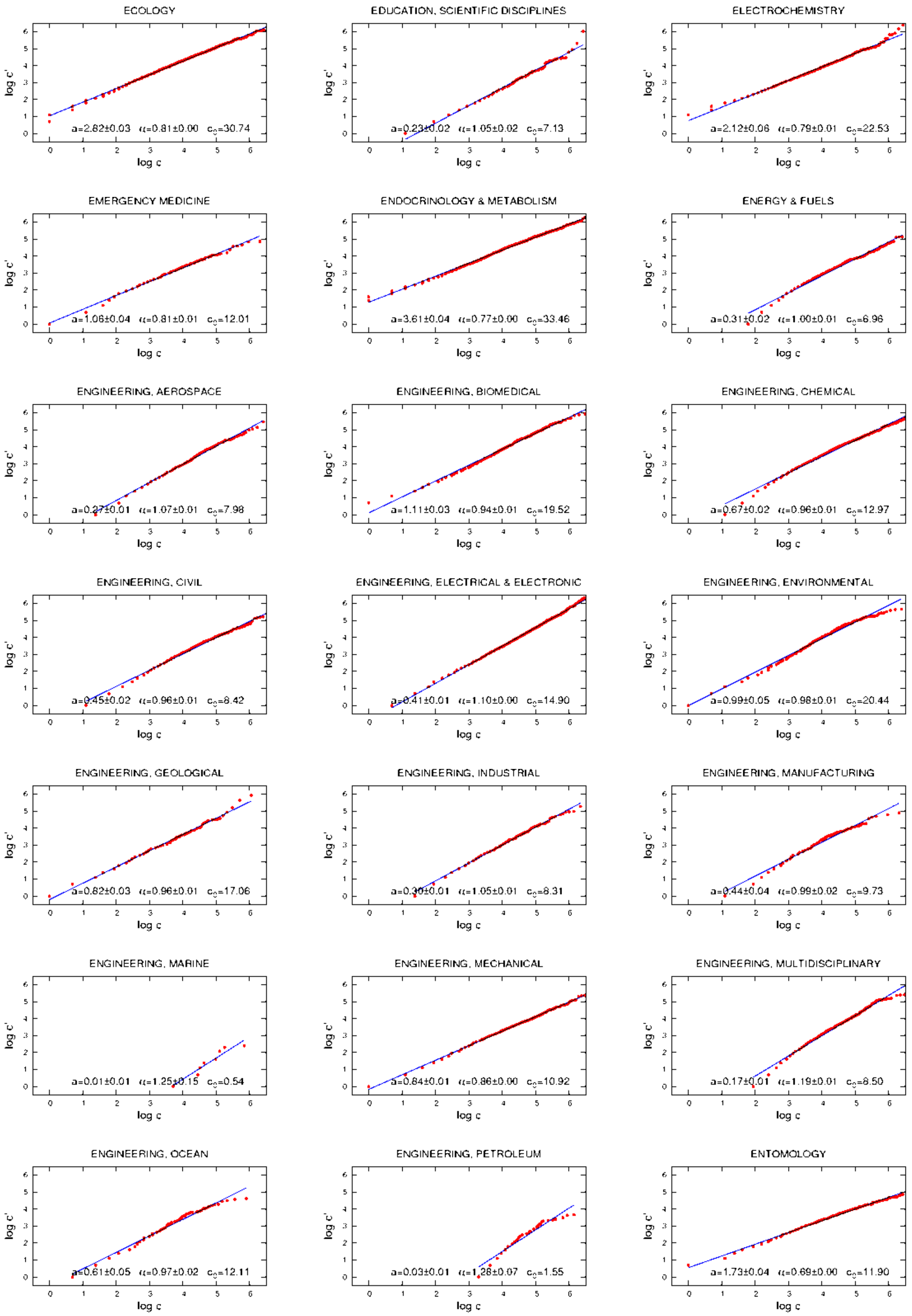

Figure S41: Publication year 1990.

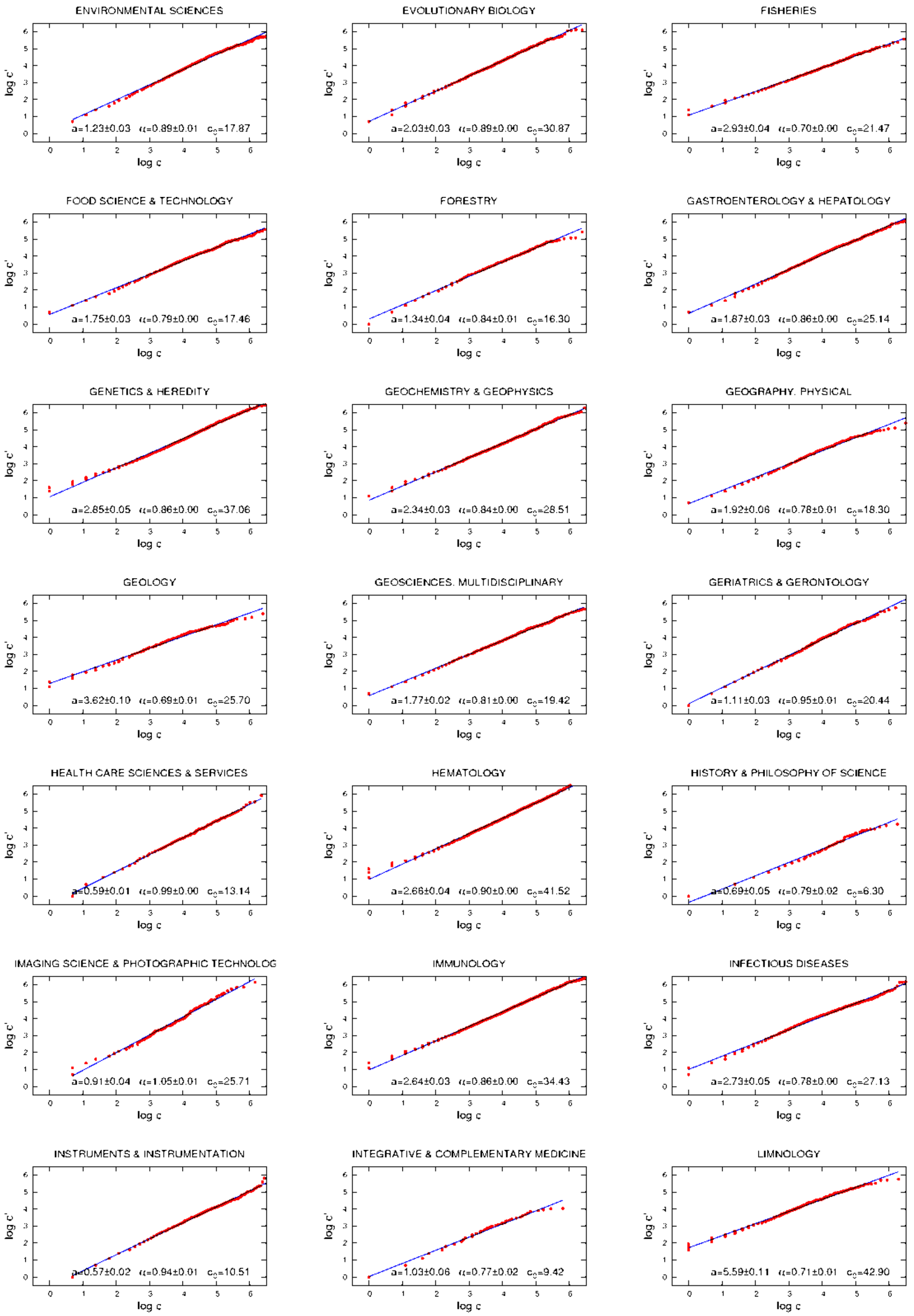

Figure S42: Publication year 1990.

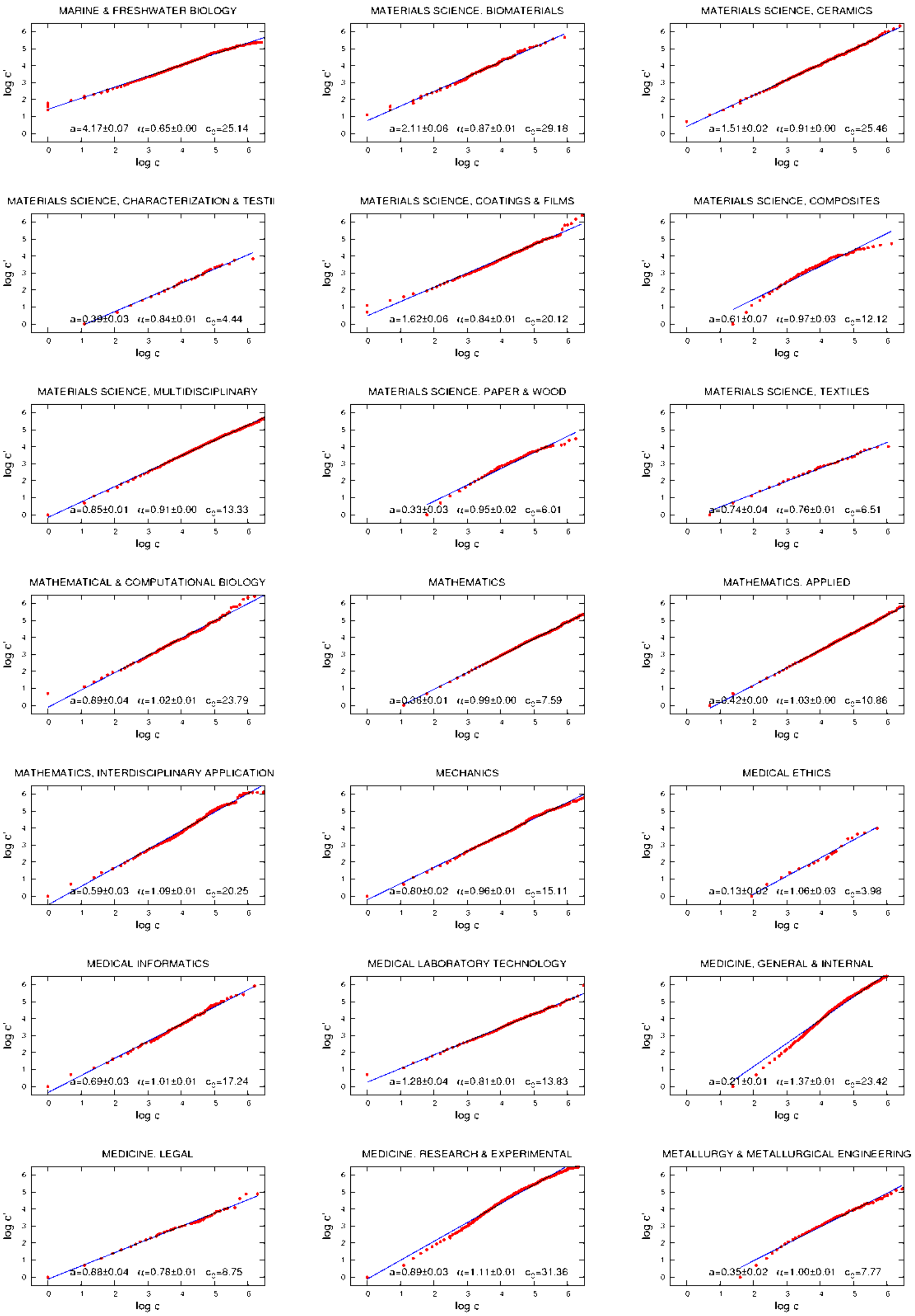

Figure S43: Publication year 1990.

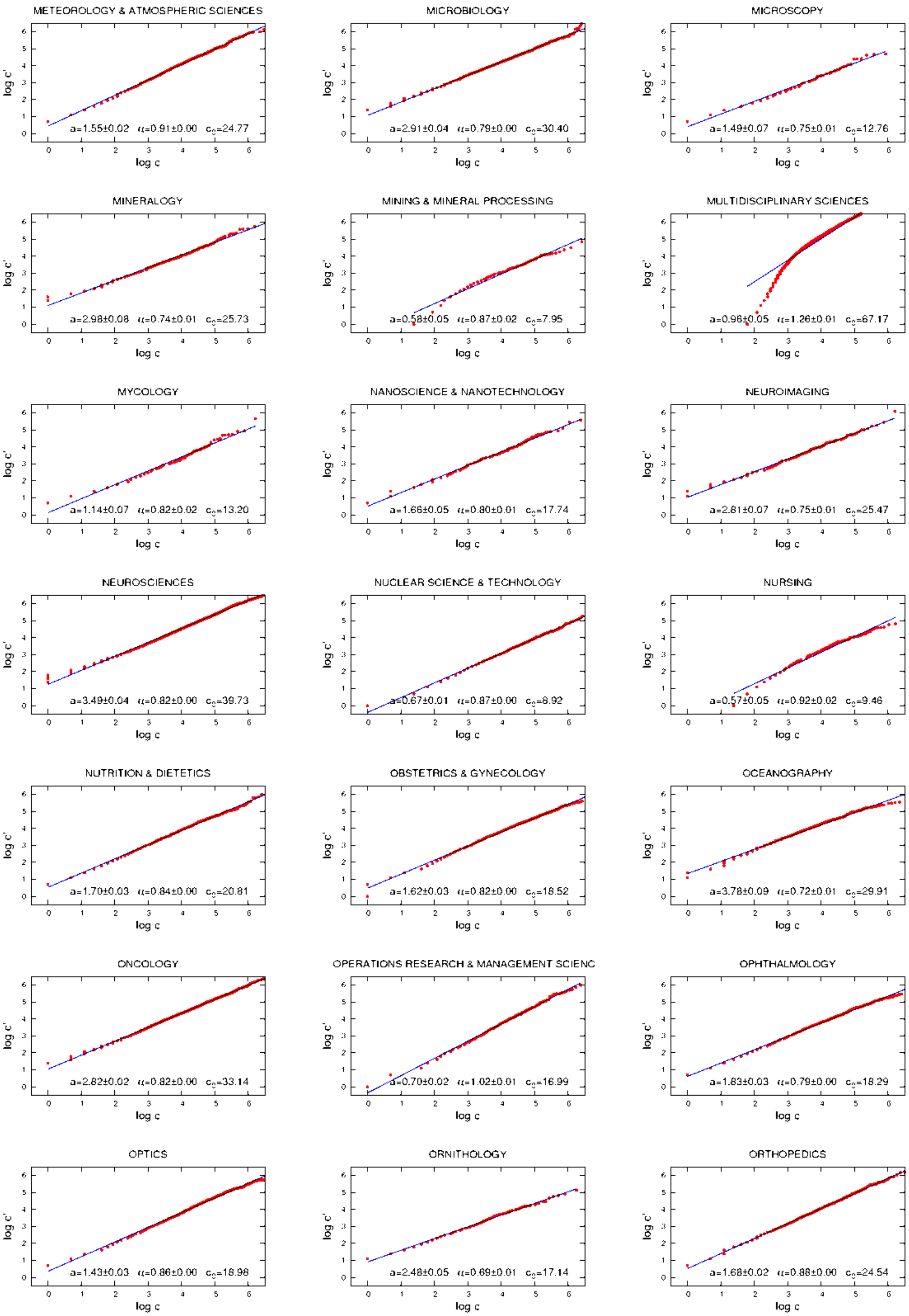

Figure S44: Publication year 1990.

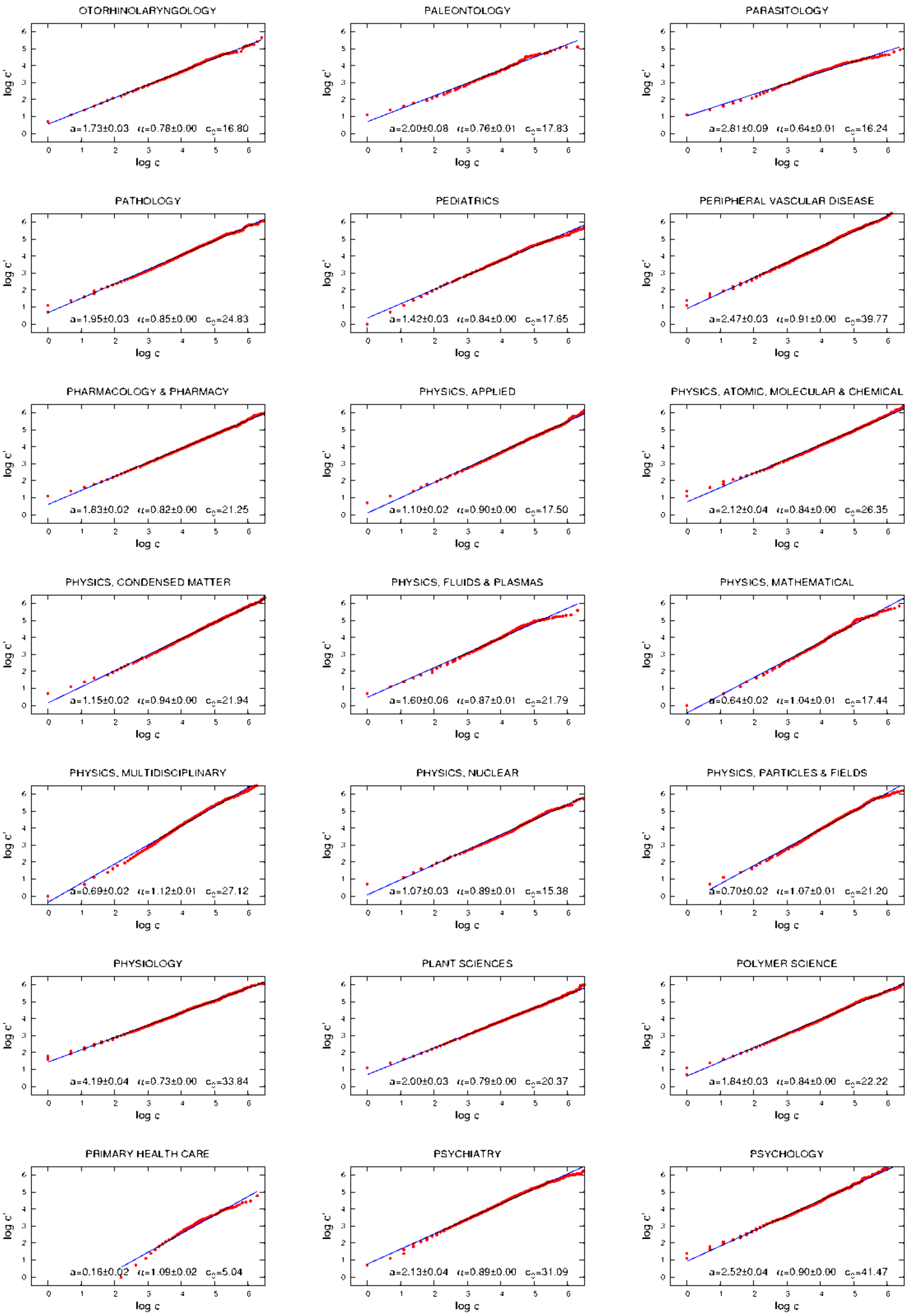

Figure S45: Publication year 1990.

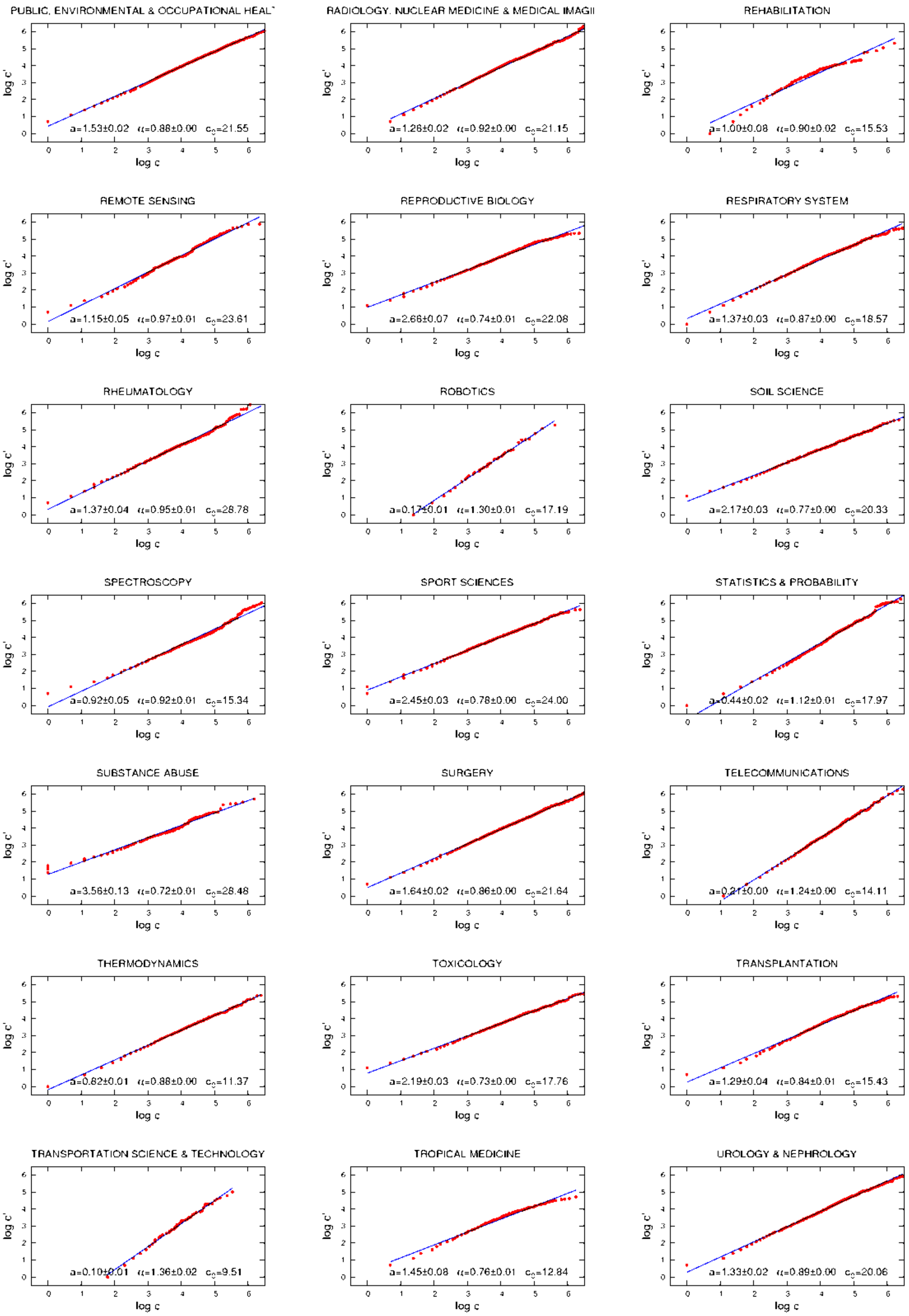

Figure S46: Publication year 1990.

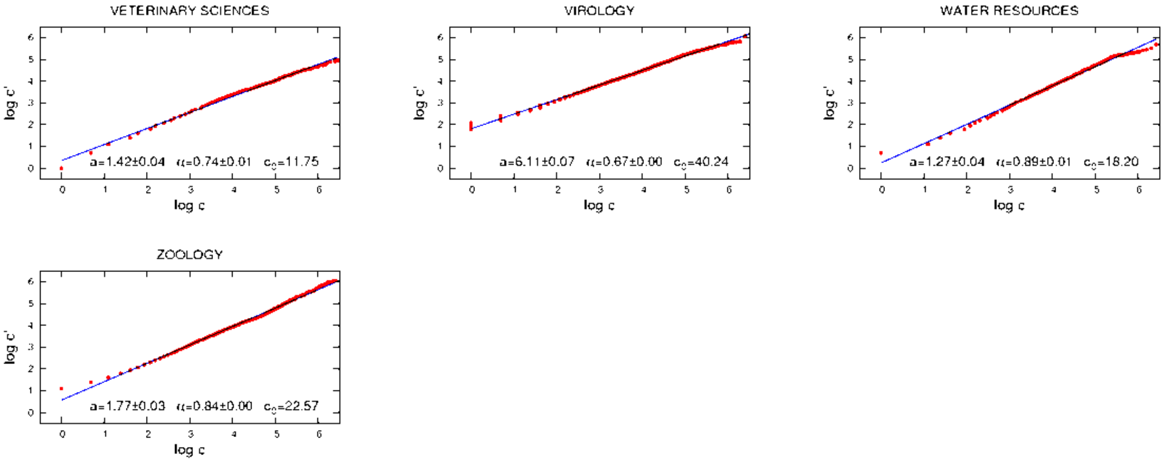

Figure S47: Publication year 1990.

Subject-category	a	α	$\langle c \rangle$	N
ACOUSTICS	1.09 ± 0.01	0.91 ± 0.00	17.66	1,789
AGRICULTURAL ECONOMICS & POLICY	0.49 ± 0.05	0.99 ± 0.02	10.16	460
AGRICULTURE, DAIRY & ANIMAL SCIENCE	1.36 ± 0.04	0.81 ± 0.01	14.95	2,656
AGRICULTURE, MULTIDISCIPLINARY	0.65 ± 0.03	0.94 ± 0.01	11.52	1,918
AGRONOMY	1.58 ± 0.03	0.76 ± 0.00	14.13	3,094
ALLERGY	1.92 ± 0.05	0.83 ± 0.01	22.90	831
ANATOMY & MORPHOLOGY	2.42 ± 0.07	0.62 ± 0.01	13.07	427
ANDROLOGY	2.28 ± 0.07	0.70 ± 0.01	17.12	215
ANESTHESIOLOGY	0.85 ± 0.03	0.98 ± 0.01	18.01	2,459
ASTRONOMY & ASTROPHYSICS	1.91 ± 0.01	0.88 ± 0.00	27.75	6,250
AUTOMATION & CONTROL SYSTEMS	0.29 ± 0.01	1.18 ± 0.01	14.66	1,600
BEHAVIORAL SCIENCES	3.00 ± 0.04	0.74 ± 0.00	25.85	2,977
BIOCHEMICAL RESEARCH METHODS	1.30 ± 0.05	0.94 ± 0.01	23.53	2,820
BIOCHEMISTRY & MOLECULAR BIOLOGY	2.84 ± 0.04	0.91 ± 0.00	47.52	25,535
BIODIVERSITY CONSERVATION	0.71 ± 0.04	0.95 ± 0.01	13.83	780
BIOLOGY	0.69 ± 0.02	1.00 ± 0.01	16.65	3,933
BIOPHYSICS	3.00 ± 0.08	0.80 ± 0.01	32.43	5,508
BIOTECHNOLOGY & APPLIED MICROBIOLOGY	2.94 ± 0.03	0.75 ± 0.00	26.15	4,788
CARDIAC & CARDIOVASCULAR SYSTEMS	1.42 ± 0.02	0.96 ± 0.00	28.44	6,922
CELL & TISSUE ENGINEERING	–	–	–	0
CELL BIOLOGY	3.00 ± 0.05	0.96 ± 0.00	58.12	10,030
CHEMISTRY, ANALYTICAL	1.55 ± 0.04	0.85 ± 0.01	19.68	6,050
CHEMISTRY, APPLIED	1.65 ± 0.04	0.79 ± 0.01	16.64	2,267
CHEMISTRY, INORGANIC & NUCLEAR	2.67 ± 0.03	0.70 ± 0.00	19.82	5,132
CHEMISTRY, MEDICINAL	1.92 ± 0.04	0.76 ± 0.00	17.47	3,137
CHEMISTRY, MULTIDISCIPLINARY	0.84 ± 0.02	1.03 ± 0.00	23.59	11,327
CHEMISTRY, ORGANIC	2.75 ± 0.04	0.71 ± 0.00	20.91	8,867
CHEMISTRY, PHYSICAL	1.49 ± 0.02	0.87 ± 0.00	21.32	11,078
CLINICAL NEUROLOGY	2.53 ± 0.03	0.84 ± 0.00	30.88	7,212
COMPUTER SCIENCE, ARTIFICIAL INTELLIGENCE	0.91 ± 0.04	1.14 ± 0.01	40.84	715
COMPUTER SCIENCE, CYBERNETICS	0.45 ± 0.02	1.07 ± 0.01	13.50	276
COMPUTER SCIENCE, HARDWARE & ARCHITECTURE	0.38 ± 0.02	1.11 ± 0.01	13.95	1,265
COMPUTER SCIENCE, INFORMATION SYSTEMS	0.52 ± 0.03	1.02 ± 0.01	13.92	1,159
COMPUTER SCIENCE, INTERDISCIPLINARY APPLICATIONS	0.17 ± 0.01	1.25 ± 0.01	12.37	2,886
COMPUTER SCIENCE, SOFTWARE ENGINEERING	0.28 ± 0.01	1.15 ± 0.01	12.48	1,947
COMPUTER SCIENCE, THEORY & METHODS	0.42 ± 0.01	1.06 ± 0.01	12.77	1,901
CONSTRUCTION & BUILDING TECHNOLOGY	0.89 ± 0.04	0.81 ± 0.01	9.23	708
CRITICAL CARE MEDICINE	1.18 ± 0.02	0.88 ± 0.00	17.11	1,346
CRYSTALLOGRAPHY	0.91 ± 0.04	0.88 ± 0.01	17.16	3,867
DENTISTRY, ORAL SURGERY & MEDICINE	1.07 ± 0.03	0.88 ± 0.01	15.25	3,248
DERMATOLOGY	1.86 ± 0.04	0.77 ± 0.00	17.34	3,136
DEVELOPMENTAL BIOLOGY	6.29 ± 0.13	0.78 ± 0.01	60.35	1,355
ECOLOGY	2.82 ± 0.03	0.81 ± 0.00	30.74	4,718
EDUCATION, SCIENTIFIC DISCIPLINES	0.23 ± 0.02	1.05 ± 0.02	7.13	1,543
ELECTROCHEMISTRY	2.12 ± 0.06	0.79 ± 0.01	22.53	2,351
EMERGENCY MEDICINE	1.06 ± 0.04	0.81 ± 0.01	12.02	602
ENDOCRINOLOGY & METABOLISM	3.61 ± 0.04	0.77 ± 0.00	33.46	6,836
ENERGY & FUELS	0.31 ± 0.02	1.00 ± 0.01	6.96	3,018
ENGINEERING, AEROSPACE	0.27 ± 0.01	1.07 ± 0.01	7.98	1,522
ENGINEERING, BIOMEDICAL	1.11 ± 0.03	0.94 ± 0.01	19.52	1,759

Table S21: Publication year 1990.

Subject-category	a	α	$\langle c \rangle$	N
ENGINEERING, CHEMICAL	0.67 ± 0.02	0.96 ± 0.01	12.97	6,807
ENGINEERING, CIVIL	0.45 ± 0.02	0.96 ± 0.01	8.42	2,998
ENGINEERING, ELECTRICAL & ELECTRONIC	0.41 ± 0.01	1.10 ± 0.00	14.90	12,081
ENGINEERING, ENVIRONMENTAL	0.99 ± 0.05	0.98 ± 0.01	20.44	1,409
ENGINEERING, GEOLOGICAL	0.82 ± 0.03	0.96 ± 0.01	17.06	347
ENGINEERING, INDUSTRIAL	0.30 ± 0.01	1.05 ± 0.01	8.31	1,274
ENGINEERING, MANUFACTURING	0.44 ± 0.04	0.99 ± 0.02	9.73	599
ENGINEERING, MARINE	0.01 ± 0.01	1.25 ± 0.15	0.54	228
ENGINEERING, MECHANICAL	0.84 ± 0.01	0.86 ± 0.00	10.92	3,390
ENGINEERING, MULTIDISCIPLINARY	0.17 ± 0.01	1.19 ± 0.01	8.50	2,603
ENGINEERING, OCEAN	0.61 ± 0.05	0.97 ± 0.02	12.11	263
ENGINEERING, PETROLEUM	0.03 ± 0.01	1.28 ± 0.07	1.55	854
ENTOMOLOGY	1.73 ± 0.04	0.69 ± 0.00	11.90	3,128
ENVIRONMENTAL SCIENCES	1.23 ± 0.03	0.89 ± 0.01	17.87	6,779
EVOLUTIONARY BIOLOGY	2.03 ± 0.03	0.89 ± 0.00	30.87	1,419
FISHERIES	2.93 ± 0.04	0.70 ± 0.00	21.47	1,599
FOOD SCIENCE & TECHNOLOGY	1.75 ± 0.03	0.79 ± 0.00	17.46	4,094
FORESTRY	1.34 ± 0.04	0.84 ± 0.01	16.30	1,388
GASTROENTEROLOGY & HEPATOLOGY	1.87 ± 0.03	0.86 ± 0.00	25.14	4,356
GENETICS & HEREDITY	2.85 ± 0.05	0.86 ± 0.00	37.06	5,260
GEOCHEMISTRY & GEOPHYSICS	2.34 ± 0.03	0.84 ± 0.00	28.51	3,314
GEOGRAPHY, PHYSICAL	1.92 ± 0.06	0.78 ± 0.01	18.30	911
GEOLOGY	3.62 ± 0.10	0.69 ± 0.01	25.70	729
GEOSCIENCES, MULTIDISCIPLINARY	1.77 ± 0.02	0.81 ± 0.00	19.42	4,037
GERIATRICS & GERONTOLOGY	1.11 ± 0.03	0.95 ± 0.01	20.44	948
HEALTH CARE SCIENCES & SERVICES	0.59 ± 0.01	0.99 ± 0.00	13.14	1,301
HEMATOLOGY	2.66 ± 0.04	0.90 ± 0.00	41.52	4,716
HISTORY & PHILOSOPHY OF SCIENCE	0.69 ± 0.05	0.79 ± 0.02	6.30	533
IMAGING SCIENCE & PHOTOGRAPHIC TECHNOLOGY	0.91 ± 0.04	1.05 ± 0.01	25.71	438
IMMUNOLOGY	2.64 ± 0.03	0.86 ± 0.00	34.43	11,079
INFECTIOUS DISEASES	2.73 ± 0.05	0.78 ± 0.00	27.13	3,629
INSTRUMENTS & INSTRUMENTATION	0.57 ± 0.02	0.94 ± 0.01	10.51	5,576
INTEGRATIVE & COMPLEMENTARY MEDICINE	1.03 ± 0.06	0.77 ± 0.02	9.42	213
LIMNOLOGY	5.59 ± 0.11	0.71 ± 0.01	42.90	563
MARINE & FRESHWATER BIOLOGY	4.17 ± 0.07	0.65 ± 0.00	25.14	3,728
MATERIALS SCIENCE, BIOMATERIALS	2.11 ± 0.06	0.87 ± 0.01	29.18	267
MATERIALS SCIENCE, CERAMICS	1.51 ± 0.02	0.91 ± 0.00	25.46	1,420
MATERIALS SCIENCE, CHARACTERIZATION & TESTING	0.39 ± 0.03	0.84 ± 0.01	4.44	429
MATERIALS SCIENCE, COATINGS & FILMS	1.62 ± 0.06	0.84 ± 0.01	20.12	1,605
MATERIALS SCIENCE, COMPOSITES	0.61 ± 0.07	0.97 ± 0.03	12.12	419
MATERIALS SCIENCE, MULTIDISCIPLINARY	0.85 ± 0.01	0.91 ± 0.00	13.33	11,074
MATERIALS SCIENCE, PAPER & WOOD	0.33 ± 0.03	0.95 ± 0.02	6.01	1,071
MATERIALS SCIENCE, TEXTILES	0.74 ± 0.04	0.76 ± 0.01	6.51	348
MATHEMATICAL & COMPUTATIONAL BIOLOGY	0.89 ± 0.04	1.02 ± 0.01	23.79	954
MATHEMATICS	0.36 ± 0.01	0.99 ± 0.00	7.59	9,160
MATHEMATICS, APPLIED	0.42 ± 0.00	1.03 ± 0.00	10.86	5,492
MATHEMATICS, INTERDISCIPLINARY APPLICATIONS	0.59 ± 0.03	1.09 ± 0.01	20.25	1,688
MECHANICS	0.80 ± 0.02	0.96 ± 0.01	15.11	4,460
MEDICAL ETHICS	0.13 ± 0.02	1.06 ± 0.03	3.98	175
MEDICAL INFORMATICS	0.69 ± 0.03	1.01 ± 0.01	17.24	474

Table S22: Publication year 1990.

Subject-category	a	α	$\langle c \rangle$	N
MEDICAL LABORATORY TECHNOLOGY	1.28 ± 0.04	0.81 ± 0.01	13.83	1,786
MEDICINE, GENERAL & INTERNAL	0.21 ± 0.01	1.37 ± 0.01	23.42	15,069
MEDICINE, LEGAL	0.88 ± 0.04	0.78 ± 0.01	8.75	573
MEDICINE, RESEARCH & EXPERIMENTAL	0.89 ± 0.03	1.11 ± 0.01	31.36	5,578
METALLURGY & METALLURGICAL ENGINEERING	0.35 ± 0.02	1.00 ± 0.01	7.77	1,652
METEOROLOGY & ATMOSPHERIC SCIENCES	1.55 ± 0.02	0.91 ± 0.00	24.77	2,618
MICROBIOLOGY	2.91 ± 0.04	0.79 ± 0.00	30.40	7,428
MICROSCOPY	1.49 ± 0.07	0.75 ± 0.01	12.76	275
MINERALOGY	2.98 ± 0.08	0.74 ± 0.01	25.73	924
MINING & MINERAL PROCESSING	0.58 ± 0.05	0.87 ± 0.02	7.95	751
MULTIDISCIPLINARY SCIENCES	0.96 ± 0.05	1.26 ± 0.01	67.17	11,404
MYCOLOGY	1.14 ± 0.07	0.82 ± 0.02	13.20	500
NANOSCIENCE & NANOTECHNOLOGY	1.66 ± 0.05	0.80 ± 0.01	17.74	689
NEUROIMAGING	2.81 ± 0.07	0.75 ± 0.01	25.47	487
NEUROSCIENCES	3.49 ± 0.04	0.82 ± 0.00	39.73	13,815
NUCLEAR SCIENCE & TECHNOLOGY	0.67 ± 0.01	0.87 ± 0.00	8.92	5,555
NURSING	0.57 ± 0.05	0.92 ± 0.02	9.46	1,007
NUTRITION & DIETETICS	1.70 ± 0.03	0.84 ± 0.00	20.81	2,929
OBSTETRICS & GYNECOLOGY	1.62 ± 0.03	0.82 ± 0.00	18.52	4,618
OCEANOGRAPHY	3.78 ± 0.09	0.72 ± 0.01	29.91	1,969
ONCOLOGY	2.82 ± 0.02	0.82 ± 0.00	33.14	9,443
OPERATIONS RESEARCH & MANAGEMENT SCIENCE	0.70 ± 0.02	1.02 ± 0.01	16.99	2,047
OPHTHALMOLOGY	1.83 ± 0.03	0.79 ± 0.00	18.29	3,787
OPTICS	1.43 ± 0.03	0.86 ± 0.00	18.98	6,966
ORNITHOLOGY	2.48 ± 0.05	0.69 ± 0.01	17.14	526
ORTHOPEDICS	1.68 ± 0.02	0.88 ± 0.00	24.54	2,732
OTORHINOLARYNGOLOGY	1.73 ± 0.03	0.78 ± 0.00	16.80	2,259
PALEONTOLOGY	2.00 ± 0.08	0.76 ± 0.01	17.83	589
PARASITOLOGY	2.81 ± 0.09	0.64 ± 0.01	16.24	1,417
PATHOLOGY	1.95 ± 0.03	0.85 ± 0.00	24.83	4,187
PEDIATRICS	1.42 ± 0.03	0.84 ± 0.00	17.65	5,337
PERIPHERAL VASCULAR DISEASE	2.47 ± 0.03	0.91 ± 0.00	39.77	3,608
PHARMACOLOGY & PHARMACY	1.83 ± 0.02	0.82 ± 0.00	21.25	15,634
PHYSICS, APPLIED	1.10 ± 0.02	0.90 ± 0.00	17.50	12,359
PHYSICS, ATOMIC, MOLECULAR & CHEMICAL	2.12 ± 0.04	0.84 ± 0.00	26.35	8,269
PHYSICS, CONDENSED MATTER	1.15 ± 0.02	0.94 ± 0.00	21.94	10,973
PHYSICS, FLUIDS & PLASMAS	1.60 ± 0.06	0.87 ± 0.01	21.79	1,175
PHYSICS, MATHEMATICAL	0.64 ± 0.02	1.04 ± 0.01	17.44	1,994
PHYSICS, MULTIDISCIPLINARY	0.69 ± 0.02	1.12 ± 0.01	27.12	9,178
PHYSICS, NUCLEAR	1.07 ± 0.03	0.89 ± 0.01	15.38	3,315
PHYSICS, PARTICLES & FIELDS	0.70 ± 0.02	1.07 ± 0.01	21.20	4,037
PHYSIOLOGY	4.19 ± 0.04	0.73 ± 0.00	33.84	5,969
PLANT SCIENCES	2.00 ± 0.03	0.79 ± 0.00	20.37	10,016
POLYMER SCIENCE	1.84 ± 0.03	0.84 ± 0.00	22.22	4,380
PRIMARY HEALTH CARE	0.16 ± 0.02	1.09 ± 0.02	5.04	1,113
PSYCHIATRY	2.13 ± 0.04	0.89 ± 0.00	31.09	5,029
PSYCHOLOGY	2.52 ± 0.04	0.90 ± 0.00	41.47	1,600
PUBLIC, ENVIRONMENTAL & OCCUPATIONAL HEALTH	1.53 ± 0.02	0.88 ± 0.00	21.55	5,378
RADIOLOGY, NUCLEAR MEDICINE & MEDICAL IMAGING	1.26 ± 0.02	0.92 ± 0.00	21.15	7,596
REHABILITATION	1.00 ± 0.08	0.90 ± 0.02	15.53	498

Table S23: Publication year 1990.

Subject-category	a	α	$\langle c \rangle$	N
REMOTE SENSING	1.15 ± 0.05	0.97 ± 0.01	23.61	649
REPRODUCTIVE BIOLOGY	2.66 ± 0.07	0.74 ± 0.01	22.08	1,917
RESPIRATORY SYSTEM	1.37 ± 0.03	0.87 ± 0.00	18.57	3,254
RHEUMATOLOGY	1.37 ± 0.04	0.95 ± 0.01	28.78	1,462
ROBOTICS	0.17 ± 0.01	1.30 ± 0.01	17.19	149
SOIL SCIENCE	2.17 ± 0.03	0.77 ± 0.00	20.33	1,939
SPECTROSCOPY	0.92 ± 0.05	0.92 ± 0.01	15.34	3,760
SPORT SCIENCES	2.45 ± 0.03	0.78 ± 0.00	24.00	2,036
STATISTICS & PROBABILITY	0.44 ± 0.02	1.12 ± 0.01	17.96	2,916
SUBSTANCE ABUSE	3.56 ± 0.13	0.72 ± 0.01	28.48	454
SURGERY	1.64 ± 0.02	0.86 ± 0.00	21.64	12,412
TELECOMMUNICATIONS	0.21 ± 0.00	1.24 ± 0.00	14.11	1,739
THERMODYNAMICS	0.82 ± 0.01	0.88 ± 0.00	11.37	2,175
TOXICOLOGY	2.19 ± 0.03	0.73 ± 0.00	17.76	3,532
TRANSPLANTATION	1.29 ± 0.04	0.84 ± 0.01	15.43	2,401
TRANSPORTATION SCIENCE & TECHNOLOGY	0.10 ± 0.01	1.36 ± 0.02	9.51	379
TROPICAL MEDICINE	1.45 ± 0.08	0.76 ± 0.01	12.84	1,072
UROLOGY & NEPHROLOGY	1.33 ± 0.02	0.89 ± 0.00	20.06	3,403
VETERINARY SCIENCES	1.42 ± 0.04	0.74 ± 0.01	11.75	5,079
VIROLOGY	6.11 ± 0.07	0.67 ± 0.00	40.24	2,836
WATER RESOURCES	1.27 ± 0.04	0.89 ± 0.01	18.20	2,366
ZOOLOGY	1.77 ± 0.03	0.84 ± 0.00	22.57	4,615
**TOTAL**	**1**.00 ± 0.00	**1**.00 ± 0.00	**2**4.16	**6**19,430

Table S24: Publication year 1990.

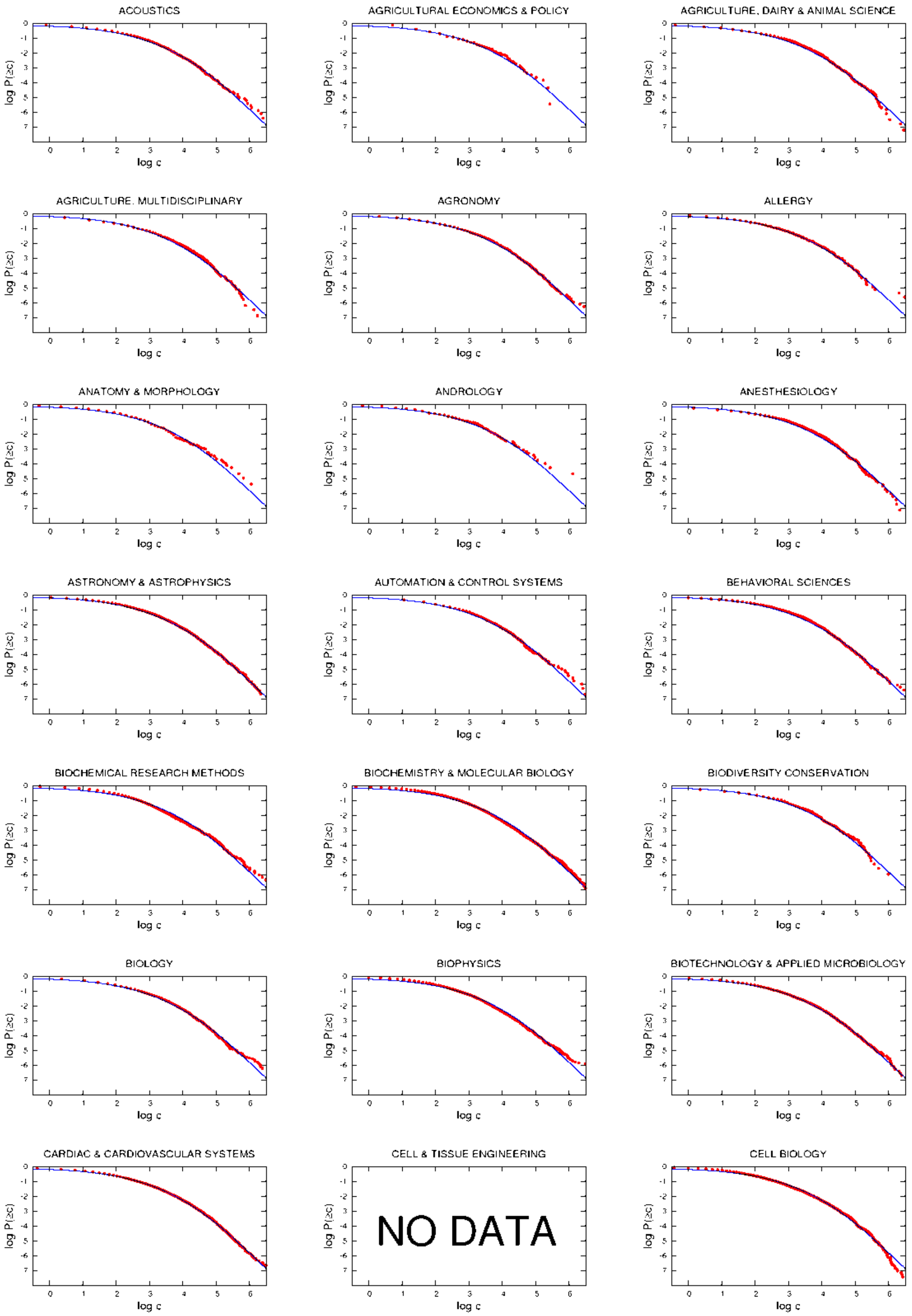

Figure S48: Publication year 1990.

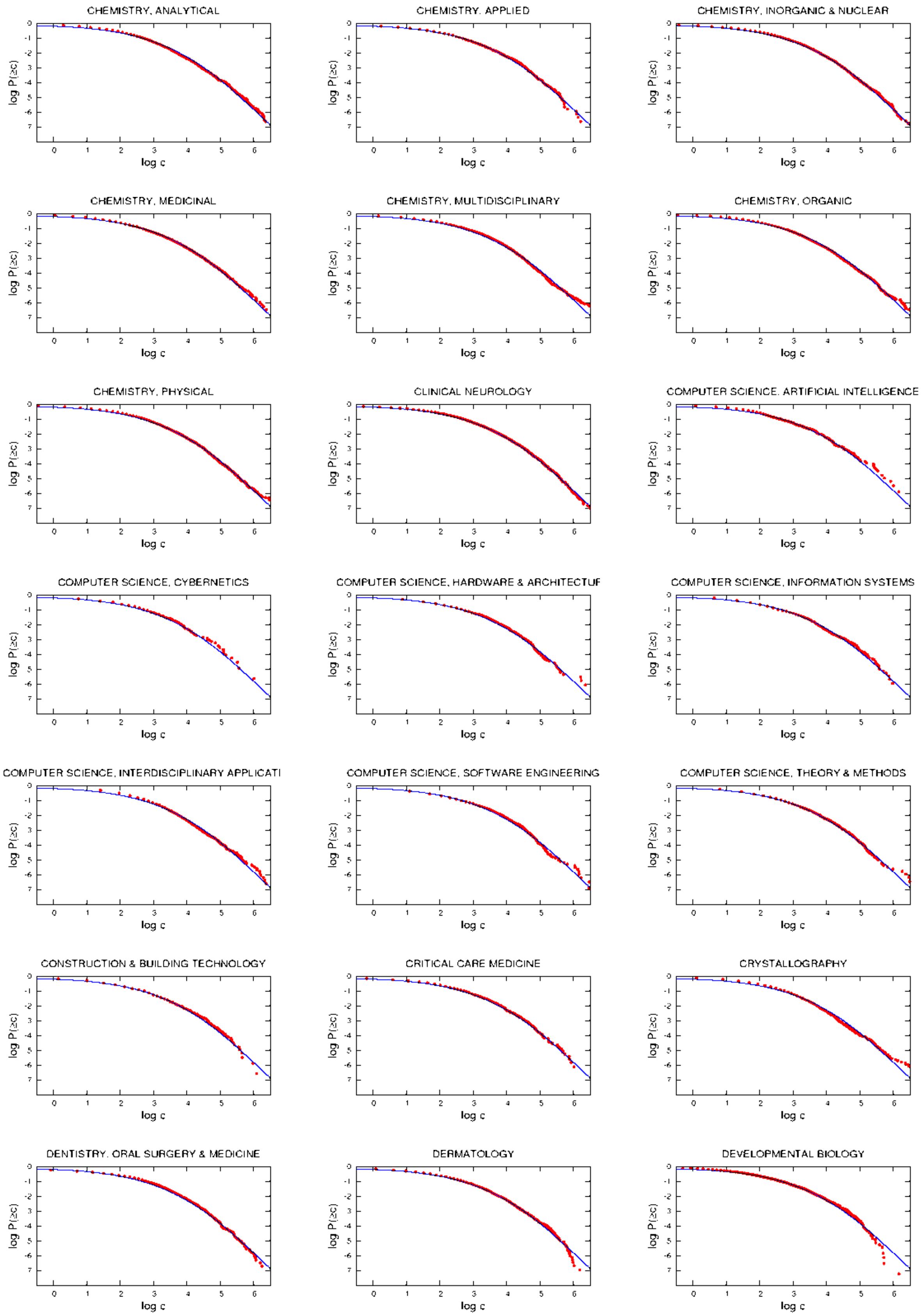

Figure S49: Publication year 1990.

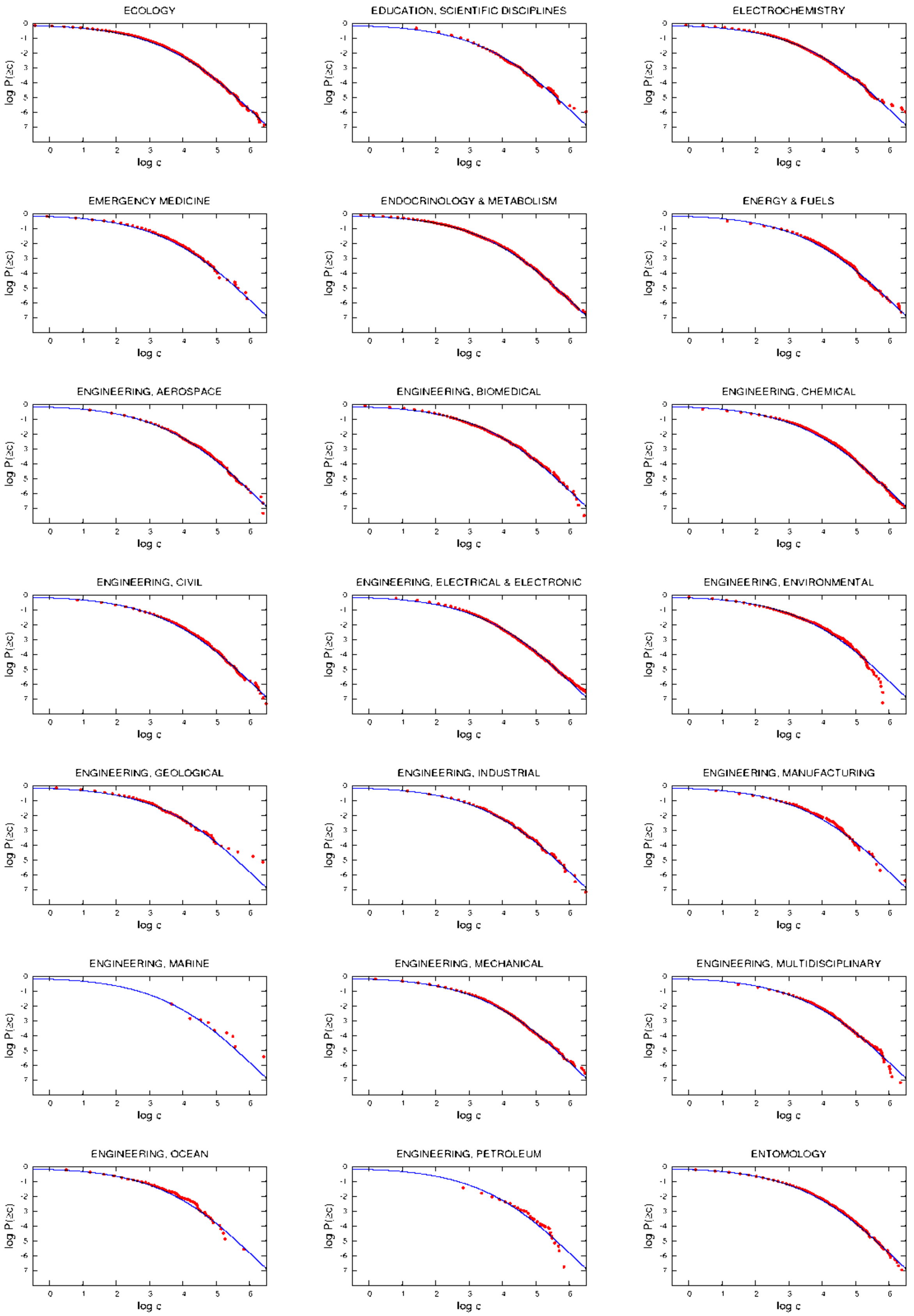

Figure S50: Publication year 1990.

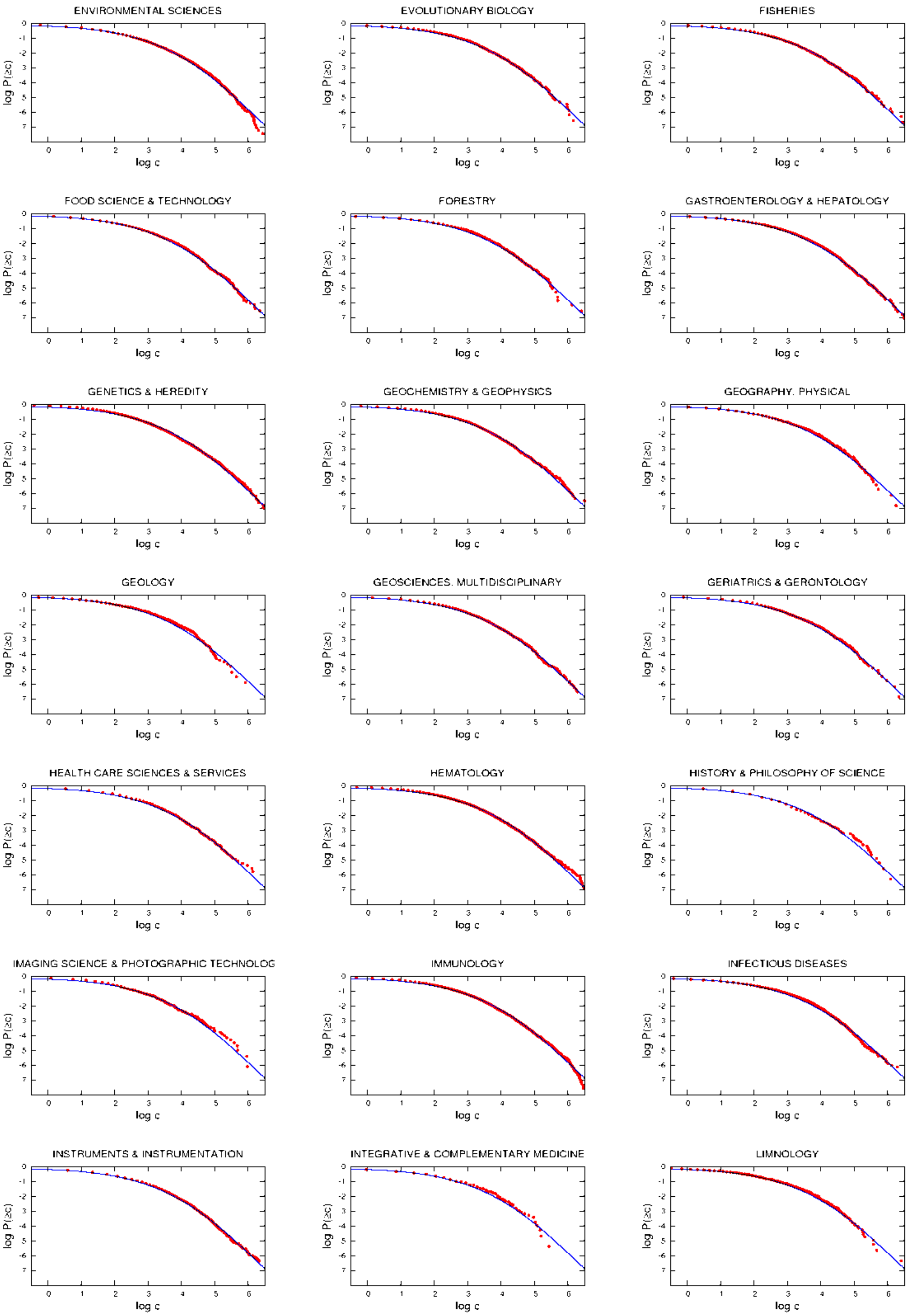

Figure S51: Publication year 1990.

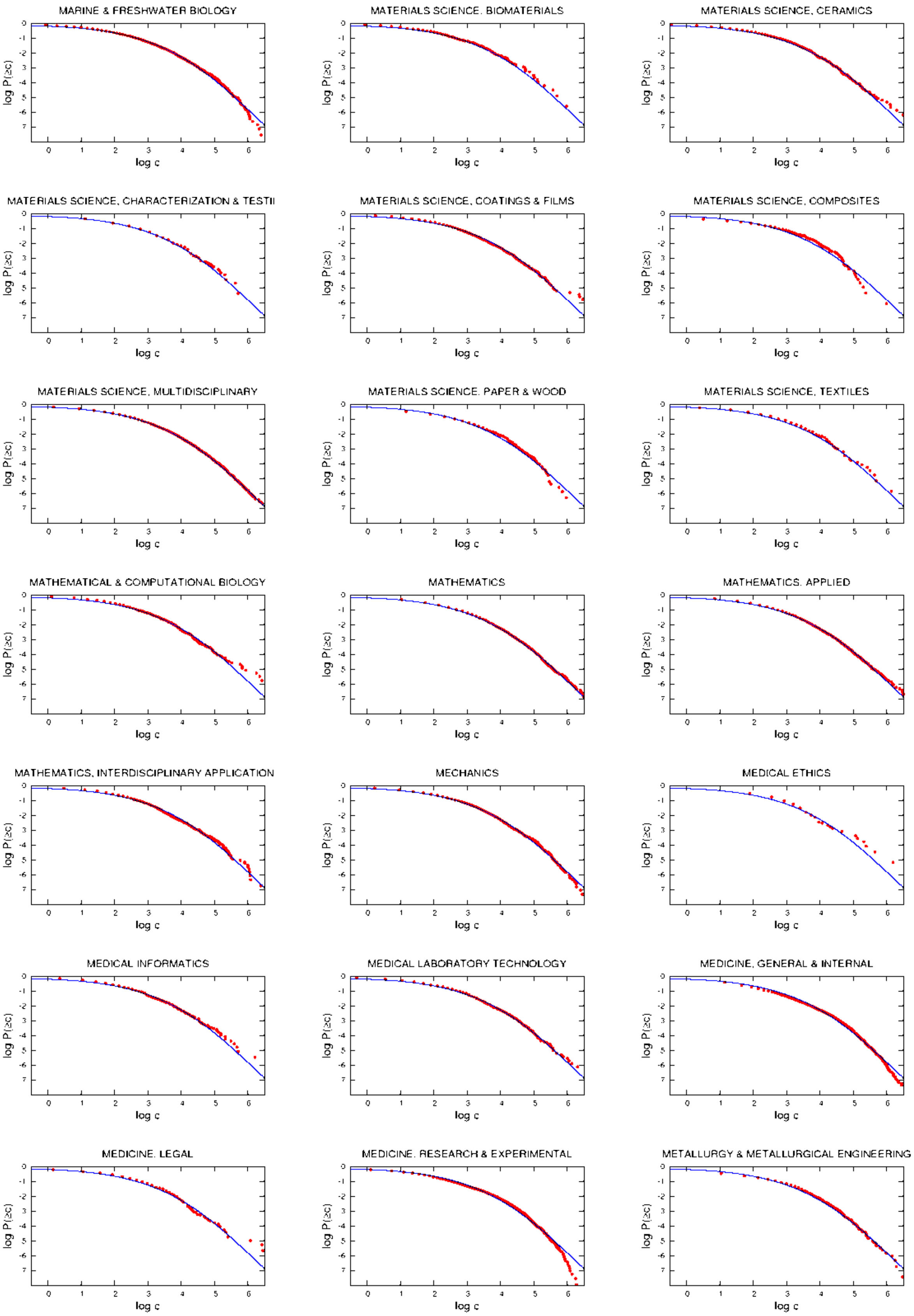

Figure S52: Publication year 1990.

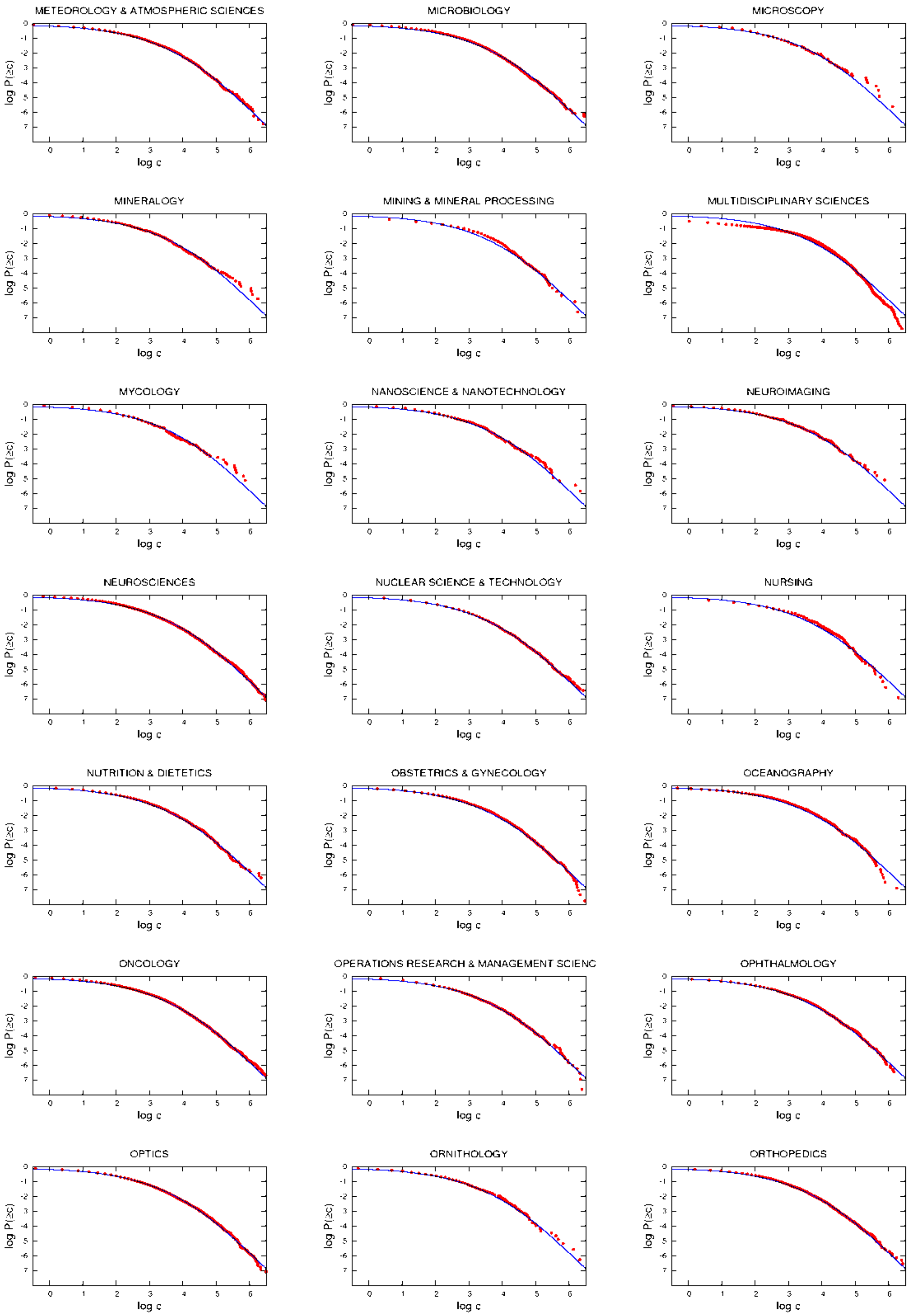

Figure S53: Publication year 1990.

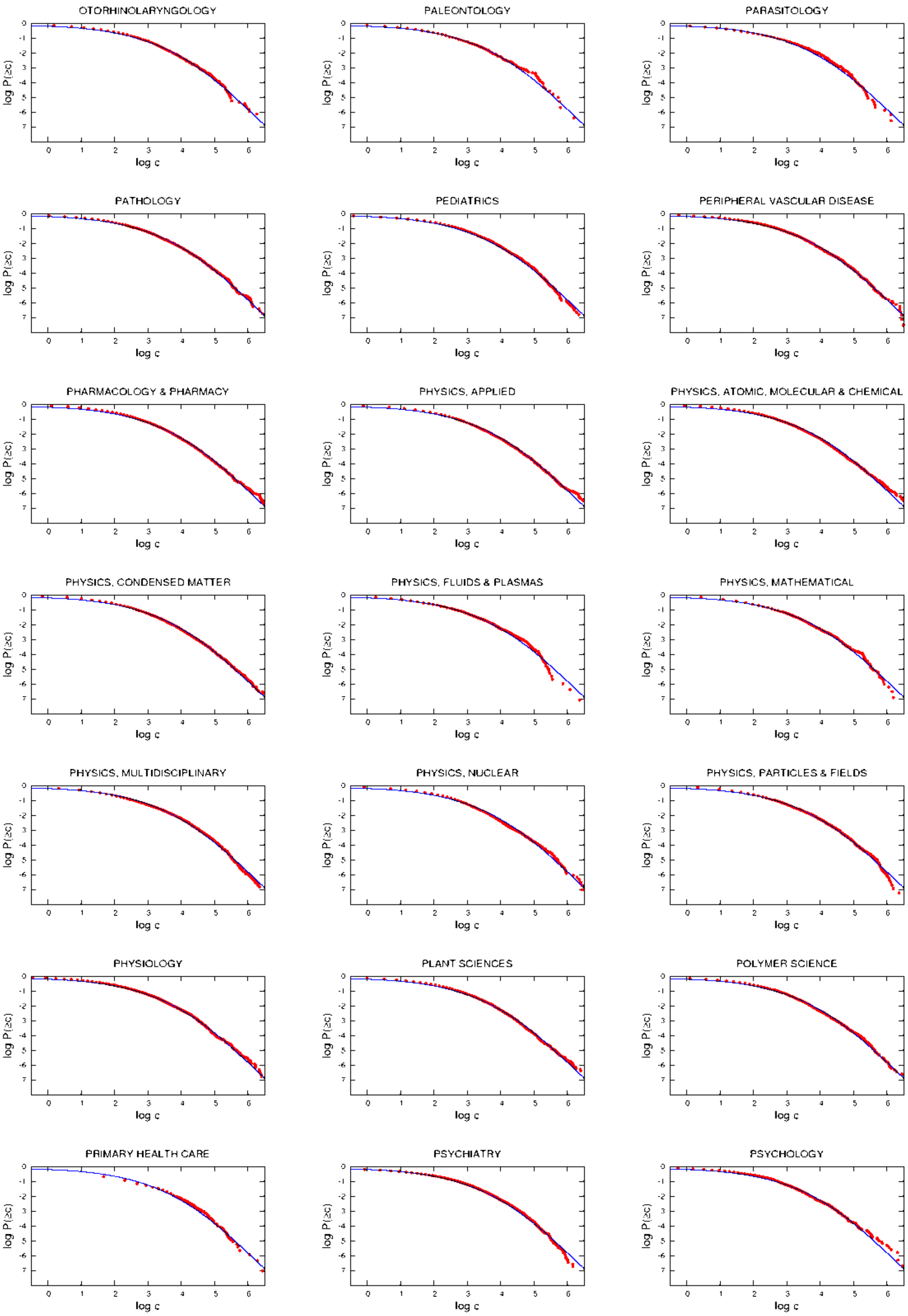

Figure S54: Publication year 1990.

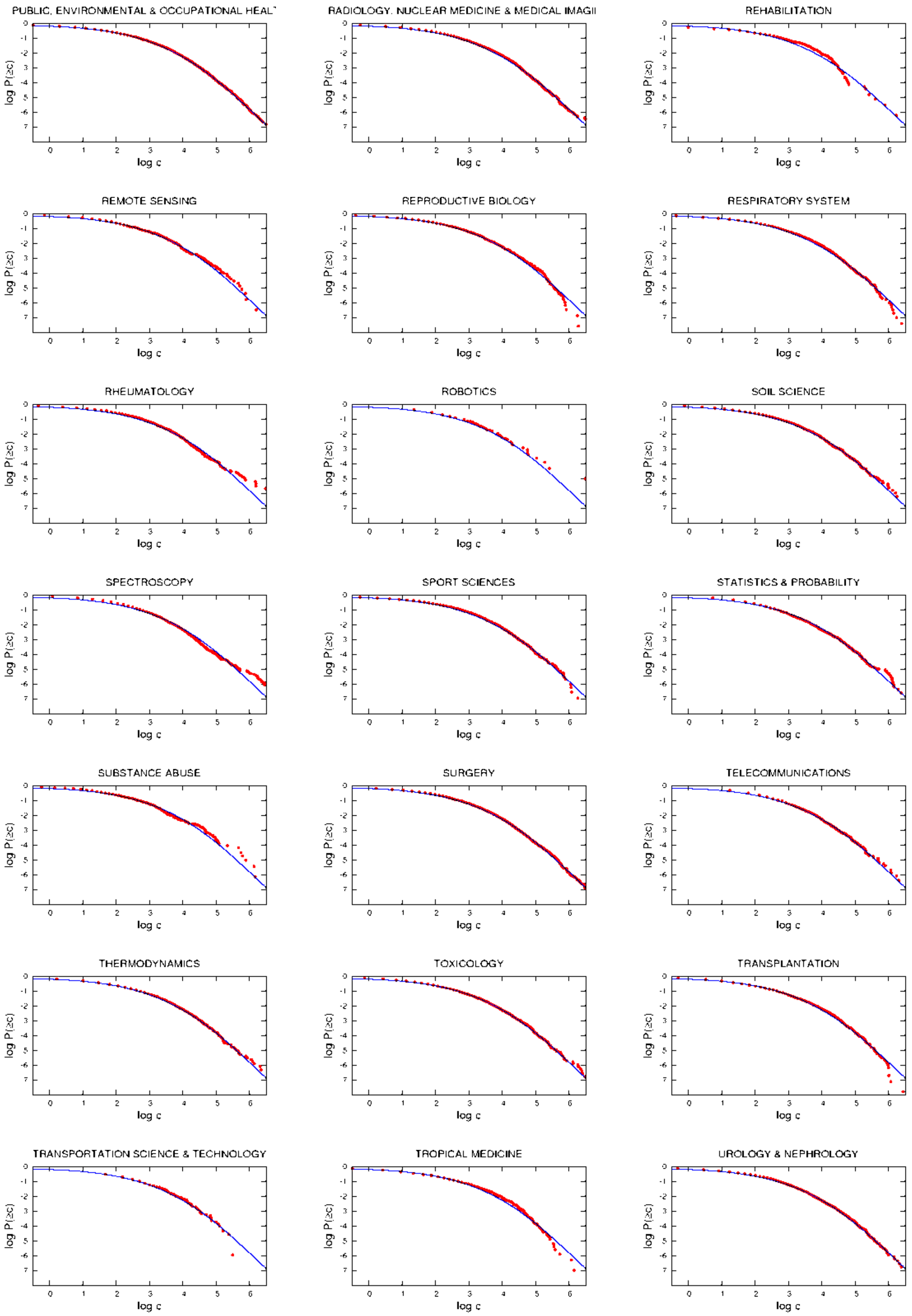

Figure S55: Publication year 1990.

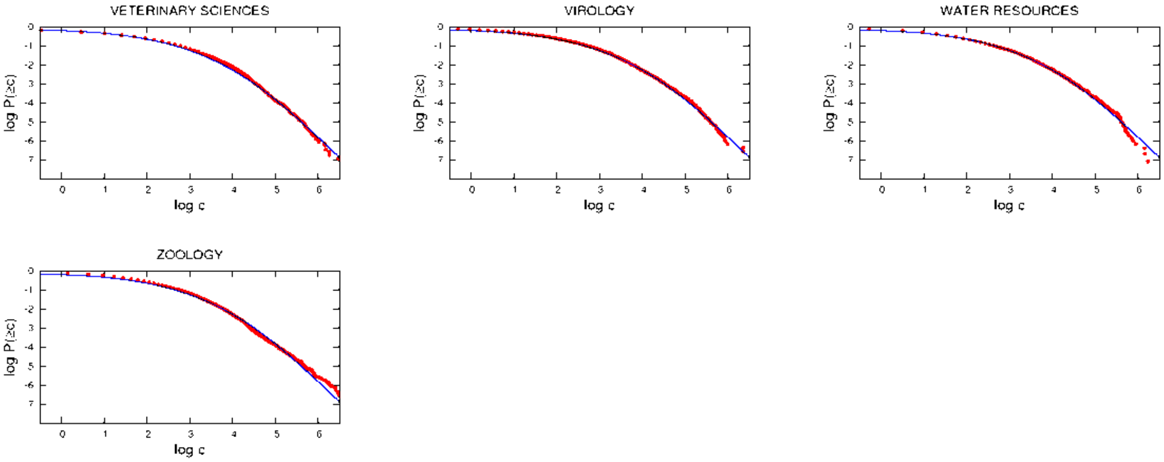

Figure S56: Publication year 1990.

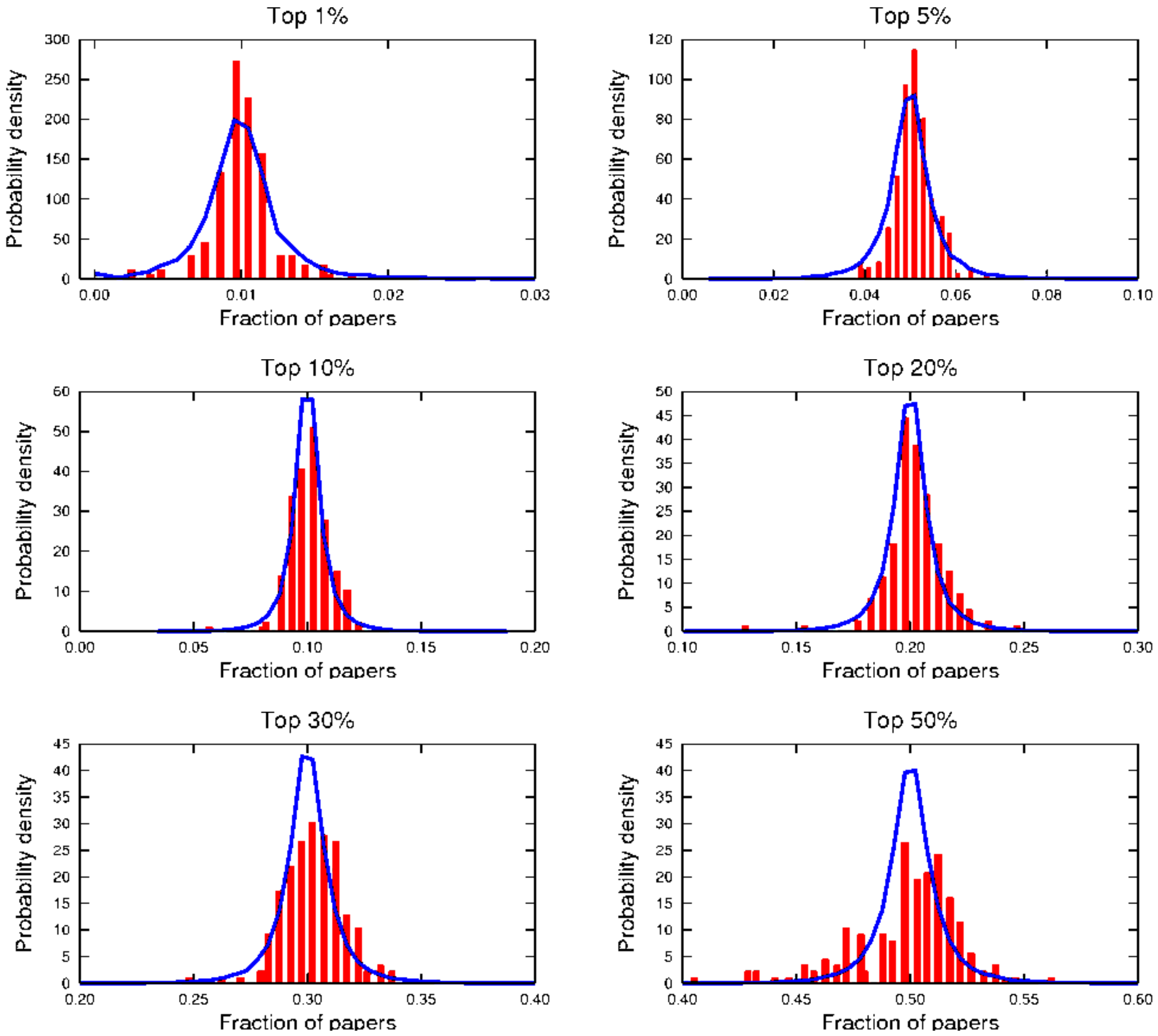

Figure S57: Publication year 1990.
