# Supplementary material for: A Reverse Engineering Approach to the Suppression of Citation Biases Reveals Universal Properties of Citation Distributions
Source: PLoS One. 2012 Mar 29;7(3):e33833. doi: 10.1371/journal.pone.0033833 (PMC3315498; doi:10.1371/journal.pone.0033833)
Supplement: Supporting Information S5 — Complete analysis for publication year . (PDF) [file pone.0033833.s005.pdf]

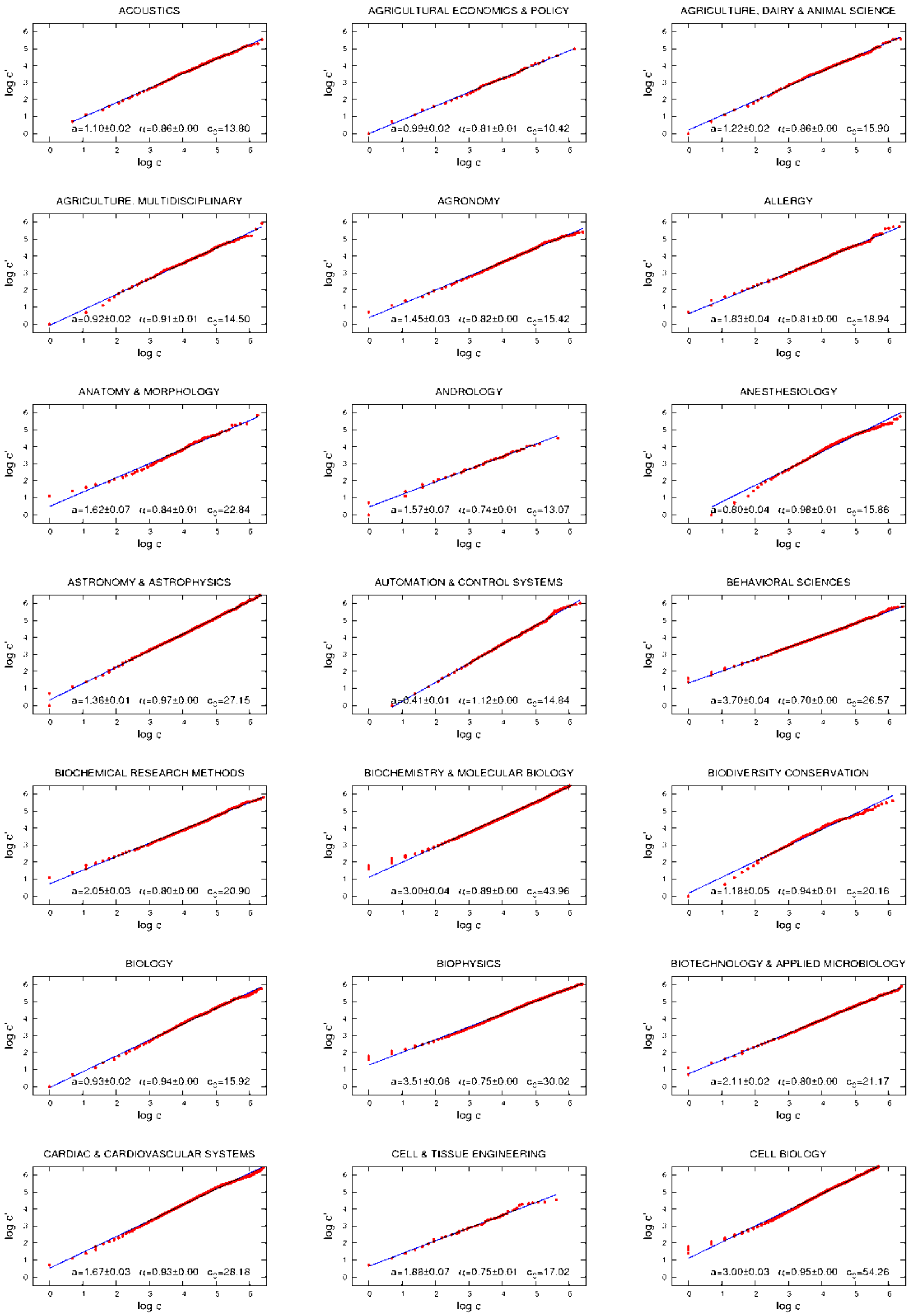

Figure S58: Publication year 1995.

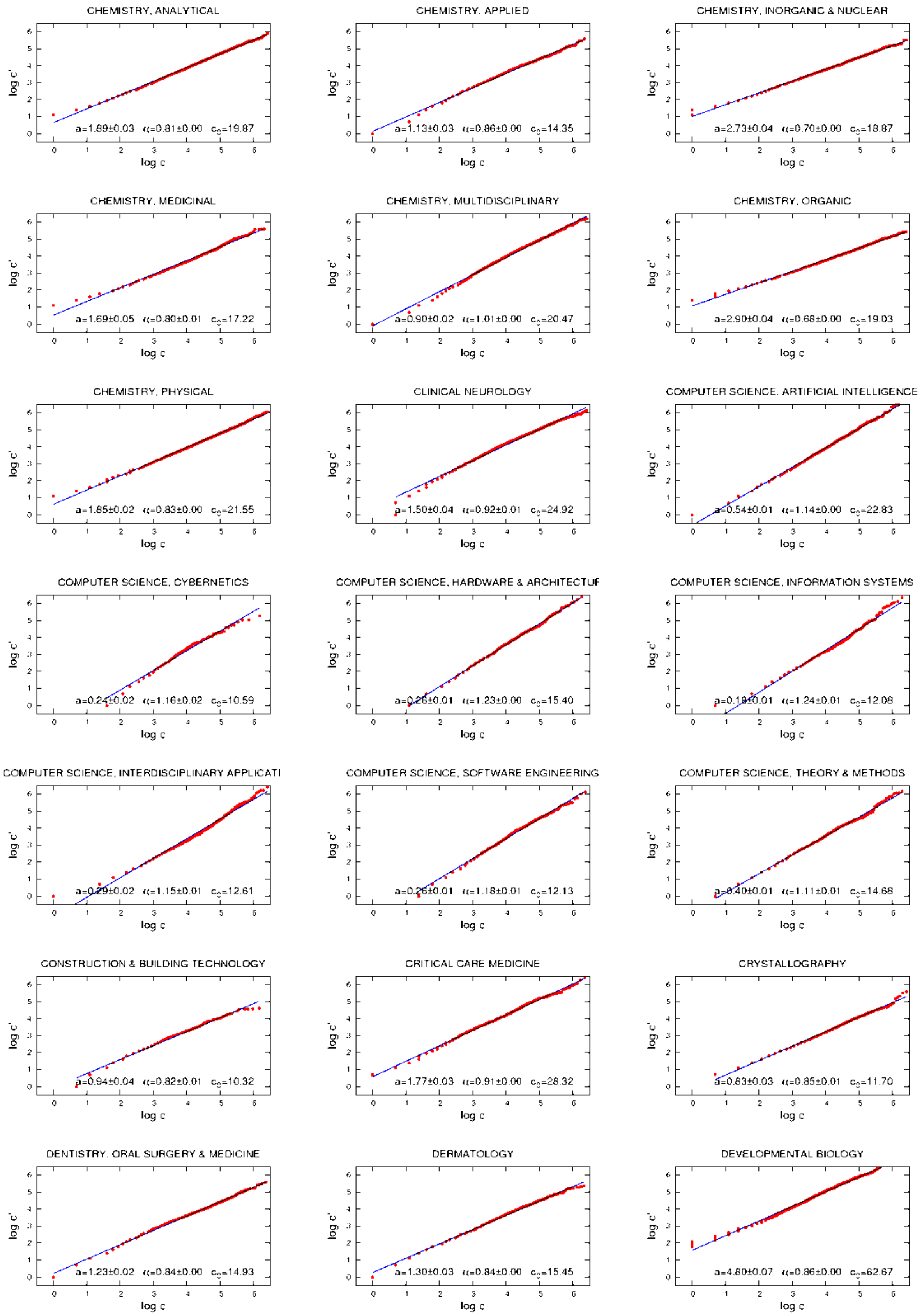

Figure S59: Publication year 1995.

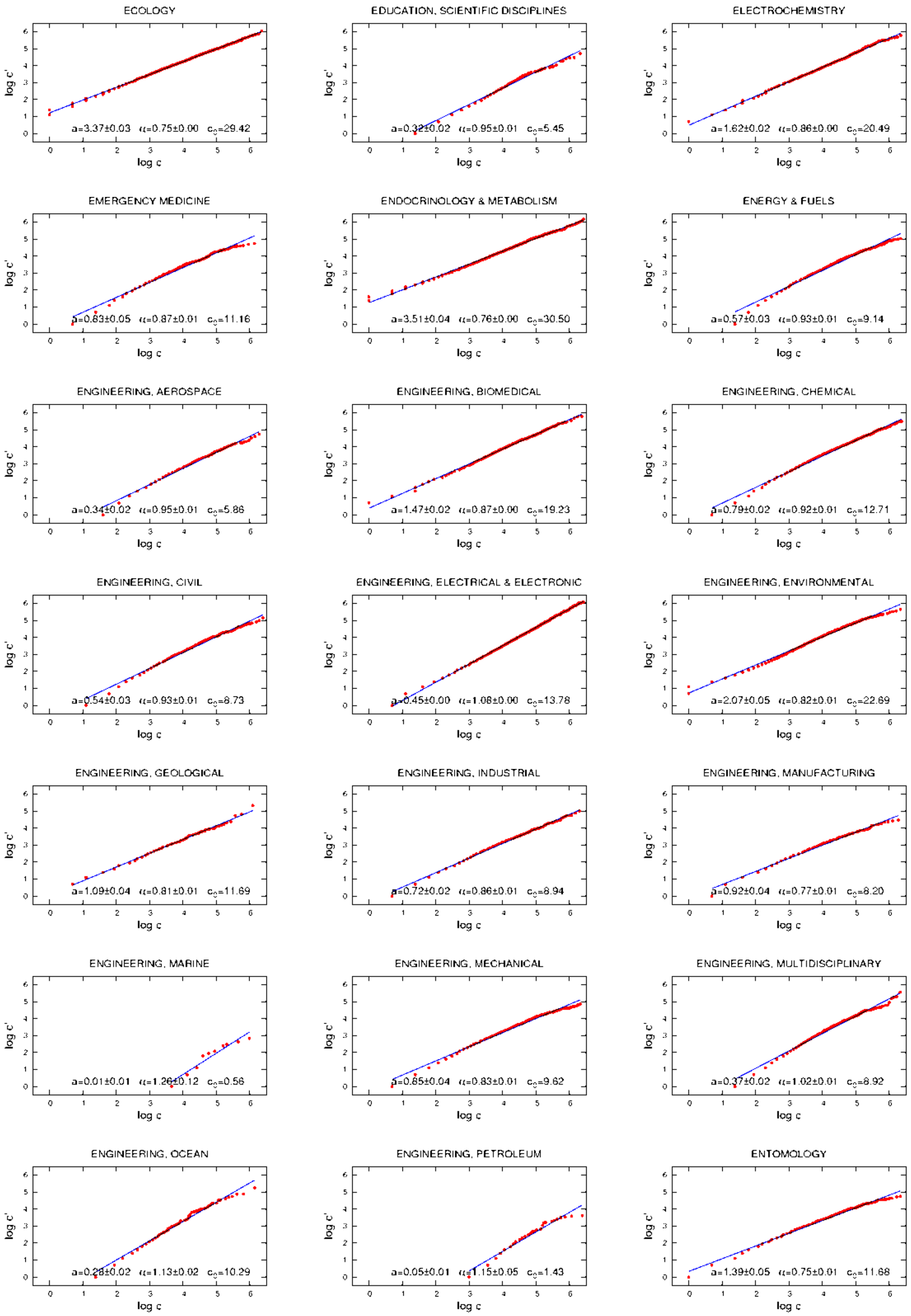

Figure S60: Publication year 1995.

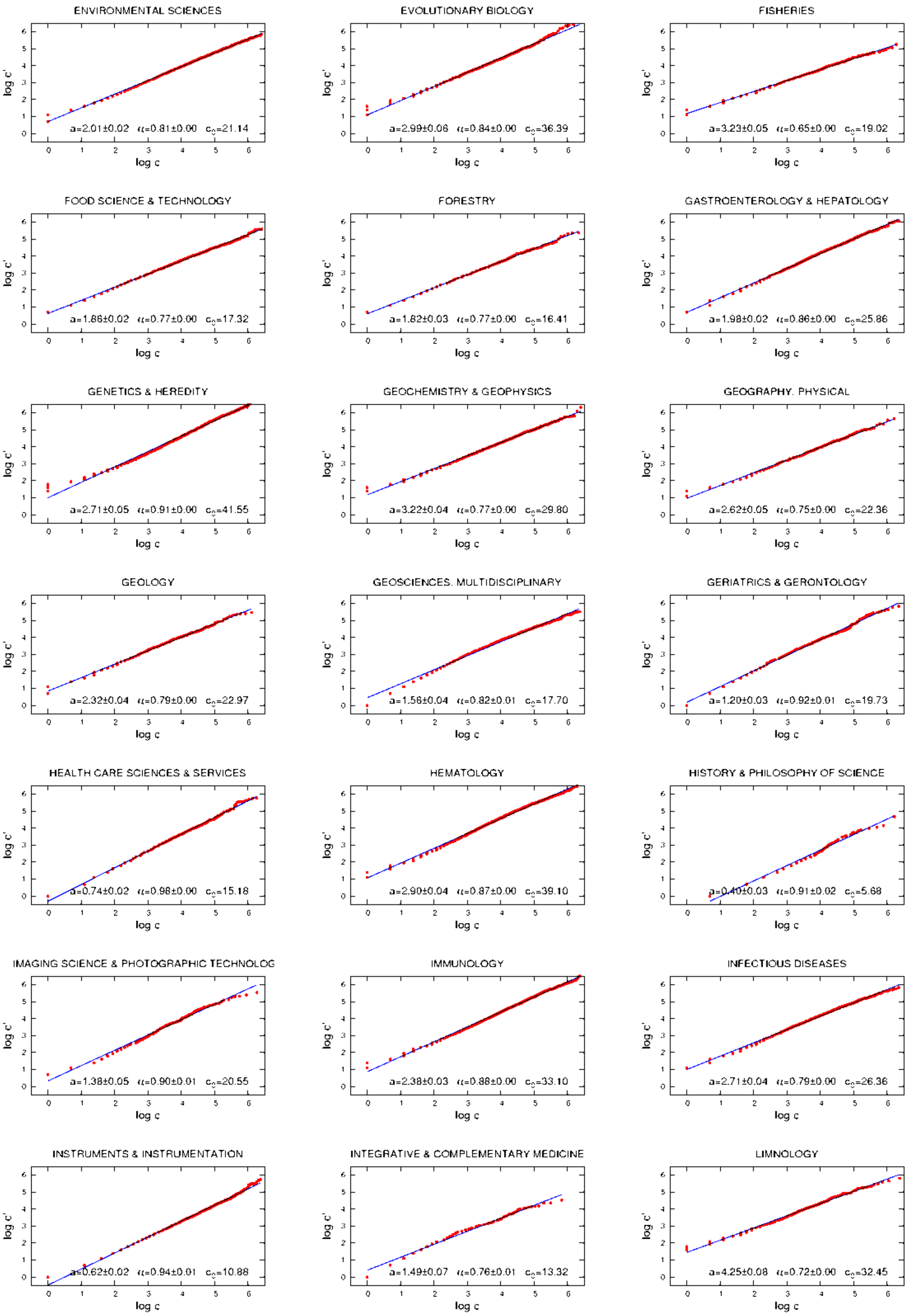

Figure S61: Publication year 1995.

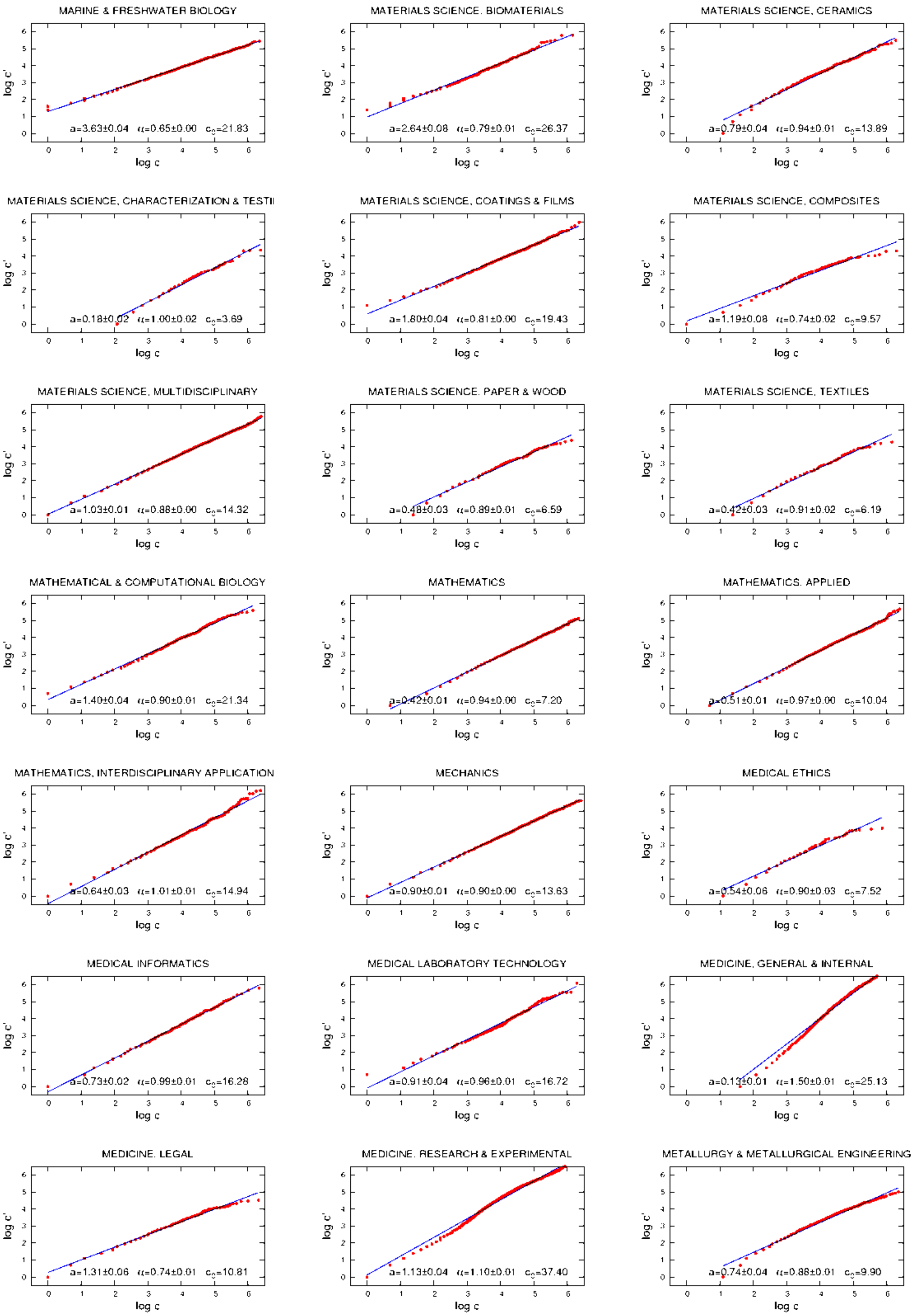

Figure S62: Publication year 1995.

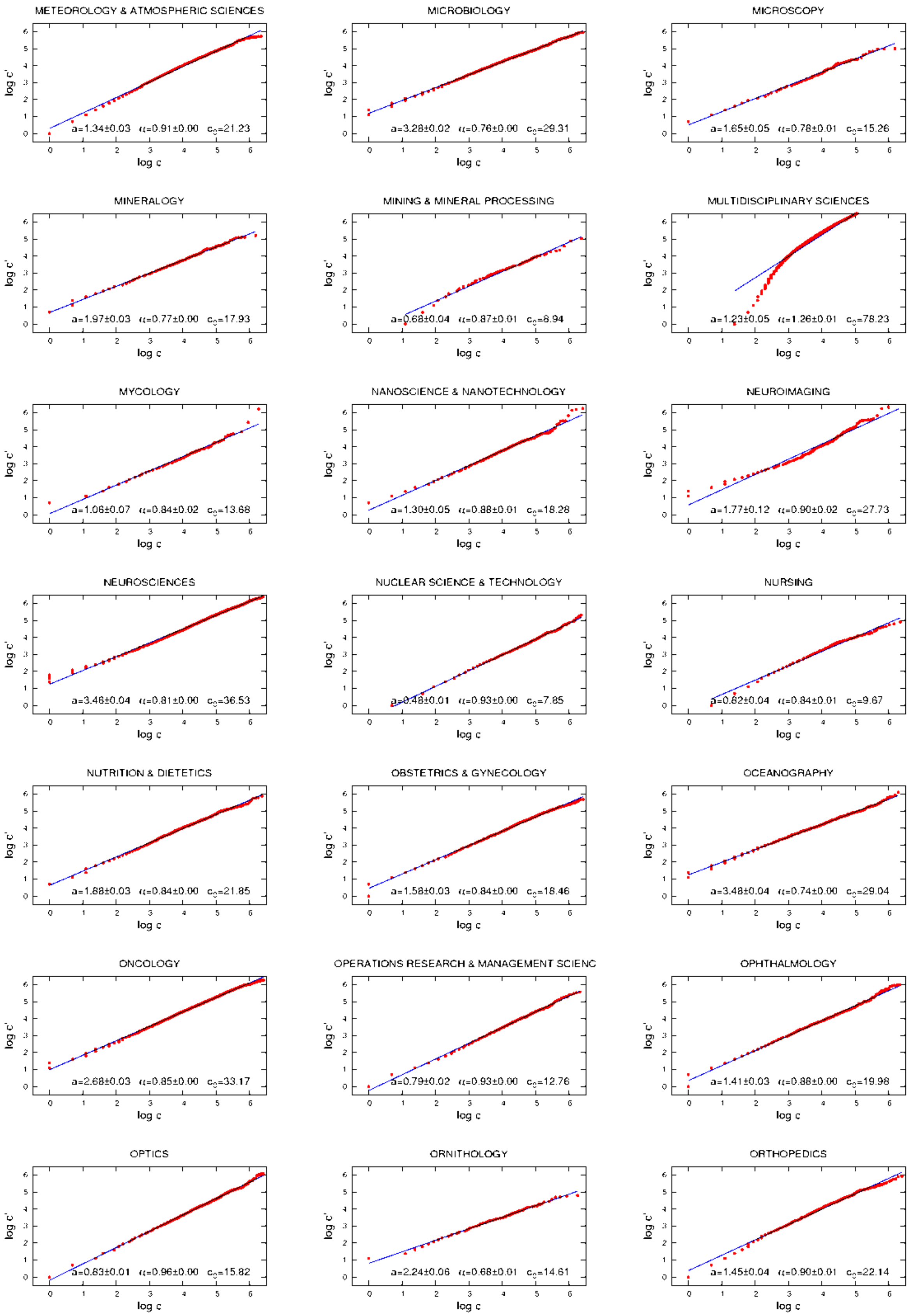

Figure S63: Publication year 1995.

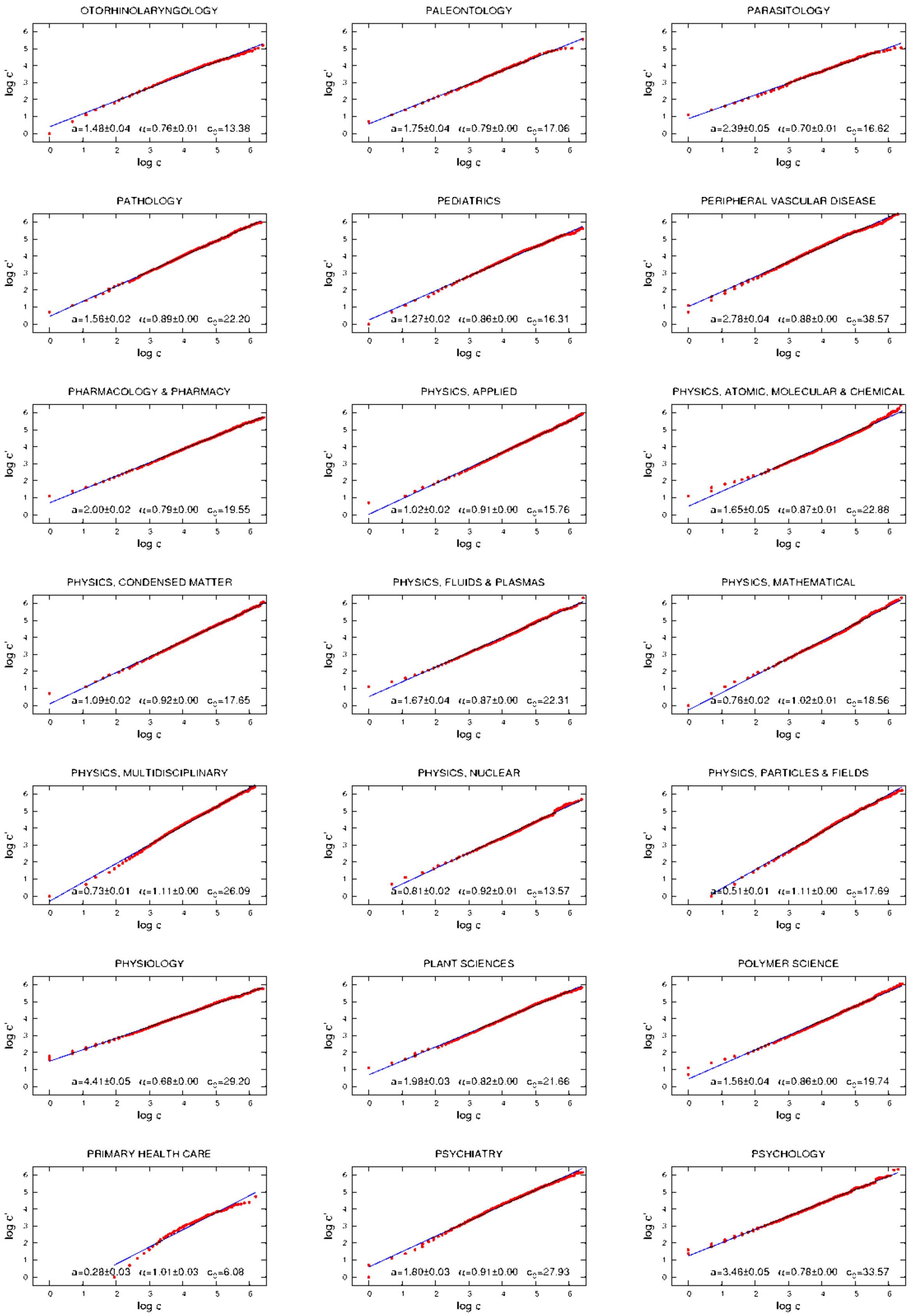

Figure S64: Publication year 1995.

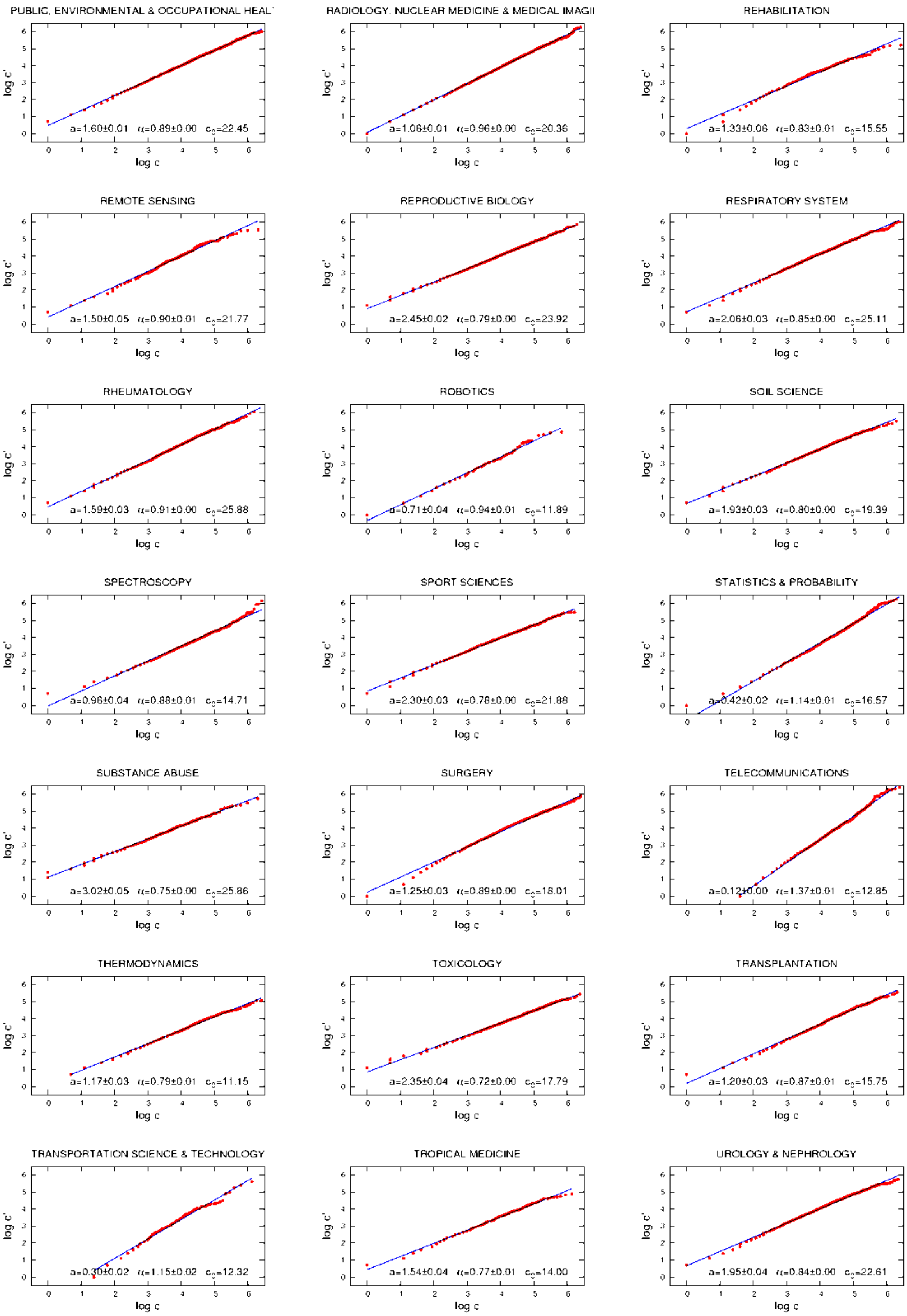

Figure S65: Publication year 1995.

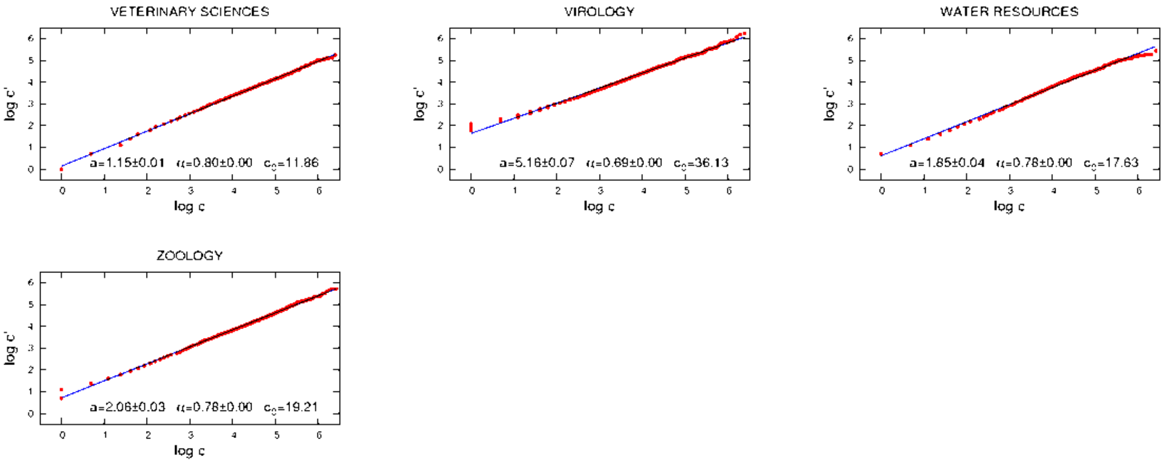

Figure S66: Publication year 1995.

Subject-category	a	α	$\langle c \rangle$	N
ACOUSTICS	1.10 ± 0.02	0.86 ± 0.00	13.80	2,719
AGRICULTURAL ECONOMICS & POLICY	0.99 ± 0.02	0.81 ± 0.01	10.42	576
AGRICULTURE, DAIRY & ANIMAL SCIENCE	1.22 ± 0.02	0.86 ± 0.00	15.90	2,610
AGRICULTURE, MULTIDISCIPLINARY	0.92 ± 0.02	0.91 ± 0.01	14.50	1,818
AGRONOMY	1.45 ± 0.03	0.82 ± 0.00	15.42	3,888
ALLERGY	1.83 ± 0.04	0.81 ± 0.00	18.94	1,625
ANATOMY & MORPHOLOGY	1.62 ± 0.07	0.84 ± 0.01	22.84	675
ANDROLOGY	1.57 ± 0.07	0.74 ± 0.01	13.07	201
ANESTHESIOLOGY	0.80 ± 0.04	0.98 ± 0.01	15.86	3,454
ASTRONOMY & ASTROPHYSICS	1.36 ± 0.01	0.97 ± 0.00	27.15	8,825
AUTOMATION & CONTROL SYSTEMS	0.41 ± 0.01	1.12 ± 0.00	14.84	2,485
BEHAVIORAL SCIENCES	3.70 ± 0.04	0.70 ± 0.00	26.57	2,950
BIOCHEMICAL RESEARCH METHODS	2.05 ± 0.03	0.80 ± 0.00	20.90	4,985
BIOCHEMISTRY & MOLECULAR BIOLOGY	3.00 ± 0.04	0.89 ± 0.00	43.96	34,475
BIODIVERSITY CONSERVATION	1.18 ± 0.05	0.94 ± 0.01	20.16	1,053
BIOLOGY	0.93 ± 0.02	0.94 ± 0.00	15.92	4,420
BIOPHYSICS	3.51 ± 0.06	0.75 ± 0.00	30.02	7,617
BIOTECHNOLOGY & APPLIED MICROBIOLOGY	2.11 ± 0.02	0.80 ± 0.00	21.17	9,366
CARDIAC & CARDIOVASCULAR SYSTEMS	1.67 ± 0.03	0.93 ± 0.00	28.18	9,760
CELL & TISSUE ENGINEERING	1.88 ± 0.07	0.75 ± 0.01	17.02	185
CELL BIOLOGY	3.00 ± 0.03	0.95 ± 0.00	54.26	14,729
CHEMISTRY, ANALYTICAL	1.89 ± 0.03	0.81 ± 0.00	19.87	9,948
CHEMISTRY, APPLIED	1.13 ± 0.03	0.86 ± 0.00	14.35	4,284
CHEMISTRY, INORGANIC & NUCLEAR	2.73 ± 0.04	0.70 ± 0.00	18.87	5,946
CHEMISTRY, MEDICINAL	1.69 ± 0.05	0.80 ± 0.01	17.22	4,043
CHEMISTRY, MULTIDISCIPLINARY	0.90 ± 0.02	1.01 ± 0.00	20.47	13,790
CHEMISTRY, ORGANIC	2.90 ± 0.04	0.68 ± 0.00	19.03	11,480
CHEMISTRY, PHYSICAL	1.85 ± 0.02	0.83 ± 0.00	21.55	17,819
CLINICAL NEUROLOGY	1.50 ± 0.04	0.92 ± 0.01	24.92	11,622
COMPUTER SCIENCE, ARTIFICIAL INTELLIGENCE	0.54 ± 0.01	1.14 ± 0.00	22.83	3,022
COMPUTER SCIENCE, CYBERNETICS	0.24 ± 0.02	1.16 ± 0.02	10.59	606
COMPUTER SCIENCE, HARDWARE & ARCHITECTURE	0.26 ± 0.01	1.23 ± 0.00	15.40	2,148
COMPUTER SCIENCE, INFORMATION SYSTEMS	0.18 ± 0.01	1.24 ± 0.01	12.08	2,271
COMPUTER SCIENCE, INTERDISCIPLINARY APPLICATIONS	0.29 ± 0.02	1.15 ± 0.01	12.61	3,913
COMPUTER SCIENCE, SOFTWARE ENGINEERING	0.26 ± 0.01	1.18 ± 0.01	12.13	2,709
COMPUTER SCIENCE, THEORY & METHODS	0.40 ± 0.01	1.11 ± 0.01	14.68	3,085
CONSTRUCTION & BUILDING TECHNOLOGY	0.94 ± 0.04	0.82 ± 0.01	10.32	1,170
CRITICAL CARE MEDICINE	1.77 ± 0.03	0.91 ± 0.00	28.32	2,641
CRYSTALLOGRAPHY	0.83 ± 0.03	0.85 ± 0.01	11.70	3,909
DENTISTRY, ORAL SURGERY & MEDICINE	1.23 ± 0.02	0.84 ± 0.00	14.93	3,608
DERMATOLOGY	1.30 ± 0.03	0.84 ± 0.00	15.45	4,152
DEVELOPMENTAL BIOLOGY	4.80 ± 0.07	0.86 ± 0.00	62.67	2,265
ECOLOGY	3.37 ± 0.03	0.75 ± 0.00	29.42	6,127
EDUCATION, SCIENTIFIC DISCIPLINES	0.32 ± 0.02	0.95 ± 0.01	5.45	1,635
ELECTROCHEMISTRY	1.62 ± 0.02	0.86 ± 0.00	20.49	3,399
EMERGENCY MEDICINE	0.83 ± 0.05	0.87 ± 0.01	11.16	1,107
ENDOCRINOLOGY & METABOLISM	3.51 ± 0.04	0.76 ± 0.00	30.50	8,898
ENERGY & FUELS	0.57 ± 0.03	0.93 ± 0.01	9.14	4,235
ENGINEERING, AEROSPACE	0.34 ± 0.02	0.95 ± 0.01	5.86	2,276
ENGINEERING, BIOMEDICAL	1.47 ± 0.02	0.87 ± 0.00	19.23	2,711

Table S25: Publication year 1995.

Subject-category	a	α	$\langle c \rangle$	N
ENGINEERING, CHEMICAL	0.79 ± 0.02	0.92 ± 0.01	12.71	9,116
ENGINEERING, CIVIL	0.54 ± 0.03	0.93 ± 0.01	8.73	3,780
ENGINEERING, ELECTRICAL & ELECTRONIC	0.45 ± 0.00	1.08 ± 0.00	13.78	19,938
ENGINEERING, ENVIRONMENTAL	2.07 ± 0.05	0.82 ± 0.01	22.69	2,516
ENGINEERING, GEOLOGICAL	1.09 ± 0.04	0.81 ± 0.01	11.69	494
ENGINEERING, INDUSTRIAL	0.72 ± 0.02	0.86 ± 0.01	8.94	2,347
ENGINEERING, MANUFACTURING	0.92 ± 0.04	0.77 ± 0.01	8.20	1,505
ENGINEERING, MARINE	0.01 ± 0.01	1.26 ± 0.12	0.56	396
ENGINEERING, MECHANICAL	0.85 ± 0.04	0.83 ± 0.01	9.62	5,749
ENGINEERING, MULTIDISCIPLINARY	0.37 ± 0.02	1.02 ± 0.01	8.92	3,267
ENGINEERING, OCEAN	0.28 ± 0.02	1.13 ± 0.02	10.29	549
ENGINEERING, PETROLEUM	0.05 ± 0.01	1.15 ± 0.05	1.43	932
ENTOMOLOGY	1.39 ± 0.05	0.75 ± 0.01	11.68	3,338
ENVIRONMENTAL SCIENCES	2.01 ± 0.02	0.81 ± 0.00	21.14	10,689
EVOLUTIONARY BIOLOGY	2.99 ± 0.06	0.84 ± 0.00	36.39	1,789
FISHERIES	3.23 ± 0.05	0.65 ± 0.00	19.02	2,229
FOOD SCIENCE & TECHNOLOGY	1.86 ± 0.02	0.77 ± 0.00	17.32	5,851
FORESTRY	1.82 ± 0.03	0.77 ± 0.00	16.41	1,643
GASTROENTEROLOGY & HEPATOLOGY	1.98 ± 0.02	0.86 ± 0.00	25.86	6,025
GENETICS & HEREDITY	2.71 ± 0.05	0.91 ± 0.00	41.55	9,477
GEOCHEMISTRY & GEOPHYSICS	3.22 ± 0.04	0.77 ± 0.00	29.80	3,829
GEOGRAPHY, PHYSICAL	2.62 ± 0.05	0.75 ± 0.00	22.36	1,281
GEOLOGY	2.32 ± 0.04	0.79 ± 0.00	22.97	1,015
GEOSCIENCES, MULTIDISCIPLINARY	1.56 ± 0.04	0.82 ± 0.01	17.70	6,210
GERIATRICS & GERONTOLOGY	1.20 ± 0.03	0.92 ± 0.01	19.73	1,730
HEALTH CARE SCIENCES & SERVICES	0.74 ± 0.02	0.98 ± 0.00	15.18	2,122
HEMATOLOGY	2.90 ± 0.04	0.87 ± 0.00	39.10	7,651
HISTORY & PHILOSOPHY OF SCIENCE	0.40 ± 0.03	0.91 ± 0.02	5.68	647
IMAGING SCIENCE & PHOTOGRAPHIC TECHNOLOGY	1.38 ± 0.05	0.90 ± 0.01	20.55	726
IMMUNOLOGY	2.38 ± 0.03	0.88 ± 0.00	33.10	15,607
INFECTIOUS DISEASES	2.71 ± 0.04	0.79 ± 0.00	26.36	5,173
INSTRUMENTS & INSTRUMENTATION	0.62 ± 0.02	0.94 ± 0.01	10.88	7,175
INTEGRATIVE & COMPLEMENTARY MEDICINE	1.49 ± 0.07	0.76 ± 0.01	13.32	282
LIMNOLOGY	4.25 ± 0.08	0.72 ± 0.00	32.45	911
MARINE & FRESHWATER BIOLOGY	3.63 ± 0.04	0.65 ± 0.00	21.83	5,200
MATERIALS SCIENCE, BIOMATERIALS	2.64 ± 0.08	0.79 ± 0.01	26.37	574
MATERIALS SCIENCE, CERAMICS	0.79 ± 0.04	0.94 ± 0.01	13.89	2,171
MATERIALS SCIENCE, CHARACTERIZATION & TESTING	0.18 ± 0.02	1.00 ± 0.02	3.69	920
MATERIALS SCIENCE, COATINGS & FILMS	1.80 ± 0.04	0.81 ± 0.00	19.43	2,600
MATERIALS SCIENCE, COMPOSITES	1.19 ± 0.08	0.74 ± 0.02	9.57	770
MATERIALS SCIENCE, MULTIDISCIPLINARY	1.03 ± 0.01	0.88 ± 0.00	14.32	18,071
MATERIALS SCIENCE, PAPER & WOOD	0.48 ± 0.03	0.89 ± 0.01	6.59	1,059
MATERIALS SCIENCE, TEXTILES	0.42 ± 0.03	0.91 ± 0.02	6.19	561
MATHEMATICAL & COMPUTATIONAL BIOLOGY	1.40 ± 0.04	0.90 ± 0.01	21.34	1,129
MATHEMATICS	0.42 ± 0.01	0.94 ± 0.00	7.20	10,070
MATHEMATICS, APPLIED	0.51 ± 0.01	0.97 ± 0.00	10.04	7,404
MATHEMATICS, INTERDISCIPLINARY APPLICATIONS	0.64 ± 0.03	1.01 ± 0.01	14.94	2,751
MECHANICS	0.90 ± 0.01	0.90 ± 0.00	13.63	6,810
MEDICAL ETHICS	0.54 ± 0.06	0.90 ± 0.03	7.52	305
MEDICAL INFORMATICS	0.73 ± 0.02	0.99 ± 0.01	16.28	835

Table S26: Publication year 1995.

Subject-category	a	α	$\langle c \rangle$	N
MEDICAL LABORATORY TECHNOLOGY	0.91 ± 0.04	0.96 ± 0.01	16.72	1,524
MEDICINE, GENERAL & INTERNAL	0.13 ± 0.01	1.50 ± 0.01	25.13	15,177
MEDICINE, LEGAL	1.31 ± 0.06	0.74 ± 0.01	10.81	806
MEDICINE, RESEARCH & EXPERIMENTAL	1.13 ± 0.04	1.10 ± 0.01	37.40	7,096
METALLURGY & METALLURGICAL ENGINEERING	0.74 ± 0.04	0.88 ± 0.01	9.90	3,460
METEOROLOGY & ATMOSPHERIC SCIENCES	1.34 ± 0.03	0.91 ± 0.00	21.23	5,137
MICROBIOLOGY	3.28 ± 0.02	0.76 ± 0.00	29.31	9,711
MICROSCOPY	1.65 ± 0.05	0.78 ± 0.01	15.26	607
MINERALOGY	1.97 ± 0.03	0.77 ± 0.00	17.93	1,201
MINING & MINERAL PROCESSING	0.68 ± 0.04	0.87 ± 0.01	8.94	901
MULTIDISCIPLINARY SCIENCES	1.23 ± 0.05	1.26 ± 0.01	78.23	10,747
MYCOLOGY	1.06 ± 0.07	0.84 ± 0.02	13.68	740
NANOSCIENCE & NANOTECHNOLOGY	1.30 ± 0.05	0.88 ± 0.01	18.28	1,921
NEUROIMAGING	1.77 ± 0.12	0.90 ± 0.02	27.73	786
NEUROSCIENCES	3.46 ± 0.04	0.81 ± 0.00	36.53	18,196
NUCLEAR SCIENCE & TECHNOLOGY	0.48 ± 0.01	0.93 ± 0.00	7.85	7,094
NURSING	0.82 ± 0.04	0.84 ± 0.01	9.67	1,694
NUTRITION & DIETETICS	1.88 ± 0.03	0.84 ± 0.00	21.85	3,582
OBSTETRICS & GYNECOLOGY	1.58 ± 0.03	0.84 ± 0.00	18.46	5,886
OCEANOGRAPHY	3.48 ± 0.04	0.74 ± 0.00	29.04	3,058
ONCOLOGY	2.68 ± 0.03	0.85 ± 0.00	33.17	13,851
OPERATIONS RESEARCH & MANAGEMENT SCIENCE	0.79 ± 0.02	0.93 ± 0.00	12.76	3,257
OPHTHALMOLOGY	1.41 ± 0.03	0.88 ± 0.00	19.98	4,086
OPTICS	0.83 ± 0.01	0.96 ± 0.00	15.82	9,713
ORNITHOLOGY	2.24 ± 0.06	0.68 ± 0.01	14.61	696
ORTHOPEDICS	1.45 ± 0.04	0.90 ± 0.01	22.14	3,826
OTORHINOLARYNGOLOGY	1.48 ± 0.04	0.76 ± 0.01	13.38	2,863
PALEONTOLOGY	1.75 ± 0.04	0.79 ± 0.00	17.06	964
PARASITOLOGY	2.39 ± 0.05	0.70 ± 0.01	16.62	1,798
PATHOLOGY	1.56 ± 0.02	0.89 ± 0.00	22.20	5,140
PEDIATRICS	1.27 ± 0.02	0.86 ± 0.00	16.31	7,744
PERIPHERAL VASCULAR DISEASE	2.78 ± 0.04	0.88 ± 0.00	38.57	6,683
PHARMACOLOGY & PHARMACY	2.00 ± 0.02	0.79 ± 0.00	19.55	17,962
PHYSICS, APPLIED	1.02 ± 0.02	0.91 ± 0.00	15.76	17,273
PHYSICS, ATOMIC, MOLECULAR & CHEMICAL	1.65 ± 0.05	0.87 ± 0.01	22.88	8,798
PHYSICS, CONDENSED MATTER	1.09 ± 0.02	0.92 ± 0.00	17.65	15,933
PHYSICS, FLUIDS & PLASMAS	1.67 ± 0.04	0.87 ± 0.00	22.31	3,972
PHYSICS, MATHEMATICAL	0.76 ± 0.02	1.02 ± 0.01	18.56	4,618
PHYSICS, MULTIDISCIPLINARY	0.73 ± 0.01	1.11 ± 0.00	26.09	10,727
PHYSICS, NUCLEAR	0.81 ± 0.02	0.92 ± 0.01	13.57	4,457
PHYSICS, PARTICLES & FIELDS	0.51 ± 0.01	1.11 ± 0.00	17.69	5,655
PHYSIOLOGY	4.41 ± 0.05	0.68 ± 0.00	29.20	6,698
PLANT SCIENCES	1.98 ± 0.03	0.82 ± 0.00	21.66	11,670
POLYMER SCIENCE	1.56 ± 0.04	0.86 ± 0.00	19.74	7,586
PRIMARY HEALTH CARE	0.28 ± 0.03	1.01 ± 0.03	6.08	1,196
PSYCHIATRY	1.80 ± 0.03	0.91 ± 0.00	27.93	6,617
PSYCHOLOGY	3.46 ± 0.05	0.78 ± 0.00	33.57	2,246
PUBLIC, ENVIRONMENTAL & OCCUPATIONAL HEALTH	1.60 ± 0.01	0.89 ± 0.00	22.45	6,847
RADIOLOGY, NUCLEAR MEDICINE & MEDICAL IMAGING	1.06 ± 0.01	0.96 ± 0.00	20.36	9,628
REHABILITATION	1.33 ± 0.06	0.83 ± 0.01	15.55	986

Table S27: Publication year 1995.

Subject-category	a	α	$\langle c \rangle$	N
REMOTE SENSING	1.50 ± 0.05	0.90 ± 0.01	21.77	797
REPRODUCTIVE BIOLOGY	2.45 ± 0.02	0.79 ± 0.00	23.92	3,055
RESPIRATORY SYSTEM	2.06 ± 0.03	0.85 ± 0.00	25.11	5,483
RHEUMATOLOGY	1.59 ± 0.03	0.91 ± 0.00	25.88	1,832
ROBOTICS	0.71 ± 0.04	0.94 ± 0.01	11.89	282
SOIL SCIENCE	1.93 ± 0.03	0.80 ± 0.00	19.39	2,286
SPECTROSCOPY	0.96 ± 0.04	0.88 ± 0.01	14.71	4,871
SPORT SCIENCES	2.30 ± 0.03	0.78 ± 0.00	21.88	2,589
STATISTICS & PROBABILITY	0.42 ± 0.02	1.14 ± 0.01	16.57	3,710
SUBSTANCE ABUSE	3.02 ± 0.05	0.75 ± 0.00	25.86	778
SURGERY	1.25 ± 0.03	0.89 ± 0.00	18.01	18,445
TELECOMMUNICATIONS	0.12 ± 0.00	1.37 ± 0.01	12.85	2,788
THERMODYNAMICS	1.17 ± 0.03	0.79 ± 0.01	11.15	2,860
TOXICOLOGY	2.35 ± 0.04	0.72 ± 0.00	17.79	4,475
TRANSPLANTATION	1.20 ± 0.03	0.87 ± 0.01	15.75	4,038
TRANSPORTATION SCIENCE & TECHNOLOGY	0.30 ± 0.02	1.15 ± 0.02	12.32	520
TROPICAL MEDICINE	1.54 ± 0.04	0.77 ± 0.01	14.00	1,075
UROLOGY & NEPHROLOGY	1.95 ± 0.04	0.84 ± 0.00	22.61	5,086
VETERINARY SCIENCES	1.15 ± 0.01	0.80 ± 0.00	11.86	5,685
VIROLOGY	5.16 ± 0.07	0.69 ± 0.00	36.13	3,743
WATER RESOURCES	1.85 ± 0.04	0.78 ± 0.00	17.63	3,904
ZOOLOGY	2.06 ± 0.03	0.78 ± 0.00	19.21	4,986
**TOTAL**	1.00 ± 0.00	1.00 ± 0.00	22.97	856,556

Table S28: Publication year 1995.

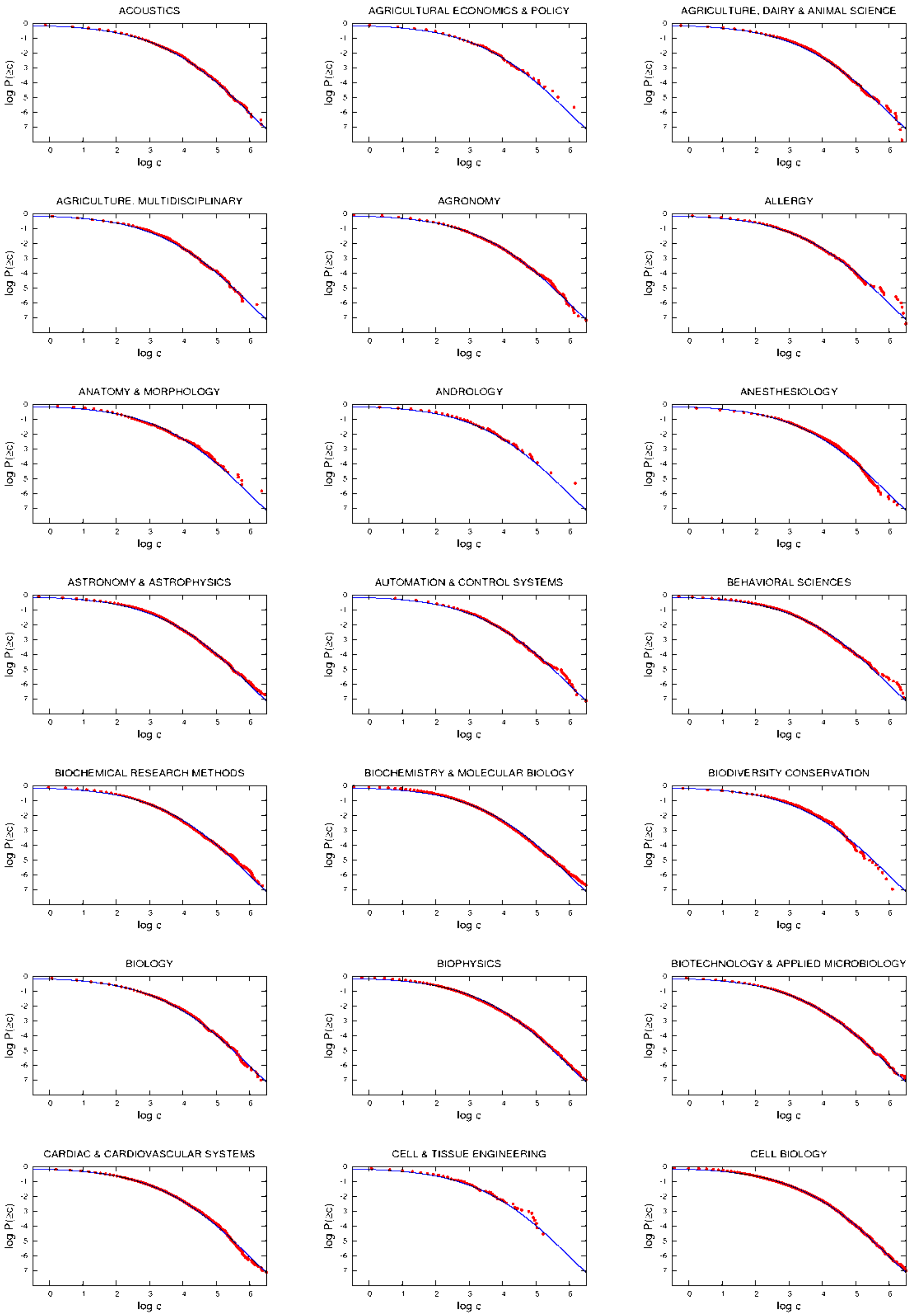

Figure S67: Publication year 1995.

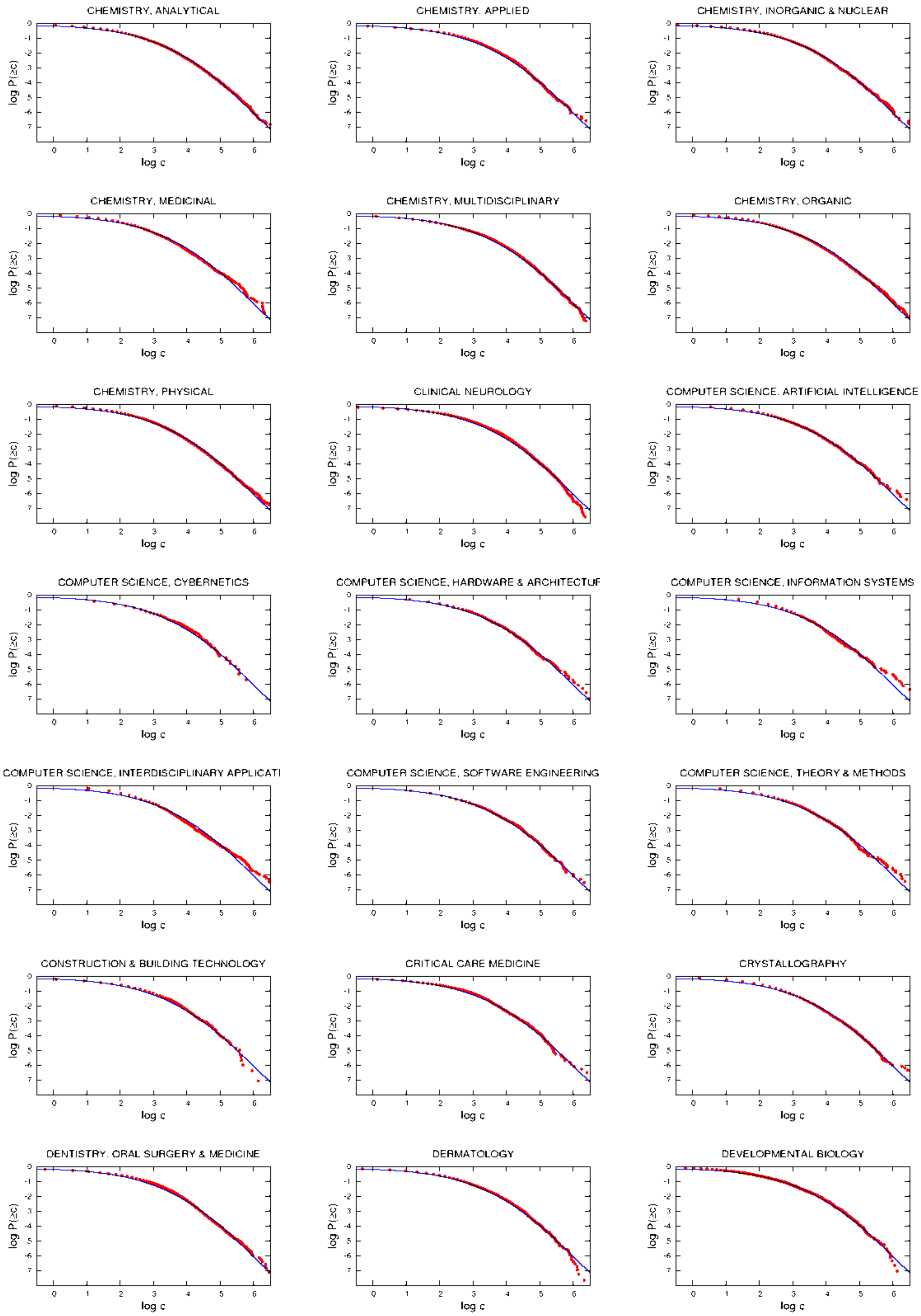

Figure S68: Publication year 1995.

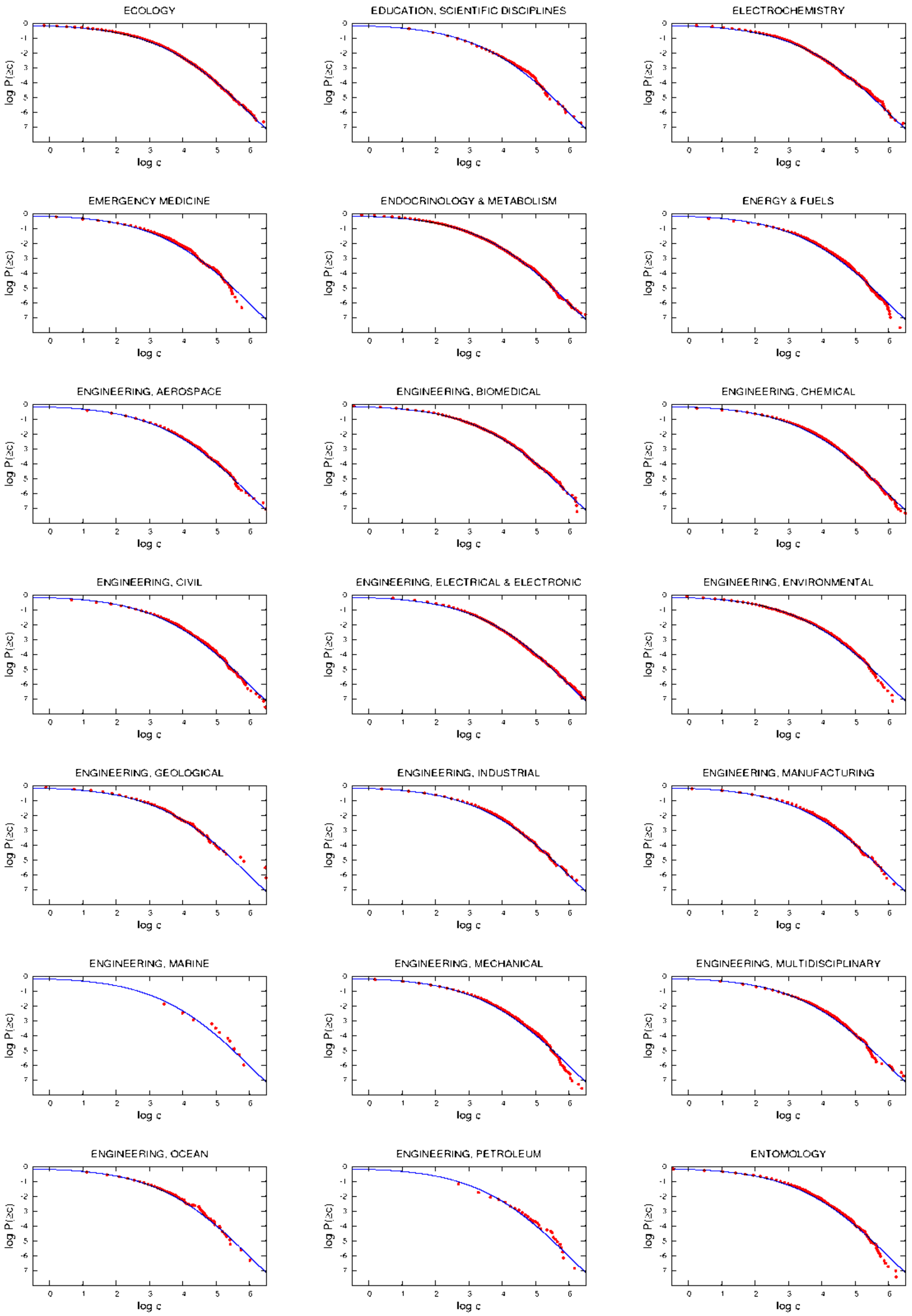

Figure S69: Publication year 1995.

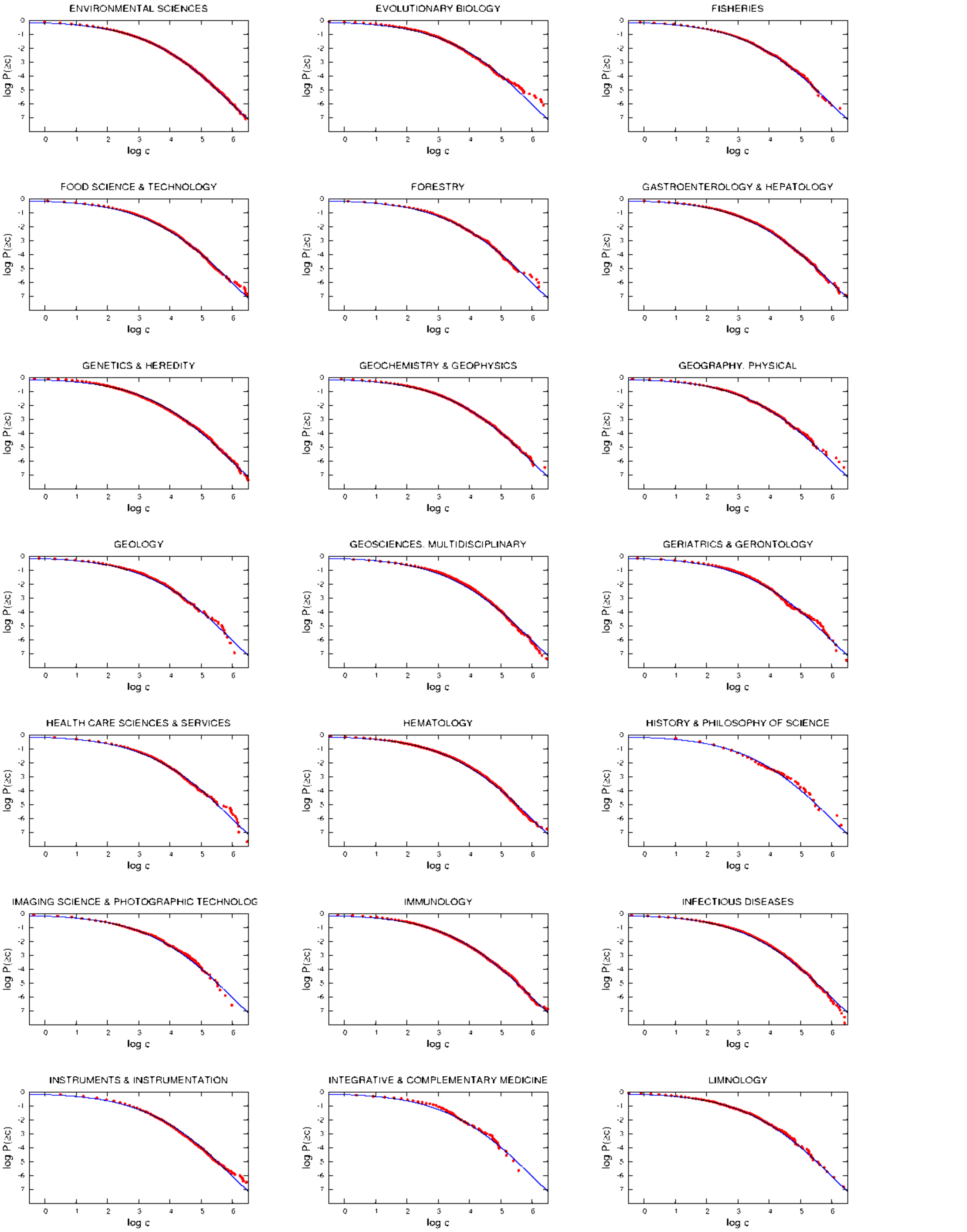

Figure S70: Publication year 1995.

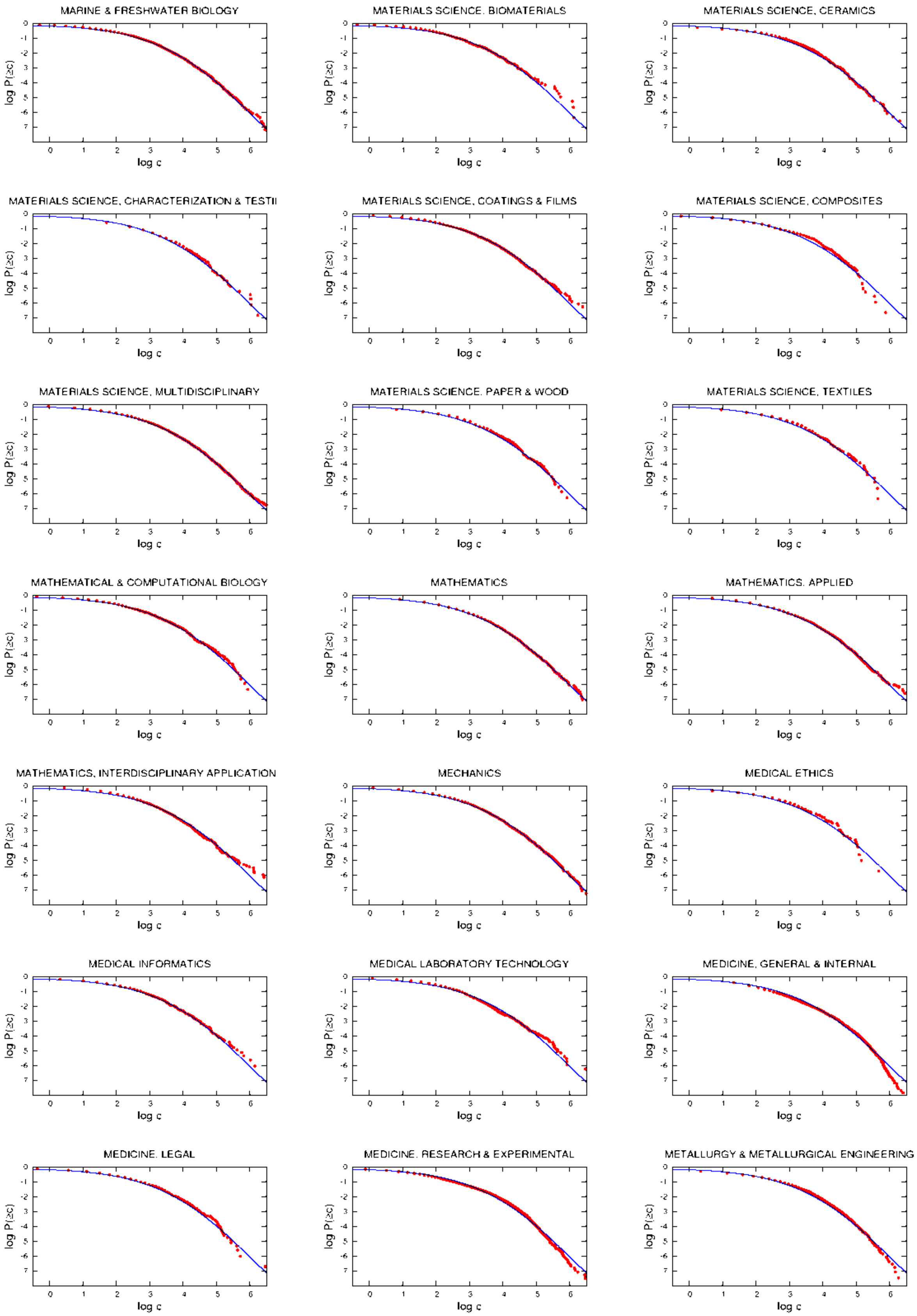

Figure S71: Publication year 1995.

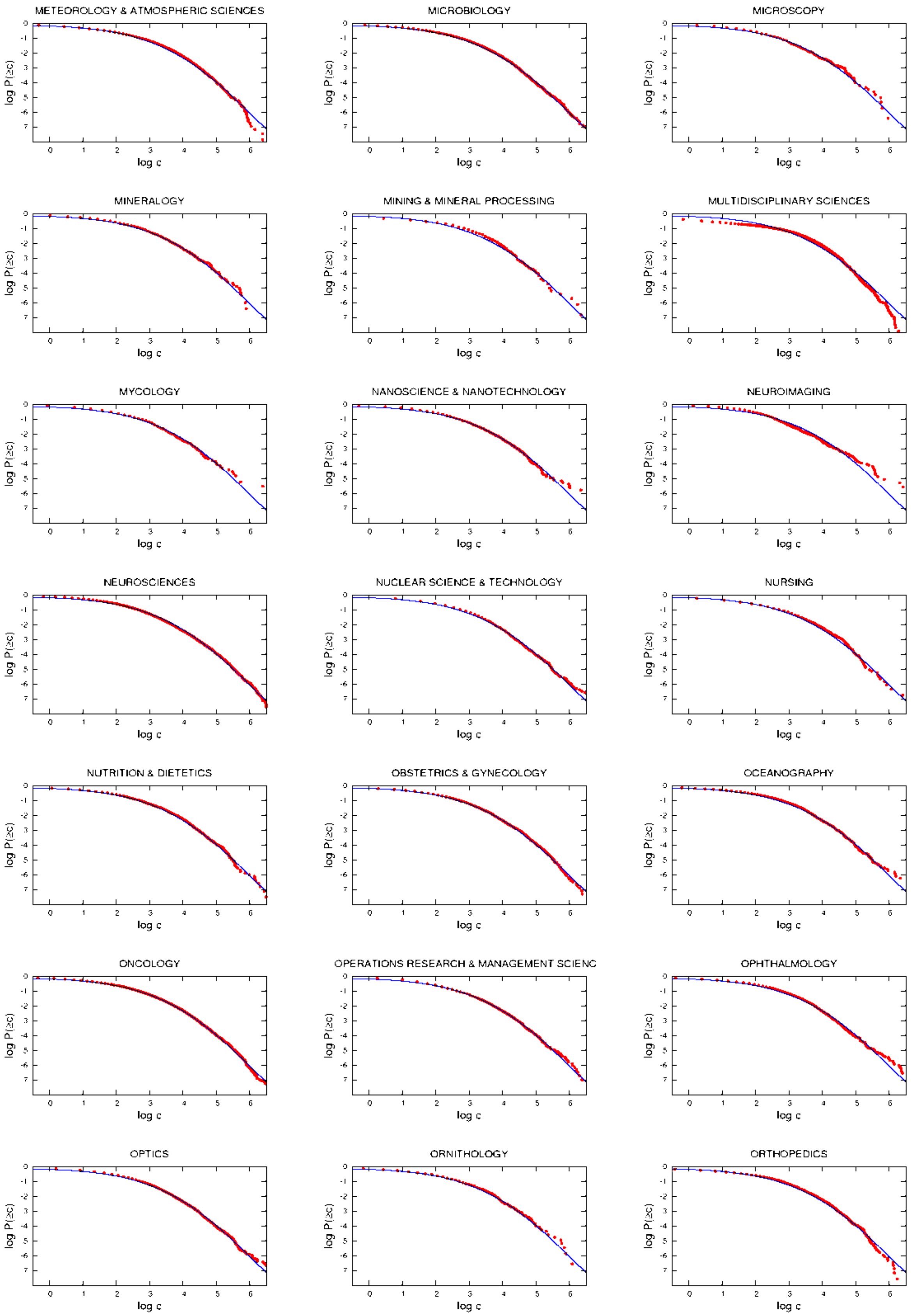

Figure S72: Publication year 1995.

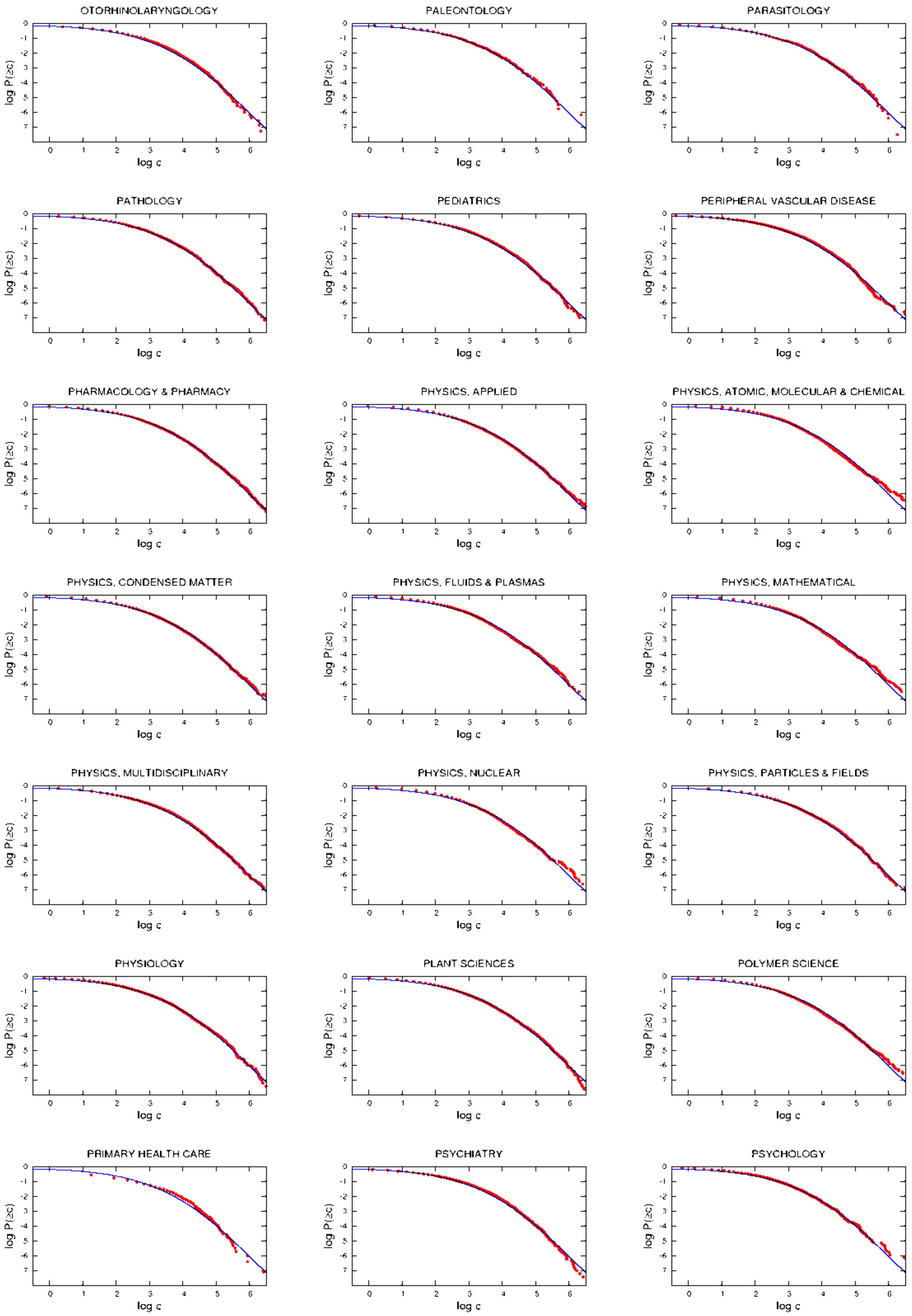

Figure S73: Publication year 1995.

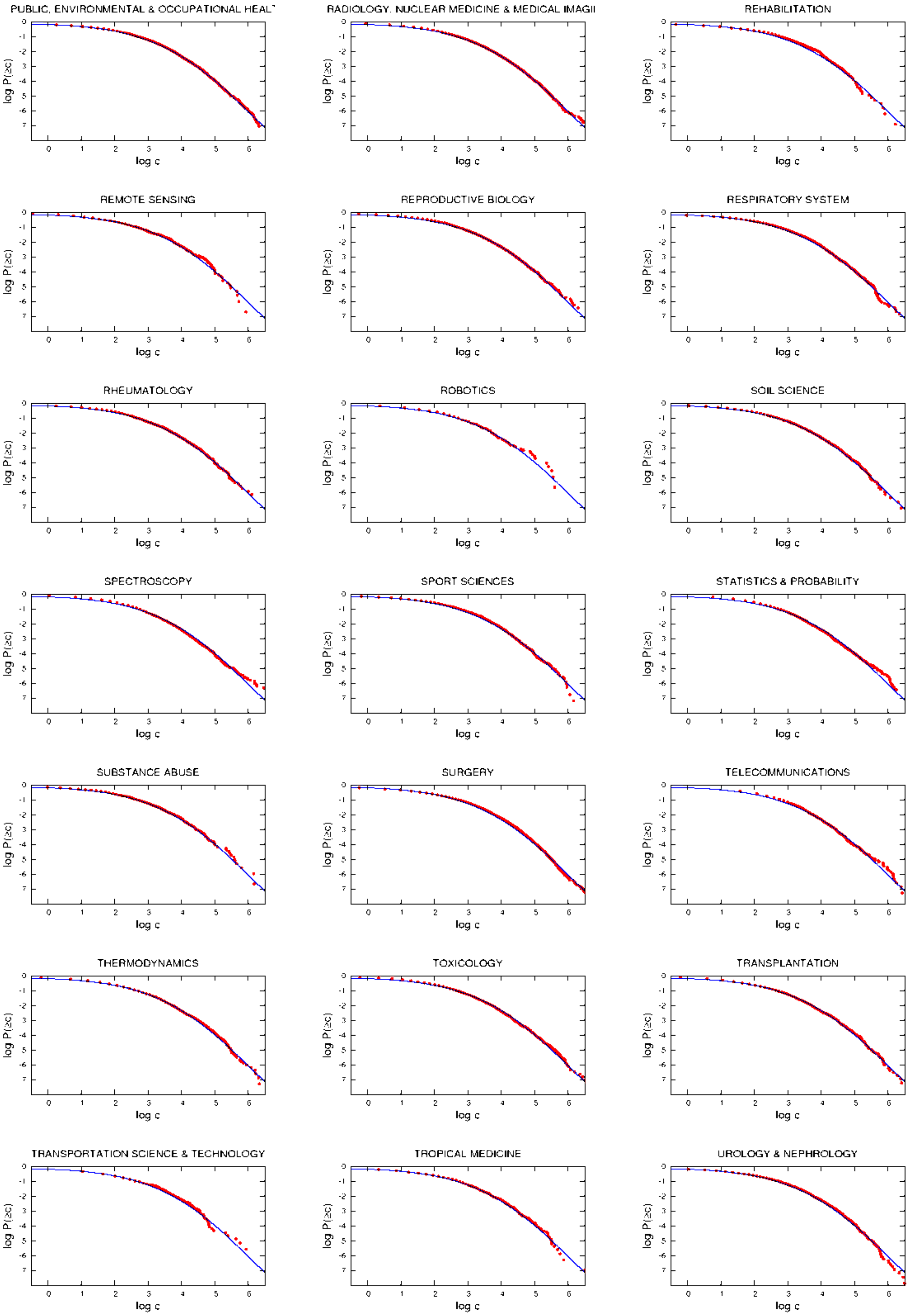

Figure S74: Publication year 1995.

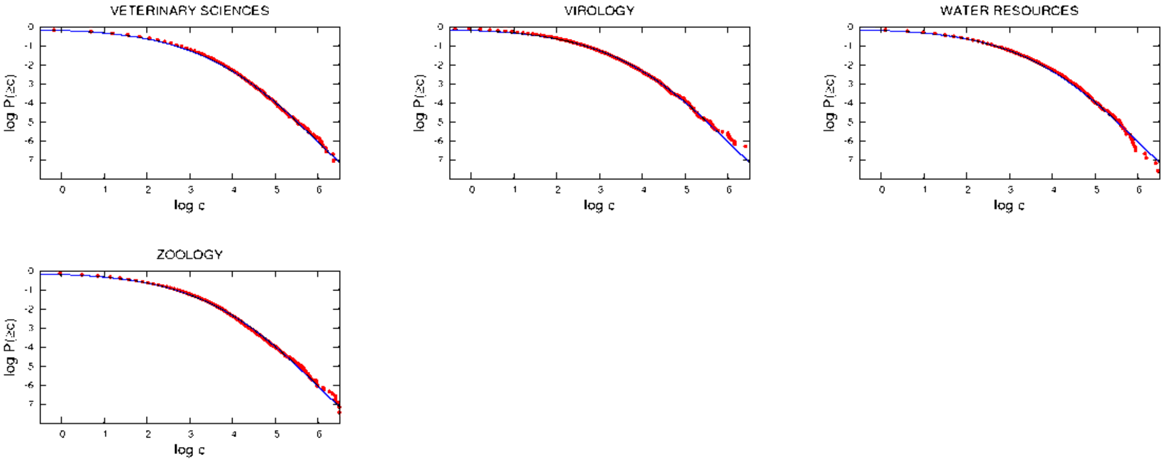

Figure S75: Publication year 1995.

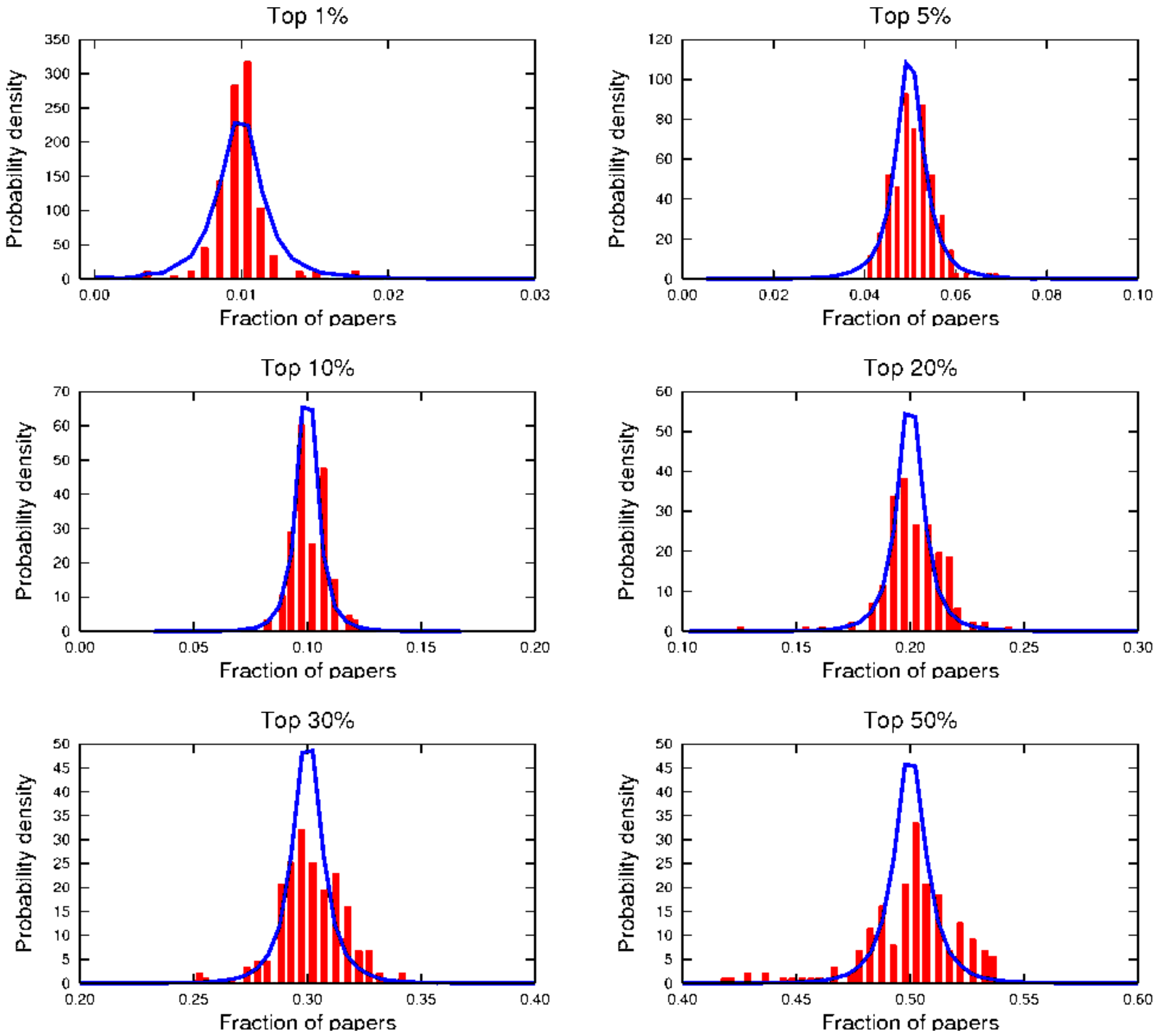

Figure S76: Publication year 1995.
