# Supplementary material for: A Reverse Engineering Approach to the Suppression of Citation Biases Reveals Universal Properties of Citation Distributions
Source: PLoS One. 2012 Mar 29;7(3):e33833. doi: 10.1371/journal.pone.0033833 (PMC3315498; doi:10.1371/journal.pone.0033833)
Supplement: Supporting Information S6 — Complete analysis for publication year . (PDF) [file pone.0033833.s006.pdf]

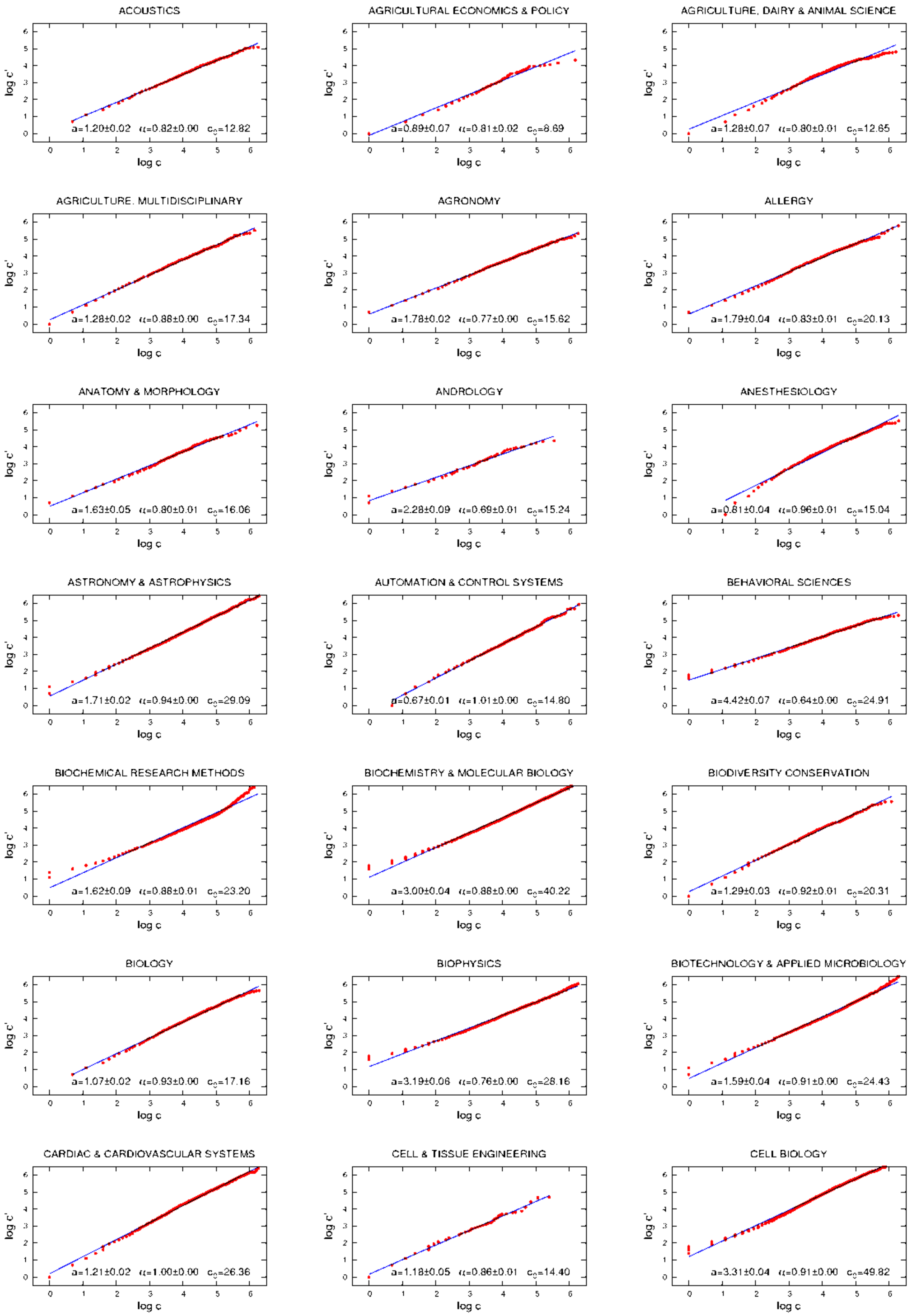

Figure S77: Publication year 1999.

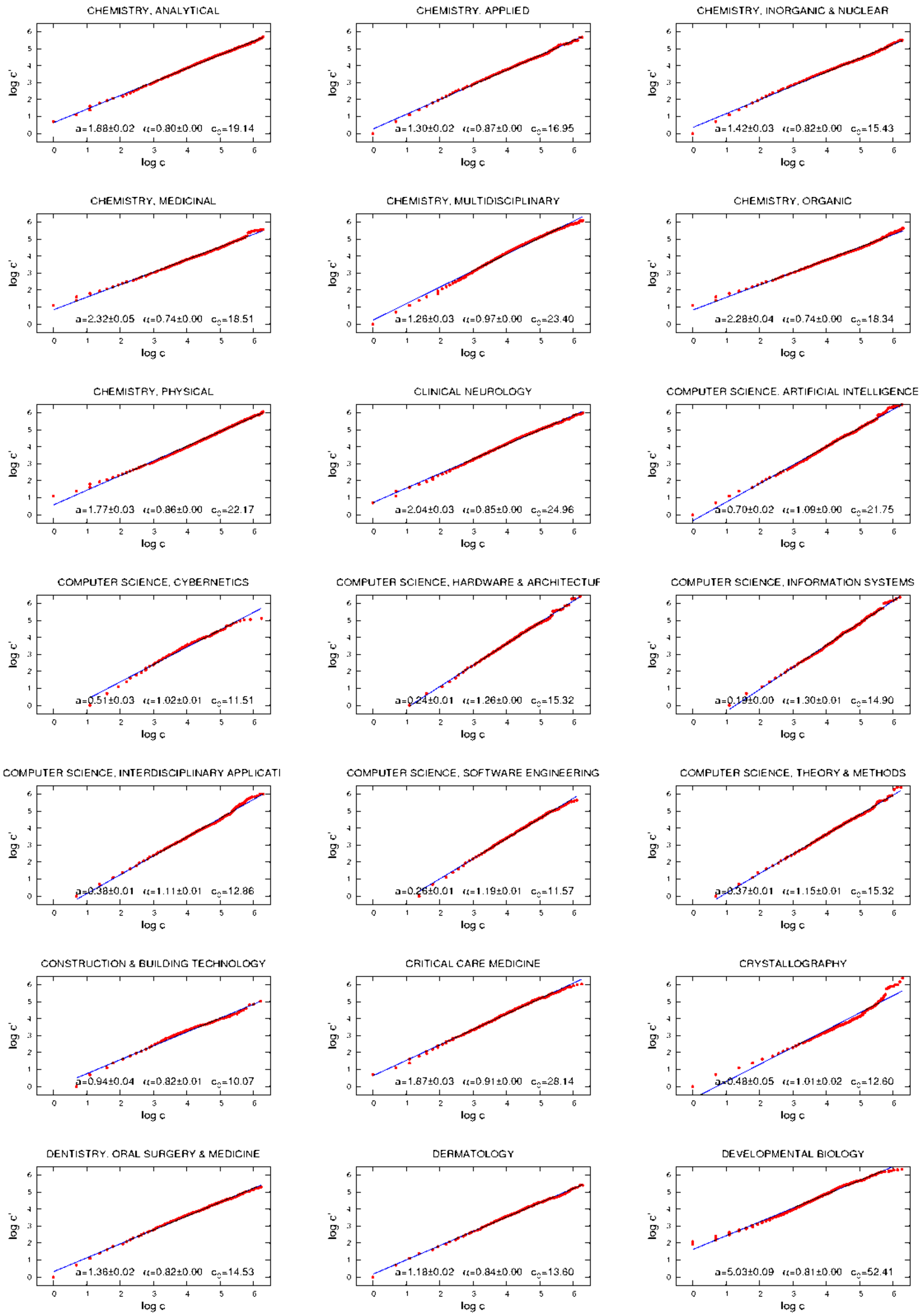

Figure S78: Publication year 1999.

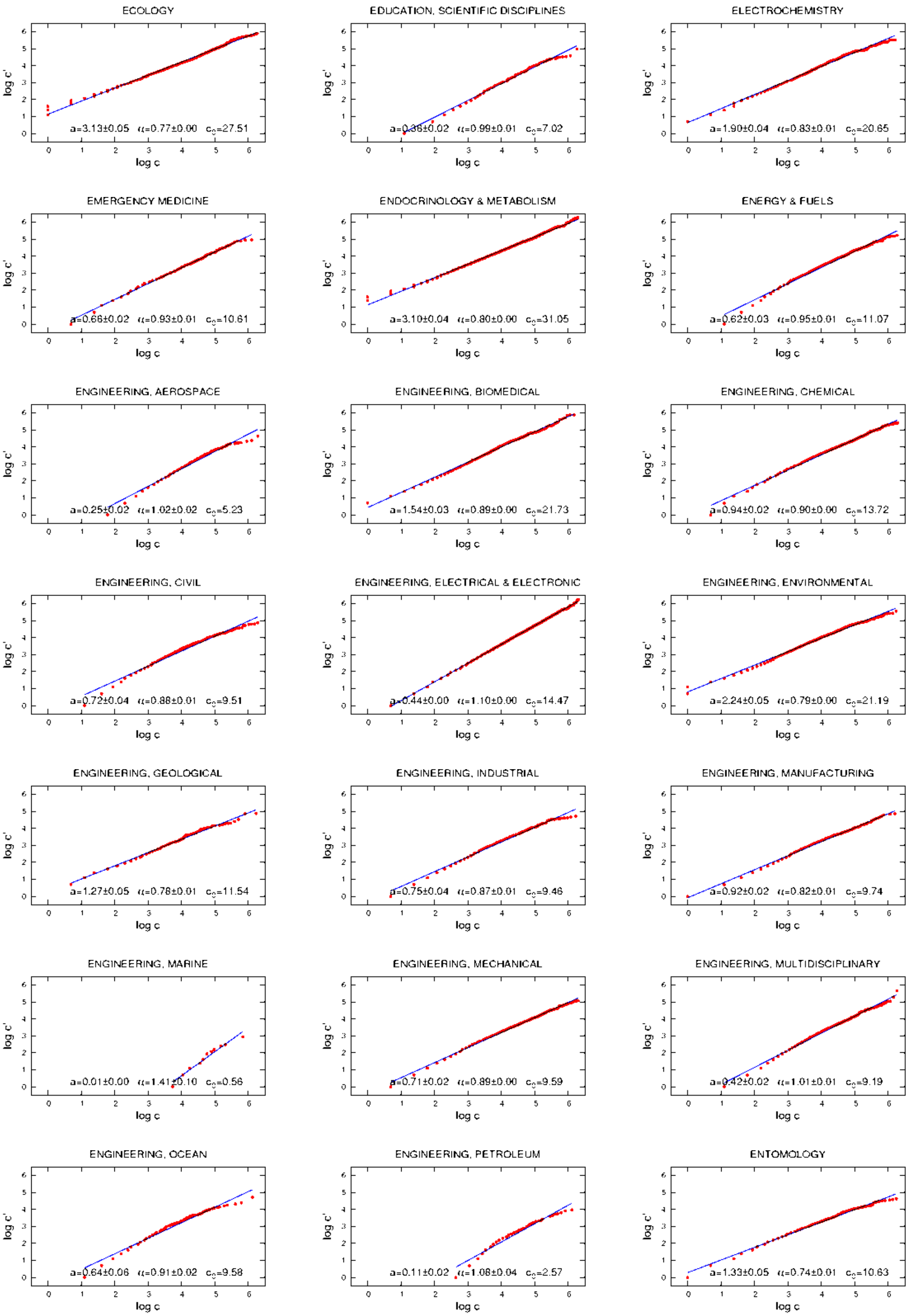

Figure S79: Publication year 1999.

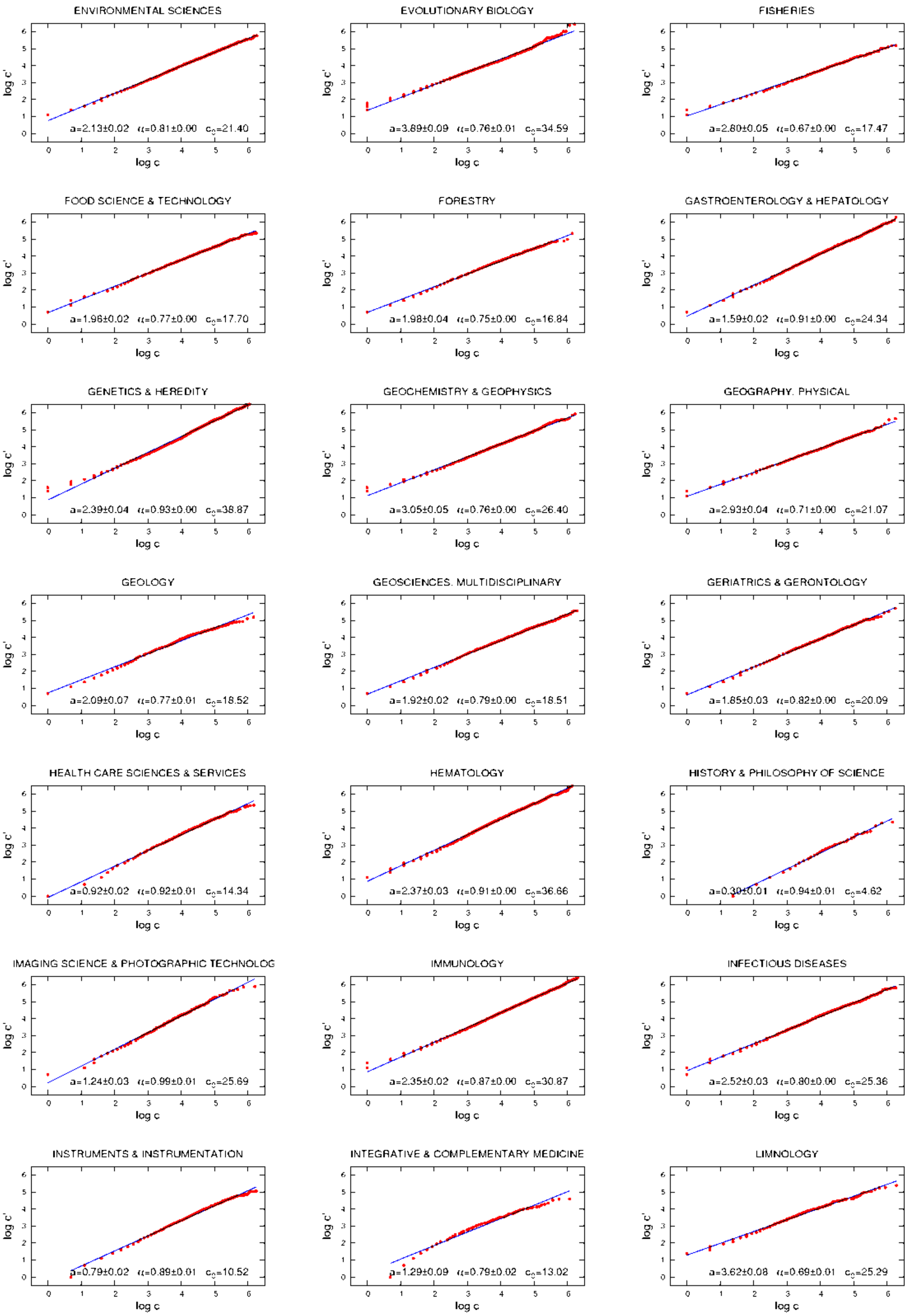

Figure S80: Publication year 1999.

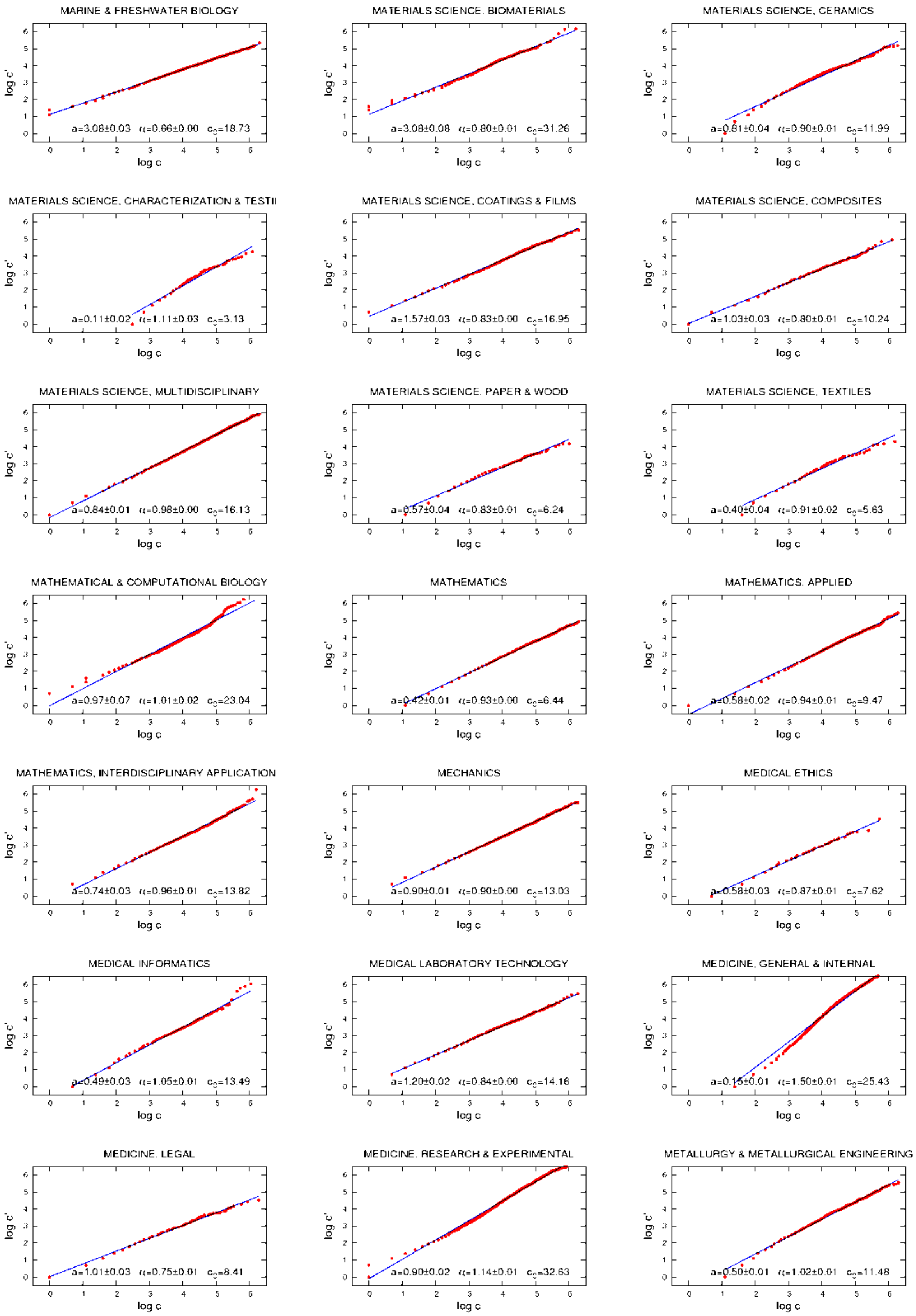

Figure S81: Publication year 1999.

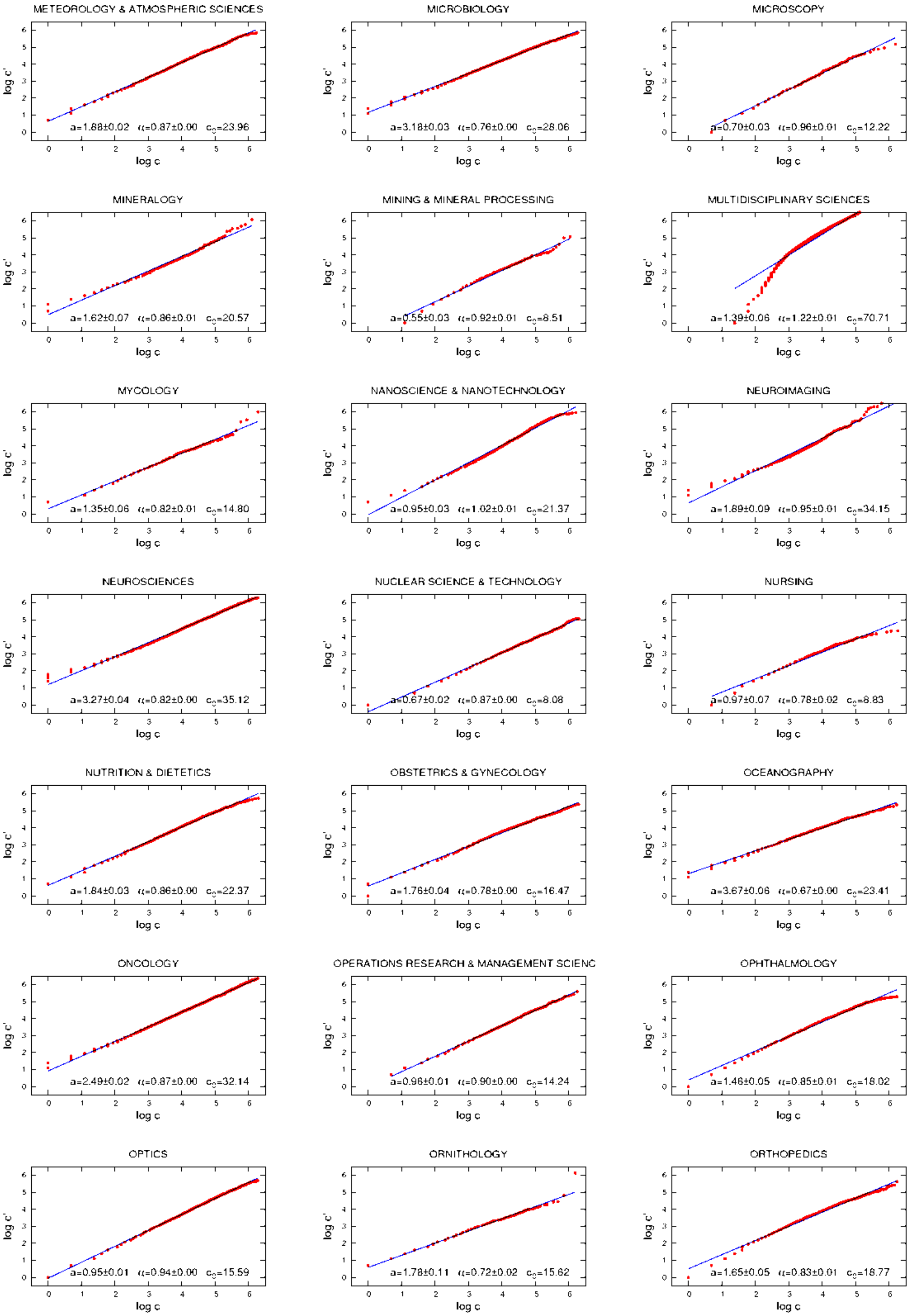

Figure S82: Publication year 1999.

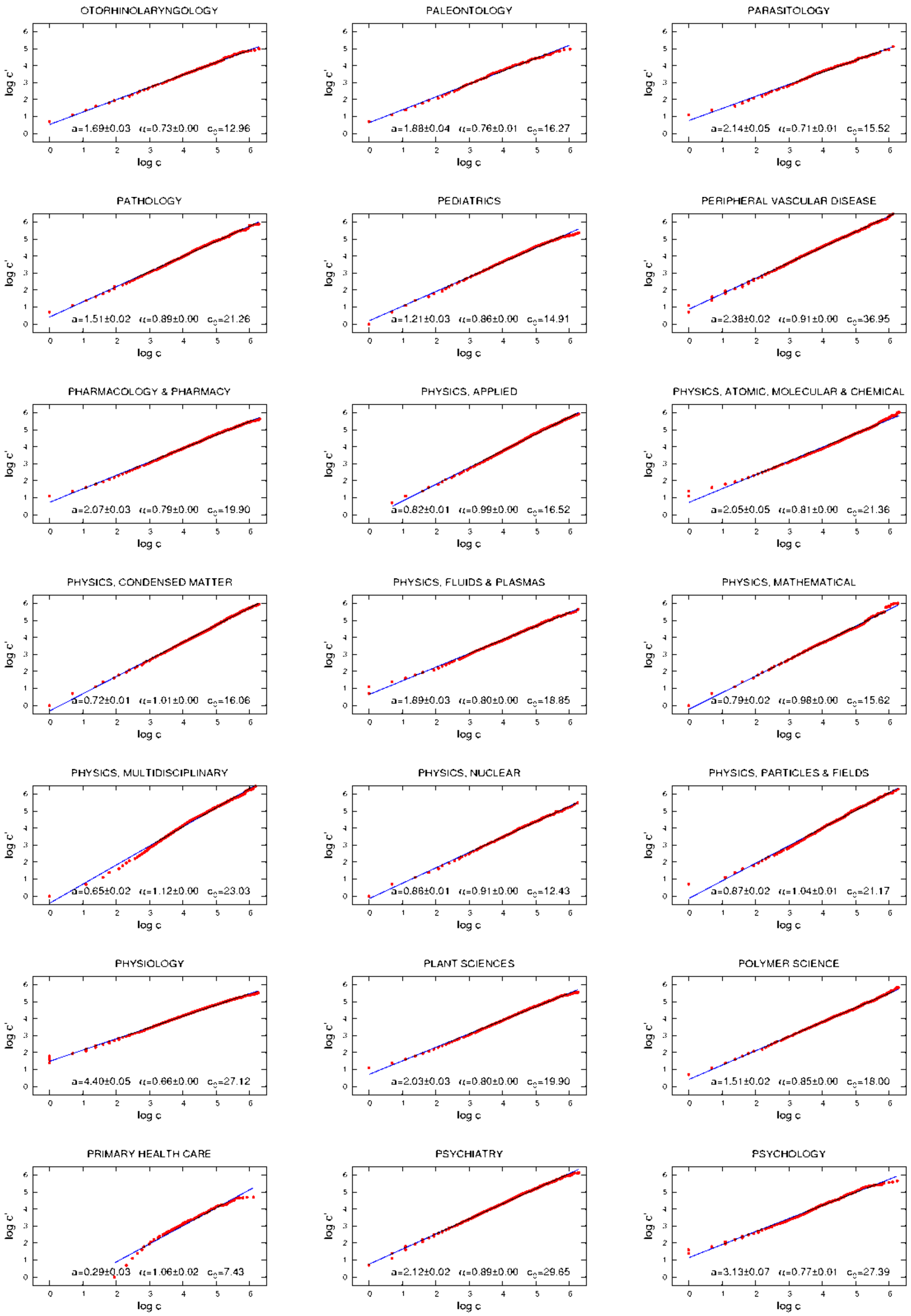

Figure S83: Publication year 1999.

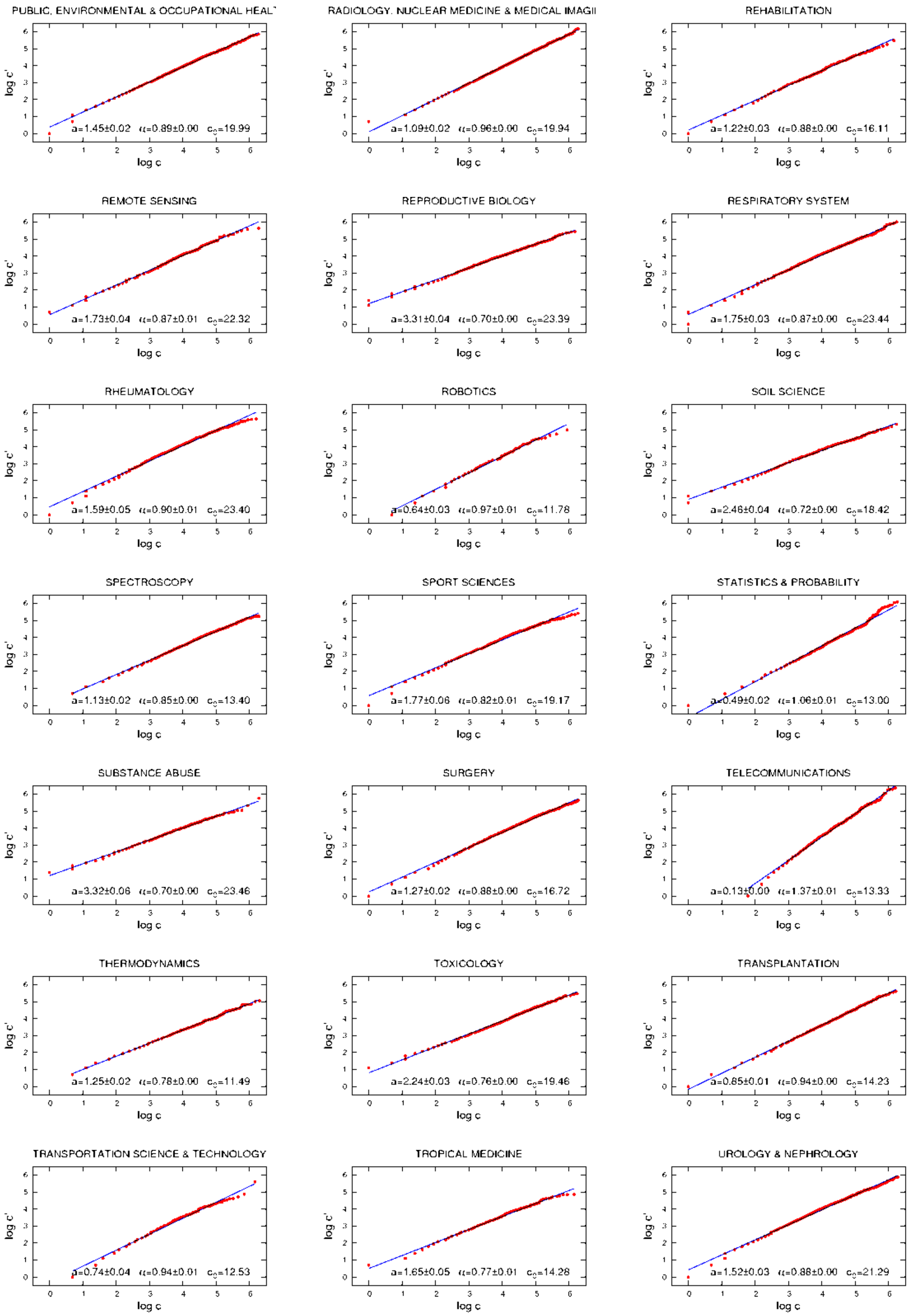

Figure S84: Publication year 1999.

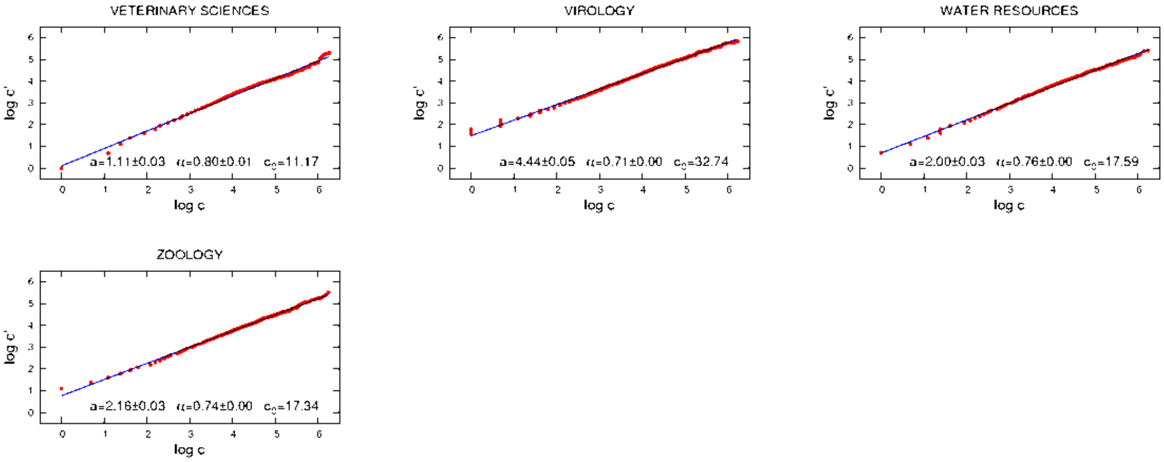

Figure S85: Publication year 1999.

Subject-category	a	α	$\langle c\rangle$	N
ACOUSTICS	1.20 ± 0.02	0.82 ± 0.00	12.82	2,670
AGRICULTURAL ECONOMICS & POLICY	0.89 ± 0.07	0.81 ± 0.02	8.69	750
AGRICULTURE, DAIRY & ANIMAL SCIENCE	1.28 ± 0.07	0.80 ± 0.01	12.65	3,202
AGRICULTURE, MULTIDISCIPLINARY	1.28 ± 0.02	0.88 ± 0.00	17.34	2,142
AGRONOMY	1.78 ± 0.02	0.77 ± 0.00	15.62	3,634
ALLERGY	1.79 ± 0.04	0.83 ± 0.01	20.13	1,918
ANATOMY & MORPHOLOGY	1.63 ± 0.05	0.80 ± 0.01	16.06	826
ANDROLOGY	2.28 ± 0.09	0.69 ± 0.01	15.24	195
ANESTHESIOLOGY	0.81 ± 0.04	0.96 ± 0.01	15.04	3,799
ASTRONOMY & ASTROPHYSICS	1.71 ± 0.02	0.94 ± 0.00	29.09	9,644
AUTOMATION & CONTROL SYSTEMS	0.67 ± 0.01	1.01 ± 0.00	14.80	2,886
BEHAVIORAL SCIENCES	4.42 ± 0.07	0.64 ± 0.00	24.91	2,792
BIOCHEMICAL RESEARCH METHODS	1.62 ± 0.09	0.88 ± 0.01	23.20	6,189
BIOCHEMISTRY & MOLECULAR BIOLOGY	3.00 ± 0.04	0.88 ± 0.00	40.22	37,591
BIODIVERSITY CONSERVATION	1.29 ± 0.03	0.92 ± 0.01	20.31	1,227
BIOLOGY	1.07 ± 0.02	0.93 ± 0.00	17.16	4,782
BIOPHYSICS	3.19 ± 0.06	0.76 ± 0.00	28.16	8,133
BIOTECHNOLOGY & APPLIED MICROBIOLOGY	1.59 ± 0.04	0.91 ± 0.00	24.43	11,328
CARDIAC & CARDIOVASCULAR SYSTEMS	1.21 ± 0.02	1.00 ± 0.00	26.36	11,097
CELL & TISSUE ENGINEERING	1.18 ± 0.05	0.86 ± 0.01	14.40	144
CELL BIOLOGY	3.31 ± 0.04	0.91 ± 0.00	49.82	16,484
CHEMISTRY, ANALYTICAL	1.88 ± 0.02	0.80 ± 0.00	19.14	11,698
CHEMISTRY, APPLIED	1.30 ± 0.02	0.87 ± 0.00	16.95	5,485
CHEMISTRY, INORGANIC & NUCLEAR	1.42 ± 0.03	0.82 ± 0.00	15.43	8,348
CHEMISTRY, MEDICINAL	2.32 ± 0.05	0.74 ± 0.00	18.51	4,742
CHEMISTRY, MULTIDISCIPLINARY	1.26 ± 0.03	0.97 ± 0.00	23.40	17,292
CHEMISTRY, ORGANIC	2.28 ± 0.04	0.74 ± 0.00	18.34	13,773
CHEMISTRY, PHYSICAL	1.77 ± 0.03	0.86 ± 0.00	22.17	22,527
CLINICAL NEUROLOGY	2.04 ± 0.03	0.85 ± 0.00	24.96	13,281
COMPUTER SCIENCE, ARTIFICIAL INTELLIGENCE	0.70 ± 0.02	1.09 ± 0.00	21.75	3,680
COMPUTER SCIENCE, CYBERNETICS	0.51 ± 0.03	1.02 ± 0.01	11.51	855
COMPUTER SCIENCE, HARDWARE & ARCHITECTURE	0.24 ± 0.01	1.26 ± 0.00	15.32	2,388
COMPUTER SCIENCE, INFORMATION SYSTEMS	0.19 ± 0.00	1.30 ± 0.01	14.90	3,185
COMPUTER SCIENCE, INTERDISCIPLINARY APPLICATIONS	0.38 ± 0.01	1.11 ± 0.01	12.86	4,712
COMPUTER SCIENCE, SOFTWARE ENGINEERING	0.26 ± 0.01	1.19 ± 0.01	11.57	3,251
COMPUTER SCIENCE, THEORY & METHODS	0.37 ± 0.01	1.15 ± 0.01	15.32	3,399
CONSTRUCTION & BUILDING TECHNOLOGY	0.94 ± 0.04	0.82 ± 0.01	10.07	1,603
CRITICAL CARE MEDICINE	1.87 ± 0.03	0.91 ± 0.00	28.14	2,659
CRYSTALLOGRAPHY	0.48 ± 0.05	1.01 ± 0.02	12.60	4,796
DENTISTRY, ORAL SURGERY & MEDICINE	1.36 ± 0.02	0.82 ± 0.00	14.53	4,196
DERMATOLOGY	1.18 ± 0.02	0.84 ± 0.00	13.60	4,720
DEVELOPMENTAL BIOLOGY	5.03 ± 0.09	0.81 ± 0.00	52.41	2,744
ECOLOGY	3.13 ± 0.05	0.77 ± 0.00	27.51	7,441
EDUCATION, SCIENTIFIC DISCIPLINES	0.36 ± 0.02	0.99 ± 0.01	7.02	1,823
ELECTROCHEMISTRY	1.90 ± 0.04	0.83 ± 0.01	20.65	4,052
EMERGENCY MEDICINE	0.66 ± 0.02	0.93 ± 0.01	10.61	1,290
ENDOCRINOLOGY & METABOLISM	3.10 ± 0.04	0.80 ± 0.00	31.05	9,803
ENERGY & FUELS	0.62 ± 0.03	0.95 ± 0.01	11.07	4,706
ENGINEERING, AEROSPACE	0.25 ± 0.02	1.02 ± 0.02	5.23	1,949
ENGINEERING, BIOMEDICAL	1.54 ± 0.03	0.89 ± 0.00	21.73	3,010

Table S29: Publication year 1999.

Subject-category	a	α	$\langle c \rangle$	N
ENGINEERING, CHEMICAL	0.94 ± 0.02	0.90 ± 0.00	13.72	10,693
ENGINEERING, CIVIL	0.72 ± 0.04	0.88 ± 0.01	9.51	3,847
ENGINEERING, ELECTRICAL & ELECTRONIC	0.44 ± 0.00	1.10 ± 0.00	14.47	22,985
ENGINEERING, ENVIRONMENTAL	2.24 ± 0.05	0.79 ± 0.00	21.19	3,449
ENGINEERING, GEOLOGICAL	1.27 ± 0.05	0.78 ± 0.01	11.54	848
ENGINEERING, INDUSTRIAL	0.75 ± 0.04	0.87 ± 0.01	9.46	2,491
ENGINEERING, MANUFACTURING	0.92 ± 0.02	0.82 ± 0.01	9.74	2,368
ENGINEERING, MARINE	0.01 ± 0.00	1.41 ± 0.10	0.56	367
ENGINEERING, MECHANICAL	0.71 ± 0.02	0.89 ± 0.00	9.59	6,959
ENGINEERING, MULTIDISCIPLINARY	0.42 ± 0.02	1.01 ± 0.01	9.19	3,627
ENGINEERING, OCEAN	0.64 ± 0.06	0.91 ± 0.02	9.58	676
ENGINEERING, PETROLEUM	0.11 ± 0.02	1.08 ± 0.04	2.57	1,314
ENTOMOLOGY	1.33 ± 0.05	0.74 ± 0.01	10.63	3,478
ENVIRONMENTAL SCIENCES	2.13 ± 0.02	0.81 ± 0.00	21.40	12,049
EVOLUTIONARY BIOLOGY	3.89 ± 0.09	0.76 ± 0.01	34.59	2,425
FISHERIES	2.80 ± 0.05	0.67 ± 0.00	17.47	2,740
FOOD SCIENCE & TECHNOLOGY	1.96 ± 0.02	0.77 ± 0.00	17.70	7,002
FORESTRY	1.98 ± 0.04	0.75 ± 0.00	16.84	2,093
GASTROENTEROLOGY & HEPATOLOGY	1.59 ± 0.02	0.91 ± 0.00	24.34	7,319
GENETICS & HEREDITY	2.39 ± 0.04	0.93 ± 0.00	38.87	11,085
GEOCHEMISTRY & GEOPHYSICS	3.05 ± 0.05	0.76 ± 0.00	26.40	4,212
GEOGRAPHY, PHYSICAL	2.93 ± 0.04	0.71 ± 0.00	21.07	1,768
GEOLOGY	2.09 ± 0.07	0.77 ± 0.01	18.52	1,483
GEOSCIENCES, MULTIDISCIPLINARY	1.92 ± 0.02	0.79 ± 0.00	18.51	7,569
GERIATRICS & GERONTOLOGY	1.85 ± 0.03	0.82 ± 0.00	20.09	1,742
HEALTH CARE SCIENCES & SERVICES	0.92 ± 0.02	0.92 ± 0.01	14.34	3,066
HEMATOLOGY	2.37 ± 0.03	0.91 ± 0.00	36.66	8,188
HISTORY & PHILOSOPHY OF SCIENCE	0.30 ± 0.01	0.94 ± 0.01	4.62	724
IMAGING SCIENCE & PHOTOGRAPHIC TECHNOLOGY	1.24 ± 0.03	0.99 ± 0.01	25.69	799
IMMUNOLOGY	2.35 ± 0.02	0.87 ± 0.00	30.87	16,919
INFECTIOUS DISEASES	2.52 ± 0.03	0.80 ± 0.00	25.36	6,365
INSTRUMENTS & INSTRUMENTATION	0.79 ± 0.02	0.89 ± 0.01	10.52	7,105
INTEGRATIVE & COMPLEMENTARY MEDICINE	1.29 ± 0.09	0.79 ± 0.02	13.02	599
LIMNOLOGY	3.62 ± 0.08	0.69 ± 0.01	25.29	948
MARINE & FRESHWATER BIOLOGY	3.08 ± 0.03	0.66 ± 0.00	18.73	5,977
MATERIALS SCIENCE, BIOMATERIALS	3.08 ± 0.08	0.80 ± 0.01	31.26	784
MATERIALS SCIENCE, CERAMICS	0.81 ± 0.04	0.90 ± 0.01	11.99	2,790
MATERIALS SCIENCE, CHARACTERIZATION & TESTING	0.11 ± 0.02	1.11 ± 0.03	3.13	1,254
MATERIALS SCIENCE, COATINGS & FILMS	1.57 ± 0.03	0.83 ± 0.00	16.95	3,808
MATERIALS SCIENCE, COMPOSITES	1.03 ± 0.03	0.80 ± 0.01	10.24	1,305
MATERIALS SCIENCE, MULTIDISCIPLINARY	0.84 ± 0.01	0.98 ± 0.00	16.13	23,804
MATERIALS SCIENCE, PAPER & WOOD	0.57 ± 0.04	0.83 ± 0.01	6.24	1,064
MATERIALS SCIENCE, TEXTILES	0.40 ± 0.04	0.91 ± 0.02	5.63	760
MATHEMATICAL & COMPUTATIONAL BIOLOGY	0.97 ± 0.07	1.01 ± 0.02	23.04	1,436
MATHEMATICS	0.42 ± 0.01	0.93 ± 0.00	6.44	11,458
MATHEMATICS, APPLIED	0.58 ± 0.02	0.94 ± 0.01	9.47	8,708
MATHEMATICS, INTERDISCIPLINARY APPLICATIONS	0.74 ± 0.03	0.96 ± 0.01	13.82	3,195
MECHANICS	0.90 ± 0.01	0.90 ± 0.00	13.03	7,338
MEDICAL ETHICS	0.58 ± 0.03	0.87 ± 0.01	7.62	285
MEDICAL INFORMATICS	0.49 ± 0.03	1.05 ± 0.01	13.49	1,159

Table S30: Publication year 1999.

Subject-category	a	α	$\langle c \rangle$	N
MEDICAL LABORATORY TECHNOLOGY	1.20 ± 0.02	0.84 ± 0.00	14.16	1,849
MEDICINE, GENERAL & INTERNAL	0.15 ± 0.01	1.50 ± 0.01	25.43	15,478
MEDICINE, LEGAL	1.01 ± 0.03	0.75 ± 0.01	8.41	928
MEDICINE, RESEARCH & EXPERIMENTAL	0.90 ± 0.02	1.14 ± 0.01	32.63	7,902
METALLURGY & METALLURGICAL ENGINEERING	0.50 ± 0.01	1.02 ± 0.01	11.48	4,936
METEOROLOGY & ATMOSPHERIC SCIENCES	1.88 ± 0.02	0.87 ± 0.00	23.96	5,099
MICROBIOLOGY	3.18 ± 0.03	0.76 ± 0.00	28.06	11,077
MICROSCOPY	0.70 ± 0.03	0.96 ± 0.01	12.21	772
MINERALOGY	1.62 ± 0.07	0.86 ± 0.01	20.57	1,294
MINING & MINERAL PROCESSING	0.55 ± 0.03	0.92 ± 0.01	8.51	1,145
MULTIDISCIPLINARY SCIENCES	1.39 ± 0.06	1.22 ± 0.01	70.71	10,958
MYCOLOGY	1.35 ± 0.06	0.82 ± 0.01	14.80	950
NANOSCIENCE & NANOTECHNOLOGY	0.95 ± 0.03	1.02 ± 0.01	21.37	3,219
NEUROIMAGING	1.89 ± 0.09	0.95 ± 0.01	34.15	968
NEUROSCIENCES	3.27 ± 0.04	0.82 ± 0.00	35.12	21,227
NUCLEAR SCIENCE & TECHNOLOGY	0.67 ± 0.02	0.87 ± 0.00	8.08	6,942
NURSING	0.97 ± 0.07	0.78 ± 0.02	8.83	1,804
NUTRITION & DIETETICS	1.84 ± 0.03	0.86 ± 0.00	22.37	3,938
OBSTETRICS & GYNECOLOGY	1.76 ± 0.04	0.78 ± 0.00	16.47	5,909
OCEANOGRAPHY	3.67 ± 0.06	0.67 ± 0.00	23.41	3,383
ONCOLOGY	2.49 ± 0.02	0.87 ± 0.00	32.14	16,269
OPERATIONS RESEARCH & MANAGEMENT SCIENCE	0.96 ± 0.01	0.90 ± 0.00	14.24	3,542
OPHTHALMOLOGY	1.46 ± 0.05	0.85 ± 0.01	18.02	5,093
OPTICS	0.95 ± 0.01	0.94 ± 0.00	15.59	10,311
ORNITHOLOGY	1.78 ± 0.11	0.72 ± 0.02	15.62	776
ORTHOPEDICS	1.65 ± 0.05	0.83 ± 0.01	18.77	4,309
OTORHINOLARYNGOLOGY	1.69 ± 0.03	0.73 ± 0.00	12.96	2,855
PALEONTOLOGY	1.88 ± 0.04	0.76 ± 0.01	16.27	1,103
PARASITOLOGY	2.14 ± 0.05	0.71 ± 0.01	15.52	2,020
PATHOLOGY	1.51 ± 0.02	0.89 ± 0.00	21.26	5,664
PEDIATRICS	1.21 ± 0.03	0.86 ± 0.00	14.91	8,609
PERIPHERAL VASCULAR DISEASE	2.38 ± 0.02	0.91 ± 0.00	36.95	7,303
PHARMACOLOGY & PHARMACY	2.07 ± 0.03	0.79 ± 0.00	19.90	18,956
PHYSICS, APPLIED	0.82 ± 0.01	0.99 ± 0.00	16.52	22,306
PHYSICS, ATOMIC, MOLECULAR & CHEMICAL	2.05 ± 0.05	0.81 ± 0.00	21.36	11,829
PHYSICS, CONDENSED MATTER	0.72 ± 0.01	1.01 ± 0.00	16.06	19,149
PHYSICS, FLUIDS & PLASMAS	1.89 ± 0.03	0.80 ± 0.00	18.85	4,626
PHYSICS, MATHEMATICAL	0.79 ± 0.02	0.98 ± 0.00	15.62	5,471
PHYSICS, MULTIDISCIPLINARY	0.65 ± 0.02	1.12 ± 0.00	23.03	12,198
PHYSICS, NUCLEAR	0.86 ± 0.01	0.91 ± 0.00	12.43	4,333
PHYSICS, PARTICLES & FIELDS	0.87 ± 0.02	1.04 ± 0.01	21.17	6,450
PHYSIOLOGY	4.40 ± 0.05	0.66 ± 0.00	27.12	7,333
PLANT SCIENCES	2.03 ± 0.03	0.80 ± 0.00	19.90	11,714
POLYMER SCIENCE	1.51 ± 0.02	0.85 ± 0.00	18.00	9,425
PRIMARY HEALTH CARE	0.29 ± 0.03	1.06 ± 0.02	7.43	1,341
PSYCHIATRY	2.12 ± 0.02	0.89 ± 0.00	29.65	7,804
PSYCHOLOGY	3.13 ± 0.07	0.77 ± 0.01	27.39	2,604
PUBLIC, ENVIRONMENTAL & OCCUPATIONAL HEALTH	1.45 ± 0.02	0.89 ± 0.00	19.99	8,439
RADIOLOGY, NUCLEAR MEDICINE & MEDICAL IMAGING	1.09 ± 0.02	0.96 ± 0.00	19.94	10,455
REHABILITATION	1.22 ± 0.03	0.88 ± 0.00	16.11	1,444

Table S31: Publication year 1999.

Subject-category	a	α	$\langle c \rangle$	N
REMOTE SENSING	1.73 ± 0.04	0.87 ± 0.01	22.32	934
REPRODUCTIVE BIOLOGY	3.31 ± 0.04	0.70 ± 0.00	23.39	3,054
RESPIRATORY SYSTEM	1.75 ± 0.03	0.87 ± 0.00	23.44	6,073
RHEUMATOLOGY	1.59 ± 0.05	0.90 ± 0.01	23.40	2,381
ROBOTICS	0.64 ± 0.03	0.97 ± 0.01	11.78	460
SOIL SCIENCE	2.46 ± 0.04	0.72 ± 0.00	18.42	2,551
SPECTROSCOPY	1.13 ± 0.02	0.85 ± 0.00	13.40	5,804
SPORT SCIENCES	1.77 ± 0.06	0.82 ± 0.01	19.17	3,737
STATISTICS & PROBABILITY	0.49 ± 0.02	1.06 ± 0.01	13.00	4,519
SUBSTANCE ABUSE	3.32 ± 0.06	0.70 ± 0.00	23.46	952
SURGERY	1.27 ± 0.02	0.88 ± 0.00	16.72	19,737
TELECOMMUNICATIONS	0.13 ± 0.00	1.37 ± 0.01	13.33	3,927
THERMODYNAMICS	1.25 ± 0.02	0.78 ± 0.00	11.49	2,956
TOXICOLOGY	2.24 ± 0.03	0.76 ± 0.00	19.46	5,205
TRANSPLANTATION	0.85 ± 0.01	0.94 ± 0.00	14.23	4,108
TRANSPORTATION SCIENCE & TECHNOLOGY	0.74 ± 0.04	0.94 ± 0.01	12.53	736
TROPICAL MEDICINE	1.65 ± 0.05	0.77 ± 0.01	14.28	1,429
UROLOGY & NEPHROLOGY	1.52 ± 0.03	0.88 ± 0.00	21.29	6,490
VETERINARY SCIENCES	1.11 ± 0.03	0.80 ± 0.01	11.17	6,331
VIROLOGY	4.44 ± 0.05	0.71 ± 0.00	32.74	4,158
WATER RESOURCES	2.00 ± 0.03	0.76 ± 0.00	17.59	4,265
ZOOLOGY	2.16 ± 0.03	0.74 ± 0.00	17.34	5,246
**TOTAL**	**1**.00 ± 0.00	**1**.00 ± 0.00	**2**1.97	**9**87,830

Table S32: Publication year 1999.

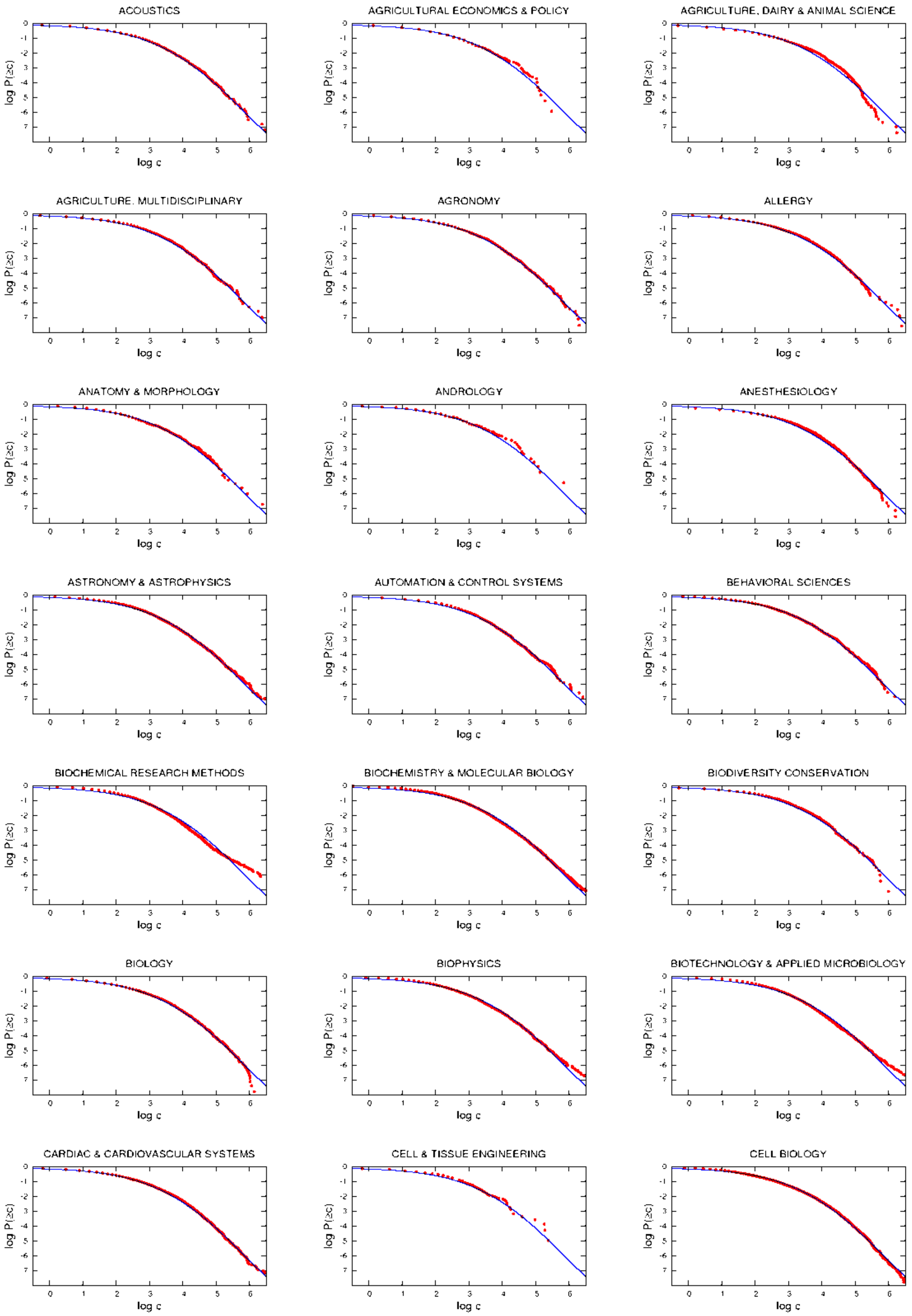

Figure S86: Publication year 1999.

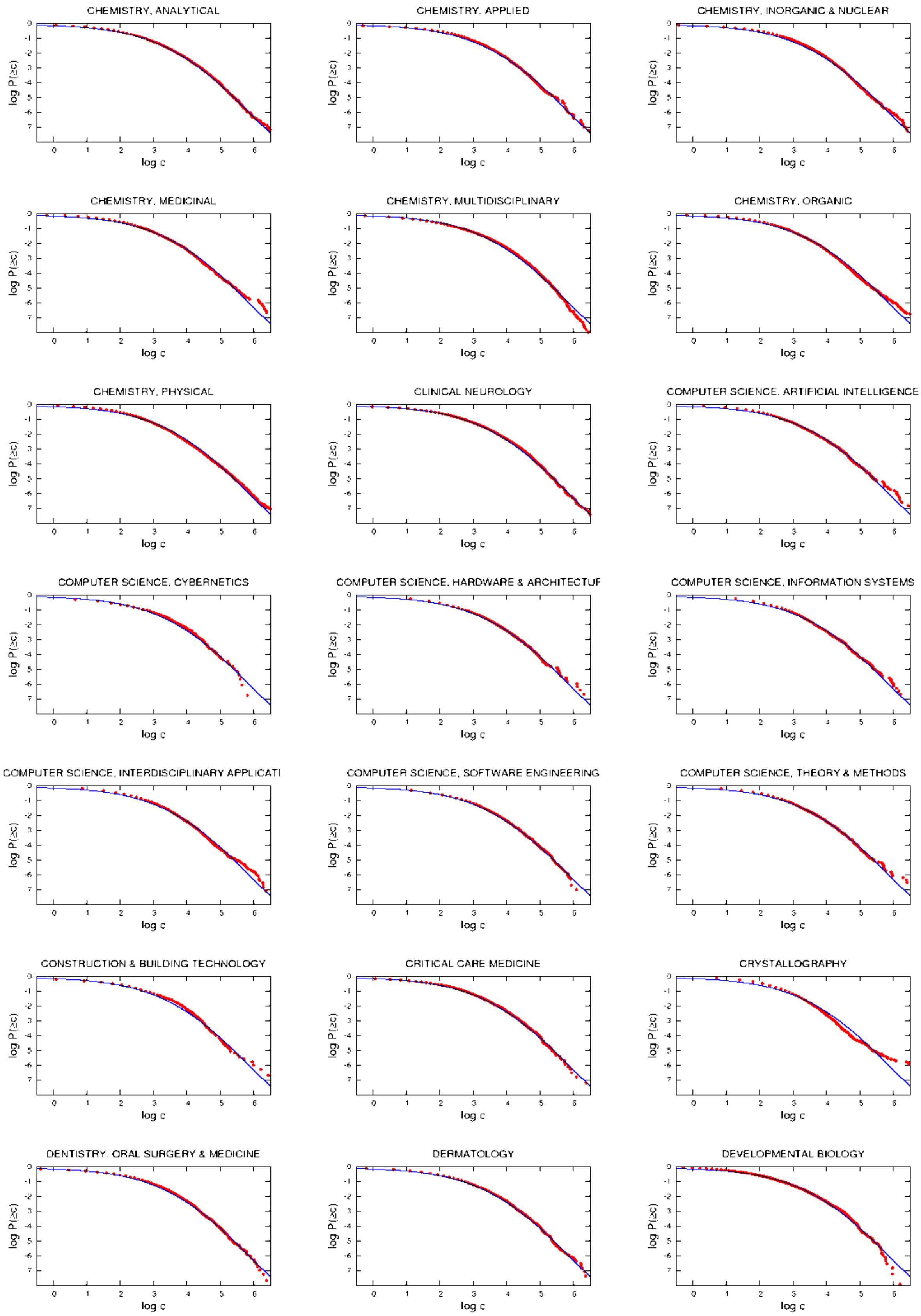

Figure S87: Publication year 1999.

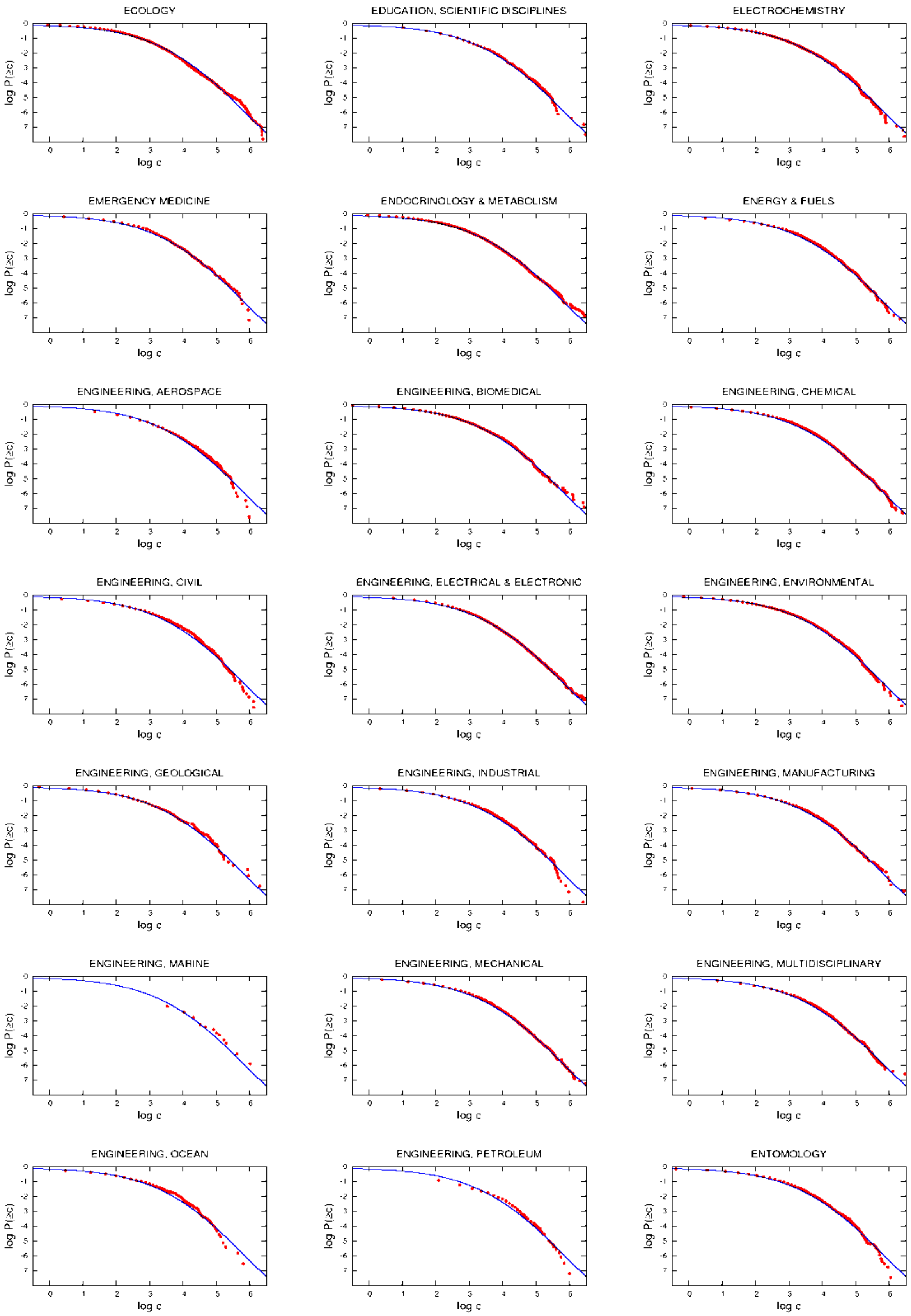

Figure S88: Publication year 1999.

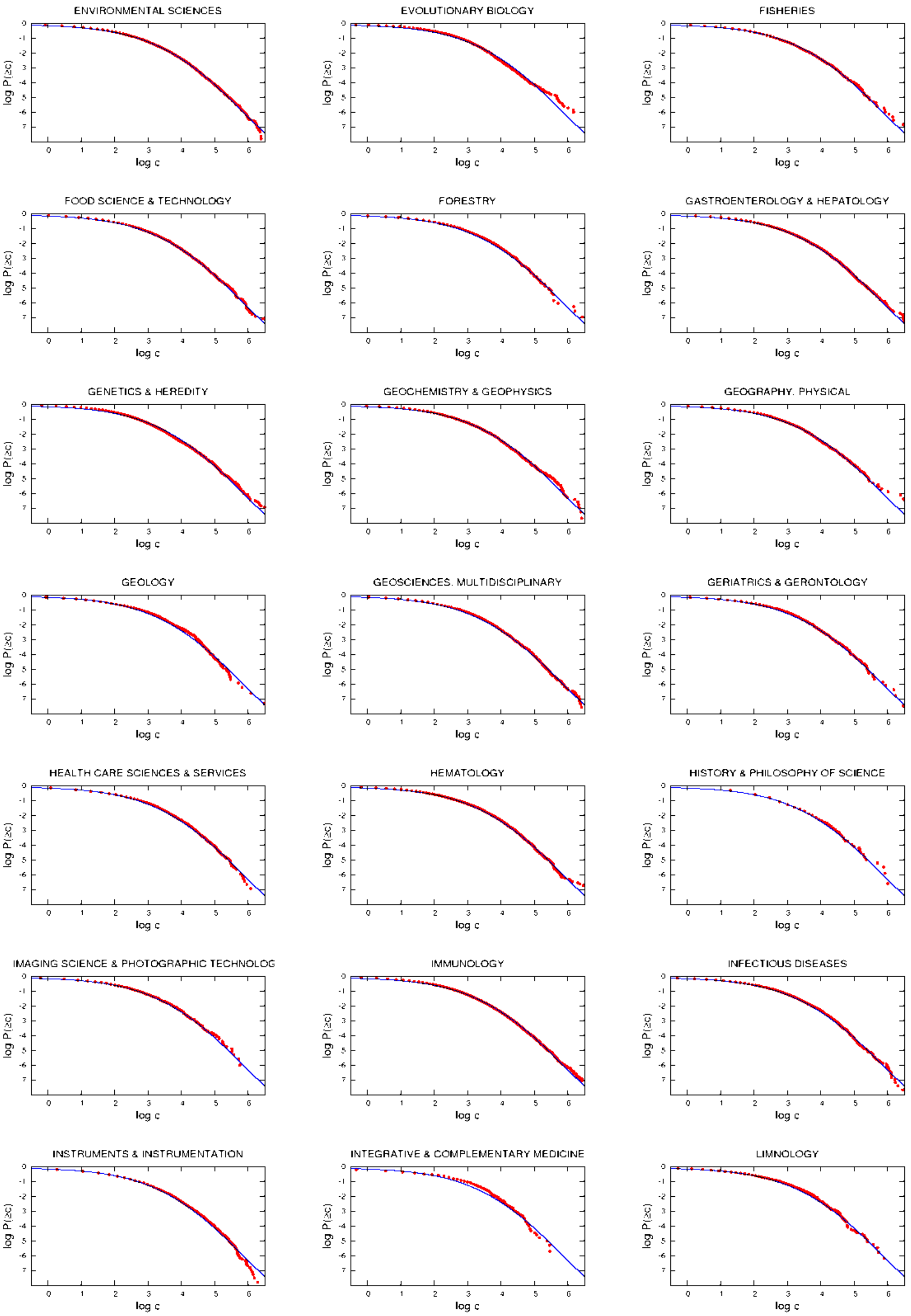

Figure S89: Publication year 1999.

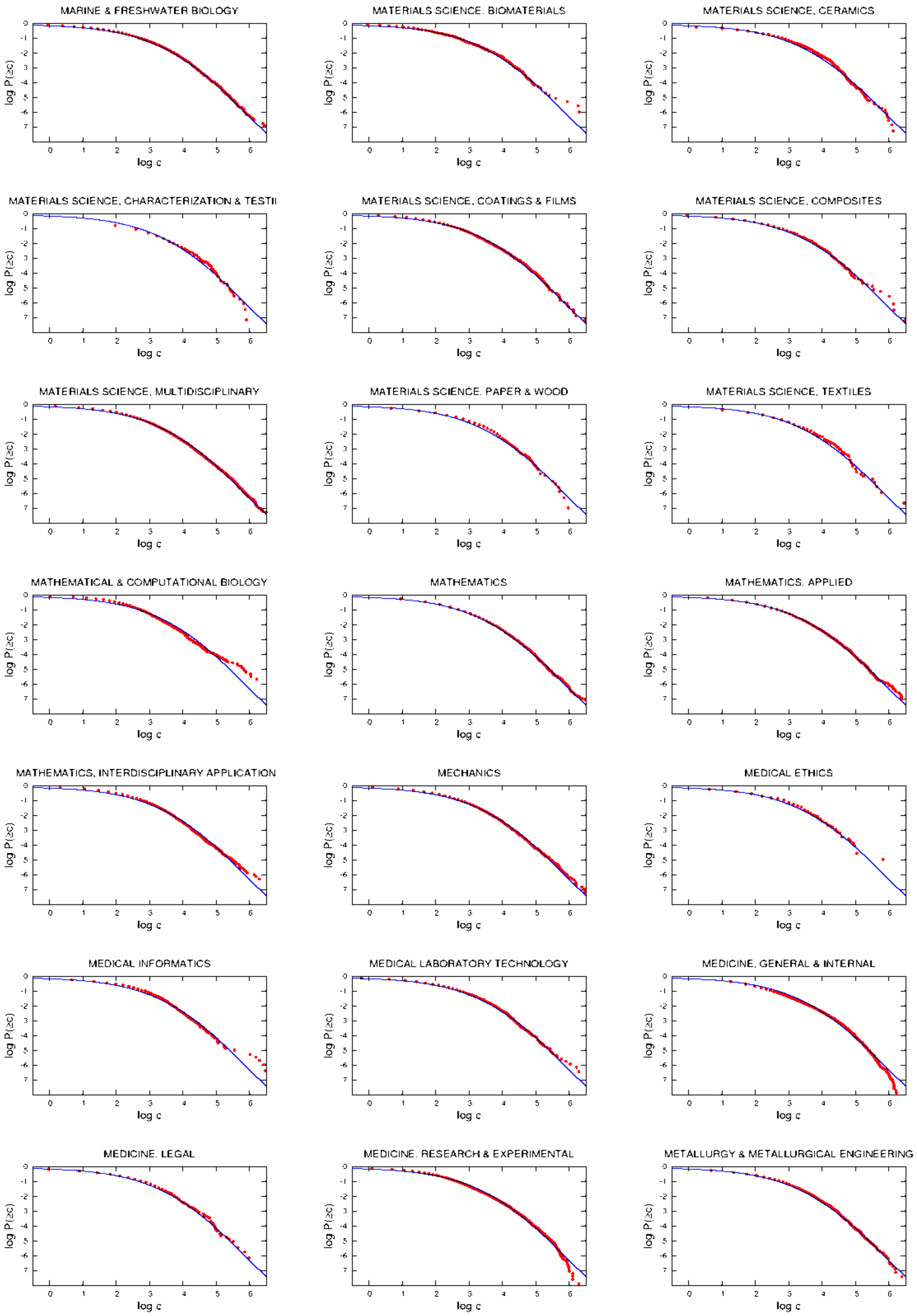

Figure S90: Publication year 1999.

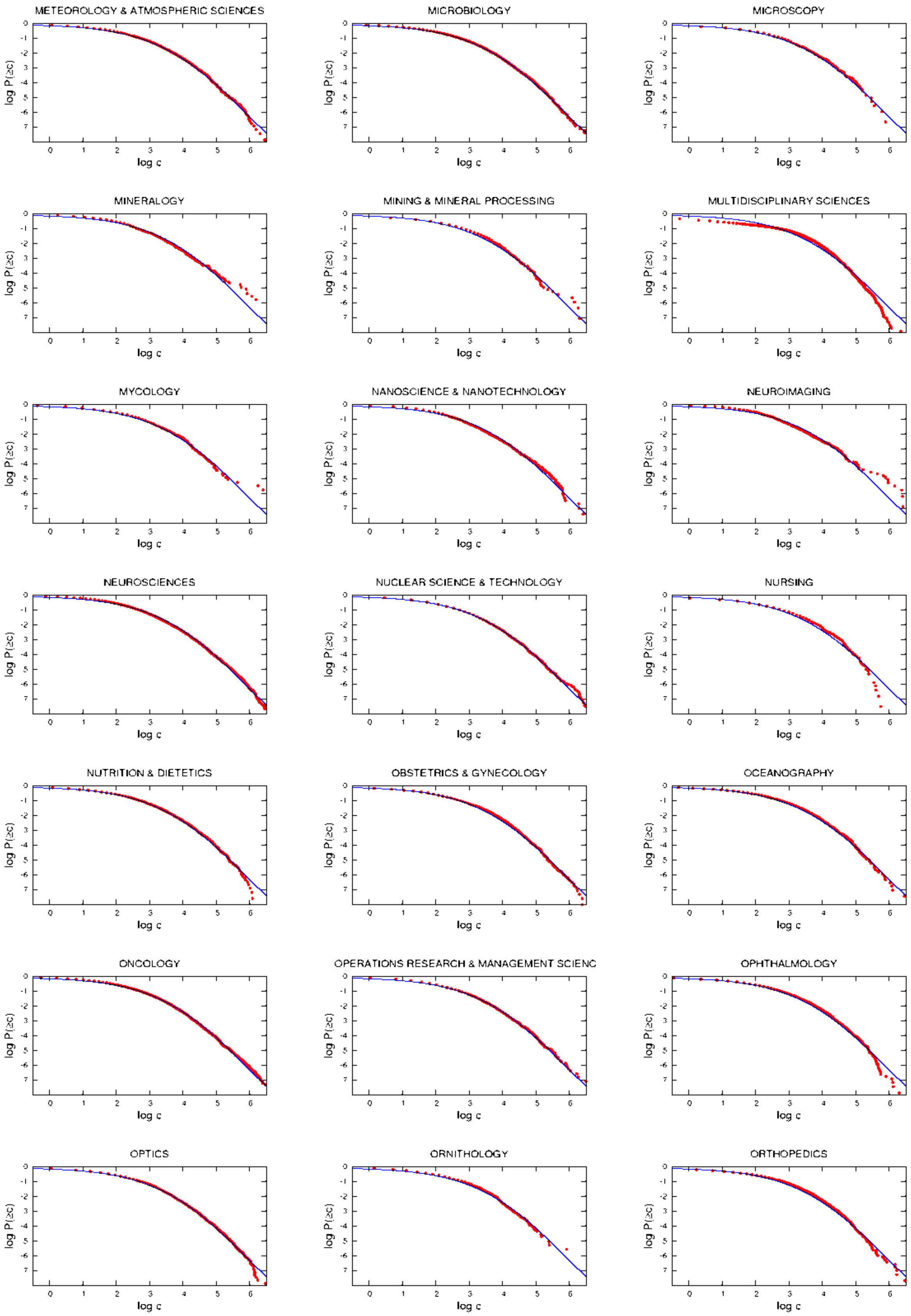

Figure S91: Publication year 1999.

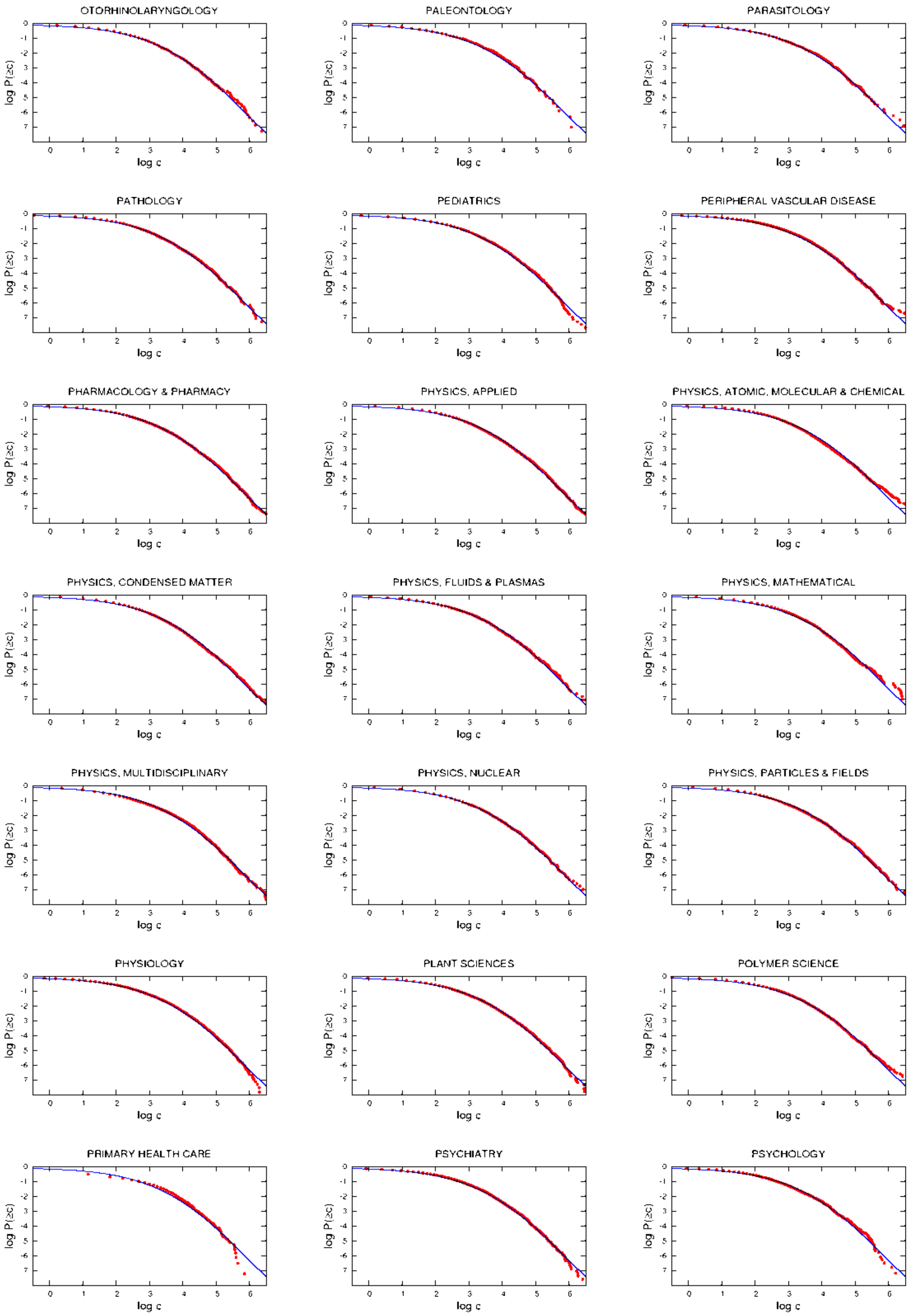

Figure S92: Publication year 1999.

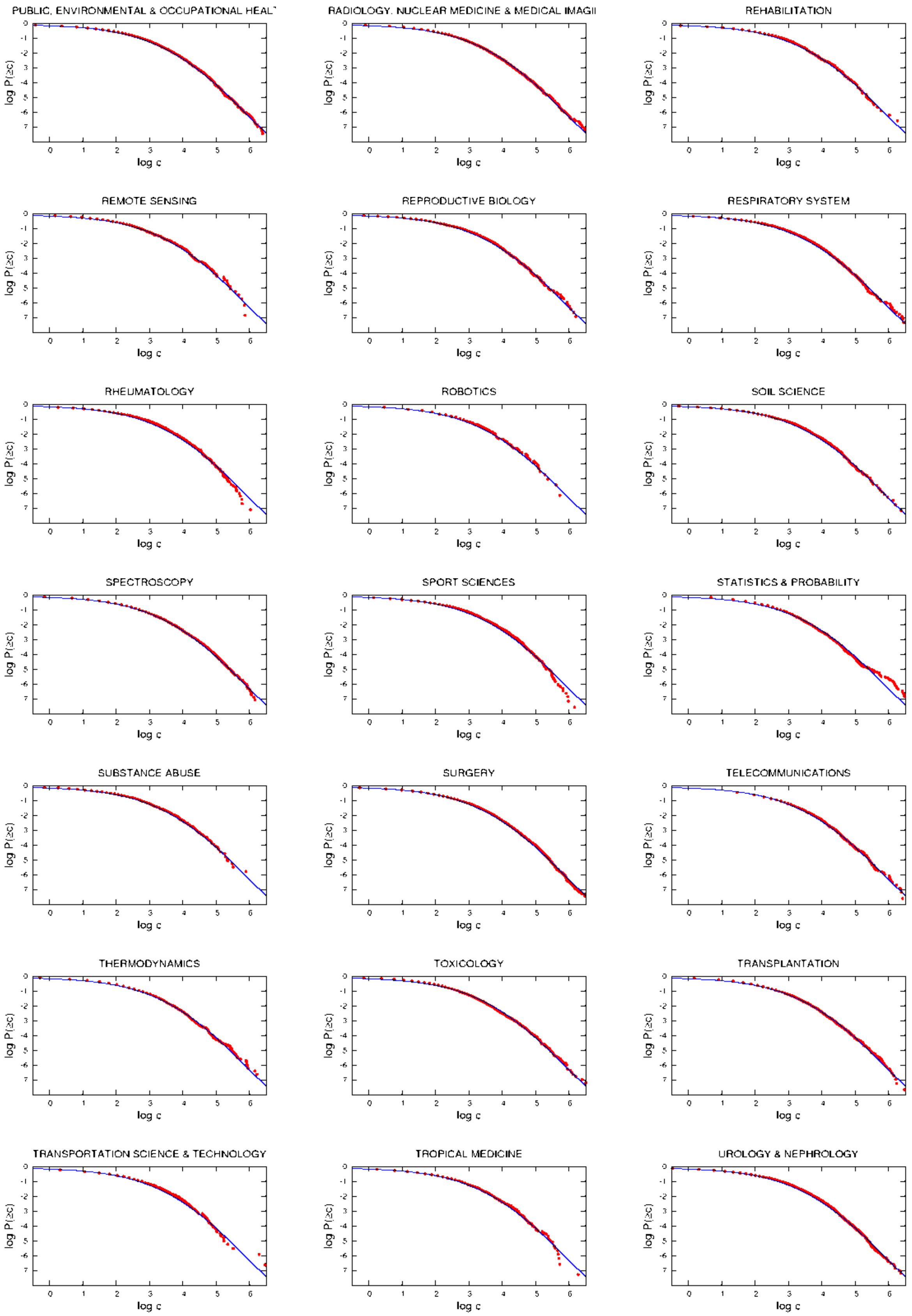

Figure S93: Publication year 1999.

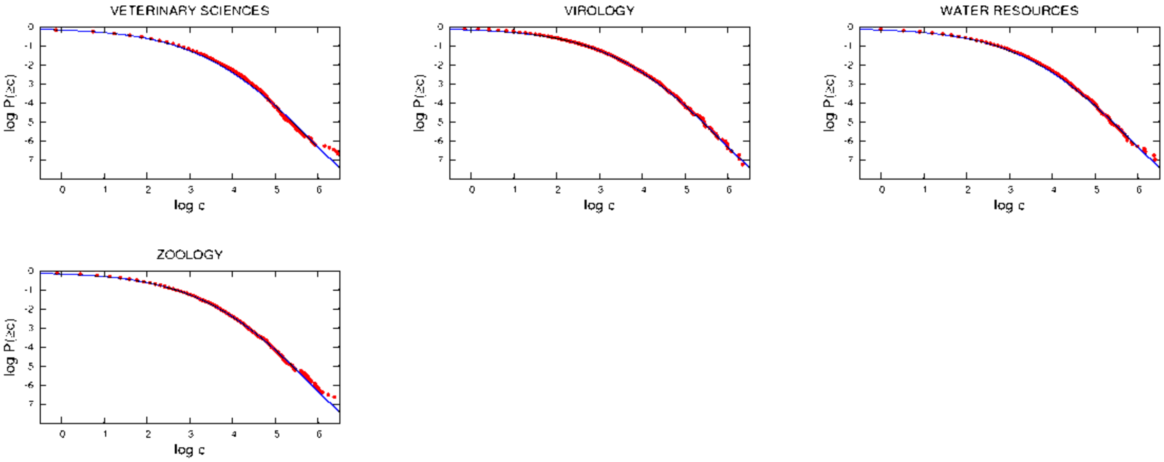

Figure S94: Publication year 1999.

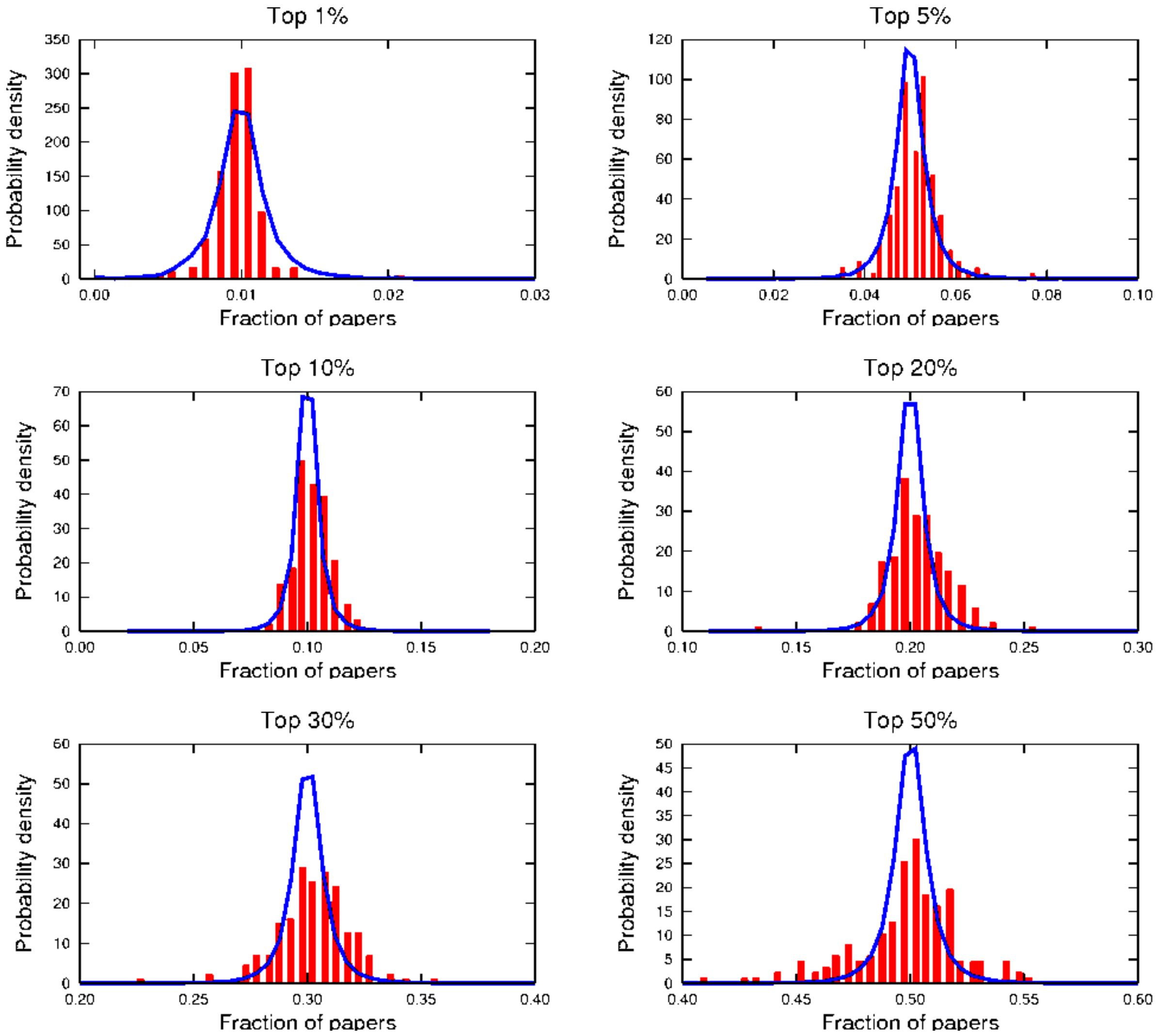

Figure S95: Publication year 1999.
