# Supplementary material for: A Reverse Engineering Approach to the Suppression of Citation Biases Reveals Universal Properties of Citation Distributions
Source: PLoS One. 2012 Mar 29;7(3):e33833. doi: 10.1371/journal.pone.0033833 (PMC3315498; doi:10.1371/journal.pone.0033833)
Supplement: Supporting Information S7 — Complete analysis for publication year . (PDF) [file pone.0033833.s007.pdf]

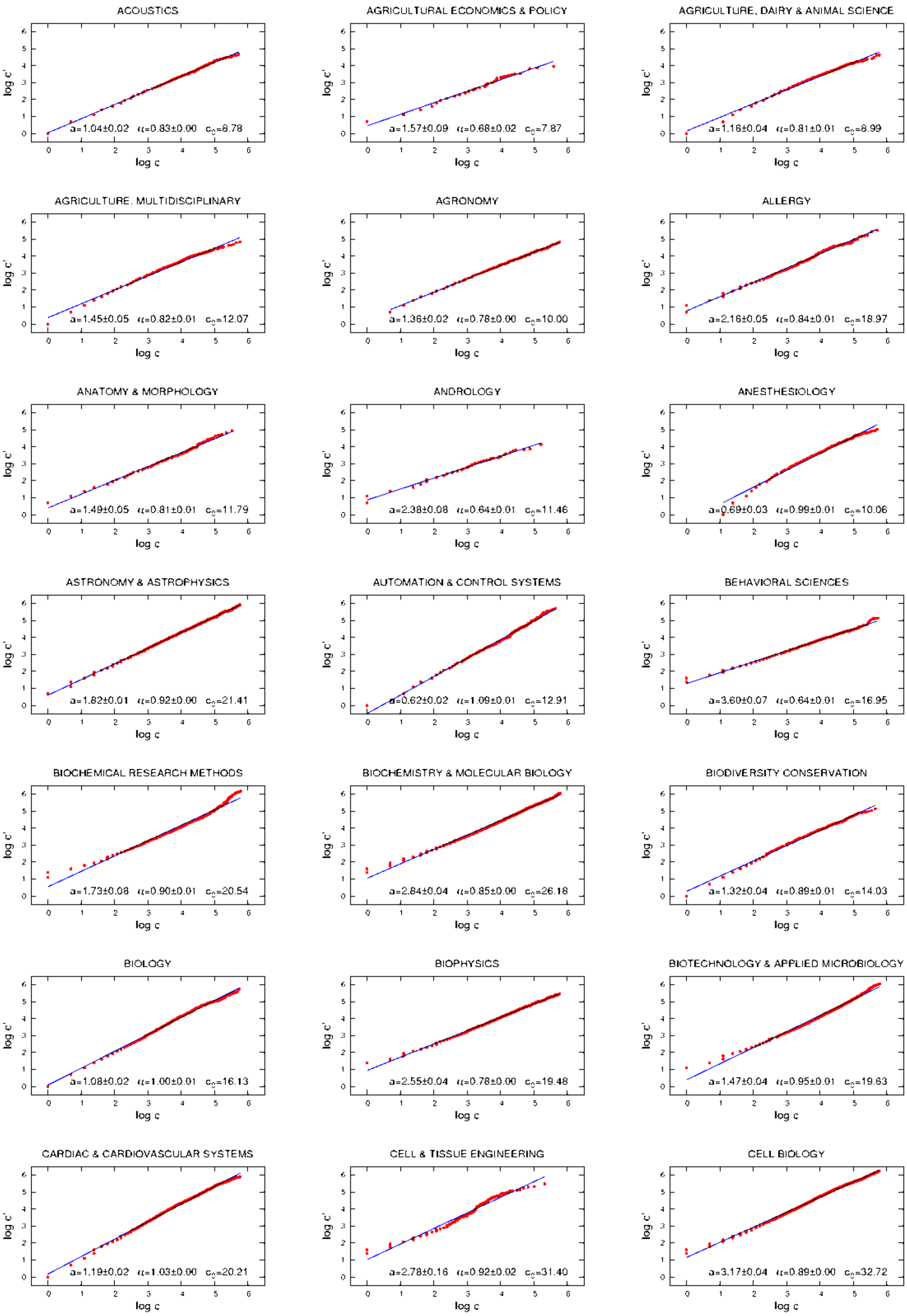

Figure S96: Publication year 2004.

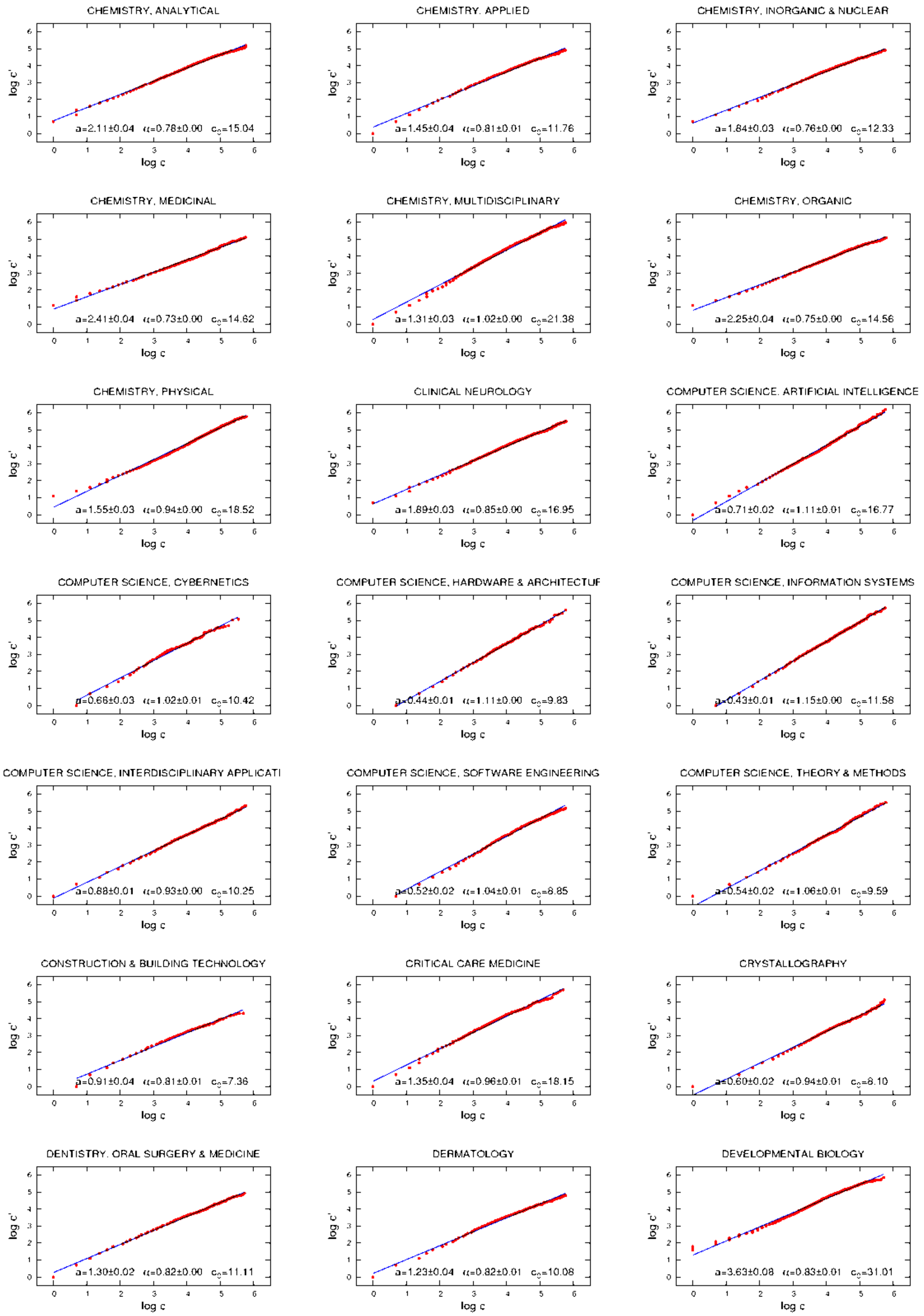

Figure S97: Publication year 2004.

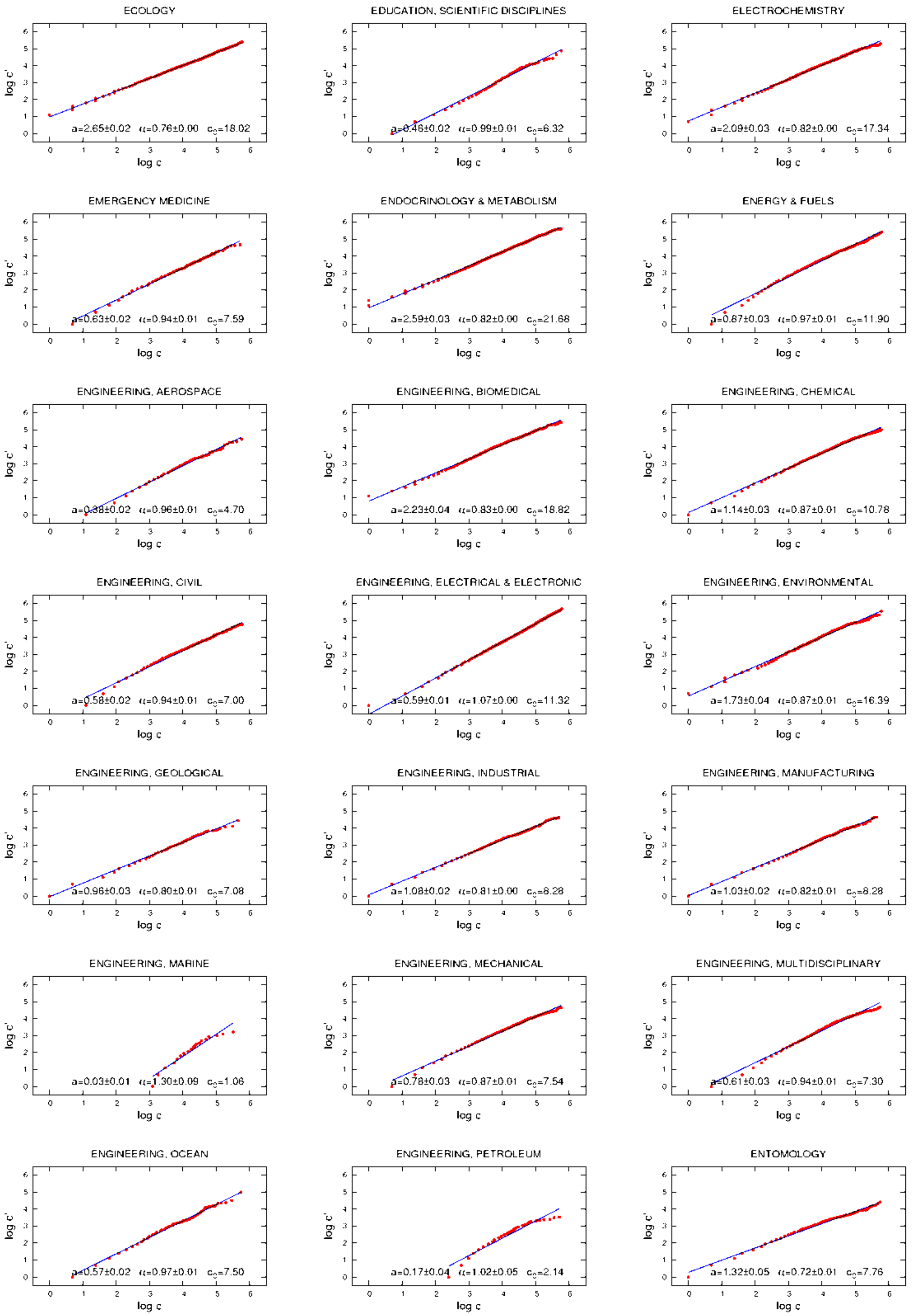

Figure S98: Publication year 2004.

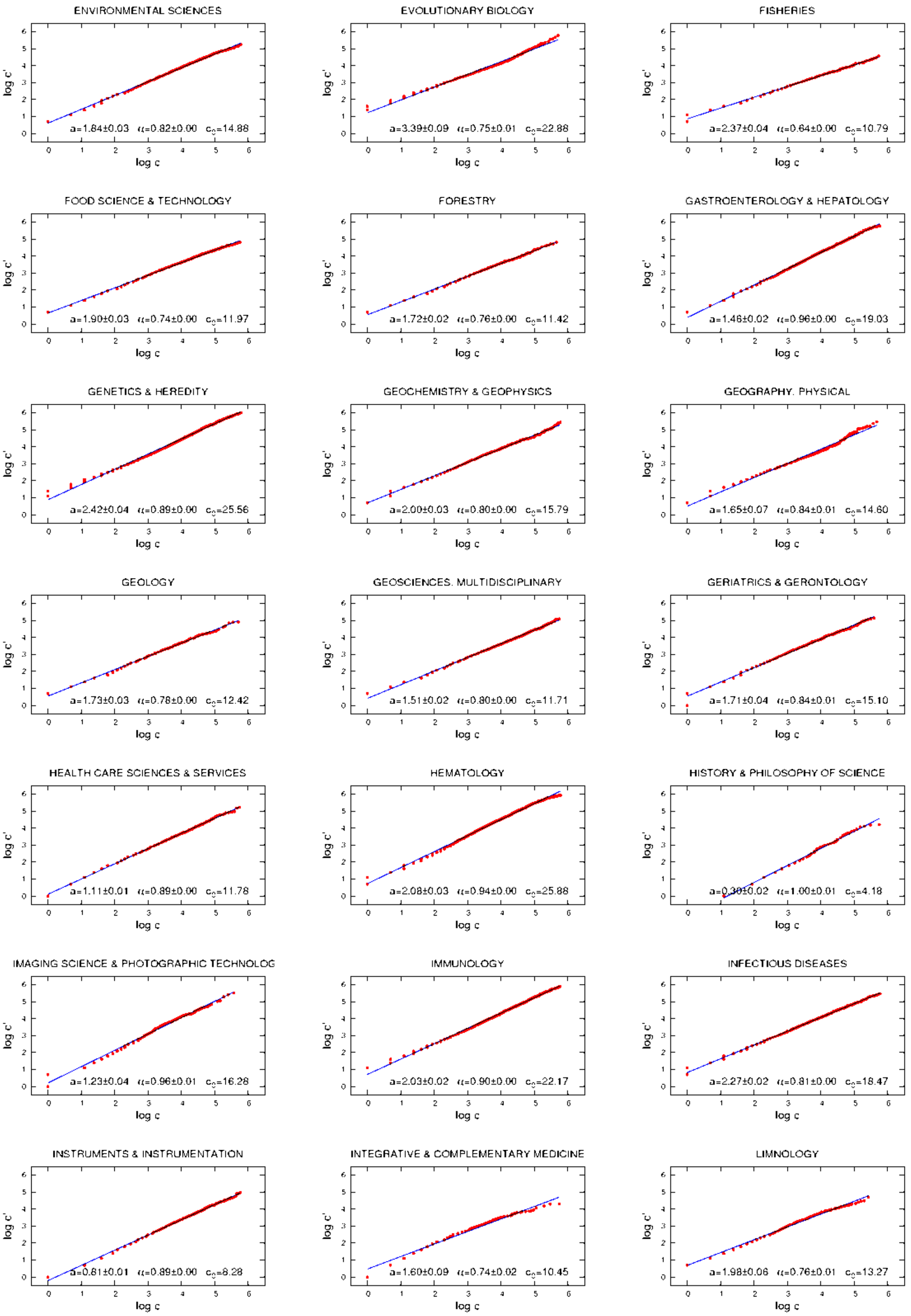

Figure S99: Publication year 2004.

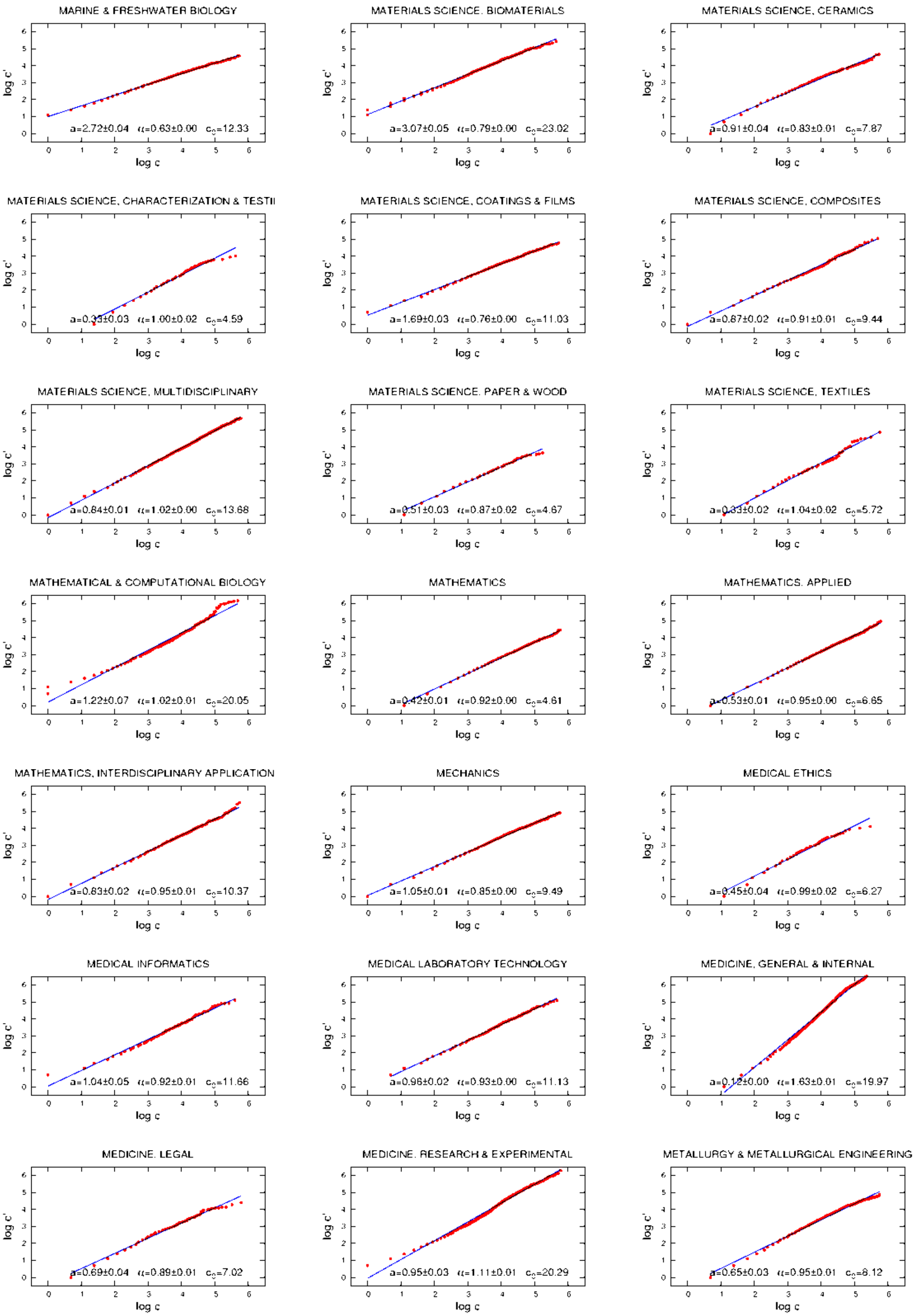

Figure S100: Publication year 2004.

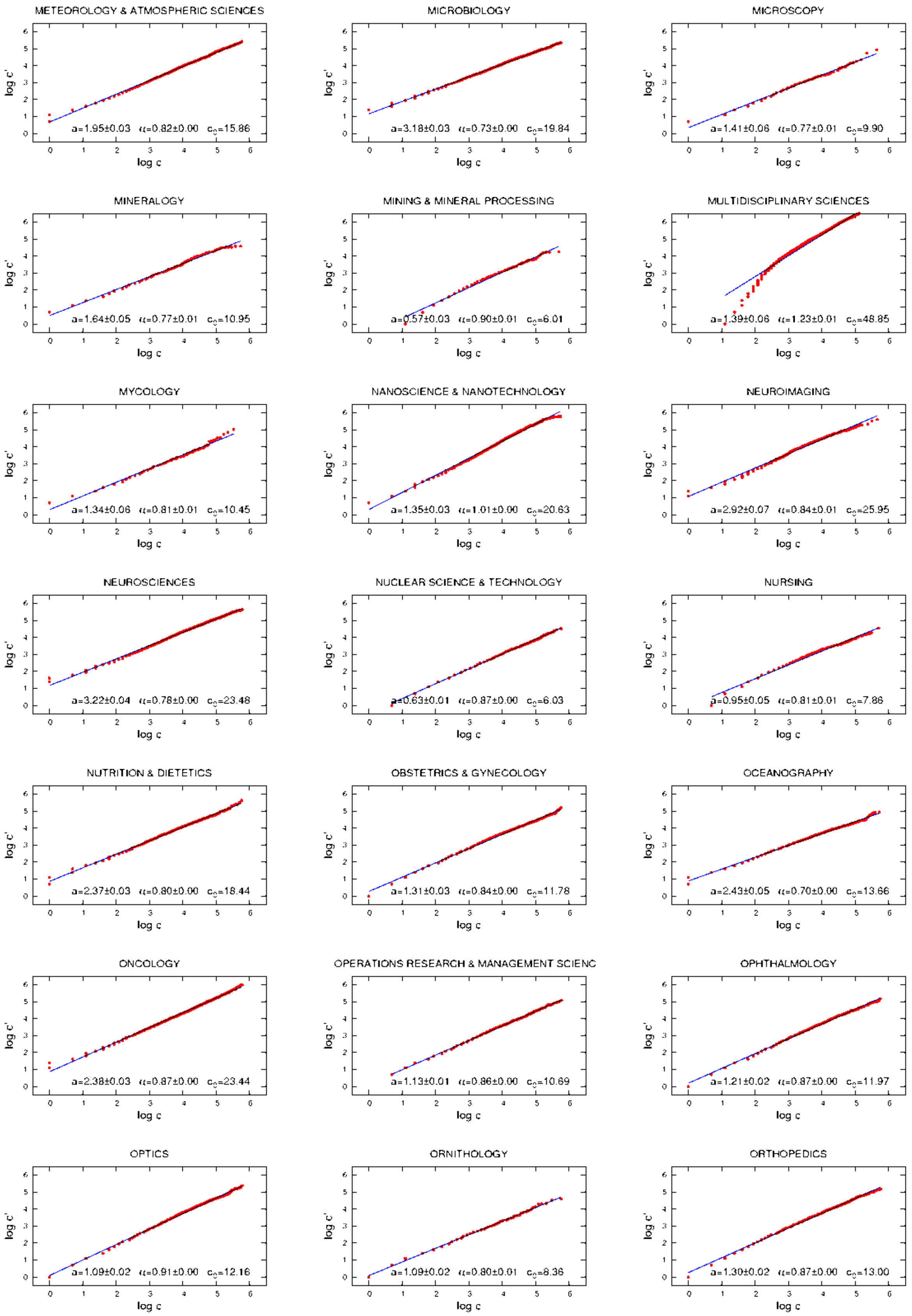

Figure S101: Publication year 2004.

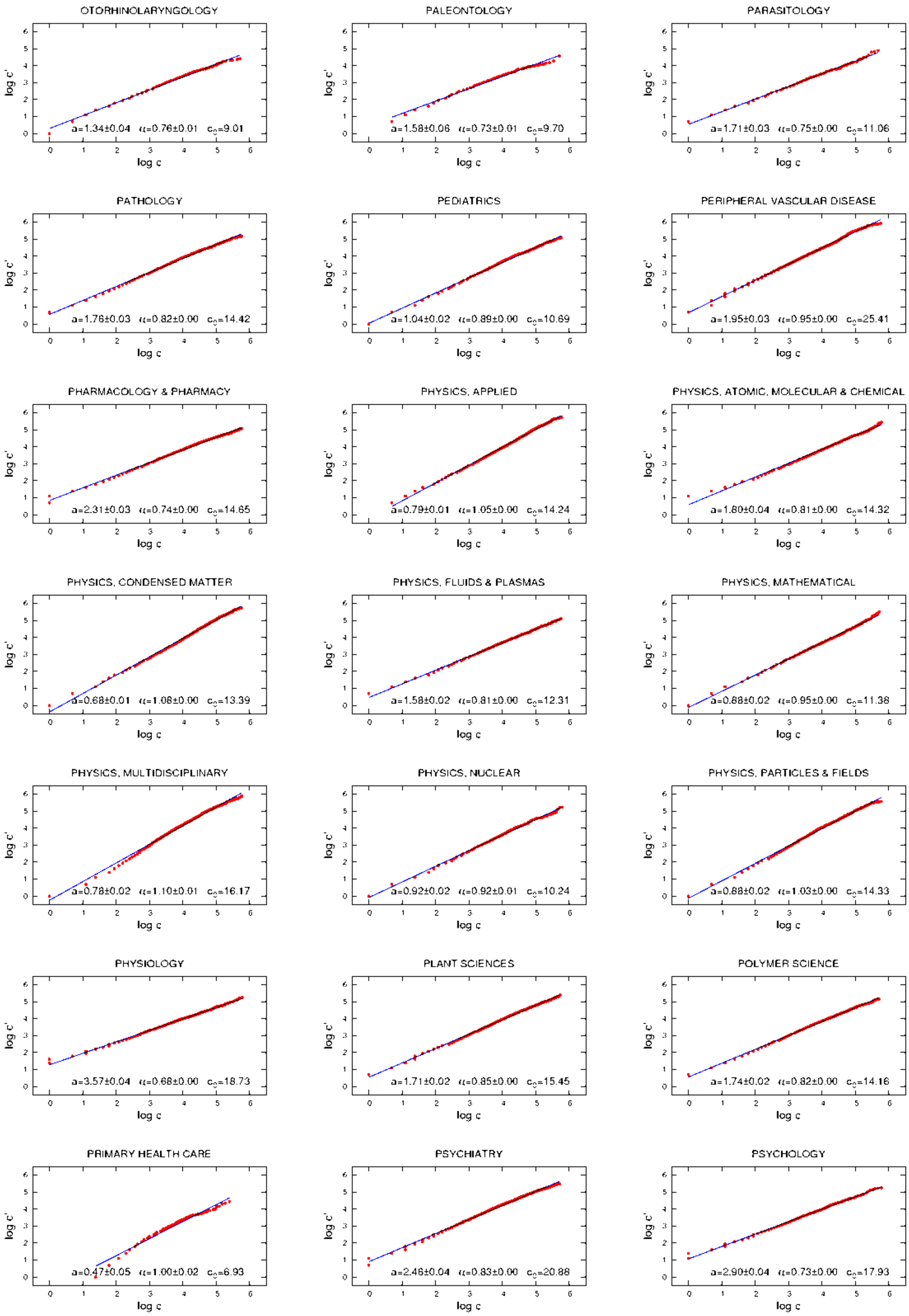

Figure S102: Publication year 2004.

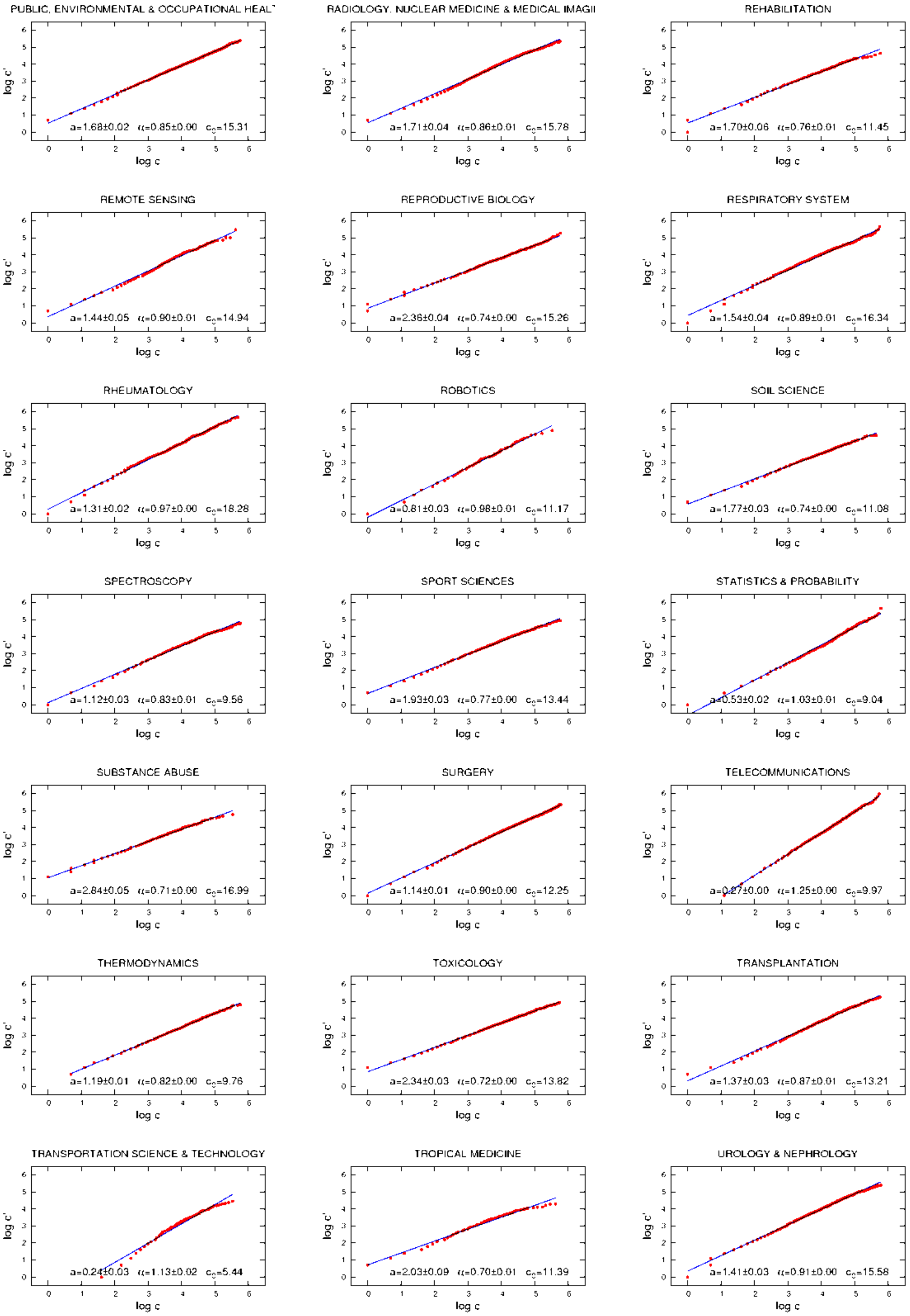

Figure S103: Publication year 2004.

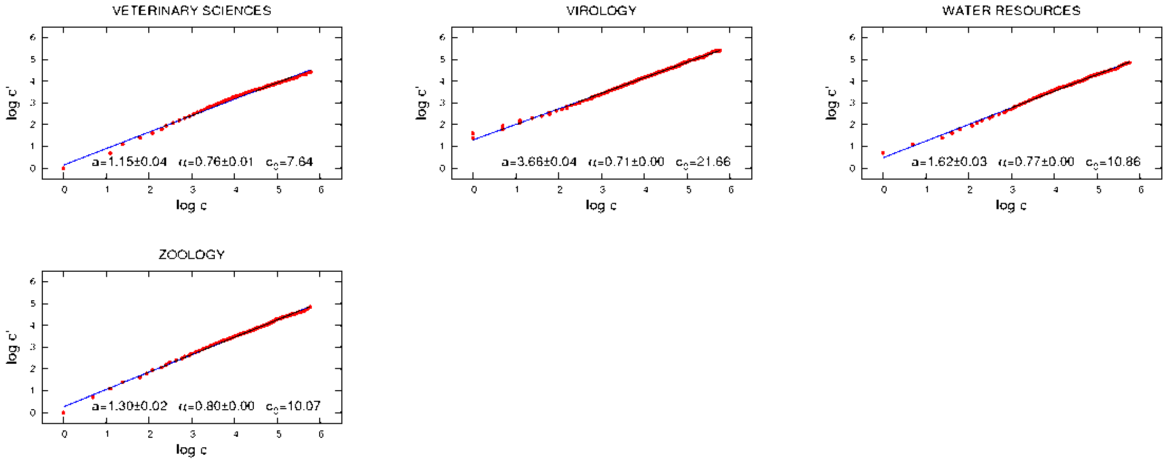

Figure S104: Publication year 2004.

Subject-category	a	α	$\langle c \rangle$	N
ACOUSTICS	1.04 ± 0.02	0.83 ± 0.00	8.78	3,361
AGRICULTURAL ECONOMICS & POLICY	1.57 ± 0.09	0.68 ± 0.02	7.87	592
AGRICULTURE, DAIRY & ANIMAL SCIENCE	1.16 ± 0.04	0.81 ± 0.01	8.99	3,868
AGRICULTURE, MULTIDISCIPLINARY	1.45 ± 0.05	0.82 ± 0.01	12.07	2,803
AGRONOMY	1.36 ± 0.02	0.78 ± 0.00	10.00	4,767
ALLERGY	2.16 ± 0.05	0.84 ± 0.01	18.97	1,617
ANATOMY & MORPHOLOGY	1.49 ± 0.05	0.81 ± 0.01	11.79	1,022
ANDROLOGY	2.38 ± 0.08	0.64 ± 0.01	11.46	248
ANESTHESIOLOGY	0.69 ± 0.03	0.99 ± 0.01	10.06	4,122
ASTRONOMY & ASTROPHYSICS	1.82 ± 0.01	0.92 ± 0.00	21.41	13,392
AUTOMATION & CONTROL SYSTEMS	0.62 ± 0.02	1.09 ± 0.01	12.91	3,449
BEHAVIORAL SCIENCES	3.60 ± 0.07	0.64 ± 0.01	16.95	3,426
BIOCHEMICAL RESEARCH METHODS	1.73 ± 0.08	0.90 ± 0.01	20.54	9,674
BIOCHEMISTRY & MOLECULAR BIOLOGY	2.84 ± 0.04	0.85 ± 0.00	26.18	43,556
BIODIVERSITY CONSERVATION	1.32 ± 0.04	0.89 ± 0.01	14.03	2,117
BIOLOGY	1.08 ± 0.02	1.00 ± 0.01	16.13	5,302
BIOPHYSICS	2.55 ± 0.04	0.78 ± 0.00	19.48	9,609
BIOTECHNOLOGY & APPLIED MICROBIOLOGY	1.47 ± 0.04	0.95 ± 0.01	19.63	13,899
CARDIAC & CARDIOVASCULAR SYSTEMS	1.19 ± 0.02	1.03 ± 0.00	20.21	12,472
CELL & TISSUE ENGINEERING	2.78 ± 0.16	0.92 ± 0.02	31.40	322
CELL BIOLOGY	3.17 ± 0.04	0.89 ± 0.00	32.72	17,610
CHEMISTRY, ANALYTICAL	2.11 ± 0.04	0.78 ± 0.00	15.04	14,446
CHEMISTRY, APPLIED	1.45 ± 0.04	0.81 ± 0.01	11.76	7,542
CHEMISTRY, INORGANIC & NUCLEAR	1.84 ± 0.03	0.76 ± 0.00	12.33	10,219
CHEMISTRY, MEDICINAL	2.41 ± 0.04	0.73 ± 0.00	14.62	6,444
CHEMISTRY, MULTIDISCIPLINARY	1.31 ± 0.03	1.02 ± 0.00	21.38	23,501
CHEMISTRY, ORGANIC	2.25 ± 0.04	0.75 ± 0.00	14.56	16,878
CHEMISTRY, PHYSICAL	1.55 ± 0.03	0.94 ± 0.00	18.52	29,735
CLINICAL NEUROLOGY	1.89 ± 0.03	0.85 ± 0.00	16.95	15,563
COMPUTER SCIENCE, ARTIFICIAL INTELLIGENCE	0.71 ± 0.02	1.11 ± 0.01	16.77	4,690
COMPUTER SCIENCE, CYBERNETICS	0.66 ± 0.03	1.02 ± 0.01	10.42	1,068
COMPUTER SCIENCE, HARDWARE & ARCHITECTURE	0.44 ± 0.01	1.11 ± 0.00	9.83	2,890
COMPUTER SCIENCE, INFORMATION SYSTEMS	0.43 ± 0.01	1.15 ± 0.00	11.58	4,633
COMPUTER SCIENCE, INTERDISCIPLINARY APPLICATIONS	0.88 ± 0.01	0.93 ± 0.00	10.25	5,761
COMPUTER SCIENCE, SOFTWARE ENGINEERING	0.52 ± 0.02	1.04 ± 0.01	8.85	4,718
COMPUTER SCIENCE, THEORY & METHODS	0.54 ± 0.02	1.06 ± 0.01	9.59	3,918
CONSTRUCTION & BUILDING TECHNOLOGY	0.91 ± 0.04	0.81 ± 0.01	7.36	2,302
CRITICAL CARE MEDICINE	1.35 ± 0.04	0.96 ± 0.01	18.15	3,116
CRYSTALLOGRAPHY	0.60 ± 0.02	0.94 ± 0.01	8.10	7,032
DENTISTRY, ORAL SURGERY & MEDICINE	1.30 ± 0.02	0.82 ± 0.00	11.11	5,040
DERMATOLOGY	1.23 ± 0.04	0.82 ± 0.01	10.08	4,808
DEVELOPMENTAL BIOLOGY	3.63 ± 0.08	0.83 ± 0.01	31.01	3,289
ECOLOGY	2.65 ± 0.02	0.76 ± 0.00	18.02	9,860
EDUCATION, SCIENTIFIC DISCIPLINES	0.46 ± 0.02	0.99 ± 0.01	6.32	1,930
ELECTROCHEMISTRY	2.09 ± 0.03	0.82 ± 0.00	17.34	5,539
EMERGENCY MEDICINE	0.63 ± 0.02	0.94 ± 0.01	7.59	1,661
ENDOCRINOLOGY & METABOLISM	2.59 ± 0.03	0.82 ± 0.00	21.68	11,259
ENERGY & FUELS	0.87 ± 0.03	0.97 ± 0.01	11.90	5,977
ENGINEERING, AEROSPACE	0.38 ± 0.02	0.96 ± 0.01	4.70	1,902
ENGINEERING, BIOMEDICAL	2.23 ± 0.04	0.83 ± 0.00	18.82	4,717

Table S33: Publication year 2004.

Subject-category	a	α	$\langle c \rangle$	N
ENGINEERING, CHEMICAL	1.14 ± 0.03	0.87 ± 0.01	10.78	13,612
ENGINEERING, CIVIL	0.58 ± 0.02	0.94 ± 0.01	7.00	5,972
ENGINEERING, ELECTRICAL & ELECTRONIC	0.59 ± 0.01	1.07 ± 0.00	11.32	26,432
ENGINEERING, ENVIRONMENTAL	1.73 ± 0.04	0.87 ± 0.01	16.39	4,850
ENGINEERING, GEOLOGICAL	0.96 ± 0.03	0.80 ± 0.01	7.08	1,406
ENGINEERING, INDUSTRIAL	1.08 ± 0.02	0.81 ± 0.00	8.28	3,109
ENGINEERING, MANUFACTURING	1.03 ± 0.02	0.82 ± 0.01	8.28	3,385
ENGINEERING, MARINE	0.03 ± 0.01	1.30 ± 0.09	1.06	489
ENGINEERING, MECHANICAL	0.78 ± 0.03	0.87 ± 0.01	7.54	8,503
ENGINEERING, MULTIDISCIPLINARY	0.61 ± 0.03	0.94 ± 0.01	7.30	4,443
ENGINEERING, OCEAN	0.57 ± 0.02	0.97 ± 0.01	7.50	874
ENGINEERING, PETROLEUM	0.17 ± 0.04	1.02 ± 0.05	2.14	1,613
ENTOMOLOGY	1.32 ± 0.05	0.72 ± 0.01	7.76	4,371
ENVIRONMENTAL SCIENCES	1.84 ± 0.03	0.82 ± 0.00	14.88	16,938
EVOLUTIONARY BIOLOGY	3.39 ± 0.09	0.75 ± 0.01	22.88	3,170
FISHERIES	2.37 ± 0.04	0.64 ± 0.00	10.79	3,495
FOOD SCIENCE & TECHNOLOGY	1.90 ± 0.03	0.74 ± 0.00	11.97	9,457
FORESTRY	1.72 ± 0.02	0.76 ± 0.00	11.42	2,811
GASTROENTEROLOGY & HEPATOLOGY	1.46 ± 0.02	0.96 ± 0.00	19.03	7,518
GENETICS & HEREDITY	2.42 ± 0.04	0.89 ± 0.00	25.56	12,947
GEOCHEMISTRY & GEOPHYSICS	2.00 ± 0.03	0.80 ± 0.00	15.79	5,777
GEOGRAPHY, PHYSICAL	1.65 ± 0.07	0.84 ± 0.01	14.60	2,230
GEOLOGY	1.73 ± 0.03	0.78 ± 0.00	12.42	1,604
GEOSCIENCES, MULTIDISCIPLINARY	1.51 ± 0.02	0.80 ± 0.00	11.71	10,683
GERIATRICS & GERONTOLOGY	1.71 ± 0.04	0.84 ± 0.01	15.10	2,387
HEALTH CARE SCIENCES & SERVICES	1.11 ± 0.01	0.89 ± 0.00	11.78	3,577
HEMATOLOGY	2.08 ± 0.03	0.94 ± 0.00	25.88	9,875
HISTORY & PHILOSOPHY OF SCIENCE	0.30 ± 0.02	1.00 ± 0.01	4.18	919
IMAGING SCIENCE & PHOTOGRAPHIC TECHNOLOGY	1.23 ± 0.04	0.96 ± 0.01	16.28	1,136
IMMUNOLOGY	2.03 ± 0.02	0.90 ± 0.00	22.17	17,048
INFECTIOUS DISEASES	2.27 ± 0.02	0.81 ± 0.00	18.47	7,727
INSTRUMENTS & INSTRUMENTATION	0.81 ± 0.01	0.89 ± 0.00	8.28	8,599
INTEGRATIVE & COMPLEMENTARY MEDICINE	1.60 ± 0.09	0.74 ± 0.02	10.45	885
LIMNOLOGY	1.98 ± 0.06	0.76 ± 0.01	13.27	1,208
MARINE & FRESHWATER BIOLOGY	2.72 ± 0.04	0.63 ± 0.00	12.33	6,939
MATERIALS SCIENCE, BIOMATERIALS	3.07 ± 0.05	0.79 ± 0.00	23.02	2,082
MATERIALS SCIENCE, CERAMICS	0.91 ± 0.04	0.83 ± 0.01	7.87	3,443
MATERIALS SCIENCE, CHARACTERIZATION & TESTING	0.33 ± 0.03	1.00 ± 0.02	4.59	1,293
MATERIALS SCIENCE, COATINGS & FILMS	1.69 ± 0.03	0.76 ± 0.00	11.03	4,993
MATERIALS SCIENCE, COMPOSITES	0.87 ± 0.02	0.91 ± 0.01	9.44	1,539
MATERIALS SCIENCE, MULTIDISCIPLINARY	0.84 ± 0.01	1.02 ± 0.00	13.68	34,391
MATERIALS SCIENCE, PAPER & WOOD	0.51 ± 0.03	0.87 ± 0.02	4.67	1,048
MATERIALS SCIENCE, TEXTILES	0.33 ± 0.02	1.04 ± 0.02	5.72	949
MATHEMATICAL & COMPUTATIONAL BIOLOGY	1.22 ± 0.07	1.02 ± 0.01	20.05	2,304
MATHEMATICS	0.42 ± 0.01	0.92 ± 0.00	4.61	13,390
MATHEMATICS, APPLIED	0.53 ± 0.01	0.95 ± 0.00	6.65	11,863
MATHEMATICS, INTERDISCIPLINARY APPLICATIONS	0.83 ± 0.02	0.95 ± 0.01	10.37	4,370
MECHANICS	1.05 ± 0.01	0.85 ± 0.00	9.49	10,165
MEDICAL ETHICS	0.45 ± 0.04	0.99 ± 0.02	6.27	443
MEDICAL INFORMATICS	1.04 ± 0.05	0.92 ± 0.01	11.66	1,196

Table S34: Publication year 2004.

Subject-category	a	α	$\langle c \rangle$	N
MEDICAL LABORATORY TECHNOLOGY	0.96 ± 0.02	0.93 ± 0.00	11.13	2,210
MEDICINE, GENERAL & INTERNAL	0.12 ± 0.00	1.63 ± 0.01	19.97	14,814
MEDICINE, LEGAL	0.69 ± 0.04	0.89 ± 0.01	7.02	993
MEDICINE, RESEARCH & EXPERIMENTAL	0.95 ± 0.03	1.11 ± 0.01	20.29	8,861
METALLURGY & METALLURGICAL ENGINEERING	0.65 ± 0.03	0.95 ± 0.01	8.12	8,077
METEOROLOGY & ATMOSPHERIC SCIENCES	1.95 ± 0.03	0.82 ± 0.00	15.86	6,720
MICROBIOLOGY	3.18 ± 0.03	0.73 ± 0.00	19.84	13,224
MICROSCOPY	1.41 ± 0.06	0.77 ± 0.01	9.90	674
MINERALOGY	1.64 ± 0.05	0.77 ± 0.01	10.95	1,724
MINING & MINERAL PROCESSING	0.57 ± 0.03	0.90 ± 0.01	6.01	1,553
MULTIDISCIPLINARY SCIENCES	1.39 ± 0.06	1.23 ± 0.01	48.85	10,909
MYCOLOGY	1.34 ± 0.06	0.81 ± 0.01	10.45	1,019
NANOSCIENCE & NANOTECHNOLOGY	1.35 ± 0.03	1.01 ± 0.00	20.63	7,183
NEUROIMAGING	2.92 ± 0.07	0.84 ± 0.01	25.95	1,430
NEUROSCIENCES	3.22 ± 0.04	0.78 ± 0.00	23.48	23,796
NUCLEAR SCIENCE & TECHNOLOGY	0.63 ± 0.01	0.87 ± 0.00	6.03	7,589
NURSING	0.95 ± 0.05	0.81 ± 0.01	7.86	2,365
NUTRITION & DIETETICS	2.37 ± 0.03	0.80 ± 0.00	18.44	4,767
OBSTETRICS & GYNECOLOGY	1.31 ± 0.03	0.84 ± 0.00	11.78	7,384
OCEANOGRAPHY	2.43 ± 0.05	0.70 ± 0.00	13.66	4,159
ONCOLOGY	2.38 ± 0.03	0.87 ± 0.00	23.44	19,647
OPERATIONS RESEARCH & MANAGEMENT SCIENCE	1.13 ± 0.01	0.86 ± 0.00	10.69	3,902
OPHTHALMOLOGY	1.21 ± 0.02	0.87 ± 0.00	11.97	6,359
OPTICS	1.09 ± 0.02	0.91 ± 0.00	12.16	12,693
ORNITHOLOGY	1.09 ± 0.02	0.80 ± 0.01	8.36	928
ORTHOPEDICS	1.30 ± 0.02	0.87 ± 0.00	13.00	5,607
OTORHINOLARYNGOLOGY	1.34 ± 0.04	0.76 ± 0.01	9.01	3,235
PALEONTOLOGY	1.58 ± 0.06	0.73 ± 0.01	9.70	1,559
PARASITOLOGY	1.71 ± 0.03	0.75 ± 0.00	11.06	2,239
PATHOLOGY	1.76 ± 0.03	0.82 ± 0.00	14.42	5,694
PEDIATRICS	1.04 ± 0.02	0.89 ± 0.00	10.69	9,553
PERIPHERAL VASCULAR DISEASE	1.95 ± 0.03	0.95 ± 0.00	25.41	8,353
PHARMACOLOGY & PHARMACY	2.31 ± 0.03	0.74 ± 0.00	14.65	20,991
PHYSICS, APPLIED	0.79 ± 0.01	1.05 ± 0.00	14.24	28,999
PHYSICS, ATOMIC, MOLECULAR & CHEMICAL	1.80 ± 0.04	0.81 ± 0.00	14.32	12,973
PHYSICS, CONDENSED MATTER	0.68 ± 0.01	1.08 ± 0.00	13.39	22,654
PHYSICS, FLUIDS & PLASMAS	1.58 ± 0.02	0.81 ± 0.00	12.31	5,648
PHYSICS, MATHEMATICAL	0.88 ± 0.02	0.95 ± 0.00	11.38	6,624
PHYSICS, MULTIDISCIPLINARY	0.78 ± 0.02	1.10 ± 0.01	16.17	15,438
PHYSICS, NUCLEAR	0.92 ± 0.02	0.92 ± 0.01	10.24	4,987
PHYSICS, PARTICLES & FIELDS	0.88 ± 0.02	1.03 ± 0.00	14.33	8,759
PHYSIOLOGY	3.57 ± 0.04	0.68 ± 0.00	18.73	7,846
PLANT SCIENCES	1.71 ± 0.02	0.85 ± 0.00	15.45	12,844
POLYMER SCIENCE	1.74 ± 0.02	0.82 ± 0.00	14.16	11,170
PRIMARY HEALTH CARE	0.47 ± 0.05	1.00 ± 0.02	6.93	1,140
PSYCHIATRY	2.46 ± 0.04	0.83 ± 0.00	20.88	9,108
PSYCHOLOGY	2.90 ± 0.04	0.73 ± 0.00	17.93	2,942
PUBLIC, ENVIRONMENTAL & OCCUPATIONAL HEALTH	1.68 ± 0.02	0.85 ± 0.00	15.31	10,171
RADIOLOGY, NUCLEAR MEDICINE & MEDICAL IMAGING	1.71 ± 0.04	0.86 ± 0.01	15.78	12,165
REHABILITATION	1.70 ± 0.06	0.76 ± 0.01	11.45	1,863

Table S35: Publication year 2004.

Subject-category	a	α	$\langle c \rangle$	N
REMOTE SENSING	1.44 ± 0.05	0.90 ± 0.01	14.94	1,301
REPRODUCTIVE BIOLOGY	2.36 ± 0.04	0.74 ± 0.00	15.26	3,710
RESPIRATORY SYSTEM	1.54 ± 0.04	0.89 ± 0.01	16.34	6,259
RHEUMATOLOGY	1.31 ± 0.02	0.97 ± 0.00	18.28	3,058
ROBOTICS	0.81 ± 0.03	0.98 ± 0.01	11.17	497
SOIL SCIENCE	1.77 ± 0.03	0.74 ± 0.00	11.08	2,766
SPECTROSCOPY	1.12 ± 0.03	0.83 ± 0.01	9.56	6,648
SPORT SCIENCES	1.93 ± 0.03	0.77 ± 0.00	13.44	4,701
STATISTICS & PROBABILITY	0.53 ± 0.02	1.03 ± 0.01	9.04	4,922
SUBSTANCE ABUSE	2.84 ± 0.05	0.71 ± 0.00	16.99	1,049
SURGERY	1.14 ± 0.01	0.90 ± 0.00	12.25	22,687
TELECOMMUNICATIONS	0.27 ± 0.00	1.25 ± 0.00	9.97	5,196
THERMODYNAMICS	1.19 ± 0.01	0.82 ± 0.00	9.76	3,809
TOXICOLOGY	2.34 ± 0.03	0.72 ± 0.00	13.82	6,214
TRANSPLANTATION	1.37 ± 0.03	0.87 ± 0.01	13.21	4,665
TRANSPORTATION SCIENCE & TECHNOLOGY	0.24 ± 0.03	1.13 ± 0.02	5.44	1,562
TROPICAL MEDICINE	2.03 ± 0.09	0.70 ± 0.01	11.39	1,298
UROLOGY & NEPHROLOGY	1.41 ± 0.03	0.91 ± 0.00	15.58	7,784
VETERINARY SCIENCES	1.15 ± 0.04	0.76 ± 0.01	7.64	7,967
VIROLOGY	3.66 ± 0.04	0.71 ± 0.00	21.66	4,713
WATER RESOURCES	1.62 ± 0.03	0.77 ± 0.00	10.86	5,490
ZOOLOGY	1.30 ± 0.02	0.80 ± 0.00	10.07	6,684
**TOTAL**	$\mathbf{1}.00 \pm 0.00$	$\mathbf{1}.00 \pm 0.00$	**1**5.79	**1**205,646

Table S36: Publication year 2004.

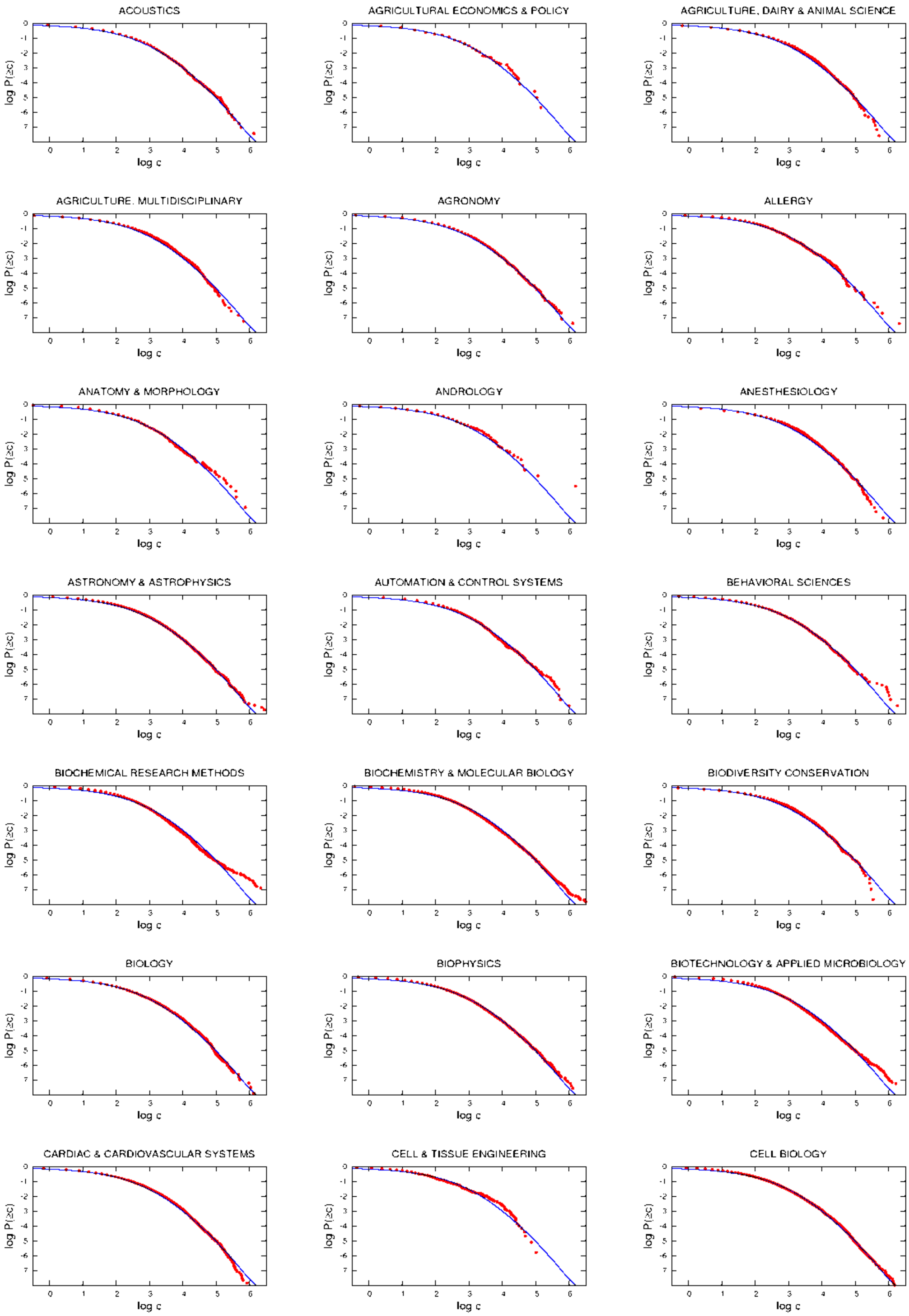

Figure S105: Publication year 2004.

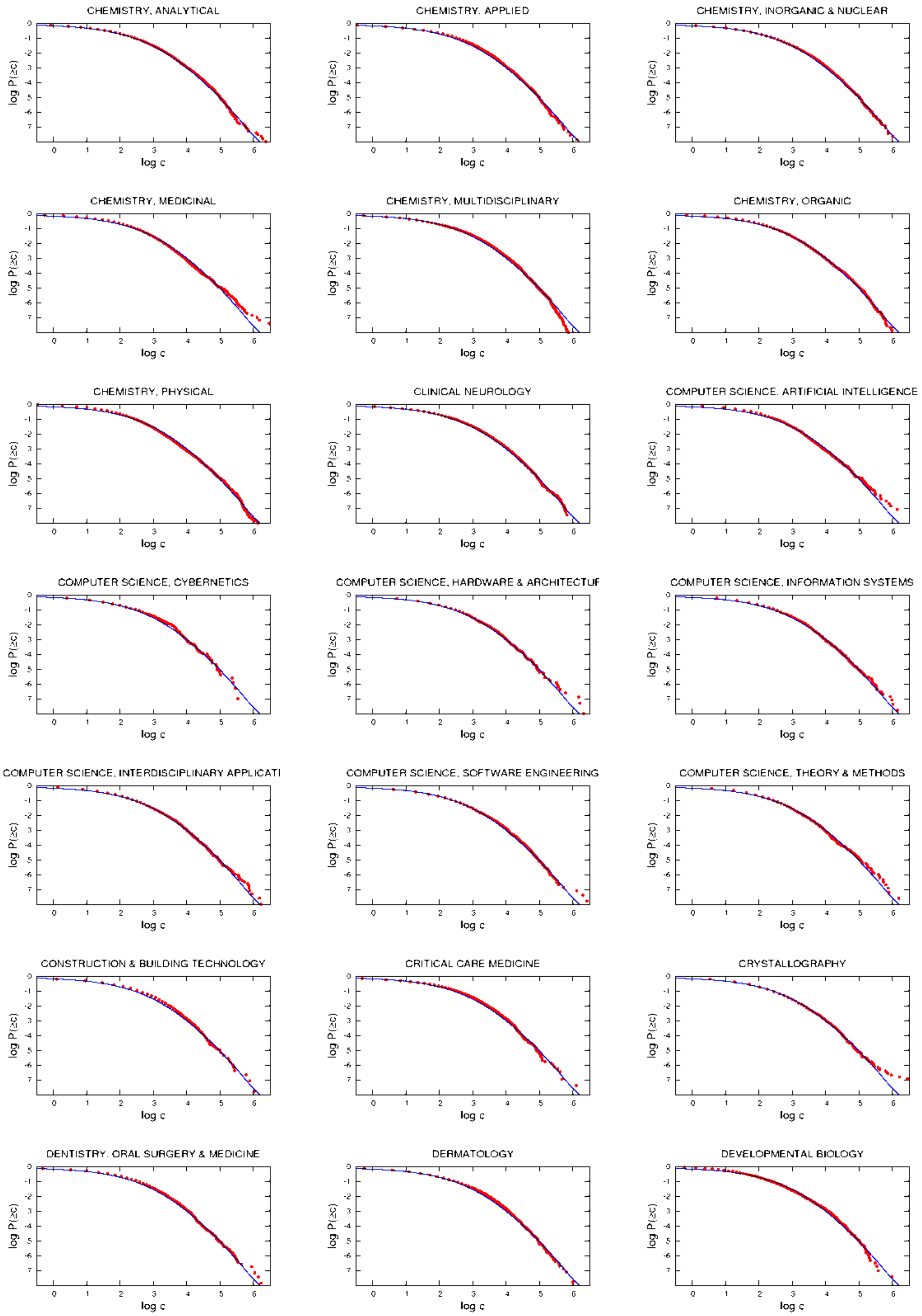

Figure S106: Publication year 2004.

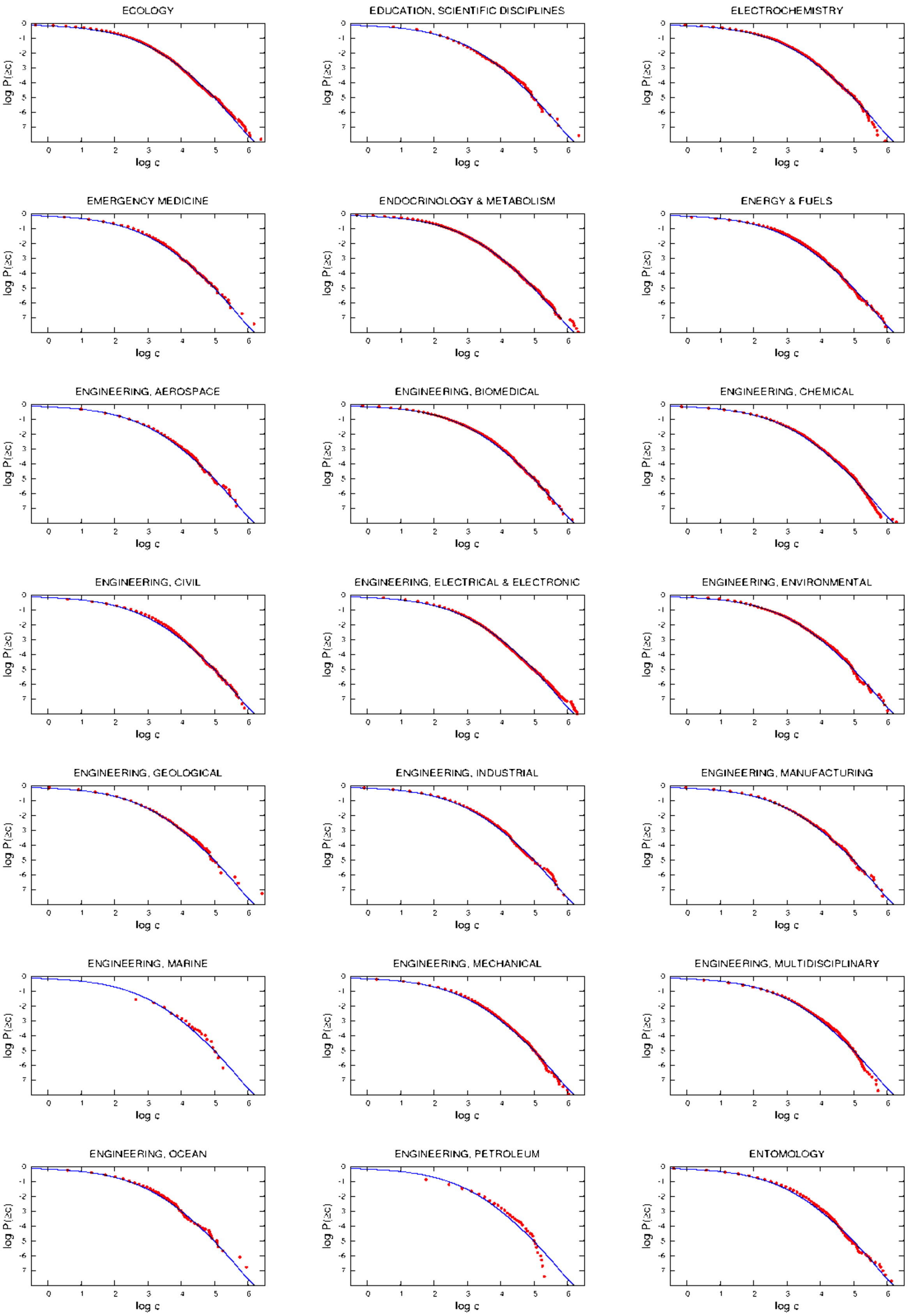

Figure S107: Publication year 2004.

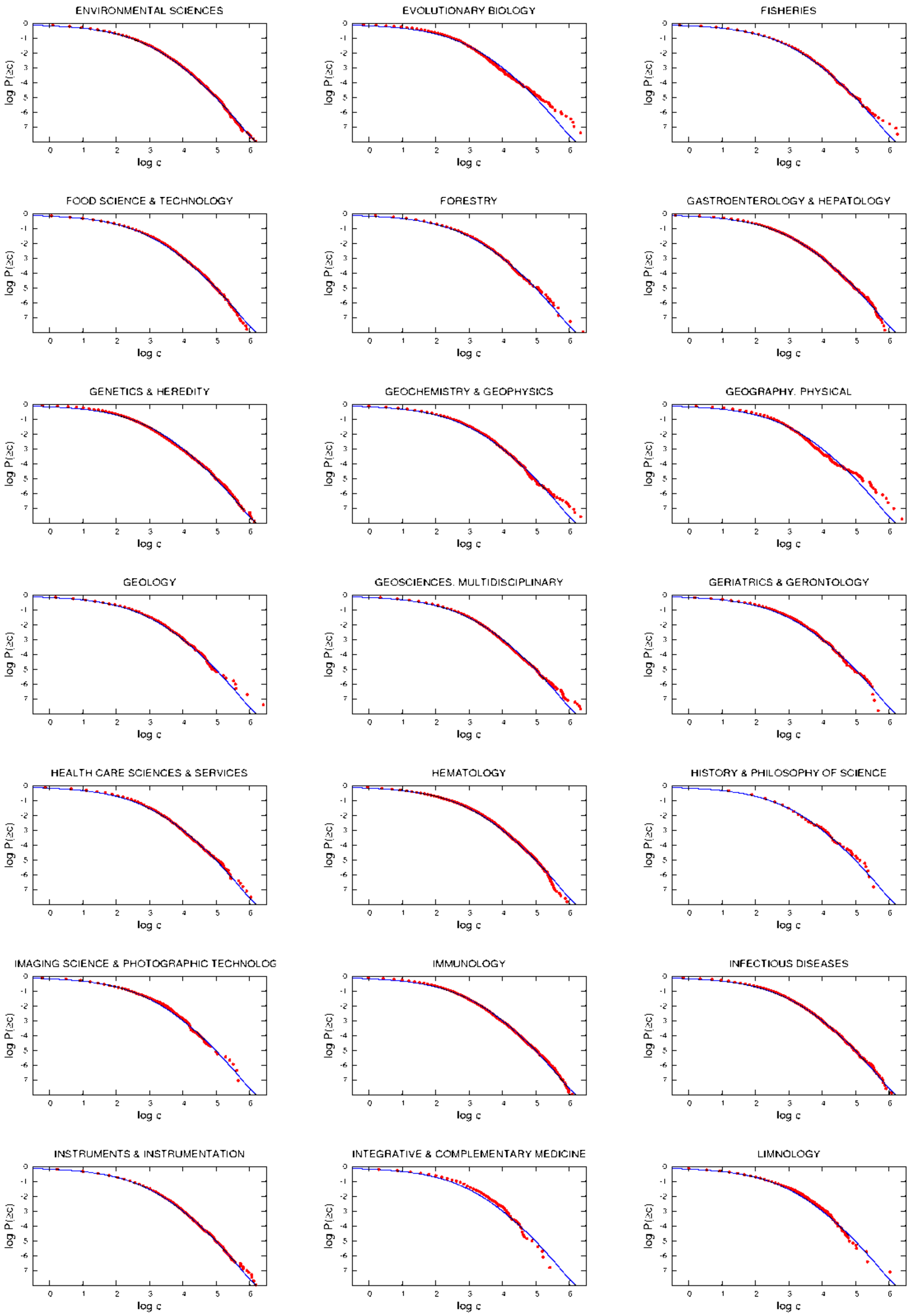

Figure S108: Publication year 2004.

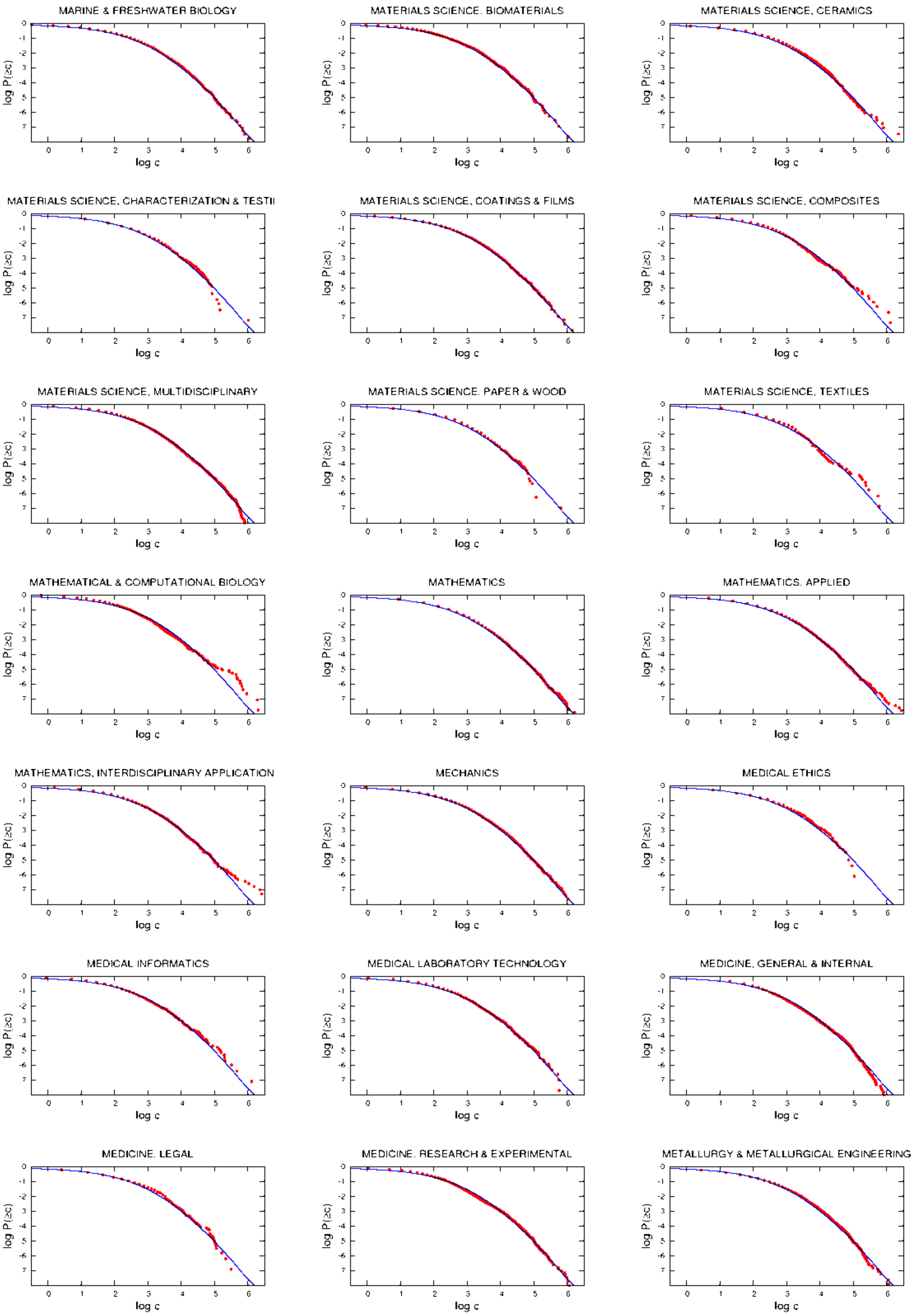

Figure S109: Publication year 2004.

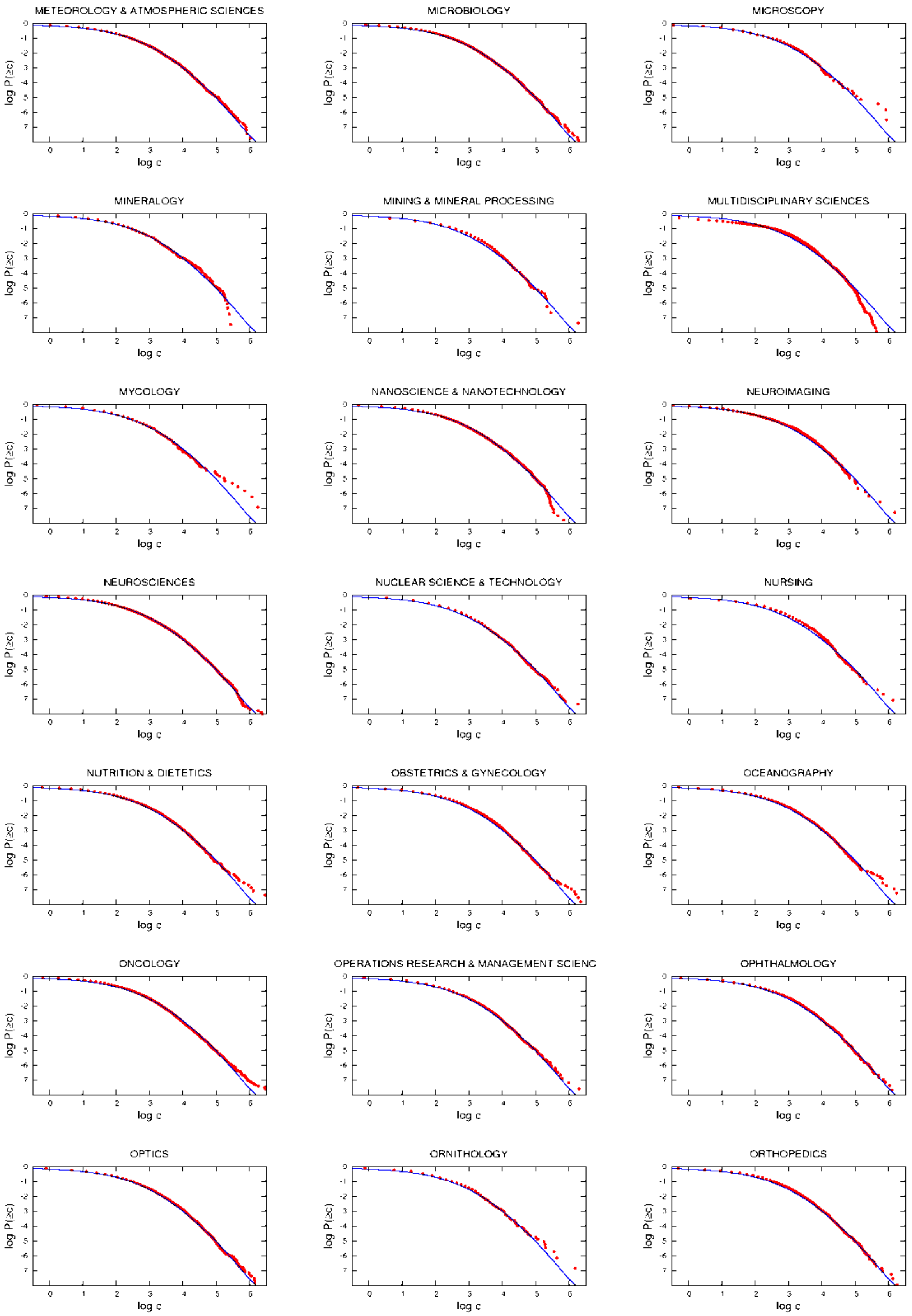

Figure S110: Publication year 2004.

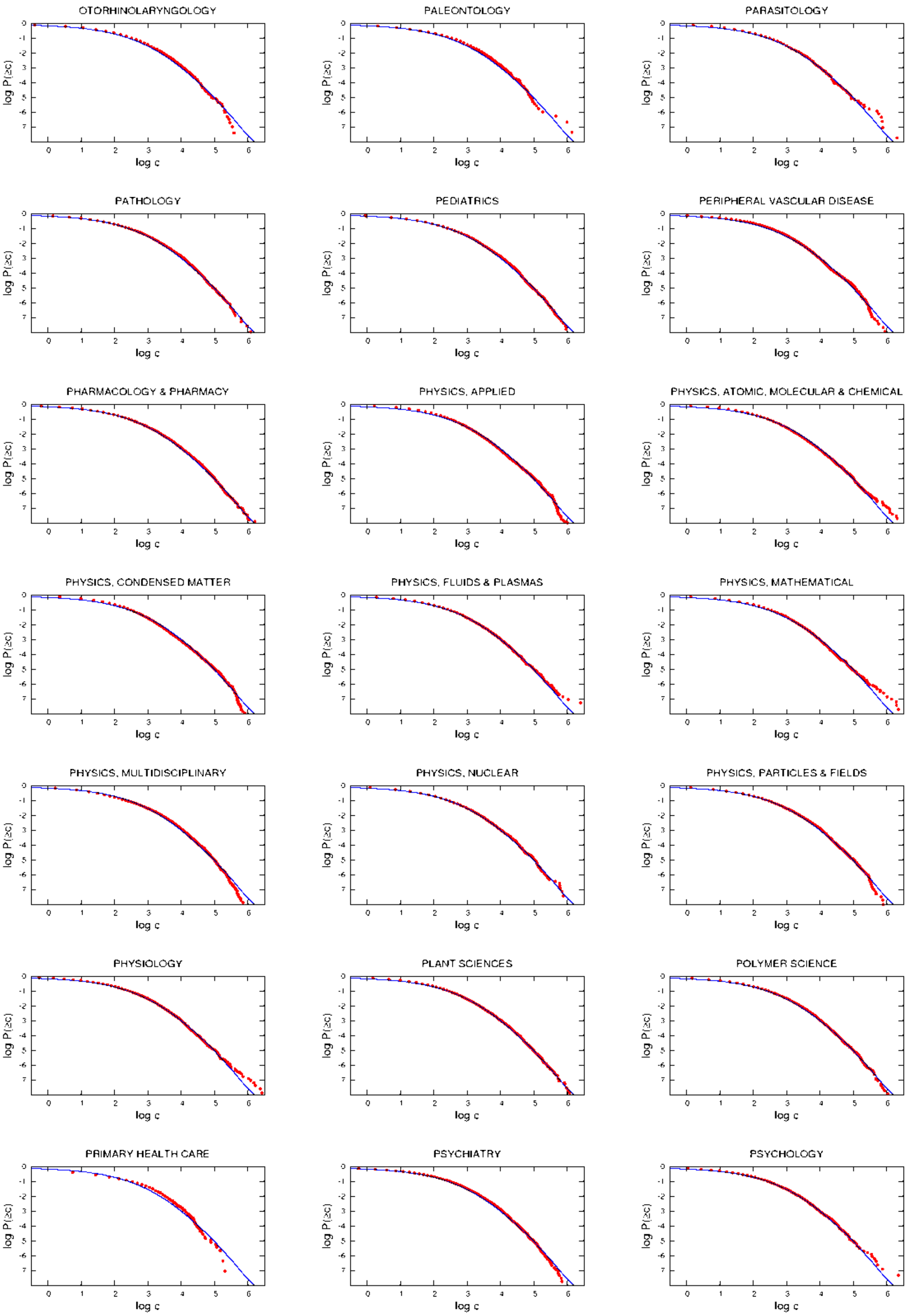

Figure S111: Publication year 2004.

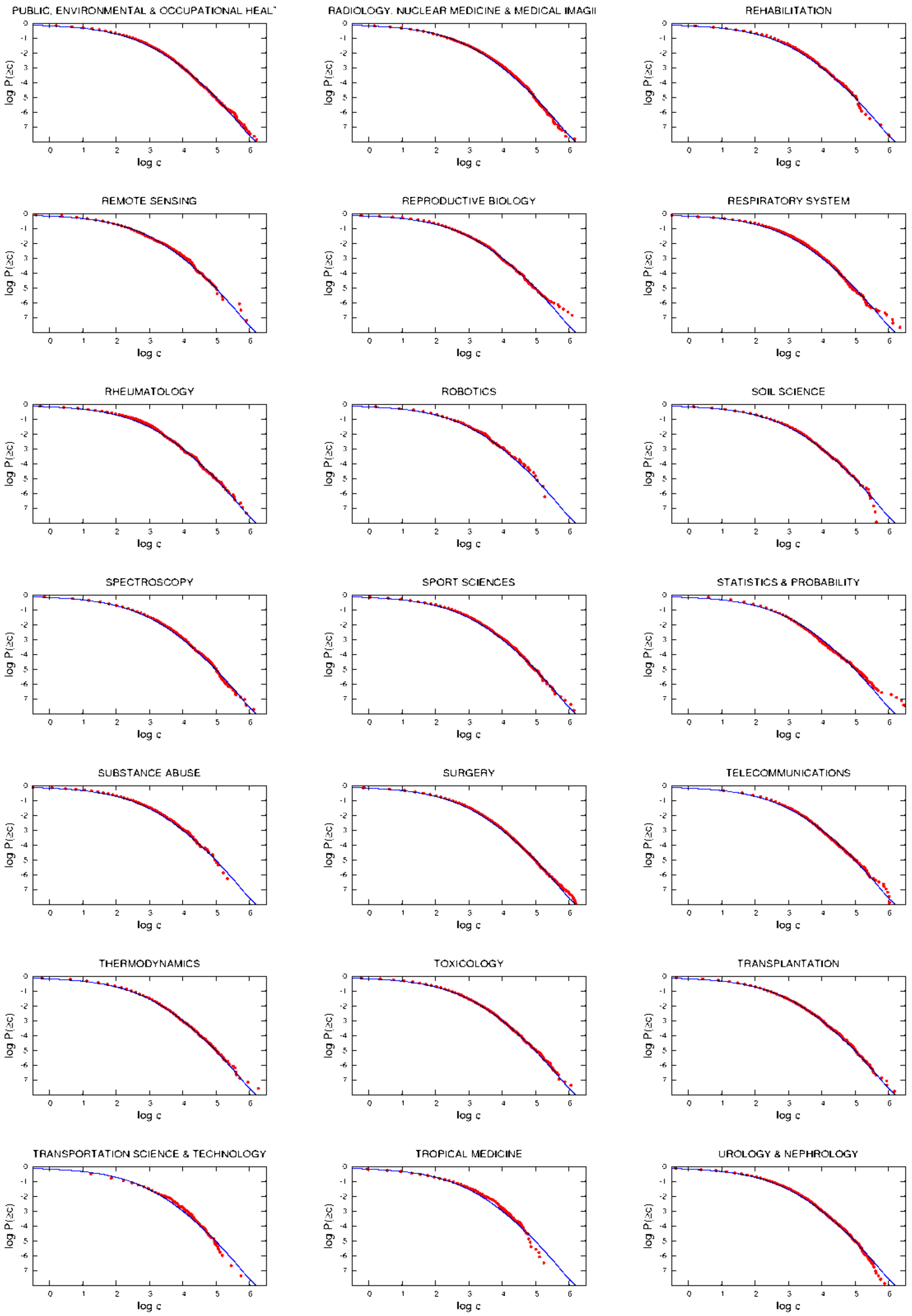

Figure S112: Publication year 2004.

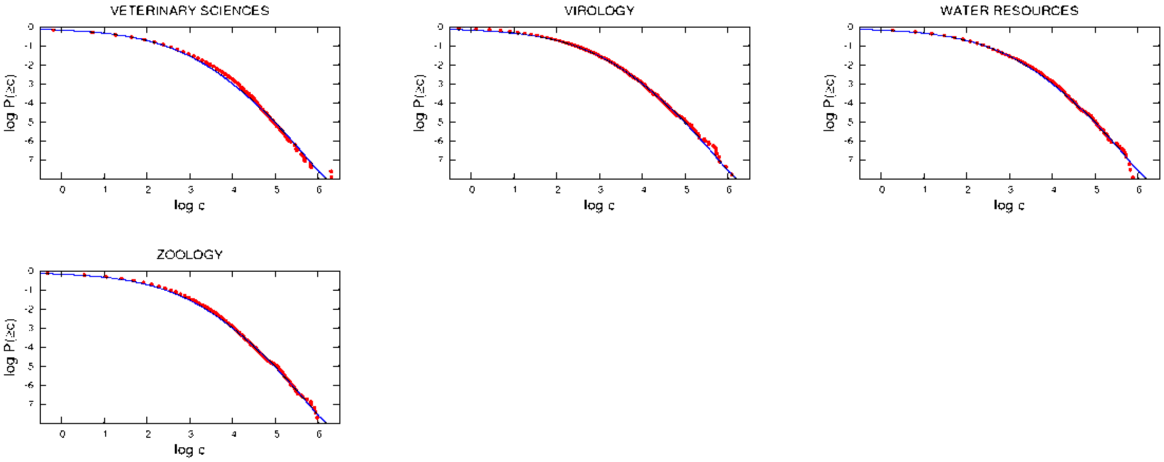

Figure S113: Publication year 2004.

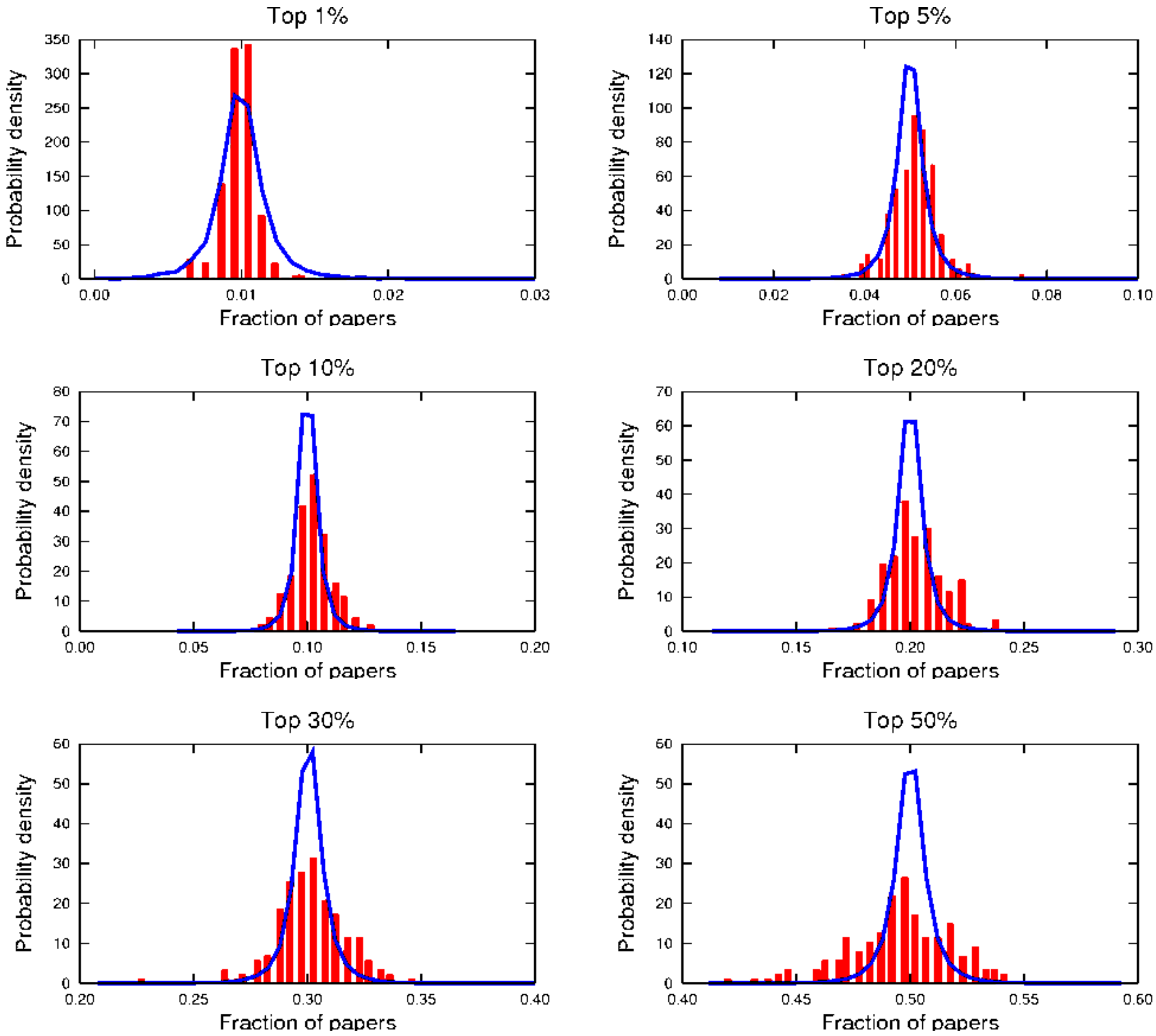

Figure S114: Publication year 2004.
