# Supplementary material for: A Reverse Engineering Approach to the Suppression of Citation Biases Reveals Universal Properties of Citation Distributions
Source: PLoS One. 2012 Mar 29;7(3):e33833. doi: 10.1371/journal.pone.0033833 (PMC3315498; doi:10.1371/journal.pone.0033833)
Supplement: Supporting Information S8 — Summary figures for all years of publication. (PDF) [file pone.0033833.s008.pdf]

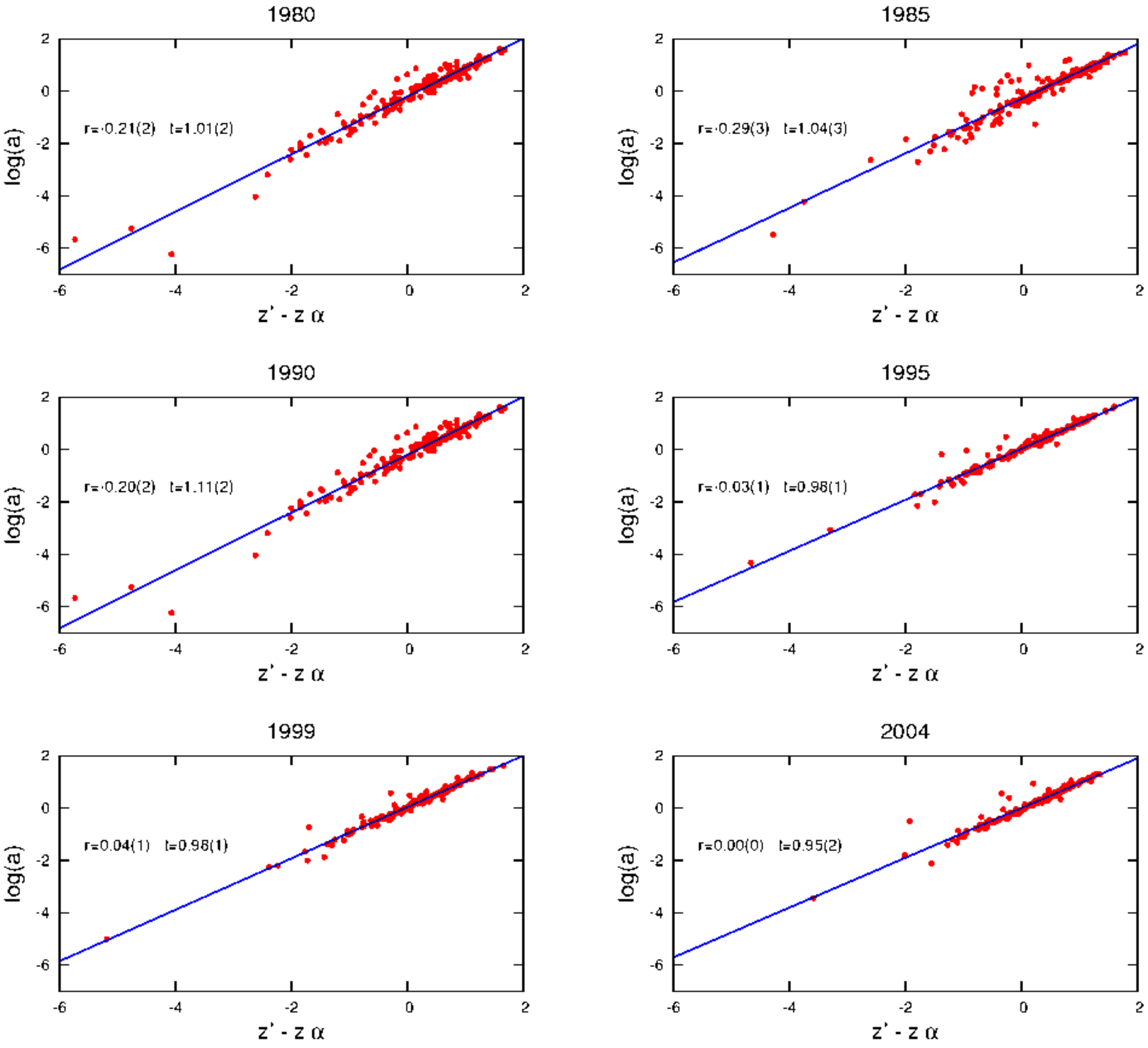


Figure S115: $\log(a) = r + t\,(z' - z\alpha)$.

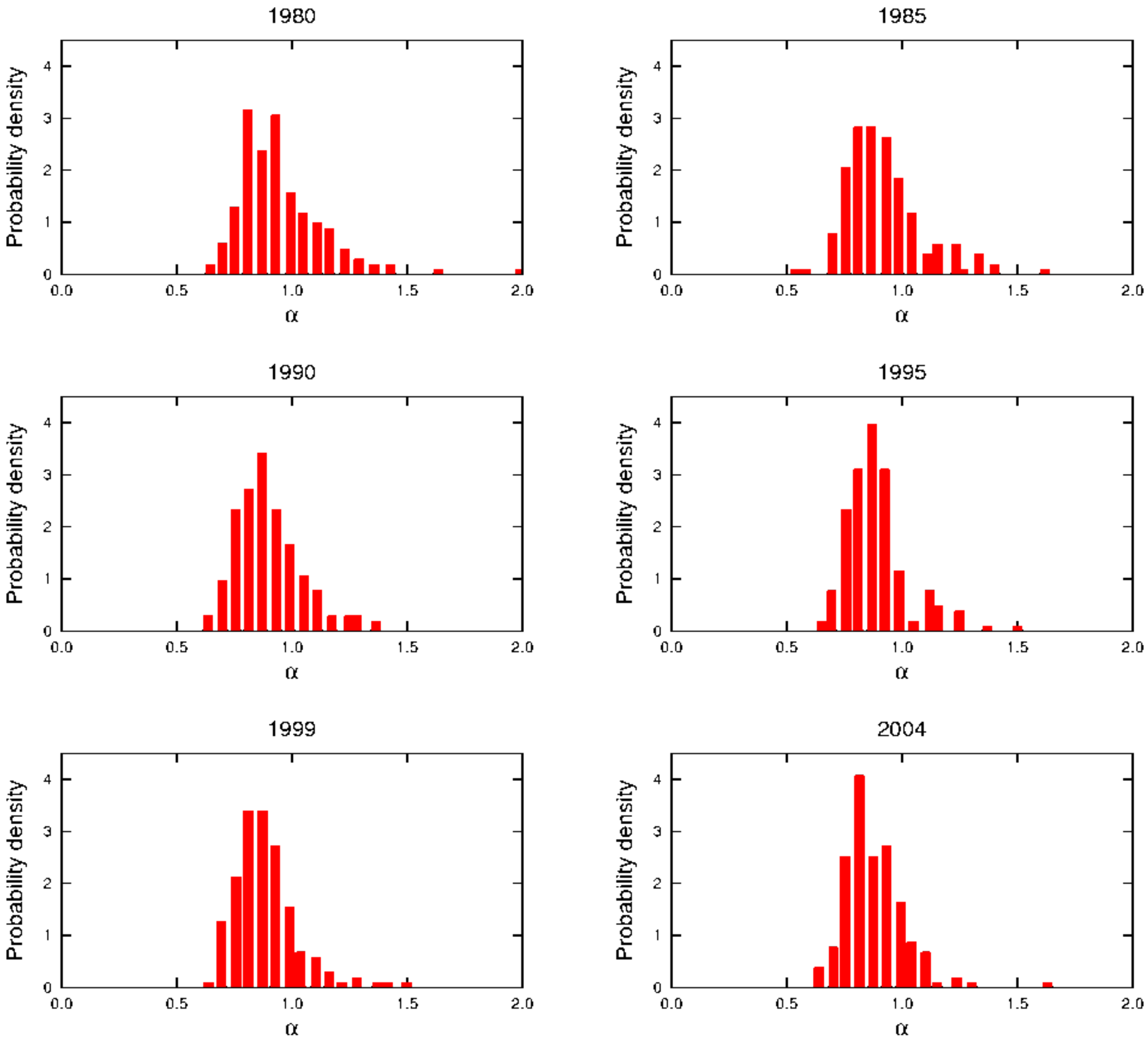


Figure S116: Probability density distribution of the transformation exponent $\alpha$ for various publication years.
