# Supplementary material for: A Reverse Engineering Approach to the Suppression of Citation Biases Reveals Universal Properties of Citation Distributions
Source: PLoS One. 2012 Mar 29;7(3):e33833. doi: 10.1371/journal.pone.0033833 (PMC3315498; doi:10.1371/journal.pone.0033833)
Supplement: Supporting Information S9 — Log-normal fit of the citation distributions. (PDF) [file pone.0033833.s009.pdf]

Fitting citation distributions with log-normal distributions

We fit the citation distributions of all subject-categories and publication years with log-normal distributions. We adopt two different ways for the determination of the parameters of the fitted log-normal distributions.

Parameters determined from the dataset

We consider the discrete log-normal distribution

$$P(c) = Q \, \frac{1}{c\, s\sqrt{2\pi}} \, e^{-[\log(c)-z]^2/\left(2s^2\right)}$$

for any value of $c > 0$. For $c = 0$, $P(0)$ is set equal to the fraction of papers with zero citations in the dataset that we are fitting. Q is the normalization factor for which $Q \sum_{c=1}^{C_{max}} P(c) = 1$. We determine the parameters of each log-normal distribution directly from data, by using the values of the average value $\langle c \rangle$ and the variance σ in the following equations

$$z = \log(\langle c \rangle) - s^2/2$$

and

$$s^2 = \log\left[1 + \left(\frac{\sigma}{\langle c \rangle}\right)^2\right] \; .$$

Best estimation of the parameters

We consider the discrete log-normal distribution

$$P(c) = Q \int_c^{c+1} dx \frac{1}{x\, s\sqrt{2\pi}} \, e^{-[\log(x)-z]^2/\left(2s^2\right)}$$

with Q equal to the normalization factor for which $Q \sum_{c=0}^{C_{max}} P(c) = 1$. We determine the distance between the theoretical distribution and the empirical distribution $P_{\rm emp}(c)$ using the Kolmogorov-Smirnov distance defined as

$$d = \max_c \left| P(\geq c) - P_{\rm emp}(\geq c) \right| \; . \qquad \text{(S1)}$$

The best estimates of z and s are determined by the values $\tilde{z}$ and $\tilde{s}$ for which the distance in Eq. S1 is minimized.

Statistical significance

We first determine the distance $d_{\rm emp}$ between the empirical distribution and the theoretical distribution using Eq. S1. We then generate $1,000$ artificial distributions of size N (N is the number of papers in the subject-category under consideration) and for each of them we estimate the Kolmogorov-Smirnov distance from the theoretical distribution. The p-value is calculated as the fraction of times that the distance of an artificial distribution is larger than $d_{\rm emp}$.

Subject-category	$\langle c\rangle$	σ	z	s	p	$\tilde{z}$	$\tilde{s}$	$\tilde{p}$
ACOUSTICS	17.61	41.19	1.93	1.37	0.00	1.53	1.97	0.03
AGRICULTURAL ECONOMICS & POLICY	6.96	19.00	0.87	1.46	0.00	0.77	1.66	0.29
AGRICULTURE, DAIRY & ANIMAL SCIENCE	14.68	28.98	1.89	1.26	0.00	1.69	1.66	0.00
AGRICULTURE, MULTIDISCIPLINARY	12.52	36.15	1.41	1.49	0.00	1.31	1.79	0.00
AGRONOMY	13.33	21.90	1.94	1.14	0.00	1.74	1.54	0.00
ALLERGY	20.99	29.50	2.50	1.04	0.04	2.20	1.64	0.07
ANATOMY & MORPHOLOGY	20.98	24.48	2.61	0.93	0.64	2.61	0.93	0.79
ANDROLOGY	12.80	15.29	2.11	0.94	0.16	2.01	1.14	0.93
ANESTHESIOLOGY	18.52	32.40	2.22	1.18	0.00	1.72	1.98	0.00
ASTRONOMY & ASTROPHYSICS	35.73	76.12	2.72	1.31	0.00	2.62	1.61	0.00
AUTOMATION & CONTROL SYSTEMS	12.07	31.86	1.45	1.44	0.00	0.75	2.34	0.02
BEHAVIORAL SCIENCES	24.87	45.12	2.48	1.21	0.00	2.18	1.71	0.00
BIOCHEMICAL RESEARCH METHODS	47.38	306.22	1.98	1.94	0.00	2.58	1.34	0.11
BIOCHEMISTRY & MOLECULAR BIOLOGY	42.99	149.40	2.48	1.60	0.00	2.88	1.30	0.00
BIODIVERSITY CONSERVATION	11.18	23.04	1.59	1.29	0.00	1.09	1.99	0.07
BIOLOGY	15.60	31.15	1.94	1.27	0.00	1.44	1.97	0.00
BIOPHYSICS	28.34	47.62	2.67	1.16	0.00	2.77	1.26	0.00
BIOTECHNOLOGY & APPLIED MICROBIOLOGY	21.62	35.55	2.42	1.14	0.00	2.32	1.45	0.00
CARDIAC & CARDIOVASCULAR SYSTEMS	34.24	107.17	2.34	1.54	0.00	2.14	2.14	0.00
CELL & TISSUE ENGINEERING	–	–	–	–	–	–	–	–
CELL BIOLOGY	42.42	90.49	2.89	1.31	0.00	2.89	1.31	0.01
CHEMISTRY, ANALYTICAL	30.14	215.83	1.43	1.99	0.00	2.23	1.49	0.00
CHEMISTRY, APPLIED	16.84	35.90	1.97	1.31	0.00	1.97	1.61	0.00
CHEMISTRY, INORGANIC & NUCLEAR	22.98	30.22	2.63	1.00	0.00	2.53	1.40	0.00
CHEMISTRY, MEDICINAL	16.44	31.31	2.03	1.24	0.00	2.03	1.24	0.00
CHEMISTRY, MULTIDISCIPLINARY	24.66	66.75	2.15	1.46	0.00	2.15	1.66	0.00
CHEMISTRY, ORGANIC	21.26	47.77	2.16	1.34	0.00	2.46	1.14	0.00
CHEMISTRY, PHYSICAL	24.57	53.98	2.32	1.33	0.00	2.22	1.73	0.00
CLINICAL NEUROLOGY	31.58	57.67	2.72	1.21	0.00	2.72	1.51	0.00
COMPUTER SCIENCE, ARTIFICIAL INTELLIGENCE	57.99	128.83	3.17	1.33	0.01	2.77	1.84	0.82
COMPUTER SCIENCE, CYBERNETICS	12.05	35.51	1.35	1.51	0.30	0.75	2.11	0.89
COMPUTER SCIENCE, HARDWARE & ARCHITECTURE	19.76	54.13	1.91	1.46	0.02	1.21	2.16	0.50
COMPUTER SCIENCE, INFORMATION SYSTEMS	14.92	46.12	1.52	1.54	0.12	1.12	1.93	0.57
COMPUTER SCIENCE, INTERDISCIPLINARY APPLICATIONS	15.27	56.32	1.38	1.64	0.00	1.28	1.84	0.17
COMPUTER SCIENCE, SOFTWARE ENGINEERING	17.34	42.41	1.88	1.39	0.01	1.28	2.10	0.25
COMPUTER SCIENCE, THEORY & METHODS	21.82	50.26	2.16	1.36	0.00	1.46	2.16	0.27
CONSTRUCTION & BUILDING TECHNOLOGY	8.37	10.26	1.67	0.96	0.03	1.27	1.66	0.35
CRITICAL CARE MEDICINE	14.85	20.62	2.16	1.03	0.00	1.76	1.74	0.06
CRYSTALLOGRAPHY	19.52	33.27	2.29	1.17	0.00	2.39	1.17	0.03
DENTISTRY, ORAL SURGERY & MEDICINE	13.72	24.08	1.92	1.19	0.00	1.42	2.09	0.00
DERMATOLOGY	17.26	27.22	2.22	1.12	0.00	1.92	1.72	0.00
DEVELOPMENTAL BIOLOGY	38.92	55.53	3.11	1.05	0.11	3.01	1.15	0.59
ECOLOGY	34.58	57.28	2.88	1.15	0.00	2.68	1.65	0.00
EDUCATION, SCIENTIFIC DISCIPLINES	3.72	10.91	0.18	1.50	0.00	0.18	1.50	0.26
ELECTROCHEMISTRY	28.28	53.94	2.57	1.24	0.00	2.57	1.34	0.04
EMERGENCY MEDICINE	6.38	11.69	1.12	1.21	0.00	0.52	2.11	0.04
ENDOCRINOLOGY & METABOLISM	36.50	49.86	3.07	1.02	0.00	3.07	1.22	0.00
ENERGY & FUELS	5.32	15.77	0.53	1.51	0.00	−0.37	2.41	0.01
ENGINEERING, AEROSPACE	6.32	17.18	0.78	1.46	0.00	0.38	1.96	0.48
ENGINEERING, BIOMEDICAL	18.95	56.15	1.80	1.51	0.02	1.60	1.71	0.06

Table S37: Publication year 1980.

Subject-category	$\langle c \rangle$	σ	z	s	p	$\tilde{z}$	$\tilde{s}$	$\tilde{p}$
ENGINEERING, CHEMICAL	14.33	29.95	1.82	1.30	0.00	1.12	2.20	0.00
ENGINEERING, CIVIL	6.92	16.54	0.98	1.38	0.00	0.28	2.28	0.01
ENGINEERING, ELECTRICAL & ELECTRONIC	14.87	53.66	1.38	1.62	0.00	1.18	1.93	0.00
ENGINEERING, ENVIRONMENTAL	21.55	39.31	2.34	1.21	0.01	1.84	2.01	0.02
ENGINEERING, GEOLOGICAL	17.52	33.62	2.09	1.24	0.05	1.89	1.74	0.19
ENGINEERING, INDUSTRIAL	15.20	47.56	1.53	1.54	0.37	1.13	1.94	0.77
ENGINEERING, MANUFACTURING	4.73	25.32	−0.14	1.84	0.27	−1.14	2.54	0.97
ENGINEERING, MARINE	0.60	2.44	−1.94	1.69	0.01	−1.74	1.99	0.95
ENGINEERING, MECHANICAL	8.94	20.20	1.29	1.35	0.00	0.89	1.94	0.02
ENGINEERING, MULTIDISCIPLINARY	5.74	21.19	0.40	1.64	0.00	−0.50	2.54	0.33
ENGINEERING, OCEAN	5.21	13.89	0.60	1.45	0.00	−0.40	2.35	0.11
ENGINEERING, PETROLEUM	0.29	1.69	−3.01	1.88	0.75	−2.11	1.88	0.20
ENTOMOLOGY	11.88	17.05	1.92	1.06	0.00	1.62	1.66	0.00
ENVIRONMENTAL SCIENCES	17.70	42.92	1.91	1.39	0.00	1.81	1.69	0.00
EVOLUTIONARY BIOLOGY	44.91	301.41	1.89	1.96	0.00	2.69	1.46	0.08
FISHERIES	30.63	97.47	2.22	1.55	0.00	2.52	1.55	0.04
FOOD SCIENCE & TECHNOLOGY	16.56	35.16	1.95	1.31	0.00	1.85	1.71	0.00
FORESTRY	11.74	22.21	1.70	1.23	0.00	1.00	2.13	0.05
GASTROENTEROLOGY & HEPATOLOGY	30.52	54.84	2.70	1.20	0.00	2.40	1.80	0.00
GENETICS & HEREDITY	34.32	194.85	1.78	1.87	0.00	2.58	1.37	0.00
GEOCHEMISTRY & GEOPHYSICS	33.18	57.50	2.81	1.18	0.00	2.51	1.68	0.00
GEOGRAPHY, PHYSICAL	22.24	32.19	2.54	1.06	0.17	2.34	1.46	0.24
GEOLOGY	23.72	35.65	2.58	1.09	0.05	2.38	1.49	0.29
GEOSCIENCES, MULTIDISCIPLINARY	23.00	38.15	2.47	1.15	0.00	2.27	1.55	0.00
GERIATRICS & GERONTOLOGY	17.66	28.98	2.22	1.14	0.01	1.92	1.64	0.17
HEALTH CARE SCIENCES & SERVICES	7.47	18.70	1.02	1.41	0.00	0.72	1.71	0.39
HEMATOLOGY	39.13	117.18	2.52	1.52	0.00	2.62	1.82	0.00
HISTORY & PHILOSOPHY OF SCIENCE	7.90	14.15	1.35	1.20	0.01	1.05	1.60	0.30
IMAGING SCIENCE & PHOTOGRAPHIC TECHNOLOGY	17.98	32.52	2.16	1.20	0.95	1.76	1.61	0.81
IMMUNOLOGY	35.61	71.57	2.76	1.27	0.00	2.66	1.47	0.00
INFECTIOUS DISEASES	29.48	48.89	2.72	1.15	0.00	2.32	1.95	0.00
INSTRUMENTS & INSTRUMENTATION	4.98	14.66	0.47	1.51	0.00	−0.33	2.41	0.02
INTEGRATIVE & COMPLEMENTARY MEDICINE	6.21	9.41	1.23	1.09	0.33	1.03	1.49	0.87
LIMNOLOGY	65.88	216.48	2.95	1.57	0.01	3.15	1.47	0.30
MARINE & FRESHWATER BIOLOGY	26.70	65.38	2.31	1.40	0.00	2.61	1.30	0.00
MATERIALS SCIENCE, BIOMATERIALS	31.11	57.01	2.70	1.21	0.46	2.40	1.31	0.84
MATERIALS SCIENCE, CERAMICS	19.73	44.60	2.08	1.35	0.00	1.48	2.24	0.00
MATERIALS SCIENCE, CHARACTERIZATION & TESTING	4.66	12.62	0.48	1.46	0.00	0.28	1.75	0.67
MATERIALS SCIENCE, COATINGS & FILMS	24.02	37.56	2.56	1.11	0.05	2.46	1.41	0.05
MATERIALS SCIENCE, COMPOSITES	21.32	24.91	2.63	0.93	0.29	2.33	1.23	0.97
MATERIALS SCIENCE, MULTIDISCIPLINARY	13.77	30.63	1.73	1.33	0.00	1.43	1.84	0.00
MATERIALS SCIENCE, PAPER & WOOD	6.26	14.70	0.90	1.37	0.01	0.40	1.97	0.63
MATERIALS SCIENCE, TEXTILES	4.83	7.03	1.01	1.07	0.00	0.61	1.67	0.24
MATHEMATICAL & COMPUTATIONAL BIOLOGY	20.33	33.69	2.35	1.15	0.00	1.95	1.65	0.10
MATHEMATICS	10.44	24.05	1.43	1.36	0.00	1.03	1.96	0.00
MATHEMATICS, APPLIED	14.07	38.69	1.57	1.47	0.00	1.27	1.87	0.00
MATHEMATICS, INTERDISCIPLINARY APPLICATIONS	22.21	170.92	1.05	2.02	0.00	1.35	2.02	0.03
MECHANICS	16.99	36.59	1.97	1.32	0.00	1.57	1.92	0.00
MEDICAL ETHICS	2.34	4.56	0.07	1.25	0.01	−0.13	1.65	0.83
MEDICAL INFORMATICS	10.43	19.40	1.60	1.22	0.37	1.30	1.62	0.95

Table S38: Publication year 1980.

Subject-category	$\langle c \rangle$	σ	z	s	p	$\tilde{z}$	$\tilde{s}$	$\tilde{p}$
MEDICAL LABORATORY TECHNOLOGY	18.01	26.89	2.30	1.08	0.01	2.20	1.38	0.00
MEDICINE, GENERAL & INTERNAL	17.08	56.12	1.60	1.57	0.00	0.70	2.47	0.00
MEDICINE, LEGAL	5.72	8.24	1.18	1.06	0.00	0.88	1.56	0.18
MEDICINE, RESEARCH & EXPERIMENTAL	30.73	63.18	2.60	1.28	0.00	1.80	2.19	0.00
METALLURGY & METALLURGICAL ENGINEERING	5.64	12.70	0.83	1.34	0.00	−0.07	2.24	0.00
METEOROLOGY & ATMOSPHERIC SCIENCES	34.36	78.07	2.63	1.35	0.16	2.43	1.75	0.04
MICROBIOLOGY	29.01	49.94	2.68	1.17	0.00	2.58	1.57	0.00
MICROSCOPY	10.29	19.32	1.58	1.23	0.26	1.28	1.63	0.84
MINERALOGY	32.06	53.24	2.81	1.15	0.21	2.71	1.35	0.08
MINING & MINERAL PROCESSING	9.43	20.93	1.35	1.33	0.00	0.65	2.23	0.00
MULTIDISCIPLINARY SCIENCES	55.30	184.64	2.76	1.58	0.00	1.86	2.48	0.00
MYCOLOGY	9.99	15.02	1.71	1.09	0.49	1.51	1.39	0.57
NANOSCIENCE & NANOTECHNOLOGY	–	–	–	–	–	–	–	–
NEUROIMAGING	18.18	23.23	2.42	0.98	0.07	2.22	1.48	0.37
NEUROSCIENCES	40.03	64.61	3.05	1.13	0.00	2.95	1.43	0.00
NUCLEAR SCIENCE & TECHNOLOGY	9.08	16.35	1.48	1.20	0.00	1.18	1.70	0.00
NURSING	2.80	6.26	0.13	1.34	0.00	−0.17	1.74	0.90
NUTRITION & DIETETICS	24.43	42.70	2.50	1.18	0.00	2.20	1.88	0.00
OBSTETRICS & GYNECOLOGY	23.67	36.95	2.55	1.11	0.01	2.35	1.51	0.00
OCEANOGRAPHY	32.97	113.07	2.22	1.60	0.00	2.12	2.10	0.00
ONCOLOGY	32.87	49.47	2.90	1.09	0.00	2.80	1.39	0.00
OPERATIONS RESEARCH & MANAGEMENT SCIENCE	13.42	32.30	1.64	1.39	0.00	1.34	1.78	0.03
OPHTHALMOLOGY	21.77	40.41	2.33	1.22	0.00	2.23	1.62	0.00
OPTICS	23.72	47.61	2.36	1.27	0.00	2.26	1.57	0.00
ORNITHOLOGY	21.85	65.79	1.93	1.52	0.00	1.93	1.72	0.16
ORTHOPEDICS	23.92	47.01	2.38	1.26	0.06	2.08	1.76	0.01
OTORHINOLARYNGOLOGY	18.38	28.13	2.31	1.10	0.01	2.21	1.50	0.00
PALEONTOLOGY	22.79	42.96	2.37	1.23	0.73	2.27	1.43	0.25
PARASITOLOGY	16.61	20.20	2.36	0.95	0.00	2.26	1.25	0.01
PATHOLOGY	23.80	37.30	2.55	1.11	0.00	2.35	1.61	0.00
PEDIATRICS	21.39	36.61	2.38	1.17	0.00	1.98	1.97	0.00
PERIPHERAL VASCULAR DISEASE	45.33	136.36	2.66	1.52	0.00	2.76	1.82	0.00
PHARMACOLOGY & PHARMACY	24.75	37.91	2.60	1.10	0.00	2.50	1.40	0.00
PHYSICS, APPLIED	19.33	44.80	2.04	1.36	0.00	1.94	1.66	0.00
PHYSICS, ATOMIC, MOLECULAR & CHEMICAL	33.83	111.08	2.29	1.57	0.00	2.69	1.27	0.00
PHYSICS, CONDENSED MATTER	23.72	55.58	2.23	1.37	0.00	2.23	1.57	0.00
PHYSICS, FLUIDS & PLASMAS	23.08	40.62	2.43	1.19	0.00	2.13	1.69	0.01
PHYSICS, MATHEMATICAL	23.10	68.80	2.00	1.51	0.04	1.60	1.91	0.05
PHYSICS, MULTIDISCIPLINARY	26.89	164.26	1.47	1.91	0.00	1.67	1.91	0.00
PHYSICS, NUCLEAR	21.34	57.32	2.01	1.45	0.00	2.21	1.55	0.00
PHYSICS, PARTICLES & FIELDS	36.32	93.72	2.57	1.43	0.08	2.27	1.83	0.11
PHYSIOLOGY	43.28	77.33	3.05	1.20	0.00	3.15	1.30	0.00
PLANT SCIENCES	20.36	49.52	2.05	1.39	0.00	2.15	1.49	0.00
POLYMER SCIENCE	24.10	62.09	2.17	1.42	0.00	2.37	1.42	0.00
PRIMARY HEALTH CARE	3.46	7.85	0.33	1.35	0.00	−0.17	2.05	0.42
PSYCHIATRY	31.38	63.60	2.63	1.28	0.00	2.33	1.88	0.00
PSYCHOLOGY	44.07	209.75	2.20	1.78	0.00	2.40	1.58	0.25
PUBLIC, ENVIRONMENTAL & OCCUPATIONAL HEALTH	18.56	34.93	2.16	1.23	0.00	1.76	1.93	0.00
RADIOLOGY, NUCLEAR MEDICINE & MEDICAL IMAGING	21.08	37.86	2.33	1.20	0.00	2.03	1.80	0.00
REHABILITATION	11.61	21.06	1.72	1.21	0.03	1.22	1.91	0.35

Table S39: Publication year 1980.

Subject-category	$\langle c \rangle$	σ	z	s	p	$\tilde{z}$	$\tilde{s}$	$\tilde{p}$
REMOTE SENSING	17.46	32.35	2.11	1.22	0.50	1.81	1.62	0.60
REPRODUCTIVE BIOLOGY	27.35	36.05	2.81	1.00	0.02	2.71	1.10	0.11
RESPIRATORY SYSTEM	21.30	35.02	2.40	1.14	0.00	2.00	1.74	0.00
RHEUMATOLOGY	28.77	97.50	2.10	1.59	0.00	2.20	1.99	0.00
ROBOTICS	–	–	–	–	–	–	–	–
SOIL SCIENCE	26.60	146.63	1.56	1.86	0.00	2.26	1.56	0.01
SPECTROSCOPY	22.50	43.04	2.34	1.24	0.00	2.44	1.24	0.09
SPORT SCIENCES	26.73	44.70	2.62	1.15	0.00	2.32	1.66	0.02
STATISTICS & PROBABILITY	25.78	160.11	1.41	1.92	0.00	1.81	1.82	0.09
SUBSTANCE ABUSE	24.37	39.52	2.55	1.14	0.09	2.35	1.44	0.94
SURGERY	24.04	45.43	2.42	1.23	0.00	2.22	1.73	0.00
TELECOMMUNICATIONS	15.33	93.89	0.90	1.91	0.00	1.00	2.01	0.09
THERMODYNAMICS	11.99	22.03	1.75	1.22	0.00	1.35	1.81	0.03
TOXICOLOGY	18.51	23.51	2.44	0.98	0.00	2.34	1.18	0.06
TRANSPLANTATION	15.76	29.17	2.01	1.22	0.33	1.71	1.72	0.13
TRANSPORTATION SCIENCE & TECHNOLOGY	9.60	36.54	0.89	1.66	0.02	0.39	2.26	0.62
TROPICAL MEDICINE	15.67	19.72	2.28	0.97	0.00	1.98	1.57	0.00
UROLOGY & NEPHROLOGY	19.91	29.82	2.40	1.09	0.00	2.00	1.78	0.00
VETERINARY SCIENCES	11.72	17.81	1.86	1.10	0.00	1.56	1.79	0.00
VIROLOGY	27.90	38.63	2.79	1.03	0.00	2.79	1.14	0.01
WATER RESOURCES	19.82	63.13	1.78	1.55	0.00	1.78	1.75	0.05
ZOOLOGY	25.08	48.96	2.44	1.25	0.04	2.34	1.45	0.00
**TOTAL**	24.55	80.00	1.97	1.57	0.00	1.97	1.97	0.00

Table S40: Publication year 1980.

Subject-category	$\langle c \rangle$	σ	z	s	p	$\tilde{z}$	$\tilde{s}$	$\tilde{p}$
ACOUSTICS	14.98	26.03	2.01	1.18	0.00	1.81	1.68	0.00
AGRICULTURAL ECONOMICS & POLICY	7.26	10.35	1.43	1.05	0.00	1.13	1.55	0.09
AGRICULTURE, DAIRY & ANIMAL SCIENCE	14.05	21.51	2.04	1.10	0.00	1.84	1.60	0.00
AGRICULTURE, MULTIDISCIPLINARY	12.12	24.95	1.67	1.29	0.00	1.17	1.89	0.01
AGRONOMY	13.72	19.97	2.05	1.07	0.00	1.85	1.47	0.00
ALLERGY	20.73	31.50	2.43	1.10	0.01	2.13	1.59	0.06
ANATOMY & MORPHOLOGY	16.05	15.25	2.45	0.80	0.09	2.45	1.00	0.25
ANDROLOGY	17.04	16.85	2.49	0.82	0.22	2.49	1.02	0.53
ANESTHESIOLOGY	17.23	37.32	1.98	1.32	0.00	1.48	2.12	0.00
ASTRONOMY & ASTROPHYSICS	33.02	68.30	2.67	1.29	0.00	2.57	1.59	0.00
AUTOMATION & CONTROL SYSTEMS	13.83	34.70	1.63	1.41	0.00	1.03	2.11	0.04
BEHAVIORAL SCIENCES	24.66	38.51	2.59	1.11	0.00	2.39	1.61	0.00
BIOCHEMICAL RESEARCH METHODS	35.71	332.22	1.34	2.11	0.00	2.24	1.41	0.00
BIOCHEMISTRY & MOLECULAR BIOLOGY	45.57	189.52	2.37	1.71	0.00	2.97	1.40	0.00
BIODIVERSITY CONSERVATION	10.17	17.34	1.64	1.17	0.00	1.14	1.97	0.00
BIOLOGY	16.38	37.78	1.87	1.36	0.00	1.57	1.86	0.00
BIOPHYSICS	30.13	47.40	2.78	1.12	0.01	2.78	1.22	0.01
BIOTECHNOLOGY & APPLIED MICROBIOLOGY	24.57	41.12	2.53	1.16	0.00	2.53	1.26	0.00
CARDIAC & CARDIOVASCULAR SYSTEMS	30.71	57.56	2.67	1.23	0.00	2.37	1.73	0.00
CELL & TISSUE ENGINEERING	–	–	–	–	–	–	–	–
CELL BIOLOGY	49.91	96.31	3.13	1.24	0.00	3.03	1.45	0.00
CHEMISTRY, ANALYTICAL	21.30	197.86	0.82	2.11	0.00	1.72	1.51	0.00
CHEMISTRY, APPLIED	16.95	29.01	2.15	1.17	0.00	1.95	1.67	0.00
CHEMISTRY, INORGANIC & NUCLEAR	21.56	28.81	2.56	1.01	0.00	2.36	1.41	0.00
CHEMISTRY, MEDICINAL	15.60	34.95	1.85	1.34	0.00	2.05	1.34	0.00
CHEMISTRY, MULTIDISCIPLINARY	22.88	141.96	1.29	1.92	0.00	1.89	1.92	0.00
CHEMISTRY, ORGANIC	22.31	33.14	2.52	1.08	0.00	2.52	1.18	0.00
CHEMISTRY, PHYSICAL	22.21	46.32	2.26	1.30	0.00	2.16	1.69	0.00
CLINICAL NEUROLOGY	28.49	51.44	2.62	1.20	0.00	2.52	1.60	0.00
COMPUTER SCIENCE, ARTIFICIAL INTELLIGENCE	29.38	59.46	2.57	1.28	0.14	2.27	1.57	0.81
COMPUTER SCIENCE, CYBERNETICS	27.90	177.56	1.47	1.93	0.25	0.97	1.93	0.87
COMPUTER SCIENCE, HARDWARE & ARCHITECTURE	17.68	51.74	1.74	1.50	0.00	1.14	2.20	0.08
COMPUTER SCIENCE, INFORMATION SYSTEMS	17.77	67.67	1.51	1.66	0.11	1.11	2.05	0.43
COMPUTER SCIENCE, INTERDISCIPLINARY APPLICATIONS	11.53	34.55	1.30	1.52	0.00	1.20	1.82	0.05
COMPUTER SCIENCE, SOFTWARE ENGINEERING	15.64	51.42	1.52	1.57	0.00	0.82	2.37	0.02
COMPUTER SCIENCE, THEORY & METHODS	20.19	57.86	1.90	1.49	0.26	1.30	2.09	0.08
CONSTRUCTION & BUILDING TECHNOLOGY	9.48	20.00	1.40	1.30	0.00	1.20	1.60	0.08
CRITICAL CARE MEDICINE	21.74	211.46	0.80	2.14	0.00	1.60	1.73	0.01
CRYSTALLOGRAPHY	15.47	101.43	0.85	1.94	0.00	1.75	1.35	0.00
DENTISTRY, ORAL SURGERY & MEDICINE	13.67	22.66	1.95	1.15	0.00	1.45	2.05	0.00
DERMATOLOGY	15.60	23.78	2.15	1.10	0.00	1.85	1.70	0.00
DEVELOPMENTAL BIOLOGY	33.83	45.47	3.01	1.01	0.09	2.91	1.22	0.50
ECOLOGY	37.84	277.60	1.63	2.00	0.00	2.53	1.60	0.00
EDUCATION, SCIENTIFIC DISCIPLINES	5.51	15.97	0.59	1.50	0.00	0.49	1.60	0.08
ELECTROCHEMISTRY	21.95	34.76	2.46	1.12	0.20	2.46	1.22	0.03
EMERGENCY MEDICINE	10.52	19.02	1.63	1.20	0.00	1.13	1.91	0.05
ENDOCRINOLOGY & METABOLISM	34.10	126.32	2.18	1.64	0.00	2.78	1.34	0.00
ENERGY & FUELS	5.21	12.34	0.71	1.37	0.00	0.11	2.17	0.01
ENGINEERING, AEROSPACE	6.73	26.15	0.52	1.67	0.00	0.42	1.87	0.15
ENGINEERING, BIOMEDICAL	17.68	39.33	1.98	1.33	0.00	1.78	1.73	0.01

Table S41: Publication year 1985.

Subject-category	$\langle c \rangle$	σ	z	s	p	$\tilde{z}$	$\tilde{s}$	$\tilde{p}$
ENGINEERING, CHEMICAL	11.35	23.25	1.61	1.28	0.00	0.81	2.18	0.00
ENGINEERING, CIVIL	7.27	16.58	1.07	1.35	0.00	0.57	2.05	0.00
ENGINEERING, ELECTRICAL & ELECTRONIC	12.39	32.75	1.48	1.44	0.00	1.08	1.94	0.00
ENGINEERING, ENVIRONMENTAL	20.03	43.01	2.13	1.31	0.03	1.63	1.81	0.04
ENGINEERING, GEOLOGICAL	15.68	31.99	1.93	1.28	0.01	1.53	1.88	0.24
ENGINEERING, INDUSTRIAL	8.80	17.93	1.35	1.28	0.00	0.85	1.98	0.04
ENGINEERING, MANUFACTURING	5.72	11.97	0.90	1.30	0.00	0.10	2.20	0.26
ENGINEERING, MARINE	0.97	3.62	−1.37	1.64	0.00	−2.07	2.54	0.76
ENGINEERING, MECHANICAL	10.53	22.59	1.49	1.31	0.00	1.19	1.81	0.00
ENGINEERING, MULTIDISCIPLINARY	7.89	29.94	0.70	1.65	0.00	0.10	2.35	0.09
ENGINEERING, OCEAN	6.97	17.76	0.94	1.42	0.00	0.34	2.22	0.48
ENGINEERING, PETROLEUM	1.39	7.40	−1.36	1.84	0.01	−1.46	2.04	0.94
ENTOMOLOGY	11.54	15.54	1.93	1.01	0.00	1.73	1.52	0.00
ENVIRONMENTAL SCIENCES	18.40	34.67	2.15	1.23	0.00	1.85	1.73	0.00
EVOLUTIONARY BIOLOGY	54.22	508.04	1.75	2.12	0.00	2.45	1.82	0.00
FISHERIES	24.94	29.04	2.79	0.93	0.00	2.59	1.33	0.00
FOOD SCIENCE & TECHNOLOGY	16.73	26.02	2.20	1.11	0.00	2.00	1.61	0.00
FORESTRY	12.52	20.64	1.87	1.14	0.00	1.27	2.05	0.00
GASTROENTEROLOGY & HEPATOLOGY	29.22	45.68	2.76	1.11	0.00	2.46	1.71	0.00
GENETICS & HEREDITY	39.67	391.03	1.39	2.14	0.00	2.29	1.44	0.00
GEOCHEMISTRY & GEOPHYSICS	30.50	52.71	2.73	1.17	0.00	2.43	1.77	0.00
GEOGRAPHY, PHYSICAL	16.99	35.38	2.00	1.29	0.00	1.80	1.69	0.01
GEOLOGY	24.64	39.38	2.57	1.13	0.01	2.27	1.73	0.01
GEOSCIENCES, MULTIDISCIPLINARY	19.58	37.51	2.20	1.24	0.00	2.00	1.74	0.00
GERIATRICS & GERONTOLOGY	19.89	39.82	2.18	1.27	0.00	2.08	1.57	0.00
HEALTH CARE SCIENCES & SERVICES	13.26	24.35	1.85	1.22	0.01	1.35	1.92	0.04
HEMATOLOGY	37.74	71.33	2.87	1.23	0.00	2.57	1.73	0.00
HISTORY & PHILOSOPHY OF SCIENCE	7.58	12.66	1.36	1.15	0.32	1.16	1.45	0.60
IMAGING SCIENCE & PHOTOGRAPHIC TECHNOLOGY	20.22	58.79	1.88	1.50	0.04	1.68	1.80	0.29
IMMUNOLOGY	33.00	64.32	2.71	1.25	0.00	2.61	1.55	0.00
INFECTIOUS DISEASES	25.49	44.29	2.54	1.18	0.00	2.24	1.78	0.00
INSTRUMENTS & INSTRUMENTATION	10.78	42.66	0.97	1.68	0.00	0.97	1.98	0.00
INTEGRATIVE & COMPLEMENTARY MEDICINE	7.98	10.65	1.57	1.01	0.37	1.27	1.61	0.72
LIMNOLOGY	41.31	57.39	3.18	1.04	0.01	2.98	1.44	0.24
MARINE & FRESHWATER BIOLOGY	25.82	34.22	2.74	1.00	0.00	2.64	1.31	0.00
MATERIALS SCIENCE, BIOMATERIALS	25.70	42.99	2.58	1.15	0.15	2.28	1.36	0.75
MATERIALS SCIENCE, CERAMICS	17.26	39.17	1.94	1.35	0.00	1.54	1.85	0.00
MATERIALS SCIENCE, CHARACTERIZATION & TESTING	3.02	15.27	−0.53	1.81	0.00	−0.63	2.21	0.66
MATERIALS SCIENCE, COATINGS & FILMS	20.53	33.34	2.38	1.14	0.11	2.28	1.34	0.01
MATERIALS SCIENCE, COMPOSITES	24.80	32.35	2.71	0.99	0.03	2.51	1.30	0.97
MATERIALS SCIENCE, MULTIDISCIPLINARY	14.20	32.21	1.75	1.35	0.00	1.55	1.85	0.00
MATERIALS SCIENCE, PAPER & WOOD	5.51	11.60	0.86	1.30	0.00	0.36	2.00	0.08
MATERIALS SCIENCE, TEXTILES	4.97	7.49	1.01	1.09	0.04	0.81	1.39	0.26
MATHEMATICAL & COMPUTATIONAL BIOLOGY	17.50	37.43	2.00	1.31	0.10	1.70	1.81	0.05
MATHEMATICS	8.88	23.03	1.16	1.43	0.00	0.86	1.83	0.00
MATHEMATICS, APPLIED	12.62	32.40	1.52	1.42	0.00	1.22	1.82	0.01
MATHEMATICS, INTERDISCIPLINARY APPLICATIONS	23.44	74.05	1.96	1.55	0.50	1.56	1.95	0.20
MECHANICS	16.92	34.86	2.00	1.29	0.00	1.60	1.79	0.00
MEDICAL ETHICS	4.35	9.58	0.59	1.33	0.00	0.09	2.23	0.31
MEDICAL INFORMATICS	13.14	38.10	1.46	1.50	0.04	1.36	1.60	0.39

Table S42: Publication year 1985.

Subject-category	$\langle c \rangle$	σ	z	s	p	$\tilde{z}$	$\tilde{s}$	$\tilde{p}$
MEDICAL LABORATORY TECHNOLOGY	16.10	27.32	2.10	1.17	0.00	2.00	1.46	0.00
MEDICINE, GENERAL & INTERNAL	19.27	77.67	1.53	1.69	0.00	0.73	2.49	0.00
MEDICINE, LEGAL	8.72	13.12	1.58	1.09	0.00	1.28	1.59	0.06
MEDICINE, RESEARCH & EXPERIMENTAL	31.20	71.65	2.52	1.36	0.00	1.72	2.26	0.00
METALLURGY & METALLURGICAL ENGINEERING	6.75	13.40	1.11	1.26	0.00	0.31	2.16	0.00
METEOROLOGY & ATMOSPHERIC SCIENCES	26.52	44.24	2.61	1.15	0.00	2.41	1.55	0.00
MICROBIOLOGY	28.19	45.52	2.70	1.13	0.00	2.60	1.43	0.00
MICROSCOPY	17.16	24.55	2.29	1.05	0.13	1.99	1.56	0.34
MINERALOGY	27.08	38.82	2.74	1.06	0.02	2.64	1.26	0.23
MINING & MINERAL PROCESSING	7.82	16.15	1.23	1.29	0.00	0.73	2.09	0.00
MULTIDISCIPLINARY SCIENCES	76.09	212.95	3.24	1.48	0.00	2.24	2.38	0.00
MYCOLOGY	9.02	12.34	1.67	1.02	0.03	1.37	1.53	0.15
NANOSCIENCE & NANOTECHNOLOGY	22.78	27.48	2.68	0.95	0.01	2.48	1.35	0.42
NEUROIMAGING	19.20	24.65	2.47	0.98	0.23	2.37	1.39	0.15
NEUROSCIENCES	39.93	71.95	2.96	1.20	0.00	2.96	1.40	0.00
NUCLEAR SCIENCE & TECHNOLOGY	8.24	32.71	0.70	1.68	0.00	1.00	1.68	0.00
NURSING	5.83	16.85	0.64	1.50	0.00	0.34	1.99	0.17
NUTRITION & DIETETICS	20.76	41.22	2.23	1.26	0.00	2.13	1.57	0.00
OBSTETRICS & GYNECOLOGY	19.36	32.78	2.29	1.16	0.00	1.99	1.76	0.00
OCEANOGRAPHY	31.72	45.41	2.90	1.06	0.00	2.60	1.75	0.00
ONCOLOGY	32.25	51.67	2.84	1.13	0.00	2.74	1.43	0.00
OPERATIONS RESEARCH & MANAGEMENT SCIENCE	13.95	32.33	1.71	1.36	0.00	1.41	1.86	0.01
OPHTHALMOLOGY	20.40	31.54	2.41	1.10	0.00	2.11	1.71	0.00
OPTICS	24.71	77.39	2.02	1.54	0.00	2.22	1.54	0.00
ORNITHOLOGY	16.34	20.57	2.32	0.97	0.00	2.12	1.47	0.07
ORTHOPEDICS	20.66	39.16	2.27	1.23	0.00	1.97	1.73	0.00
OTORHINOLARYNGOLOGY	15.50	23.73	2.14	1.10	0.00	2.04	1.50	0.00
PALEONTOLOGY	18.97	27.32	2.38	1.06	0.06	2.28	1.36	0.56
PARASITOLOGY	17.25	23.18	2.33	1.01	0.09	2.23	1.22	0.08
PATHOLOGY	25.17	44.65	2.51	1.19	0.00	2.31	1.59	0.00
PEDIATRICS	19.64	33.15	2.30	1.16	0.00	2.00	1.76	0.00
PERIPHERAL VASCULAR DISEASE	38.89	67.62	2.96	1.18	0.00	2.66	1.68	0.00
PHARMACOLOGY & PHARMACY	21.65	39.01	2.35	1.20	0.00	2.35	1.40	0.00
PHYSICS, APPLIED	19.26	39.60	2.13	1.28	0.00	2.03	1.59	0.00
PHYSICS, ATOMIC, MOLECULAR & CHEMICAL	33.17	151.49	1.96	1.75	0.00	2.56	1.36	0.00
PHYSICS, CONDENSED MATTER	26.35	62.70	2.32	1.38	0.00	2.42	1.48	0.00
PHYSICS, FLUIDS & PLASMAS	22.49	39.08	2.42	1.18	0.07	2.22	1.68	0.00
PHYSICS, MATHEMATICAL	19.06	61.38	1.73	1.56	0.00	1.63	1.86	0.01
PHYSICS, MULTIDISCIPLINARY	27.89	95.20	2.06	1.59	0.00	1.86	1.99	0.00
PHYSICS, NUCLEAR	20.37	76.11	1.66	1.65	0.00	1.96	1.55	0.00
PHYSICS, PARTICLES & FIELDS	29.46	92.06	2.19	1.54	0.58	1.99	1.74	0.02
PHYSIOLOGY	38.30	61.08	3.01	1.12	0.00	2.91	1.52	0.00
PLANT SCIENCES	18.96	29.75	2.32	1.11	0.00	2.22	1.41	0.00
POLYMER SCIENCE	23.65	49.17	2.33	1.29	0.00	2.33	1.39	0.00
PRIMARY HEALTH CARE	3.87	17.53	−0.18	1.75	0.00	−0.08	1.95	0.46
PSYCHIATRY	29.31	62.10	2.53	1.30	0.00	2.33	1.81	0.00
PSYCHOLOGY	35.48	87.98	2.59	1.40	0.00	2.59	1.50	0.01
PUBLIC, ENVIRONMENTAL & OCCUPATIONAL HEALTH	21.04	54.74	2.02	1.43	0.00	1.82	1.83	0.00
RADIOLOGY, NUCLEAR MEDICINE & MEDICAL IMAGING	20.58	38.04	2.28	1.22	0.00	1.98	1.72	0.00
REHABILITATION	14.12	34.84	1.67	1.40	0.00	1.37	1.90	0.11

Table S43: Publication year 1985.

Subject-category	$\langle c \rangle$	σ	z	s	p	$\tilde{z}$	$\tilde{s}$	$\tilde{p}$
REMOTE SENSING	21.10	55.19	2.02	1.44	0.01	1.82	1.84	0.08
REPRODUCTIVE BIOLOGY	22.81	32.23	2.58	1.05	0.00	2.48	1.45	0.00
RESPIRATORY SYSTEM	19.14	33.60	2.25	1.19	0.00	1.85	1.89	0.00
RHEUMATOLOGY	22.40	39.06	2.41	1.18	0.15	2.21	1.68	0.00
ROBOTICS	39.95	88.91	2.80	1.33	0.34	1.80	2.24	0.97
SOIL SCIENCE	20.52	43.75	2.16	1.31	0.00	2.26	1.51	0.00
SPECTROSCOPY	18.56	92.17	1.30	1.80	0.00	2.00	1.40	0.00
SPORT SCIENCES	27.90	44.30	2.70	1.12	0.00	2.40	1.72	0.00
STATISTICS & PROBABILITY	19.42	59.66	1.79	1.53	0.08	1.39	2.03	0.02
SUBSTANCE ABUSE	20.19	25.42	2.53	0.97	0.28	2.43	1.27	0.29
SURGERY	21.32	37.87	2.35	1.19	0.00	2.05	1.79	0.00
TELECOMMUNICATIONS	10.89	34.32	1.19	1.55	0.00	0.69	2.25	0.03
THERMODYNAMICS	13.22	53.33	1.16	1.69	0.00	1.36	1.79	0.00
TOXICOLOGY	17.52	27.73	2.24	1.12	0.00	2.24	1.32	0.00
TRANSPLANTATION	14.53	27.90	1.90	1.24	0.20	1.60	1.64	0.08
TRANSPORTATION SCIENCE & TECHNOLOGY	5.47	14.90	0.63	1.46	0.00	−0.07	2.36	0.61
TROPICAL MEDICINE	14.70	23.07	2.07	1.11	0.01	1.77	1.62	0.07
UROLOGY & NEPHROLOGY	17.73	36.58	2.05	1.29	0.00	1.85	1.79	0.00
VETERINARY SCIENCES	11.13	16.73	1.82	1.09	0.00	1.52	1.79	0.00
VIROLOGY	34.07	49.33	2.96	1.06	0.00	2.86	1.26	0.03
WATER RESOURCES	15.58	29.70	1.98	1.24	0.00	1.58	1.84	0.00
ZOOLOGY	23.93	46.57	2.39	1.25	0.00	2.29	1.55	0.00
**TOTAL**	**2**4.13	**9**3.26	**1**.80	**1**.66	**0**.00	**2**.00	**1**.86	**0**.00

Table S44: Publication year 1985.

Subject-category	$\langle c \rangle$	σ	z	s	p	$\tilde{z}$	$\tilde{s}$	$\tilde{p}$
ACOUSTICS	17.66	34.33	2.09	1.25	0.00	1.89	1.75	0.00
AGRICULTURAL ECONOMICS & POLICY	10.16	17.24	1.64	1.17	0.00	1.24	1.57	0.37
AGRICULTURE, DAIRY & ANIMAL SCIENCE	14.95	22.36	2.12	1.08	0.00	1.72	1.68	0.00
AGRICULTURE, MULTIDISCIPLINARY	11.52	21.57	1.69	1.23	0.00	1.19	1.93	0.00
AGRONOMY	14.13	21.61	2.05	1.10	0.00	1.85	1.50	0.00
ALLERGY	22.90	43.12	2.37	1.23	0.03	2.27	1.53	0.03
ANATOMY & MORPHOLOGY	13.07	15.71	2.12	0.94	0.08	2.12	1.04	0.17
ANDROLOGY	17.12	25.29	2.26	1.08	0.27	2.26	1.17	0.73
ANESTHESIOLOGY	18.01	36.70	2.07	1.28	0.00	1.47	2.18	0.00
ASTRONOMY & ASTROPHYSICS	27.75	50.63	2.59	1.21	0.00	2.39	1.71	0.00
AUTOMATION & CONTROL SYSTEMS	14.66	47.75	1.46	1.57	0.00	0.96	2.17	0.01
BEHAVIORAL SCIENCES	25.85	35.80	2.72	1.03	0.00	2.52	1.54	0.00
BIOCHEMICAL RESEARCH METHODS	23.53	51.13	2.29	1.32	0.00	2.29	1.42	0.00
BIOCHEMISTRY & MOLECULAR BIOLOGY	47.52	213.07	2.34	1.75	0.00	3.04	1.45	0.00
BIODIVERSITY CONSERVATION	13.83	35.51	1.61	1.42	0.00	1.31	2.02	0.00
BIOLOGY	16.65	43.68	1.78	1.44	0.00	1.58	1.84	0.00
BIOPHYSICS	32.43	58.88	2.75	1.21	0.00	2.85	1.21	0.00
BIOTECHNOLOGY & APPLIED MICROBIOLOGY	26.15	46.10	2.56	1.19	0.00	2.66	1.29	0.00
CARDIAC & CARDIOVASCULAR SYSTEMS	28.44	61.64	2.48	1.32	0.00	2.18	1.82	0.00
CELL & TISSUE ENGINEERING	–	–	–	–	–	–	–	–
CELL BIOLOGY	58.12	112.61	3.28	1.25	0.00	3.08	1.55	0.00
CHEMISTRY, ANALYTICAL	19.68	42.38	2.12	1.32	0.00	2.22	1.41	0.00
CHEMISTRY, APPLIED	16.64	26.40	2.18	1.12	0.00	1.98	1.62	0.00
CHEMISTRY, INORGANIC & NUCLEAR	19.82	27.21	2.46	1.03	0.00	2.36	1.33	0.00
CHEMISTRY, MEDICINAL	17.47	27.82	2.23	1.12	0.00	2.23	1.32	0.00
CHEMISTRY, MULTIDISCIPLINARY	23.59	78.98	1.91	1.58	0.00	1.81	1.98	0.00
CHEMISTRY, ORGANIC	20.91	28.86	2.51	1.03	0.00	2.51	1.13	0.00
CHEMISTRY, PHYSICAL	21.32	51.03	2.11	1.38	0.00	2.11	1.68	0.00
CLINICAL NEUROLOGY	30.88	52.04	2.76	1.16	0.00	2.56	1.76	0.00
COMPUTER SCIENCE, ARTIFICIAL INTELLIGENCE	40.84	137.62	2.45	1.58	0.24	2.25	1.89	0.13
COMPUTER SCIENCE, CYBERNETICS	13.50	28.03	1.77	1.29	0.14	1.27	1.99	0.29
COMPUTER SCIENCE, HARDWARE & ARCHITECTURE	13.95	40.01	1.52	1.49	0.00	1.12	2.09	0.04
COMPUTER SCIENCE, INFORMATION SYSTEMS	13.92	61.94	1.12	1.74	0.00	1.22	1.84	0.11
COMPUTER SCIENCE, INTERDISCIPLINARY APPLICATIONS	12.37	57.59	0.95	1.77	0.00	0.85	1.87	0.01
COMPUTER SCIENCE, SOFTWARE ENGINEERING	12.48	34.84	1.44	1.47	0.00	0.84	2.17	0.04
COMPUTER SCIENCE, THEORY & METHODS	12.77	33.38	1.52	1.44	0.00	1.12	1.93	0.06
CONSTRUCTION & BUILDING TECHNOLOGY	9.23	13.99	1.63	1.09	0.00	1.33	1.59	0.03
CRITICAL CARE MEDICINE	17.11	31.19	2.11	1.21	0.00	1.81	1.81	0.00
CRYSTALLOGRAPHY	17.16	263.09	0.11	2.34	0.00	1.11	1.64	0.00
DENTISTRY, ORAL SURGERY & MEDICINE	15.25	25.84	2.05	1.16	0.00	1.55	2.06	0.00
DERMATOLOGY	17.34	25.95	2.27	1.09	0.00	2.07	1.58	0.00
DEVELOPMENTAL BIOLOGY	60.35	80.14	3.59	1.01	0.00	3.39	1.31	0.27
ECOLOGY	30.74	46.84	2.83	1.10	0.00	2.53	1.70	0.00
EDUCATION, SCIENTIFIC DISCIPLINES	7.13	36.89	0.30	1.82	0.00	0.60	1.72	0.08
ELECTROCHEMISTRY	22.53	44.87	2.31	1.26	0.00	2.41	1.37	0.00
EMERGENCY MEDICINE	12.01	24.82	1.66	1.29	0.00	1.56	1.79	0.00
ENDOCRINOLOGY & METABOLISM	33.46	48.72	2.94	1.07	0.00	2.84	1.37	0.00
ENERGY & FUELS	6.96	15.77	1.03	1.35	0.00	0.43	2.25	0.00
ENGINEERING, AEROSPACE	7.98	18.73	1.14	1.37	0.00	0.64	1.97	0.02
ENGINEERING, BIOMEDICAL	19.52	37.06	2.21	1.24	0.14	1.91	1.73	0.00

Table S45: Publication year 1990.

Subject-category	$\langle c\rangle$	σ	z	s	p	$\tilde{z}$	$\tilde{s}$	$\tilde{p}$
ENGINEERING, CHEMICAL	12.97	29.85	1.64	1.36	0.00	1.14	2.16	0.00
ENGINEERING, CIVIL	8.42	17.07	1.32	1.28	0.00	0.82	1.98	0.00
ENGINEERING, ELECTRICAL & ELECTRONIC	14.90	56.53	1.33	1.65	0.00	1.33	1.95	0.00
ENGINEERING, ENVIRONMENTAL	20.44	36.59	2.30	1.20	0.00	1.80	1.90	0.05
ENGINEERING, GEOLOGICAL	17.06	41.81	1.86	1.40	0.00	1.66	1.79	0.16
ENGINEERING, INDUSTRIAL	8.31	18.72	1.22	1.34	0.00	0.72	2.04	0.01
ENGINEERING, MANUFACTURING	9.73	19.80	1.46	1.28	0.00	0.86	2.08	0.11
ENGINEERING, MARINE	0.54	2.55	−2.21	1.78	0.16	−1.61	1.68	0.78
ENGINEERING, MECHANICAL	10.92	19.50	1.67	1.20	0.00	1.37	1.70	0.00
ENGINEERING, MULTIDISCIPLINARY	8.50	24.56	1.02	1.50	0.00	0.32	2.30	0.00
ENGINEERING, OCEAN	12.11	20.37	1.82	1.16	0.05	1.22	1.96	0.53
ENGINEERING, PETROLEUM	1.55	4.97	−0.77	1.56	0.00	−1.47	2.46	0.60
ENTOMOLOGY	11.90	16.71	1.93	1.04	0.00	1.73	1.54	0.00
ENVIRONMENTAL SCIENCES	17.87	32.44	2.15	1.21	0.00	1.85	1.71	0.00
EVOLUTIONARY BIOLOGY	30.87	52.23	2.75	1.16	0.00	2.45	1.86	0.00
FISHERIES	21.47	31.64	2.49	1.07	0.00	2.49	1.27	0.01
FOOD SCIENCE & TECHNOLOGY	17.46	27.02	2.25	1.10	0.00	1.95	1.61	0.00
FORESTRY	16.30	26.02	2.16	1.13	0.00	1.76	1.93	0.00
GASTROENTEROLOGY & HEPATOLOGY	25.14	44.15	2.52	1.19	0.00	2.22	1.79	0.00
GENETICS & HEREDITY	37.06	62.76	2.94	1.16	0.01	2.84	1.36	0.00
GEOCHEMISTRY & GEOPHYSICS	28.51	47.45	2.69	1.15	0.00	2.49	1.45	0.00
GEOGRAPHY, PHYSICAL	18.30	25.62	2.36	1.04	0.00	2.06	1.64	0.01
GEOLOGY	25.70	31.15	2.79	0.95	0.00	2.59	1.35	0.03
GEOSCIENCES, MULTIDISCIPLINARY	19.42	31.70	2.32	1.14	0.00	2.12	1.64	0.00
GERIATRICS & GERONTOLOGY	20.44	37.27	2.29	1.21	0.07	1.89	1.91	0.00
HEALTH CARE SCIENCES & SERVICES	13.14	29.74	1.67	1.35	0.00	1.37	1.85	0.00
HEMATOLOGY	41.52	79.67	2.95	1.24	0.00	2.75	1.64	0.00
HISTORY & PHILOSOPHY OF SCIENCE	6.30	9.88	1.22	1.11	0.01	1.02	1.51	0.14
IMAGING SCIENCE & PHOTOGRAPHIC TECHNOLOGY	25.71	53.94	2.40	1.30	0.54	2.00	1.80	0.18
IMMUNOLOGY	34.43	57.35	2.87	1.15	0.00	2.77	1.45	0.00
INFECTIOUS DISEASES	27.13	40.78	2.71	1.09	0.00	2.41	1.69	0.00
INSTRUMENTS & INSTRUMENTATION	10.51	28.81	1.28	1.46	0.00	1.18	1.86	0.00
INTEGRATIVE & COMPLEMENTARY MEDICINE	9.42	12.16	1.75	0.99	0.02	1.35	1.69	0.14
LIMNOLOGY	42.90	52.51	3.30	0.96	0.00	3.20	1.26	0.07
MARINE & FRESHWATER BIOLOGY	25.14	29.67	2.79	0.93	0.00	2.79	1.14	0.00
MATERIALS SCIENCE, BIOMATERIALS	29.18	45.99	2.75	1.12	0.04	2.55	1.52	0.67
MATERIALS SCIENCE, CERAMICS	25.46	50.42	2.44	1.26	0.00	2.24	1.66	0.00
MATERIALS SCIENCE, CHARACTERIZATION & TESTING	4.44	8.74	0.70	1.26	0.00	0.50	1.56	0.26
MATERIALS SCIENCE, COATINGS & FILMS	20.12	40.64	2.19	1.27	0.00	2.19	1.47	0.01
MATERIALS SCIENCE, COMPOSITES	12.12	20.55	1.82	1.16	0.00	1.02	2.06	0.00
MATERIALS SCIENCE, MULTIDISCIPLINARY	13.33	27.95	1.75	1.30	0.00	1.45	1.80	0.00
MATERIALS SCIENCE, PAPER & WOOD	6.01	13.55	0.89	1.34	0.00	0.49	2.04	0.02
MATERIALS SCIENCE, TEXTILES	6.51	9.10	1.33	1.04	0.00	1.03	1.64	0.06
MATHEMATICAL & COMPUTATIONAL BIOLOGY	23.79	68.50	2.05	1.49	0.00	1.95	1.79	0.02
MATHEMATICS	7.59	16.68	1.15	1.33	0.00	0.75	1.93	0.00
MATHEMATICS, APPLIED	10.86	27.38	1.39	1.41	0.00	1.09	1.81	0.00
MATHEMATICS, INTERDISCIPLINARY APPLICATIONS	20.25	56.39	1.92	1.47	0.00	1.62	1.87	0.00
MECHANICS	15.11	29.58	1.93	1.26	0.00	1.53	1.85	0.00
MEDICAL ETHICS	3.98	9.95	0.39	1.41	0.00	0.19	1.71	0.60
MEDICAL INFORMATICS	17.24	39.76	1.93	1.36	0.24	1.63	1.86	0.22

Table S46: Publication year 1990.

Subject-category	$\langle c \rangle$	σ	z	s	p	$\tilde{z}$	$\tilde{s}$	$\tilde{p}$
MEDICAL LABORATORY TECHNOLOGY	13.83	25.62	1.88	1.22	0.00	1.78	1.52	0.00
MEDICINE, GENERAL & INTERNAL	23.42	89.39	1.78	1.66	0.00	0.78	2.56	0.00
MEDICINE, LEGAL	8.75	17.89	1.35	1.28	0.00	1.35	1.58	0.00
MEDICINE, RESEARCH & EXPERIMENTAL	31.36	74.19	2.50	1.37	0.00	1.80	2.27	0.00
METALLURGY & METALLURGICAL ENGINEERING	7.77	16.14	1.21	1.29	0.00	0.61	2.19	0.00
METEOROLOGY & ATMOSPHERIC SCIENCES	24.77	45.76	2.47	1.22	0.00	2.17	1.82	0.00
MICROBIOLOGY	30.40	60.62	2.61	1.26	0.00	2.71	1.37	0.00
MICROSCOPY	12.76	18.50	1.98	1.06	0.60	1.88	1.46	0.17
MINERALOGY	25.73	40.03	2.63	1.11	0.09	2.63	1.31	0.02
MINING & MINERAL PROCESSING	7.95	13.19	1.41	1.15	0.00	0.81	2.05	0.00
MULTIDISCIPLINARY SCIENCES	67.17	200.71	3.06	1.52	0.00	2.06	2.41	0.00
MYCOLOGY	13.20	26.53	1.77	1.27	0.00	1.77	1.37	0.13
NANOSCIENCE & NANOTECHNOLOGY	17.74	29.53	2.21	1.15	0.04	2.11	1.35	0.26
NEUROIMAGING	25.47	43.49	2.56	1.17	0.03	2.56	1.37	0.20
NEUROSCIENCES	39.73	65.09	3.03	1.14	0.00	2.93	1.54	0.00
NUCLEAR SCIENCE & TECHNOLOGY	8.92	18.14	1.37	1.28	0.00	1.17	1.68	0.00
NURSING	9.46	16.51	1.55	1.18	0.00	0.95	2.08	0.00
NUTRITION & DIETETICS	20.81	37.31	2.32	1.20	0.00	2.12	1.60	0.00
OBSTETRICS & GYNECOLOGY	18.52	28.61	2.31	1.10	0.00	1.91	1.81	0.00
OCEANOGRAPHY	29.91	43.66	2.83	1.07	0.00	2.73	1.47	0.00
ONCOLOGY	33.14	65.52	2.71	1.26	0.00	2.71	1.46	0.00
OPERATIONS RESEARCH & MANAGEMENT SCIENCE	16.99	36.29	1.97	1.31	0.01	1.57	1.81	0.01
OPHTHALMOLOGY	18.29	27.67	2.31	1.09	0.00	2.01	1.69	0.00
OPTICS	18.98	46.25	1.97	1.39	0.00	1.97	1.69	0.00
ORNITHOLOGY	17.14	21.34	2.37	0.97	0.03	2.27	1.27	0.14
ORTHOPEDICS	24.54	44.53	2.47	1.21	0.00	2.17	1.71	0.00
OTORHINOLARYNGOLOGY	16.80	29.82	2.11	1.19	0.00	2.11	1.49	0.00
PALEONTOLOGY	17.83	24.98	2.34	1.04	0.06	2.24	1.34	0.13
PARASITOLOGY	16.24	18.60	2.37	0.92	0.00	2.17	1.32	0.01
PATHOLOGY	24.83	51.04	2.39	1.28	0.00	2.29	1.69	0.00
PEDIATRICS	17.65	29.42	2.21	1.15	0.00	1.91	1.85	0.00
PERIPHERAL VASCULAR DISEASE	39.77	74.01	2.94	1.22	0.00	2.64	1.72	0.00
PHARMACOLOGY & PHARMACY	21.25	37.00	2.36	1.18	0.00	2.36	1.38	0.00
PHYSICS, APPLIED	17.50	67.71	1.48	1.66	0.00	1.88	1.57	0.00
PHYSICS, ATOMIC, MOLECULAR & CHEMICAL	26.35	56.32	2.41	1.31	0.00	2.51	1.41	0.00
PHYSICS, CONDENSED MATTER	21.94	91.55	1.63	1.71	0.00	2.03	1.61	0.00
PHYSICS, FLUIDS & PLASMAS	21.79	35.04	2.44	1.13	0.00	2.14	1.63	0.10
PHYSICS, MATHEMATICAL	17.44	48.72	1.77	1.48	0.00	1.47	1.77	0.01
PHYSICS, MULTIDISCIPLINARY	27.12	86.34	2.10	1.55	0.00	1.70	2.05	0.00
PHYSICS, NUCLEAR	15.38	28.03	2.00	1.21	0.00	1.80	1.51	0.00
PHYSICS, PARTICLES & FIELDS	21.20	47.90	2.15	1.35	0.00	1.75	1.84	0.01
PHYSIOLOGY	33.84	47.69	2.97	1.04	0.00	2.87	1.24	0.00
PLANT SCIENCES	20.37	34.31	2.34	1.16	0.00	2.34	1.36	0.00
POLYMER SCIENCE	22.22	36.49	2.45	1.14	0.00	2.25	1.64	0.00
PRIMARY HEALTH CARE	5.04	12.67	0.62	1.41	0.00	−0.08	2.31	0.10
PSYCHIATRY	31.09	53.09	2.75	1.17	0.00	2.35	1.97	0.00
PSYCHOLOGY	41.47	132.91	2.51	1.56	0.00	2.81	1.46	0.00
PUBLIC, ENVIRONMENTAL & OCCUPATIONAL HEALTH	21.55	38.35	2.36	1.20	0.00	2.06	1.79	0.00
RADIOLOGY, NUCLEAR MEDICINE & MEDICAL IMAGING	21.15	42.63	2.24	1.27	0.00	1.94	1.87	0.00
REHABILITATION	15.53	25.12	2.10	1.14	0.00	1.50	2.03	0.00

Table S47: Publication year 1990.

Subject-category	$\langle c \rangle$	σ	z	s	p	$\tilde{z}$	$\tilde{s}$	$\tilde{p}$
REMOTE SENSING	23.61	45.68	2.38	1.25	0.15	1.98	1.75	0.09
REPRODUCTIVE BIOLOGY	22.08	29.32	2.59	1.01	0.00	2.39	1.51	0.00
RESPIRATORY SYSTEM	18.57	30.16	2.28	1.14	0.00	1.88	1.84	0.00
RHEUMATOLOGY	28.78	100.43	2.07	1.61	0.00	2.17	1.81	0.00
ROBOTICS	17.19	68.34	1.43	1.68	0.34	0.83	2.18	0.83
SOIL SCIENCE	20.33	31.74	2.39	1.11	0.00	2.29	1.41	0.00
SPECTROSCOPY	15.34	33.58	1.85	1.33	0.00	1.85	1.53	0.00
SPORT SCIENCES	24.00	34.35	2.62	1.05	0.00	2.32	1.56	0.00
STATISTICS & PROBABILITY	17.97	71.06	1.48	1.68	0.00	1.38	1.88	0.01
SUBSTANCE ABUSE	28.48	39.40	2.81	1.03	0.38	2.71	1.23	0.22
SURGERY	21.64	38.23	2.37	1.19	0.00	2.17	1.69	0.00
TELECOMMUNICATIONS	14.11	46.60	1.41	1.57	0.00	0.91	2.07	0.07
THERMODYNAMICS	11.37	20.34	1.71	1.20	0.00	1.41	1.80	0.00
TOXICOLOGY	17.76	26.27	2.30	1.08	0.00	2.20	1.38	0.00
TRANSPLANTATION	15.43	24.45	2.11	1.12	0.00	1.81	1.62	0.00
TRANSPORTATION SCIENCE & TECHNOLOGY	9.51	22.64	1.30	1.38	0.00	0.40	2.28	0.54
TROPICAL MEDICINE	12.84	17.29	2.04	1.01	0.00	1.74	1.62	0.00
UROLOGY & NEPHROLOGY	20.06	40.17	2.19	1.27	0.00	1.99	1.77	0.00
VETERINARY SCIENCES	11.75	16.50	1.92	1.04	0.00	1.62	1.74	0.00
VIROLOGY	40.24	48.34	3.25	0.94	0.00	3.15	1.14	0.00
WATER RESOURCES	18.20	31.70	2.20	1.18	0.00	1.90	1.68	0.00
ZOOLOGY	22.57	41.80	2.37	1.22	0.00	2.27	1.42	0.00
**TOTAL**	**2**4.16	**7**5.34	**2**.00	**1**.54	**0**.00	**2**.00	**1**.84	**0**.00

Table S48: Publication year 1990.

Subject-category	$\langle c \rangle$	σ	z	s	p	$\tilde{z}$	$\tilde{s}$	$\tilde{p}$
ACOUSTICS	13.80	23.13	1.96	1.16	0.00	1.76	1.66	0.00
AGRICULTURAL ECONOMICS & POLICY	10.42	15.61	1.76	1.09	0.01	1.56	1.48	0.01
AGRICULTURE, DAIRY & ANIMAL SCIENCE	15.90	25.31	2.14	1.12	0.00	1.84	1.72	0.00
AGRICULTURE, MULTIDISCIPLINARY	14.50	28.34	1.89	1.25	0.00	1.59	1.85	0.00
AGRONOMY	15.42	23.94	2.12	1.11	0.00	1.92	1.51	0.00
ALLERGY	18.94	29.05	2.34	1.10	0.00	2.24	1.50	0.00
ANATOMY & MORPHOLOGY	22.84	106.87	1.56	1.77	0.00	2.26	1.27	0.25
ANDROLOGY	13.07	17.24	2.07	1.00	0.01	1.87	1.70	0.03
ANESTHESIOLOGY	15.86	30.97	1.98	1.25	0.00	1.38	2.15	0.00
ASTRONOMY & ASTROPHYSICS	27.15	58.10	2.44	1.31	0.00	2.14	1.91	0.00
AUTOMATION & CONTROL SYSTEMS	14.84	37.26	1.70	1.41	0.00	1.30	1.91	0.00
BEHAVIORAL SCIENCES	26.57	33.86	2.80	0.98	0.00	2.70	1.18	0.00
BIOCHEMICAL RESEARCH METHODS	20.90	45.45	2.17	1.32	0.00	2.37	1.32	0.00
BIOCHEMISTRY & MOLECULAR BIOLOGY	43.96	95.07	2.92	1.32	0.00	3.02	1.32	0.00
BIODIVERSITY CONSERVATION	20.16	32.60	2.36	1.13	0.00	1.86	2.03	0.00
BIOLOGY	15.92	36.15	1.86	1.35	0.00	1.66	1.75	0.00
BIOPHYSICS	30.02	45.49	2.81	1.09	0.00	2.81	1.19	0.00
BIOTECHNOLOGY & APPLIED MICROBIOLOGY	21.17	33.14	2.43	1.11	0.00	2.33	1.51	0.00
CARDIAC & CARDIOVASCULAR SYSTEMS	28.18	54.75	2.56	1.25	0.00	2.26	1.85	0.00
CELL & TISSUE ENGINEERING	17.02	26.64	2.22	1.11	0.18	2.22	1.31	0.60
CELL BIOLOGY	54.26	107.66	3.20	1.26	0.00	3.10	1.56	0.00
CHEMISTRY, ANALYTICAL	19.87	32.25	2.34	1.14	0.00	2.24	1.44	0.00
CHEMISTRY, APPLIED	14.35	24.89	1.97	1.18	0.00	1.67	1.88	0.00
CHEMISTRY, INORGANIC & NUCLEAR	18.87	23.94	2.46	0.98	0.00	2.46	1.18	0.00
CHEMISTRY, MEDICINAL	17.22	28.41	2.19	1.14	0.00	2.19	1.24	0.00
CHEMISTRY, MULTIDISCIPLINARY	20.47	48.71	2.07	1.38	0.00	1.67	1.98	0.00
CHEMISTRY, ORGANIC	19.03	23.76	2.48	0.97	0.00	2.48	1.07	0.00
CHEMISTRY, PHYSICAL	21.55	35.83	2.41	1.15	0.00	2.31	1.35	0.00
CLINICAL NEUROLOGY	24.92	44.16	2.51	1.19	0.00	2.01	2.09	0.00
COMPUTER SCIENCE, ARTIFICIAL INTELLIGENCE	22.83	94.49	1.68	1.70	0.00	1.68	1.90	0.00
COMPUTER SCIENCE, CYBERNETICS	10.59	35.95	1.10	1.59	0.00	0.70	2.19	0.27
COMPUTER SCIENCE, HARDWARE & ARCHITECTURE	15.40	51.37	1.49	1.58	0.00	1.09	2.08	0.04
COMPUTER SCIENCE, INFORMATION SYSTEMS	12.08	60.80	0.86	1.81	0.00	0.96	1.81	0.02
COMPUTER SCIENCE, INTERDISCIPLINARY APPLICATIONS	12.61	50.89	1.11	1.69	0.00	1.21	1.69	0.00
COMPUTER SCIENCE, SOFTWARE ENGINEERING	12.13	35.09	1.38	1.50	0.00	0.88	2.10	0.03
COMPUTER SCIENCE, THEORY & METHODS	14.68	46.00	1.50	1.54	0.00	1.30	1.84	0.00
CONSTRUCTION & BUILDING TECHNOLOGY	10.32	14.78	1.78	1.06	0.00	1.38	1.75	0.00
CRITICAL CARE MEDICINE	28.32	56.21	2.54	1.26	0.00	2.24	1.86	0.00
CRYSTALLOGRAPHY	11.70	84.17	0.48	1.99	0.00	1.38	1.49	0.00
DENTISTRY, ORAL SURGERY & MEDICINE	14.93	23.56	2.08	1.12	0.00	1.68	1.92	0.00
DERMATOLOGY	15.45	23.76	2.13	1.10	0.00	1.83	1.80	0.00
DEVELOPMENTAL BIOLOGY	62.67	107.81	3.45	1.17	0.00	3.35	1.37	0.05
ECOLOGY	29.42	44.39	2.79	1.09	0.00	2.69	1.49	0.00
EDUCATION, SCIENTIFIC DISCIPLINES	5.45	11.93	0.82	1.33	0.00	0.52	1.73	0.20
ELECTROCHEMISTRY	20.49	34.06	2.36	1.15	0.00	2.16	1.55	0.00
EMERGENCY MEDICINE	11.16	17.66	1.79	1.12	0.00	1.29	1.92	0.00
ENDOCRINOLOGY & METABOLISM	30.50	45.27	2.84	1.08	0.00	2.84	1.28	0.00
ENERGY & FUELS	9.14	16.41	1.49	1.20	0.00	0.89	2.10	0.00
ENGINEERING, AEROSPACE	5.86	11.73	0.96	1.27	0.00	0.56	1.87	0.00
ENGINEERING, BIOMEDICAL	19.23	34.69	2.23	1.20	0.00	2.03	1.70	0.00

Table S49: Publication year 1995.

Subject-category	$\langle c \rangle$	σ	z	s	p	$\tilde{z}$	$\tilde{s}$	$\tilde{p}$
ENGINEERING, CHEMICAL	12.71	23.54	1.80	1.22	0.00	1.30	2.02	0.00
ENGINEERING, CIVIL	8.73	16.31	1.42	1.22	0.00	0.92	1.93	0.00
ENGINEERING, ELECTRICAL & ELECTRONIC	13.78	40.66	1.49	1.51	0.00	1.29	1.91	0.00
ENGINEERING, ENVIRONMENTAL	22.69	33.92	2.54	1.08	0.00	2.34	1.58	0.00
ENGINEERING, GEOLOGICAL	11.69	19.09	1.81	1.14	0.03	1.61	1.44	0.04
ENGINEERING, INDUSTRIAL	8.94	15.24	1.51	1.17	0.00	1.21	1.77	0.00
ENGINEERING, MANUFACTURING	8.20	11.50	1.56	1.04	0.00	1.26	1.64	0.00
ENGINEERING, MARINE	0.56	2.12	−1.94	1.65	0.00	−2.04	2.25	0.92
ENGINEERING, MECHANICAL	9.62	14.99	1.65	1.11	0.00	1.25	1.81	0.00
ENGINEERING, MULTIDISCIPLINARY	8.92	20.59	1.27	1.36	0.00	0.77	2.06	0.00
ENGINEERING, OCEAN	10.29	22.26	1.46	1.32	0.00	0.86	2.12	0.23
ENGINEERING, PETROLEUM	1.43	4.39	−0.81	1.53	0.00	−1.01	2.03	0.81
ENTOMOLOGY	11.68	15.34	1.96	1.00	0.00	1.66	1.70	0.00
ENVIRONMENTAL SCIENCES	21.14	35.05	2.39	1.15	0.00	2.29	1.55	0.00
EVOLUTIONARY BIOLOGY	36.39	70.19	2.82	1.24	0.00	2.82	1.45	0.00
FISHERIES	19.02	21.84	2.52	0.92	0.00	2.42	1.22	0.00
FOOD SCIENCE & TECHNOLOGY	17.32	25.53	2.27	1.08	0.00	2.07	1.47	0.00
FORESTRY	16.41	23.58	2.24	1.06	0.00	2.04	1.56	0.00
GASTROENTEROLOGY & HEPATOLOGY	25.86	42.92	2.59	1.15	0.00	2.29	1.65	0.00
GENETICS & HEREDITY	41.55	86.94	2.89	1.30	0.00	2.89	1.40	0.00
GEOCHEMISTRY & GEOPHYSICS	29.80	51.99	2.70	1.18	0.00	2.80	1.28	0.00
GEOGRAPHY, PHYSICAL	22.36	32.03	2.55	1.06	0.01	2.45	1.36	0.01
GEOLOGY	22.97	31.38	2.61	1.02	0.00	2.31	1.62	0.00
GEOSCIENCES, MULTIDISCIPLINARY	17.70	27.42	2.26	1.10	0.00	1.86	1.81	0.00
GERIATRICS & GERONTOLOGY	19.73	33.97	2.29	1.17	0.00	1.89	1.87	0.00
HEALTH CARE SCIENCES & SERVICES	15.18	30.15	1.92	1.26	0.00	1.52	1.87	0.00
HEMATOLOGY	39.10	67.28	2.98	1.17	0.00	2.78	1.57	0.00
HISTORY & PHILOSOPHY OF SCIENCE	5.68	10.62	0.98	1.22	0.03	0.78	1.53	0.20
IMAGING SCIENCE & PHOTOGRAPHIC TECHNOLOGY	20.55	32.75	2.39	1.12	0.00	1.99	1.72	0.11
IMMUNOLOGY	33.10	64.60	2.71	1.25	0.00	2.71	1.45	0.00
INFECTIOUS DISEASES	26.36	36.72	2.73	1.04	0.00	2.53	1.54	0.00
INSTRUMENTS & INSTRUMENTATION	10.88	24.13	1.50	1.33	0.00	1.40	1.73	0.00
INTEGRATIVE & COMPLEMENTARY MEDICINE	13.32	16.00	2.14	0.94	0.00	1.84	1.55	0.03
LIMNOLOGY	32.45	40.41	3.01	0.97	0.00	2.91	1.17	0.28
MARINE & FRESHWATER BIOLOGY	21.83	25.21	2.66	0.92	0.00	2.66	1.12	0.00
MATERIALS SCIENCE, BIOMATERIALS	26.37	38.85	2.70	1.07	0.21	2.60	1.17	0.19
MATERIALS SCIENCE, CERAMICS	13.89	25.36	1.90	1.21	0.00	1.40	2.11	0.00
MATERIALS SCIENCE, CHARACTERIZATION & TESTING	3.69	7.99	0.44	1.32	0.00	0.04	1.92	0.09
MATERIALS SCIENCE, COATINGS & FILMS	19.43	32.83	2.29	1.16	0.00	2.29	1.36	0.00
MATERIALS SCIENCE, COMPOSITES	9.57	12.02	1.79	0.97	0.00	1.39	1.67	0.01
MATERIALS SCIENCE, MULTIDISCIPLINARY	14.32	27.09	1.90	1.23	0.00	1.70	1.63	0.00
MATERIALS SCIENCE, PAPER & WOOD	6.59	11.11	1.21	1.16	0.00	0.81	1.86	0.00
MATERIALS SCIENCE, TEXTILES	6.19	10.08	1.17	1.14	0.00	0.77	1.84	0.04
MATHEMATICAL & COMPUTATIONAL BIOLOGY	21.34	53.32	2.07	1.41	0.00	2.07	1.61	0.01
MATHEMATICS	7.20	15.71	1.10	1.32	0.00	0.90	1.72	0.00
MATHEMATICS, APPLIED	10.04	24.35	1.34	1.39	0.00	1.14	1.79	0.00
MATHEMATICS, INTERDISCIPLINARY APPLICATIONS	14.94	38.39	1.69	1.42	0.00	1.59	1.72	0.00
MECHANICS	13.63	28.62	1.77	1.30	0.00	1.67	1.60	0.00
MEDICAL ETHICS	7.52	11.29	1.43	1.09	0.00	0.93	1.89	0.16
MEDICAL INFORMATICS	16.28	42.37	1.76	1.43	0.00	1.56	1.83	0.02

Table S50: Publication year 1995.

Subject-category	$\langle c \rangle$	σ	z	s	p	$\tilde{z}$	$\tilde{s}$	$\tilde{p}$
MEDICAL LABORATORY TECHNOLOGY	16.72	35.40	1.97	1.30	0.00	1.87	1.60	0.00
MEDICINE, GENERAL & INTERNAL	25.13	106.06	1.76	1.71	0.00	0.76	2.41	0.02
MEDICINE, LEGAL	10.81	14.20	1.88	1.00	0.00	1.58	1.50	0.00
MEDICINE, RESEARCH & EXPERIMENTAL	37.40	86.49	2.70	1.36	0.00	2.10	2.16	0.00
METALLURGY & METALLURGICAL ENGINEERING	9.90	16.14	1.64	1.14	0.00	1.14	1.94	0.00
METEOROLOGY & ATMOSPHERIC SCIENCES	21.23	40.11	2.30	1.23	0.00	2.00	1.93	0.00
MICROBIOLOGY	29.31	60.90	2.54	1.29	0.00	2.74	1.39	0.00
MICROSCOPY	15.26	20.89	2.20	1.03	0.23	2.10	1.33	0.09
MINERALOGY	17.93	24.66	2.36	1.03	0.00	2.26	1.43	0.00
MINING & MINERAL PROCESSING	8.94	14.37	1.55	1.13	0.00	1.15	1.93	0.00
MULTIDISCIPLINARY SCIENCES	78.23	203.64	3.33	1.43	0.00	2.33	2.33	0.00
MYCOLOGY	13.68	42.01	1.44	1.53	0.00	1.74	1.43	0.09
NANOSCIENCE & NANOTECHNOLOGY	18.28	42.25	1.98	1.36	0.00	1.98	1.56	0.00
NEUROIMAGING	27.73	68.27	2.35	1.40	0.00	2.45	1.40	0.00
NEUROSCIENCES	36.53	58.90	2.96	1.13	0.00	2.96	1.33	0.00
NUCLEAR SCIENCE & TECHNOLOGY	7.85	17.35	1.17	1.33	0.00	0.97	1.73	0.00
NURSING	9.67	15.56	1.63	1.13	0.00	1.23	1.83	0.00
NUTRITION & DIETETICS	21.85	33.47	2.48	1.10	0.00	2.18	1.60	0.00
OBSTETRICS & GYNECOLOGY	18.46	29.26	2.29	1.12	0.00	1.99	1.72	0.00
OCEANOGRAPHY	29.04	39.88	2.84	1.03	0.00	2.74	1.43	0.00
ONCOLOGY	33.17	56.34	2.82	1.17	0.00	2.62	1.57	0.00
OPERATIONS RESEARCH & MANAGEMENT SCIENCE	12.76	23.98	1.79	1.23	0.00	1.49	1.63	0.00
OPHTHALMOLOGY	19.98	35.94	2.27	1.20	0.00	2.07	1.70	0.00
OPTICS	15.82	35.96	1.85	1.35	0.00	1.65	1.75	0.00
ORNITHOLOGY	14.61	17.03	2.25	0.93	0.02	2.15	1.22	0.03
ORTHOPEDICS	22.14	36.15	2.45	1.14	0.00	1.95	2.04	0.00
OTORHINOLARYNGOLOGY	13.38	18.47	2.06	1.03	0.00	1.76	1.63	0.00
PALEONTOLOGY	17.06	26.93	2.21	1.12	0.01	2.11	1.42	0.01
PARASITOLOGY	16.62	19.96	2.36	0.94	0.00	2.26	1.24	0.01
PATHOLOGY	22.20	38.05	2.41	1.17	0.00	2.21	1.67	0.00
PEDIATRICS	16.31	29.57	2.06	1.21	0.00	1.76	1.81	0.00
PERIPHERAL VASCULAR DISEASE	38.57	67.86	2.95	1.19	0.00	2.65	1.79	0.00
PHARMACOLOGY & PHARMACY	19.55	31.51	2.33	1.13	0.00	2.23	1.33	0.00
PHYSICS, APPLIED	15.76	31.43	1.96	1.26	0.00	1.86	1.57	0.00
PHYSICS, ATOMIC, MOLECULAR & CHEMICAL	22.88	59.22	2.11	1.43	0.00	2.41	1.33	0.00
PHYSICS, CONDENSED MATTER	17.65	37.70	2.01	1.31	0.00	1.91	1.61	0.00
PHYSICS, FLUIDS & PLASMAS	22.31	42.72	2.33	1.24	0.00	2.33	1.44	0.00
PHYSICS, MATHEMATICAL	18.56	52.87	1.82	1.49	0.00	1.82	1.69	0.00
PHYSICS, MULTIDISCIPLINARY	26.09	76.18	2.13	1.50	0.00	1.73	2.10	0.00
PHYSICS, NUCLEAR	13.57	42.97	1.41	1.55	0.00	1.61	1.55	0.00
PHYSICS, PARTICLES & FIELDS	17.69	46.10	1.85	1.43	0.00	1.45	2.03	0.00
PHYSIOLOGY	29.20	35.08	2.93	0.94	0.00	2.93	1.14	0.00
PLANT SCIENCES	21.66	34.05	2.45	1.11	0.00	2.35	1.41	0.00
POLYMER SCIENCE	19.74	41.95	2.13	1.31	0.00	2.23	1.41	0.00
PRIMARY HEALTH CARE	6.08	12.57	0.97	1.29	0.00	0.27	2.19	0.03
PSYCHIATRY	27.93	56.42	2.52	1.28	0.00	2.22	1.88	0.00
PSYCHOLOGY	33.57	53.37	2.88	1.12	0.00	2.88	1.22	0.01
PUBLIC, ENVIRONMENTAL & OCCUPATIONAL HEALTH	22.45	40.00	2.40	1.20	0.00	2.10	1.79	0.00
RADIOLOGY, NUCLEAR MEDICINE & MEDICAL IMAGING	20.36	43.85	2.15	1.32	0.00	1.95	1.81	0.00
REHABILITATION	15.55	22.38	2.18	1.06	0.00	1.78	1.86	0.00

Table S51: Publication year 1995.

Subject-category	$\langle c \rangle$	σ	z	s	p	$\tilde{z}$	$\tilde{s}$	$\tilde{p}$
REMOTE SENSING	21.77	34.22	2.46	1.11	0.00	2.16	1.62	0.20
REPRODUCTIVE BIOLOGY	23.92	35.20	2.60	1.07	0.00	2.50	1.47	0.00
RESPIRATORY SYSTEM	25.11	43.50	2.53	1.18	0.00	2.23	1.78	0.00
RHEUMATOLOGY	25.88	61.74	2.30	1.38	0.00	2.20	1.78	0.00
ROBOTICS	11.89	20.18	1.80	1.17	0.18	1.40	1.76	0.12
SOIL SCIENCE	19.39	29.35	2.37	1.09	0.00	2.17	1.59	0.00
SPECTROSCOPY	14.71	80.83	0.97	1.85	0.00	1.77	1.46	0.00
SPORT SCIENCES	21.88	30.45	2.55	1.04	0.00	2.35	1.64	0.00
STATISTICS & PROBABILITY	16.57	51.45	1.63	1.54	0.00	1.43	1.84	0.00
SUBSTANCE ABUSE	25.86	34.17	2.75	1.00	0.01	2.65	1.40	0.00
SURGERY	18.01	32.55	2.17	1.20	0.00	1.77	2.00	0.00
TELECOMMUNICATIONS	12.85	49.53	1.17	1.66	0.00	0.67	2.26	0.00
THERMODYNAMICS	11.15	16.29	1.84	1.07	0.00	1.64	1.47	0.00
TOXICOLOGY	17.79	23.35	2.38	1.00	0.00	2.38	1.20	0.00
TRANSPLANTATION	15.75	29.49	2.00	1.23	0.00	1.80	1.63	0.00
TRANSPORTATION SCIENCE & TECHNOLOGY	12.32	33.26	1.45	1.45	0.00	0.95	2.05	0.21
TROPICAL MEDICINE	14.00	19.46	2.10	1.04	0.00	1.90	1.54	0.00
UROLOGY & NEPHROLOGY	22.61	34.72	2.51	1.10	0.00	2.21	1.80	0.00
VETERINARY SCIENCES	11.86	18.55	1.85	1.11	0.00	1.55	1.71	0.00
VIROLOGY	36.13	46.21	3.10	0.98	0.00	3.10	1.09	0.00
WATER RESOURCES	17.63	25.33	2.31	1.06	0.00	2.11	1.56	0.00
ZOOLOGY	19.21	27.70	2.39	1.06	0.00	2.19	1.46	0.00
**TOTAL**	22.97	55.82	2.17	1.39	0.00	2.07	1.79	0.00

Table S52: Publication year 1995.

Subject-category	$\langle c \rangle$	σ	z	s	p	$\tilde{z}$	$\tilde{s}$	$\tilde{p}$
ACOUSTICS	12.82	18.77	1.98	1.07	0.00	1.78	1.57	0.00
AGRICULTURAL ECONOMICS & POLICY	8.69	11.76	1.64	1.02	0.01	1.34	1.52	0.02
AGRICULTURE, DAIRY & ANIMAL SCIENCE	12.65	17.75	1.99	1.04	0.00	1.59	1.74	0.00
AGRICULTURE, MULTIDISCIPLINARY	17.34	29.81	2.17	1.17	0.00	1.97	1.67	0.00
AGRONOMY	15.62	21.02	2.23	1.01	0.00	2.13	1.32	0.00
ALLERGY	20.13	28.87	2.44	1.06	0.00	2.24	1.46	0.00
ANATOMY & MORPHOLOGY	16.06	22.60	2.23	1.04	0.17	2.13	1.45	0.01
ANDROLOGY	15.24	17.08	2.32	0.90	0.30	2.22	1.20	0.74
ANESTHESIOLOGY	15.04	26.11	2.02	1.18	0.00	1.52	2.08	0.00
ASTRONOMY & ASTROPHYSICS	29.09	56.27	2.59	1.25	0.00	2.49	1.65	0.00
AUTOMATION & CONTROL SYSTEMS	14.80	33.39	1.79	1.35	0.00	1.59	1.84	0.00
BEHAVIORAL SCIENCES	24.91	27.12	2.82	0.88	0.00	2.82	0.98	0.00
BIOCHEMICAL RESEARCH METHODS	23.20	70.69	1.98	1.53	0.00	2.48	1.22	0.00
BIOCHEMISTRY & MOLECULAR BIOLOGY	40.22	71.21	2.98	1.19	0.00	2.98	1.19	0.00
BIODIVERSITY CONSERVATION	20.31	31.23	2.40	1.10	0.00	2.00	1.80	0.00
BIOLOGY	17.16	30.55	2.13	1.20	0.00	1.83	1.69	0.00
BIOPHYSICS	28.16	56.17	2.54	1.27	0.00	2.84	1.07	0.00
BIOTECHNOLOGY & APPLIED MICROBIOLOGY	24.43	55.57	2.29	1.35	0.00	2.39	1.55	0.00
CARDIAC & CARDIOVASCULAR SYSTEMS	26.36	52.83	2.47	1.27	0.00	2.17	1.87	0.00
CELL & TISSUE ENGINEERING	14.40	19.57	2.14	1.02	0.25	1.94	1.62	0.17
CELL BIOLOGY	49.82	86.73	3.21	1.18	0.00	3.11	1.38	0.00
CHEMISTRY, ANALYTICAL	19.14	41.02	2.09	1.31	0.00	2.29	1.41	0.00
CHEMISTRY, APPLIED	16.95	28.06	2.17	1.15	0.00	1.87	1.85	0.00
CHEMISTRY, INORGANIC & NUCLEAR	15.43	22.00	2.18	1.05	0.00	1.98	1.55	0.00
CHEMISTRY, MEDICINAL	18.51	25.30	2.39	1.02	0.00	2.39	1.13	0.00
CHEMISTRY, MULTIDISCIPLINARY	23.40	42.97	2.41	1.22	0.00	2.01	1.92	0.00
CHEMISTRY, ORGANIC	18.34	29.08	2.28	1.12	0.00	2.38	1.22	0.00
CHEMISTRY, PHYSICAL	22.17	37.34	2.43	1.16	0.00	2.43	1.36	0.00
CLINICAL NEUROLOGY	24.96	43.49	2.52	1.18	0.00	2.32	1.68	0.00
COMPUTER SCIENCE, ARTIFICIAL INTELLIGENCE	21.75	56.79	2.05	1.44	0.00	1.95	1.83	0.00
COMPUTER SCIENCE, CYBERNETICS	11.51	20.51	1.73	1.20	0.00	1.13	2.10	0.01
COMPUTER SCIENCE, HARDWARE & ARCHITECTURE	15.32	51.74	1.47	1.59	0.00	1.17	1.99	0.00
COMPUTER SCIENCE, INFORMATION SYSTEMS	14.90	72.55	1.10	1.79	0.00	1.10	1.89	0.00
COMPUTER SCIENCE, INTERDISCIPLINARY APPLICATIONS	12.86	35.82	1.47	1.47	0.00	1.37	1.77	0.00
COMPUTER SCIENCE, SOFTWARE ENGINEERING	11.57	36.48	1.25	1.55	0.00	0.95	2.05	0.00
COMPUTER SCIENCE, THEORY & METHODS	15.32	49.65	1.51	1.56	0.00	1.41	1.86	0.00
CONSTRUCTION & BUILDING TECHNOLOGY	10.07	14.91	1.73	1.08	0.00	1.43	1.68	0.00
CRITICAL CARE MEDICINE	28.14	45.77	2.69	1.14	0.00	2.39	1.74	0.00
CRYSTALLOGRAPHY	12.60	92.45	0.53	2.00	0.00	1.43	1.50	0.00
DENTISTRY, ORAL SURGERY & MEDICINE	14.53	20.49	2.13	1.04	0.00	1.83	1.75	0.00
DERMATOLOGY	13.60	20.42	2.02	1.09	0.00	1.82	1.59	0.00
DEVELOPMENTAL BIOLOGY	52.41	70.14	3.45	1.01	0.00	3.35	1.21	0.20
ECOLOGY	27.51	36.61	2.80	1.01	0.00	2.80	1.21	0.00
EDUCATION, SCIENTIFIC DISCIPLINES	7.02	13.91	1.15	1.26	0.00	0.75	1.76	0.01
ELECTROCHEMISTRY	20.65	30.03	2.46	1.07	0.00	2.26	1.57	0.00
EMERGENCY MEDICINE	10.61	17.17	1.72	1.14	0.00	1.32	1.73	0.00
ENDOCRINOLOGY & METABOLISM	31.05	50.24	2.79	1.14	0.00	2.79	1.33	0.00
ENERGY & FUELS	11.07	26.12	1.46	1.37	0.00	1.16	2.07	0.00
ENGINEERING, AEROSPACE	5.23	10.40	0.85	1.26	0.00	0.35	1.96	0.00
ENGINEERING, BIOMEDICAL	21.73	41.28	2.31	1.24	0.00	2.21	1.64	0.00

Table S53: Publication year 1999.

Subject-category	$\langle c \rangle$	σ	z	s	p	$\tilde{z}$	$\tilde{s}$	$\tilde{p}$
ENGINEERING, CHEMICAL	13.72	26.11	1.85	1.24	0.00	1.55	1.84	0.00
ENGINEERING, CIVIL	9.51	14.93	1.63	1.11	0.00	1.13	1.92	0.00
ENGINEERING, ELECTRICAL & ELECTRONIC	14.47	44.62	1.50	1.53	0.00	1.40	1.83	0.00
ENGINEERING, ENVIRONMENTAL	21.19	28.76	2.53	1.02	0.00	2.33	1.42	0.00
ENGINEERING, GEOLOGICAL	11.54	15.56	1.93	1.02	0.04	1.73	1.42	0.01
ENGINEERING, INDUSTRIAL	9.46	14.46	1.64	1.10	0.00	1.24	1.70	0.00
ENGINEERING, MANUFACTURING	9.74	14.56	1.69	1.08	0.00	1.39	1.68	0.00
ENGINEERING, MARINE	0.56	2.49	−2.08	1.74	0.00	−2.08	2.24	0.91
ENGINEERING, MECHANICAL	9.59	17.17	1.54	1.20	0.00	1.24	1.80	0.00
ENGINEERING, MULTIDISCIPLINARY	9.19	24.17	1.18	1.44	0.00	0.88	1.94	0.00
ENGINEERING, OCEAN	9.58	14.33	1.67	1.08	0.00	1.17	1.88	0.01
ENGINEERING, PETROLEUM	2.57	6.16	−0.01	1.38	0.00	−0.51	2.08	0.14
ENTOMOLOGY	10.63	13.21	1.90	0.96	0.00	1.60	1.57	0.00
ENVIRONMENTAL SCIENCES	21.40	30.57	2.51	1.05	0.00	2.41	1.35	0.00
EVOLUTIONARY BIOLOGY	34.59	66.06	2.78	1.24	0.00	2.98	1.24	0.00
FISHERIES	17.47	20.28	2.43	0.92	0.00	2.43	1.12	0.00
FOOD SCIENCE & TECHNOLOGY	17.70	24.81	2.33	1.04	0.00	2.23	1.34	0.00
FORESTRY	16.84	21.46	2.34	0.98	0.00	2.14	1.48	0.00
GASTROENTEROLOGY & HEPATOLOGY	24.34	43.72	2.47	1.20	0.00	2.27	1.70	0.00
GENETICS & HEREDITY	38.87	73.26	2.90	1.23	0.00	2.90	1.33	0.00
GEOCHEMISTRY & GEOPHYSICS	26.40	34.69	2.77	1.00	0.00	2.77	1.20	0.00
GEOGRAPHY, PHYSICAL	21.07	26.35	2.58	0.97	0.00	2.58	1.17	0.00
GEOLOGY	18.52	23.63	2.44	0.98	0.00	2.24	1.48	0.00
GEOSCIENCES, MULTIDISCIPLINARY	18.51	26.10	2.37	1.04	0.00	2.17	1.45	0.00
GERIATRICS & GERONTOLOGY	20.09	28.69	2.44	1.05	0.00	2.24	1.56	0.00
HEALTH CARE SCIENCES & SERVICES	14.34	25.33	1.96	1.19	0.00	1.66	1.79	0.00
HEMATOLOGY	36.66	64.14	2.90	1.18	0.00	2.70	1.58	0.00
HISTORY & PHILOSOPHY OF SCIENCE	4.62	8.31	0.81	1.20	0.00	0.61	1.60	0.27
IMAGING SCIENCE & PHOTOGRAPHIC TECHNOLOGY	25.69	57.02	2.36	1.33	0.01	2.16	1.73	0.03
IMMUNOLOGY	30.87	53.15	2.74	1.17	0.00	2.74	1.37	0.00
INFECTIOUS DISEASES	25.36	36.36	2.67	1.06	0.00	2.57	1.46	0.00
INSTRUMENTS & INSTRUMENTATION	10.52	17.69	1.68	1.16	0.00	1.38	1.76	0.00
INTEGRATIVE & COMPLEMENTARY MEDICINE	13.02	18.01	2.03	1.03	0.00	1.63	1.93	0.00
LIMNOLOGY	25.29	30.99	2.77	0.96	0.00	2.77	1.16	0.04
MARINE & FRESHWATER BIOLOGY	18.73	21.97	2.50	0.93	0.00	2.50	1.13	0.00
MATERIALS SCIENCE, BIOMATERIALS	31.26	49.51	2.81	1.12	0.19	2.81	1.32	0.17
MATERIALS SCIENCE, CERAMICS	11.99	23.96	1.68	1.27	0.00	1.38	1.97	0.00
MATERIALS SCIENCE, CHARACTERIZATION & TESTING	3.13	7.40	0.20	1.37	0.00	−0.30	2.07	0.16
MATERIALS SCIENCE, COATINGS & FILMS	16.95	25.42	2.24	1.09	0.00	2.14	1.39	0.00
MATERIALS SCIENCE, COMPOSITES	10.24	14.75	1.76	1.06	0.00	1.56	1.56	0.00
MATERIALS SCIENCE, MULTIDISCIPLINARY	16.13	33.42	1.95	1.29	0.00	1.75	1.69	0.00
MATERIALS SCIENCE, PAPER & WOOD	6.24	8.84	1.28	1.05	0.00	0.88	1.65	0.00
MATERIALS SCIENCE, TEXTILES	5.63	9.88	1.03	1.19	0.00	0.63	1.88	0.01
MATHEMATICAL & COMPUTATIONAL BIOLOGY	23.04	66.07	2.03	1.49	0.00	2.23	1.39	0.00
MATHEMATICS	6.44	12.16	1.10	1.23	0.00	0.90	1.63	0.00
MATHEMATICS, APPLIED	9.47	22.26	1.31	1.37	0.00	1.21	1.67	0.00
MATHEMATICS, INTERDISCIPLINARY APPLICATIONS	13.82	33.55	1.66	1.39	0.00	1.66	1.59	0.00
MECHANICS	13.03	24.19	1.82	1.22	0.00	1.72	1.52	0.00
MEDICAL ETHICS	7.62	13.98	1.29	1.21	0.00	1.09	1.71	0.10
MEDICAL INFORMATICS	13.49	36.15	1.55	1.45	0.00	1.35	1.95	0.00

Table S54: Publication year 1999.

Subject-category	$\langle c \rangle$	σ	z	s	p	$\tilde{z}$	$\tilde{s}$	$\tilde{p}$
MEDICAL LABORATORY TECHNOLOGY	14.16	23.27	2.00	1.14	0.00	1.90	1.54	0.00
MEDICINE, GENERAL & INTERNAL	25.43	97.70	1.86	1.66	0.00	0.96	2.46	0.01
MEDICINE, LEGAL	8.41	11.29	1.61	1.01	0.00	1.31	1.62	0.00
MEDICINE, RESEARCH & EXPERIMENTAL	32.63	74.67	2.57	1.35	0.01	2.17	1.85	0.00
METALLURGY & METALLURGICAL ENGINEERING	11.48	23.36	1.62	1.28	0.00	1.22	1.98	0.00
METEOROLOGY & ATMOSPHERIC SCIENCES	23.96	37.17	2.56	1.11	0.00	2.36	1.51	0.00
MICROBIOLOGY	28.06	39.05	2.80	1.04	0.00	2.70	1.24	0.00
MICROSCOPY	12.22	20.12	1.85	1.14	0.00	1.45	1.94	0.00
MINERALOGY	20.57	46.41	2.12	1.35	0.00	2.32	1.35	0.00
MINING & MINERAL PROCESSING	8.51	14.65	1.45	1.17	0.00	1.05	1.87	0.00
MULTIDISCIPLINARY SCIENCES	70.71	174.44	3.28	1.40	0.00	2.28	2.30	0.00
MYCOLOGY	14.80	28.84	1.91	1.25	0.00	1.91	1.45	0.01
NANOSCIENCE & NANOTECHNOLOGY	21.37	44.02	2.23	1.29	0.01	2.13	1.59	0.00
NEUROIMAGING	34.15	71.03	2.69	1.29	0.00	2.69	1.39	0.02
NEUROSCIENCES	35.12	51.93	2.98	1.08	0.00	2.98	1.28	0.00
NUCLEAR SCIENCE & TECHNOLOGY	8.08	13.41	1.43	1.15	0.00	1.23	1.55	0.00
NURSING	8.83	11.39	1.69	0.99	0.00	1.29	1.69	0.00
NUTRITION & DIETETICS	22.37	32.79	2.53	1.07	0.00	2.33	1.57	0.00
OBSTETRICS & GYNECOLOGY	16.47	22.39	2.28	1.02	0.00	2.08	1.62	0.00
OCEANOGRAPHY	23.41	25.72	2.76	0.89	0.00	2.66	1.19	0.00
ONCOLOGY	32.14	57.94	2.75	1.20	0.00	2.75	1.40	0.00
OPERATIONS RESEARCH & MANAGEMENT SCIENCE	14.24	30.81	1.79	1.32	0.00	1.79	1.52	0.00
OPHTHALMOLOGY	18.02	28.47	2.27	1.12	0.00	1.97	1.72	0.00
OPTICS	15.59	27.98	2.03	1.20	0.00	1.73	1.70	0.00
ORNITHOLOGY	15.62	65.83	1.28	1.71	0.00	2.08	1.21	0.02
ORTHOPEDICS	18.77	26.48	2.38	1.05	0.00	1.98	1.85	0.00
OTORHINOLARYNGOLOGY	12.96	16.60	2.08	0.98	0.00	1.98	1.28	0.00
PALEONTOLOGY	16.27	20.30	2.32	0.97	0.00	2.12	1.37	0.02
PARASITOLOGY	15.52	21.07	2.22	1.02	0.00	2.22	1.12	0.02
PATHOLOGY	21.26	46.69	2.18	1.33	0.00	2.18	1.63	0.00
PEDIATRICS	14.91	24.31	2.05	1.14	0.00	1.75	1.64	0.00
PERIPHERAL VASCULAR DISEASE	36.95	69.57	2.85	1.23	0.00	2.65	1.63	0.00
PHARMACOLOGY & PHARMACY	19.90	28.87	2.42	1.06	0.00	2.32	1.36	0.00
PHYSICS, APPLIED	16.52	34.47	1.97	1.30	0.00	1.87	1.59	0.00
PHYSICS, ATOMIC, MOLECULAR & CHEMICAL	21.36	35.21	2.40	1.14	0.00	2.50	1.14	0.00
PHYSICS, CONDENSED MATTER	16.06	59.48	1.43	1.64	0.00	1.73	1.74	0.00
PHYSICS, FLUIDS & PLASMAS	18.85	28.15	2.35	1.08	0.00	2.25	1.38	0.00
PHYSICS, MATHEMATICAL	15.62	32.88	1.90	1.30	0.00	1.70	1.70	0.00
PHYSICS, MULTIDISCIPLINARY	23.03	73.25	1.93	1.55	0.00	1.63	1.95	0.00
PHYSICS, NUCLEAR	12.43	22.40	1.80	1.20	0.00	1.60	1.60	0.00
PHYSICS, PARTICLES & FIELDS	21.17	51.38	2.09	1.39	0.00	1.99	1.69	0.00
PHYSIOLOGY	27.12	29.67	2.91	0.89	0.00	2.81	1.09	0.00
PLANT SCIENCES	19.90	28.28	2.44	1.05	0.00	2.34	1.35	0.00
POLYMER SCIENCE	18.00	30.75	2.21	1.17	0.00	2.11	1.37	0.00
PRIMARY HEALTH CARE	7.43	14.27	1.23	1.24	0.00	0.53	2.14	0.00
PSYCHIATRY	29.65	48.60	2.74	1.14	0.00	2.54	1.64	0.00
PSYCHOLOGY	27.39	35.81	2.81	1.00	0.00	2.71	1.20	0.00
PUBLIC, ENVIRONMENTAL & OCCUPATIONAL HEALTH	19.99	32.65	2.35	1.14	0.00	2.05	1.74	0.00
RADIOLOGY, NUCLEAR MEDICINE & MEDICAL IMAGING	19.94	42.63	2.13	1.31	0.00	2.03	1.71	0.00
REHABILITATION	16.11	25.41	2.15	1.12	0.00	1.85	1.72	0.00

Table S55: Publication year 1999.

Subject-category	$\langle c \rangle$	σ	z	s	p	$\tilde{z}$	$\tilde{s}$	$\tilde{p}$
REMOTE SENSING	22.32	32.74	2.53	1.07	0.01	2.33	1.47	0.00
REPRODUCTIVE BIOLOGY	23.39	27.95	2.71	0.94	0.00	2.61	1.24	0.00
RESPIRATORY SYSTEM	23.44	38.54	2.50	1.14	0.00	2.20	1.84	0.00
RHEUMATOLOGY	23.40	42.96	2.42	1.21	0.00	2.12	1.81	0.00
ROBOTICS	11.78	19.05	1.82	1.14	0.01	1.42	1.83	0.05
SOIL SCIENCE	18.42	22.48	2.46	0.95	0.00	2.36	1.36	0.00
SPECTROSCOPY	13.40	32.58	1.63	1.39	0.00	1.73	1.49	0.00
SPORT SCIENCES	19.17	26.22	2.43	1.02	0.00	2.13	1.73	0.00
STATISTICS & PROBABILITY	13.00	33.93	1.54	1.44	0.00	1.44	1.73	0.00
SUBSTANCE ABUSE	23.46	28.59	2.70	0.95	0.00	2.70	1.15	0.05
SURGERY	16.72	28.37	2.14	1.17	0.00	1.84	1.76	0.00
TELECOMMUNICATIONS	13.33	47.42	1.28	1.62	0.00	0.68	2.32	0.00
THERMODYNAMICS	11.49	18.89	1.79	1.14	0.00	1.79	1.35	0.00
TOXICOLOGY	19.46	27.75	2.41	1.05	0.00	2.41	1.15	0.00
TRANSPLANTATION	14.23	25.88	1.93	1.21	0.00	1.73	1.61	0.00
TRANSPORTATION SCIENCE & TECHNOLOGY	12.53	22.56	1.81	1.20	0.00	1.41	1.90	0.00
TROPICAL MEDICINE	14.28	18.27	2.17	0.98	0.00	1.97	1.48	0.00
UROLOGY & NEPHROLOGY	21.29	39.33	2.32	1.22	0.00	2.12	1.72	0.00
VETERINARY SCIENCES	11.17	16.37	1.84	1.07	0.00	1.64	1.67	0.00
VIROLOGY	32.74	39.60	3.04	0.95	0.00	3.04	1.05	0.00
WATER RESOURCES	17.59	23.96	2.34	1.02	0.00	2.24	1.42	0.00
ZOOLOGY	17.34	23.51	2.33	1.02	0.00	2.23	1.22	0.00
**TOTAL**	21.97	49.06	2.19	1.34	0.00	2.09	1.74	0.00

Table S56: Publication year 1999.

Subject-category	$\langle c \rangle$	σ	z	s	p	$\tilde{z}$	$\tilde{s}$	$\tilde{p}$
ACOUSTICS	8.78	12.02	1.64	1.03	0.00	1.44	1.33	0.00
AGRICULTURAL ECONOMICS & POLICY	7.87	8.31	1.69	0.87	0.00	1.59	1.17	0.03
AGRICULTURE, DAIRY & ANIMAL SCIENCE	8.99	11.61	1.71	0.99	0.00	1.41	1.59	0.00
AGRICULTURE, MULTIDISCIPLINARY	12.07	16.68	1.96	1.03	0.00	1.76	1.53	0.00
AGRONOMY	10.00	13.82	1.77	1.03	0.00	1.67	1.33	0.00
ALLERGY	18.97	26.59	2.40	1.04	0.00	2.30	1.34	0.00
ANATOMY & MORPHOLOGY	11.79	15.76	1.96	1.01	0.01	1.86	1.21	0.01
ANDROLOGY	11.46	12.55	2.04	0.89	0.02	2.04	1.09	0.12
ANESTHESIOLOGY	10.06	16.76	1.64	1.15	0.00	1.14	1.95	0.00
ASTRONOMY & ASTROPHYSICS	21.41	38.53	2.34	1.20	0.00	2.34	1.40	0.00
AUTOMATION & CONTROL SYSTEMS	12.91	29.20	1.65	1.35	0.00	1.45	1.75	0.00
BEHAVIORAL SCIENCES	16.95	17.16	2.48	0.84	0.00	2.48	0.94	0.00
BIOCHEMICAL RESEARCH METHODS	20.54	106.59	1.36	1.82	0.00	2.26	1.13	0.00
BIOCHEMISTRY & MOLECULAR BIOLOGY	26.18	52.63	2.46	1.27	0.00	2.76	1.07	0.00
BIODIVERSITY CONSERVATION	14.03	19.32	2.11	1.03	0.00	1.71	1.73	0.00
BIOLOGY	16.13	28.66	2.07	1.20	0.00	1.77	1.69	0.00
BIOPHYSICS	19.48	67.74	1.68	1.60	0.00	2.48	1.10	0.00
BIOTECHNOLOGY & APPLIED MICROBIOLOGY	19.63	77.20	1.58	1.67	0.00	2.28	1.27	0.00
CARDIAC & CARDIOVASCULAR SYSTEMS	20.21	40.31	2.20	1.27	0.00	2.00	1.77	0.00
CELL & TISSUE ENGINEERING	31.40	42.38	2.93	1.02	0.02	2.73	1.32	0.84
CELL BIOLOGY	32.72	49.31	2.90	1.09	0.00	2.80	1.19	0.00
CHEMISTRY, ANALYTICAL	15.04	20.35	2.19	1.02	0.00	2.09	1.32	0.00
CHEMISTRY, APPLIED	11.76	15.77	1.95	1.01	0.00	1.75	1.51	0.00
CHEMISTRY, INORGANIC & NUCLEAR	12.33	15.74	2.03	0.98	0.00	1.93	1.38	0.00
CHEMISTRY, MEDICINAL	14.62	19.02	2.19	0.99	0.00	2.29	0.99	0.00
CHEMISTRY, MULTIDISCIPLINARY	21.38	40.73	2.30	1.24	0.00	1.90	1.84	0.00
CHEMISTRY, ORGANIC	14.56	17.46	2.23	0.94	0.00	2.23	1.14	0.00
CHEMISTRY, PHYSICAL	18.52	30.50	2.26	1.14	0.00	2.26	1.24	0.00
CLINICAL NEUROLOGY	16.95	24.50	2.27	1.06	0.00	2.07	1.56	0.00
COMPUTER SCIENCE, ARTIFICIAL INTELLIGENCE	16.77	67.60	1.40	1.69	0.00	1.70	1.59	0.00
COMPUTER SCIENCE, CYBERNETICS	10.42	17.38	1.68	1.15	0.00	1.28	1.75	0.00
COMPUTER SCIENCE, HARDWARE & ARCHITECTURE	9.83	22.85	1.36	1.36	0.00	1.06	1.86	0.00
COMPUTER SCIENCE, INFORMATION SYSTEMS	11.58	44.23	1.08	1.66	0.00	1.18	1.76	0.00
COMPUTER SCIENCE, INTERDISCIPLINARY APPLICATIONS	10.25	17.01	1.67	1.15	0.00	1.47	1.55	0.00
COMPUTER SCIENCE, SOFTWARE ENGINEERING	8.85	18.78	1.33	1.30	0.00	1.03	1.81	0.00
COMPUTER SCIENCE, THEORY & METHODS	9.59	20.47	1.40	1.31	0.00	1.20	1.61	0.00
CONSTRUCTION & BUILDING TECHNOLOGY	7.36	9.60	1.50	0.99	0.00	1.20	1.60	0.00
CRITICAL CARE MEDICINE	18.15	30.29	2.23	1.15	0.00	1.93	1.85	0.00
CRYSTALLOGRAPHY	8.10	75.93	−0.15	2.12	0.00	0.85	1.62	0.00
DENTISTRY, ORAL SURGERY & MEDICINE	11.11	16.74	1.82	1.09	0.00	1.72	1.59	0.00
DERMATOLOGY	10.08	13.33	1.80	1.00	0.00	1.50	1.61	0.00
DEVELOPMENTAL BIOLOGY	31.01	41.21	2.93	1.01	0.00	2.93	1.11	0.08
ECOLOGY	18.02	21.70	2.44	0.95	0.00	2.44	1.24	0.00
EDUCATION, SCIENTIFIC DISCIPLINES	6.32	12.06	1.08	1.24	0.00	0.88	1.54	0.00
ELECTROCHEMISTRY	17.34	22.32	2.36	0.99	0.00	2.26	1.29	0.00
EMERGENCY MEDICINE	7.59	12.42	1.38	1.14	0.00	1.08	1.64	0.00
ENDOCRINOLOGY & METABOLISM	21.68	36.95	2.40	1.17	0.00	2.50	1.27	0.00
ENERGY & FUELS	11.90	19.92	1.81	1.16	0.00	1.41	1.95	0.00
ENGINEERING, AEROSPACE	4.70	8.01	0.87	1.17	0.00	0.57	1.67	0.01
ENGINEERING, BIOMEDICAL	18.82	25.18	2.42	1.01	0.00	2.32	1.31	0.00

Table S57: Publication year 2004.

Subject-category	$\langle c \rangle$	σ	z	s	p	$\tilde{z}$	$\tilde{s}$	$\tilde{p}$
ENGINEERING, CHEMICAL	10.78	16.21	1.79	1.09	0.00	1.49	1.69	0.00
ENGINEERING, CIVIL	7.00	11.36	1.30	1.14	0.00	0.90	1.73	0.00
ENGINEERING, ELECTRICAL & ELECTRONIC	11.32	29.08	1.41	1.42	0.00	1.31	1.72	0.00
ENGINEERING, ENVIRONMENTAL	16.39	23.38	2.24	1.05	0.00	2.14	1.35	0.00
ENGINEERING, GEOLOGICAL	7.08	9.86	1.42	1.04	0.00	1.32	1.44	0.00
ENGINEERING, INDUSTRIAL	8.28	10.86	1.61	1.00	0.00	1.41	1.50	0.00
ENGINEERING, MANUFACTURING	8.28	11.17	1.59	1.02	0.00	1.39	1.42	0.00
ENGINEERING, MARINE	1.06	3.27	−1.13	1.54	0.00	−1.53	2.34	0.33
ENGINEERING, MECHANICAL	7.54	11.09	1.44	1.07	0.00	1.14	1.67	0.00
ENGINEERING, MULTIDISCIPLINARY	7.30	12.04	1.33	1.14	0.00	0.93	1.75	0.00
ENGINEERING, OCEAN	7.50	13.00	1.32	1.18	0.00	1.02	1.68	0.01
ENGINEERING, PETROLEUM	2.14	4.55	−0.09	1.31	0.00	−0.49	2.01	0.09
ENTOMOLOGY	7.76	8.93	1.63	0.92	0.00	1.43	1.42	0.00
ENVIRONMENTAL SCIENCES	14.88	19.95	2.19	1.01	0.00	2.09	1.32	0.00
EVOLUTIONARY BIOLOGY	22.88	29.08	2.65	0.98	0.00	2.75	0.98	0.00
FISHERIES	10.79	10.86	2.03	0.84	0.00	2.03	1.03	0.00
FOOD SCIENCE & TECHNOLOGY	11.97	14.66	2.02	0.96	0.00	1.92	1.36	0.00
FORESTRY	11.42	13.84	1.98	0.95	0.00	1.88	1.25	0.00
GASTROENTEROLOGY & HEPATOLOGY	19.03	30.82	2.30	1.14	0.00	2.10	1.63	0.00
GENETICS & HEREDITY	25.56	43.16	2.57	1.16	0.00	2.67	1.16	0.00
GEOCHEMISTRY & GEOPHYSICS	15.79	31.09	1.97	1.26	0.00	2.17	1.26	0.00
GEOGRAPHY, PHYSICAL	14.60	21.12	2.12	1.06	0.00	2.22	1.16	0.00
GEOLOGY	12.42	16.08	2.03	0.99	0.00	1.93	1.39	0.00
GEOSCIENCES, MULTIDISCIPLINARY	11.71	16.21	1.92	1.03	0.00	1.82	1.33	0.00
GERIATRICS & GERONTOLOGY	15.10	19.40	2.23	0.98	0.00	2.03	1.59	0.00
HEALTH CARE SCIENCES & SERVICES	11.78	18.65	1.84	1.12	0.00	1.74	1.52	0.00
HEMATOLOGY	25.88	45.76	2.54	1.19	0.00	2.34	1.59	0.00
HISTORY & PHILOSOPHY OF SCIENCE	4.18	7.61	0.70	1.21	0.00	0.50	1.51	0.10
IMAGING SCIENCE & PHOTOGRAPHIC TECHNOLOGY	16.28	25.75	2.16	1.12	0.01	1.86	1.62	0.00
IMMUNOLOGY	22.17	34.49	2.48	1.11	0.00	2.48	1.31	0.00
INFECTIOUS DISEASES	18.47	26.71	2.35	1.06	0.00	2.35	1.26	0.00
INSTRUMENTS & INSTRUMENTATION	8.28	13.74	1.45	1.15	0.00	1.25	1.55	0.00
INTEGRATIVE & COMPLEMENTARY MEDICINE	10.45	11.01	1.97	0.87	0.00	1.77	1.36	0.00
LIMNOLOGY	13.27	15.46	2.16	0.93	0.00	2.06	1.33	0.00
MARINE & FRESHWATER BIOLOGY	12.33	11.89	2.18	0.81	0.00	2.18	1.01	0.00
MATERIALS SCIENCE, BIOMATERIALS	23.02	28.18	2.68	0.96	0.00	2.68	1.16	0.01
MATERIALS SCIENCE, CERAMICS	7.87	11.12	1.51	1.05	0.00	1.31	1.65	0.00
MATERIALS SCIENCE, CHARACTERIZATION & TESTING	4.59	8.17	0.81	1.20	0.00	0.51	1.59	0.04
MATERIALS SCIENCE, COATINGS & FILMS	11.03	13.54	1.94	0.96	0.00	1.84	1.26	0.00
MATERIALS SCIENCE, COMPOSITES	9.44	15.20	1.61	1.13	0.00	1.51	1.43	0.00
MATERIALS SCIENCE, MULTIDISCIPLINARY	13.68	25.74	1.86	1.23	0.00	1.66	1.53	0.00
MATERIALS SCIENCE, PAPER & WOOD	4.67	6.58	0.99	1.04	0.00	0.79	1.55	0.00
MATERIALS SCIENCE, TEXTILES	5.72	11.00	0.97	1.24	0.00	0.77	1.54	0.01
MATHEMATICAL & COMPUTATIONAL BIOLOGY	20.05	43.32	2.13	1.32	0.00	2.23	1.32	0.01
MATHEMATICS	4.61	8.58	0.78	1.22	0.00	0.68	1.52	0.00
MATHEMATICS, APPLIED	6.65	12.71	1.13	1.24	0.00	1.03	1.54	0.00
MATHEMATICS, INTERDISCIPLINARY APPLICATIONS	10.37	19.46	1.58	1.23	0.00	1.48	1.53	0.00
MECHANICS	9.49	13.84	1.68	1.07	0.00	1.48	1.47	0.00
MEDICAL ETHICS	6.27	9.54	1.24	1.10	0.00	0.84	1.69	0.17
MEDICAL INFORMATICS	11.66	19.14	1.80	1.14	0.03	1.70	1.44	0.02

Table S58: Publication year 2004.

Subject-category	$\langle c \rangle$	σ	z	s	p	$\tilde{z}$	$\tilde{s}$	$\tilde{p}$
MEDICAL LABORATORY TECHNOLOGY	11.13	17.04	1.81	1.10	0.00	1.61	1.50	0.00
MEDICINE, GENERAL & INTERNAL	19.97	84.77	1.52	1.72	0.00	0.92	2.12	0.00
MEDICINE, LEGAL	7.02	10.16	1.38	1.06	0.00	0.98	1.66	0.00
MEDICINE, RESEARCH & EXPERIMENTAL	20.29	44.48	2.13	1.33	0.00	2.03	1.62	0.00
METALLURGY & METALLURGICAL ENGINEERING	8.12	13.88	1.41	1.17	0.00	1.11	1.77	0.00
METEOROLOGY & ATMOSPHERIC SCIENCES	15.86	21.70	2.24	1.03	0.00	2.14	1.33	0.00
MICROBIOLOGY	19.84	23.71	2.54	0.94	0.00	2.54	1.04	0.00
MICROSCOPY	9.90	13.14	1.78	1.01	0.00	1.58	1.31	0.02
MINERALOGY	10.95	13.14	1.95	0.94	0.00	1.85	1.35	0.00
MINING & MINERAL PROCESSING	6.01	9.48	1.17	1.12	0.00	0.77	1.82	0.00
MULTIDISCIPLINARY SCIENCES	48.85	110.03	2.99	1.34	0.00	1.99	2.24	0.00
MYCOLOGY	10.45	15.32	1.77	1.07	0.00	1.77	1.27	0.01
NANOSCIENCE & NANOTECHNOLOGY	20.63	36.07	2.33	1.18	0.00	2.13	1.58	0.00
NEUROIMAGING	25.95	34.69	2.74	1.01	0.00	2.64	1.31	0.01
NEUROSCIENCES	23.48	31.27	2.65	1.01	0.00	2.65	1.11	0.00
NUCLEAR SCIENCE & TECHNOLOGY	6.03	10.61	1.09	1.19	0.00	0.99	1.49	0.00
NURSING	7.86	16.59	1.21	1.30	0.00	1.21	1.70	0.00
NUTRITION & DIETETICS	18.44	25.43	2.38	1.03	0.00	2.28	1.33	0.00
OBSTETRICS & GYNECOLOGY	11.78	19.40	1.81	1.14	0.00	1.71	1.55	0.00
OCEANOGRAPHY	13.66	15.12	2.21	0.89	0.00	2.21	1.10	0.00
ONCOLOGY	23.44	39.33	2.48	1.16	0.00	2.48	1.36	0.00
OPERATIONS RESEARCH & MANAGEMENT SCIENCE	10.69	15.73	1.79	1.07	0.00	1.69	1.37	0.00
OPHTHALMOLOGY	11.97	19.12	1.85	1.13	0.00	1.65	1.53	0.00
OPTICS	12.16	19.35	1.87	1.12	0.00	1.67	1.62	0.00
ORNITHOLOGY	8.36	11.28	1.61	1.02	0.00	1.51	1.32	0.00
ORTHOPEDICS	13.00	18.67	2.01	1.06	0.00	1.81	1.56	0.00
OTORHINOLARYNGOLOGY	9.01	10.45	1.77	0.92	0.00	1.57	1.42	0.00
PALEONTOLOGY	9.70	11.22	1.85	0.92	0.00	1.75	1.32	0.00
PARASITOLOGY	11.06	13.19	1.96	0.94	0.00	1.96	1.14	0.00
PATHOLOGY	14.42	19.37	2.15	1.01	0.00	1.95	1.52	0.00
PEDIATRICS	10.69	16.21	1.77	1.09	0.00	1.47	1.59	0.00
PERIPHERAL VASCULAR DISEASE	25.41	45.74	2.51	1.20	0.00	2.41	1.60	0.00
PHARMACOLOGY & PHARMACY	14.65	17.58	2.24	0.94	0.00	2.14	1.24	0.00
PHYSICS, APPLIED	14.24	27.63	1.88	1.25	0.00	1.78	1.55	0.00
PHYSICS, ATOMIC, MOLECULAR & CHEMICAL	14.32	20.54	2.10	1.06	0.00	2.10	1.16	0.00
PHYSICS, CONDENSED MATTER	13.39	27.74	1.76	1.29	0.00	1.56	1.59	0.00
PHYSICS, FLUIDS & PLASMAS	12.31	17.96	1.94	1.07	0.00	1.94	1.27	0.00
PHYSICS, MATHEMATICAL	11.38	20.47	1.71	1.20	0.00	1.61	1.50	0.00
PHYSICS, MULTIDISCIPLINARY	16.17	33.00	1.96	1.28	0.00	1.56	1.88	0.00
PHYSICS, NUCLEAR	10.24	17.27	1.65	1.16	0.00	1.45	1.56	0.00
PHYSICS, PARTICLES & FIELDS	14.33	26.23	1.93	1.21	0.00	1.63	1.71	0.00
PHYSIOLOGY	18.73	19.82	2.55	0.87	0.00	2.55	0.96	0.00
PLANT SCIENCES	15.45	23.54	2.14	1.10	0.00	2.14	1.30	0.00
POLYMER SCIENCE	14.16	18.65	2.15	1.00	0.00	2.05	1.30	0.00
PRIMARY HEALTH CARE	6.93	11.16	1.30	1.13	0.00	0.70	2.03	0.00
PSYCHIATRY	20.88	27.61	2.53	1.00	0.00	2.33	1.51	0.00
PSYCHOLOGY	17.93	21.34	2.45	0.94	0.00	2.45	1.14	0.00
PUBLIC, ENVIRONMENTAL & OCCUPATIONAL HEALTH	15.31	22.65	2.15	1.08	0.00	2.05	1.48	0.00
RADIOLOGY, NUCLEAR MEDICINE & MEDICAL IMAGING	15.78	23.34	2.18	1.08	0.00	1.98	1.58	0.00
REHABILITATION	11.45	13.36	2.01	0.93	0.00	1.81	1.43	0.00

Table S59: Publication year 2004.

Subject-category	$\langle c\rangle$	σ	z	s	p	$\tilde{z}$	$\tilde{s}$	$\tilde{p}$
REMOTE SENSING	14.94	22.64	2.11	1.09	0.07	1.91	1.49	0.01
REPRODUCTIVE BIOLOGY	15.26	23.22	2.13	1.10	0.00	2.23	1.30	0.00
RESPIRATORY SYSTEM	16.34	24.94	2.19	1.10	0.00	1.99	1.70	0.00
RHEUMATOLOGY	18.28	29.21	2.27	1.13	0.00	1.97	1.73	0.00
ROBOTICS	11.17	17.35	1.80	1.11	0.02	1.50	1.61	0.04
SOIL SCIENCE	11.08	12.70	1.99	0.92	0.00	1.89	1.32	0.00
SPECTROSCOPY	9.56	13.38	1.71	1.04	0.00	1.51	1.44	0.00
SPORT SCIENCES	13.44	16.51	2.14	0.96	0.00	2.04	1.36	0.00
STATISTICS & PROBABILITY	9.04	21.90	1.24	1.39	0.00	1.24	1.49	0.00
SUBSTANCE ABUSE	16.99	21.11	2.37	0.96	0.00	2.37	1.17	0.01
SURGERY	12.25	19.60	1.87	1.13	0.00	1.67	1.63	0.00
TELECOMMUNICATIONS	9.97	26.84	1.24	1.45	0.00	0.84	2.05	0.00
THERMODYNAMICS	9.76	13.53	1.74	1.03	0.00	1.64	1.33	0.00
TOXICOLOGY	13.82	18.32	2.12	1.00	0.00	2.22	1.11	0.00
TRANSPLANTATION	13.21	19.69	2.00	1.08	0.00	1.90	1.48	0.00
TRANSPORTATION SCIENCE & TECHNOLOGY	5.44	11.31	0.86	1.29	0.00	0.36	1.99	0.05
TROPICAL MEDICINE	11.39	13.41	2.00	0.93	0.00	1.80	1.43	0.00
UROLOGY & NEPHROLOGY	15.58	23.99	2.14	1.10	0.00	1.94	1.60	0.00
VETERINARY SCIENCES	7.64	10.73	1.49	1.04	0.00	1.29	1.54	0.00
VIROLOGY	21.66	23.79	2.68	0.89	0.00	2.68	0.99	0.00
WATER RESOURCES	10.86	13.89	1.90	0.98	0.00	1.80	1.39	0.00
ZOOLOGY	10.07	13.12	1.81	0.99	0.00	1.61	1.50	0.00
**TOTAL**	**1**5.79	**3**4.42	**1**.88	**1**.32	**0**.00	**1**.88	**1**.62	**0**.00

Table S60: Publication year 2004.
